# Design, Synthesis, and Evaluation of Novel Δ^2^-Thiazolino 2-Pyridone Derivatives That Potentiate Isoniazid Activity in an Isoniazid-Resistant *Mycobacterium tuberculosis* Mutant

**DOI:** 10.1021/acs.jmedchem.3c00358

**Published:** 2023-07-24

**Authors:** Souvik Sarkar, Anne E. Mayer Bridwell, James A. D. Good, Erin R. Wang, Samuel R. McKee, Joy Valenta, Gregory A. Harrison, Kelly N. Flentie, Frederick L. Henry, Torbjörn Wixe, Peter Demirel, Siva K. Vagolu, Jonathan Chatagnon, Arnaud Machelart, Priscille Brodin, Tone Tønjum, Christina L. Stallings, Fredrik Almqvist

**Affiliations:** † Department of Chemistry, Umeå University, SE-90187 Umeå, Sweden; ‡ Department of Molecular Microbiology, Center for Women’s Infectious Disease Research, Washington University School of Medicine, St. Louis, 63110 Missouri, United States; § Department of Microbiology, University of Oslo, N-0316 Oslo, Norway; ∥ University Lille, CNRS, INSERM, CHU Lille, Institut Pasteur de Lille, U1019-UMR 9017-CIIL-Center for Infection and Immunity of Lille, 59000 Lille, France; ⊥ Oslo University Hospital, N-0424 Oslo, Norway

## Abstract

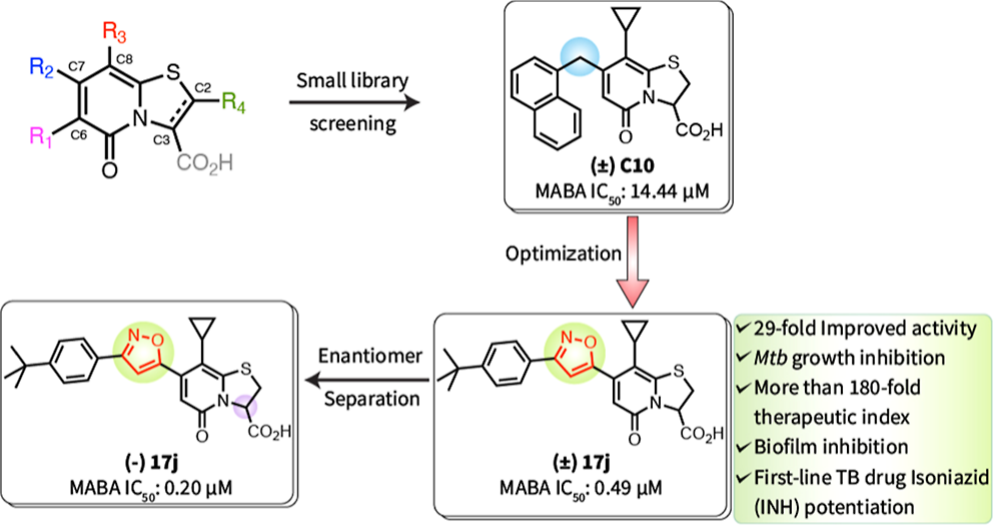

*Mycobacterium tuberculosis* (*Mtb*) drug resistance poses an alarming threat to global tuberculosis control. We previously reported that **C10**, a ring-fused thiazolo-2-pyridone, inhibits *Mtb* respiration, blocks biofilm formation, and restores the activity of the antibiotic isoniazid (INH) in INH-resistant *Mtb* isolates. This discovery revealed a new strategy to address INH resistance. Expanding upon this strategy, we identified C10 analogues with improved potency and drug-like properties. By exploring three heterocycle spacers (oxadiazole, 1,2,3-triazole, and isoxazole) on the ring-fused thiazolo-2-pyridone scaffold, we identified two novel isoxazoles, **17h** and **17j**. **17h** and **17j** inhibited *Mtb* respiration and biofilm formation more potently with a broader therapeutic window, were better potentiators of INH-mediated inhibition of an INH-resistant *Mtb* mutant, and more effectively inhibited intracellular *Mtb* replication than **C10**. The **(−)17j** enantiomer showed further enhanced activity compared to its enantiomer and the **17j** racemic mixture. Our potent second-generation **C10** analogues offer promise for therapeutic development against drug-resistant *Mtb*.

## Introduction

*Mycobacterium tuberculosis* (*Mtb*), the causative agent of tuberculosis (TB), is the deadliest pathogen in the world. In 2020, 1.5 million deaths worldwide were due to TB,^1^ a number that represents an increase for the first time in over a decade. The COVID-19 pandemic has disrupted access to TB resources, interventions, and research. Without renewed efforts and commitment to improving TB treatment, the global impact of this ancient disease will continue to expand.

*Mtb* has co-evolved with humans for thousands of years^2^ to be able to persist in the face of host immune defenses. When confronted with the stresses imposed by the host immune response, including reactive oxygen species, low pH, and hypoxia, *Mtb* enters a physiologically stress-tolerant state that also renders *Mtb* antibiotic-tolerant, which contributes to the need for drug regimens of long duration to treat TB. The standard of care for drug-sensitive active TB disease involves isoniazid (INH) and rifampicin (RIF) taken in combination with ethambutol and pyrazinamide for two months, followed by a continuation phase of INH and RIF for an additional four months.^3^ This complex and lengthy TB drug regimen can have toxic side effects^4^ and poses significant challenges to patient adherence. Thus, it creates an opportunity for selection of drug-resistant (DR), multidrug-resistant (MDR, resistant to INH and RIF), or extensively drug-resistant (XDR) *Mtb* mutants.^5−8^ Much effort has been devoted to identifying shorter and more highly effective drug treatments. In fact, recently, the WHO put forth a conditional recommendation for a shorter four-month regimen consisting of 8 weeks of intensive treatment with INH, rifapentine, moxifloxacin, and pyrazinamide followed by a 9 week continuation phase of treatment with INH, rifapentine, and moxifloxacin.^3^ Challenges of this new regimen include a higher pill burden despite the shorter regimen and high cost due to the inclusion of pyrazinamide. There is a dire need for further drug discovery and TB treatment optimization.

INH is on the WHO’s list of essential medicines for the treatment of TB for both children and adults, is prescribed exclusively to treat TB, and is safe for use during pregnancy and HIV co-infection.^9,10^ Unfortunately, between 2002 and 2018, an estimated 10.7% of new TB cases and 27.2% of previously treated TB cases were INH-resistant,^11^ threatening the utility of this frontline antibiotic in the treatment of TB. INH is a prodrug that is activated by the bacterial enzyme KatG to INH-NAD, which rapidly kills *Mtb.* Multiple targets and mechanisms of action for INH-NAD have been identified,^12,13^ but the most thoroughly studied is inhibition of InhA, an enzyme required for mycolic acid synthesis.^12,13^ The majority of resistant clinical isolates have mutations in the *katG* gene that decrease the ability of the KatG enzyme to convert INH to its active form.^14,15^

**Figure 1:**
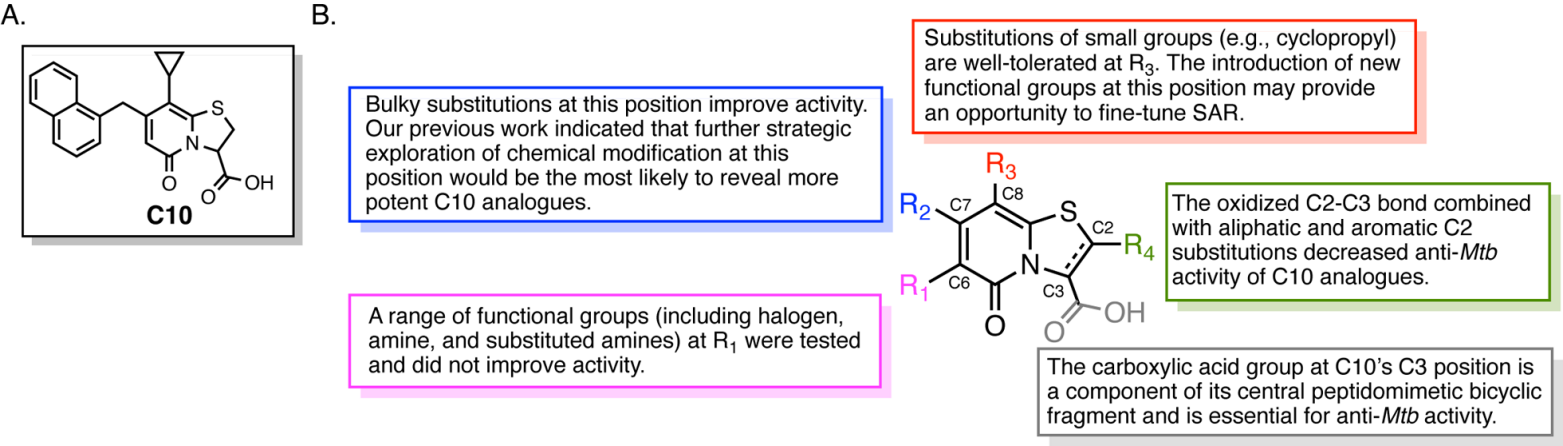
(A) Structure of C10. (B) SAR summary for the ring-fused thiazolo-2-pyridone compounds in our first-generation screening library.

We previously reported the discovery of the ring-fused thiazolo-2-pyridone compound **C10** (Figure 1A) that inhibits *Mtb* respiration, blocks biofilm formation, sensitizes *Mtb* to stresses encountered during infection, potentiates the activity of INH, prevents the selection of INH resistant mutants, and restores INH activity in INH-resistant *katG* mutants.^16^ Those findings represented the first evidence that INH resistance can be reversed in clinically relevant INH-resistant mutants and revealed a new strategy for TB drug development that could be used for the treatment of drug-resistant TB. Starting with **C10** as our first-generation ring-fused thiazolo-2-pyridone hit, here we describe our efforts to increase the potency of this new class of anti-TB ring-fused thiazolo-2-pyridone compounds while preserving their biofilm inhibition and INH potentiation behaviors. Our findings reveal two potent novel isoxazole-ring-fused thiazolo-2-pyridone derivatives, **17h** and **17j**, which were 20-fold and 29-fold more potent at inhibiting *Mtb* respiration and growth than **C10**, respectively. These two second-generation compounds improve upon **C10**’s ability to chemically disarm INH-resistant *Mtb* while also exhibiting favorable toxicity profiles in a human lung epithelial cell line. In addition, we provide the first evidence that the ring-fused thiazolo-2-pyridones can inhibit intracellular *Mtb* growth in macrophages, where the novel isoxazole-ring-fused thiazolo-2-pyridone derivatives **17h** and **17j** were more potent than **C10**. We further assessed **17j**, the more potent of these two compounds, by separating the enantiomers. **(−)17j** enantiomer was the most active component compared to the **17j** racemic mixture and **(+)17j** enantiomer. Our findings present a promising advance in our ability to develop a new class of inhibitors for the treatment of drug-sensitive and drug-resistant TB.

## Results and Discussion

### Chemistry

**C10** was first identified in a screen of a small library of thiazolo ring-fused 2-pyridones for inhibitors of *Mtb* biofilm formation.^16^ All compounds in the library contained the ring-fused thiazolo-2-pyridone scaffold with a wide variety of substituents at four positions (R_1_, R_2_, R_3_, and R_4_) with some core scaffold modification in different combinations (Figure 1B; Supporting Information Figures S1–S4). The structure–activity relationship obtained from the small library of ring-fused thiazolo-2-pyridones steered our attention to the C7 position (Figure 1B). We observed that a large C7 substituent was required to maintain the anti-*Mtb* activity, and the most active analogues were equipped with a naphthyl, as in **C10**, or substituted aryl linked by a methylene group. However, we had not exhaustively explored C7 substituents in the initial library. Thus, we hypothesized that additional fine-tuning at this position could yield more potent compounds. We envisioned that substituting a naphthyl group with heteroatom-containing substituents at position C7 could potentially improve the binding affinity by providing hydrogen bond acceptors to the target and also the pharmacokinetic and pharmacodynamic properties to aid future drug development. Additionally, we designed compounds to test the effect of rotatable bonds on activity: a more rigid substituent (i.e., less rotatability) should lose less conformational entropy while binding to its target, but increased rigidity could limit potential positional shifts to optimize interactions with the target. Exploration of these possibilities required a strategy to efficiently synthesize a variety of C7-substituted analogues.

**Scheme 1:**
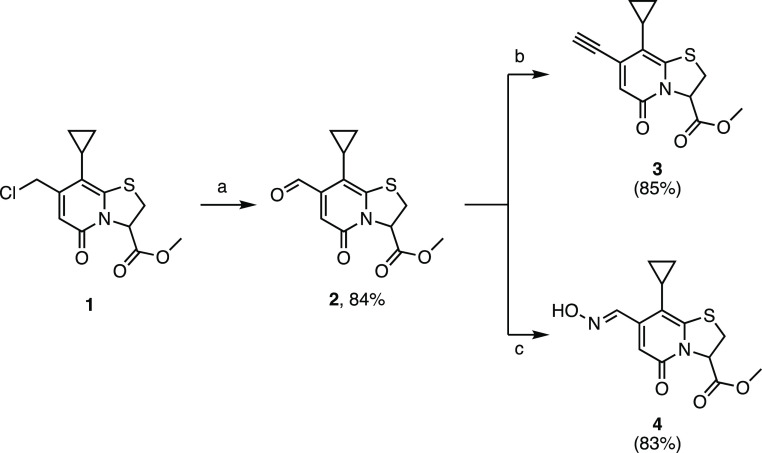
Transformation of Chloromethyl (**1**) to Aldehyde (**2**) on Ring-Fused Thiazolo-2-pyridone and Subsequent Alkyne (**3**) and Oxime (**4**) Intermediate Synthesis Reagents and conditions: (a) NMO, KI, THF, 4 h, reflux. (b) Bestmann–Ohira reagent, K_2_CO_3_, MeOH, 3 h, rt. (c) NH_2_OH·HCl, NaOAc, MeOH, 4 h, rt.

We determined that a C7 chloromethylene-substituted ring-fused thiazolo-2-pyridone (**1**) would be a key intermediate and had previously developed a method to synthesize **1** in gram scale.^17^ This chloromethylene analogue could then serve as an electrophilic partner, enabling us to introduce heteroatom-containing nucleophiles. Furthermore, the C7 chloromethylene intermediate enabled us to synthesize the corresponding C7 formyl (**2**), acetylene (**3**), and oxime (**4**) via known methodologies^18−20^ (Scheme 1).

**Figure 2:**
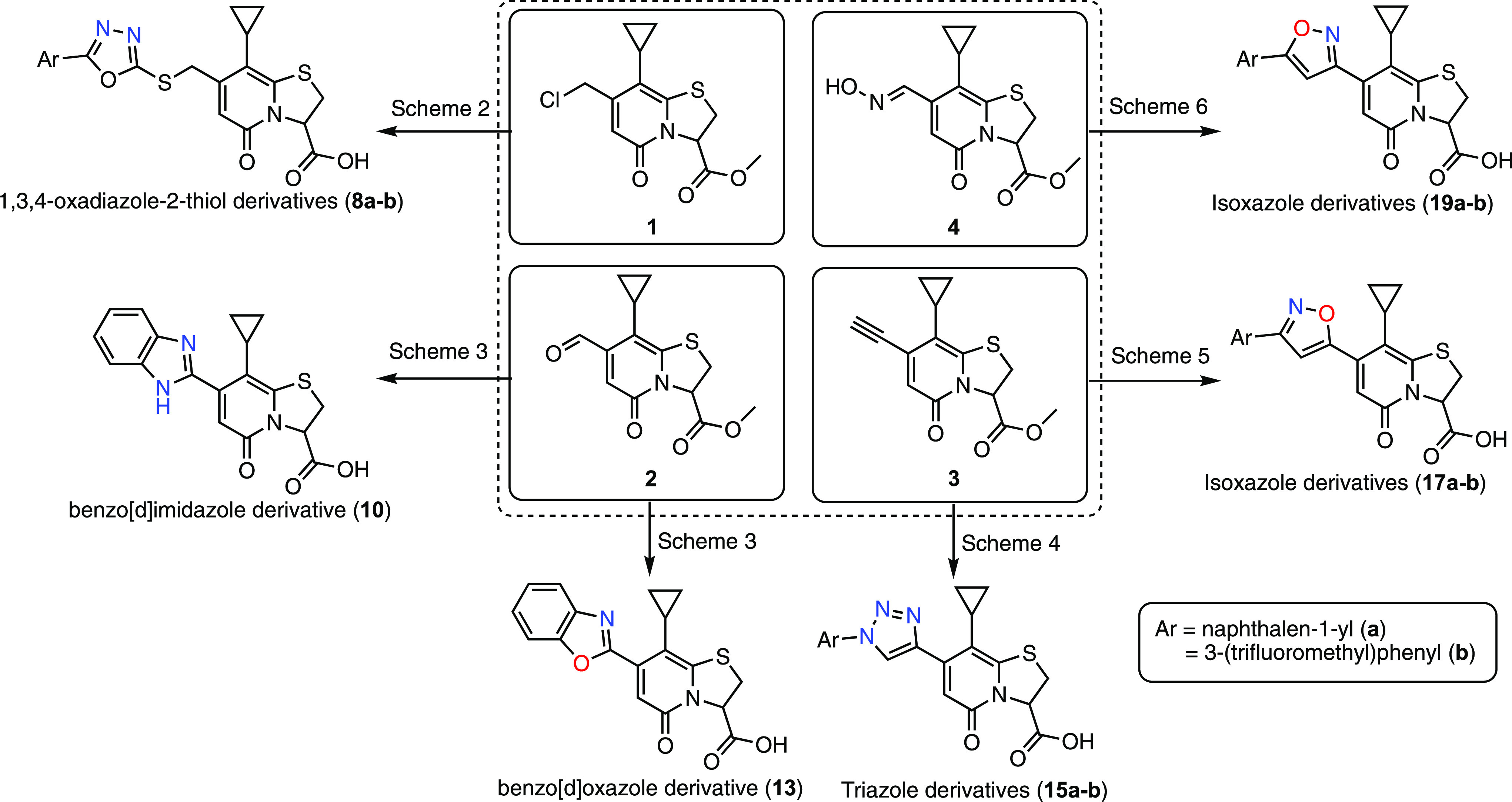
Functional group diversification at the C7 position of ring-fused thiazolo-2-pyridone through accessible common intermediates.

**Scheme 2:**
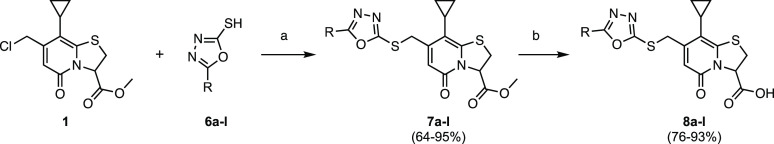
Synthesis of Ring-Fused 2-Pyridone Containing 1,3,4-Oxadiazoles **7a–l** and Subsequent Hydrolysis Products **8a–l**

**Scheme 3:**
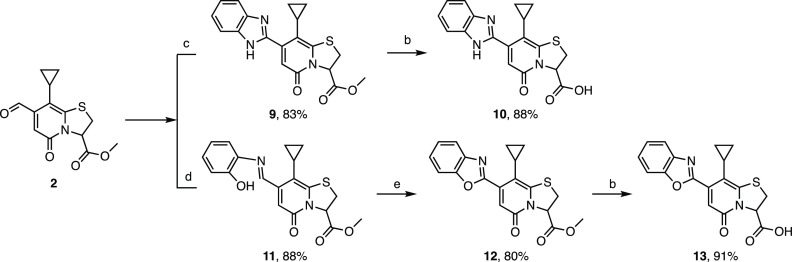
Synthesis of Benzo[*d*]imidazole and Benzo[*d*]oxazole 2-Pyridone

**Scheme 4:**
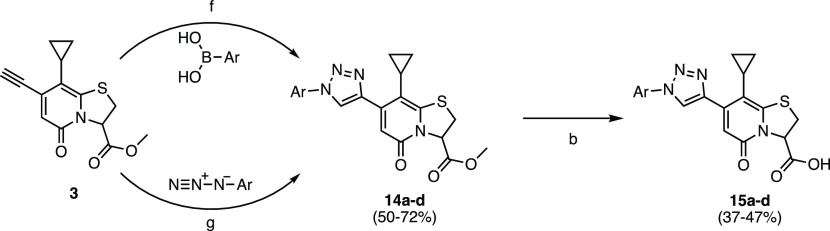
Synthesis of Ring-Fused 2-Pyridone Containing Triazoles **14a–d** from C7 Alkyne Intermediate 3 and Subsequent Hydrolysis Products **15a–d**

**Scheme 5:**
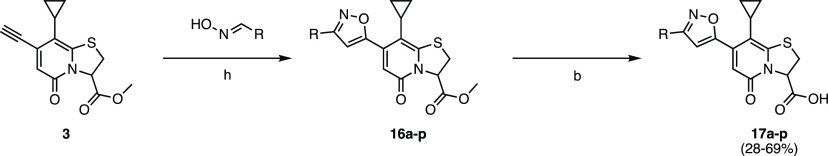
Synthesis of Ring-Fused 2-Pyridone Containing Isoxazoles **16a–p** from Alkyne Intermediate 3 and Subsequent Hydrolysis Products **17a–p**

**Scheme 6:**
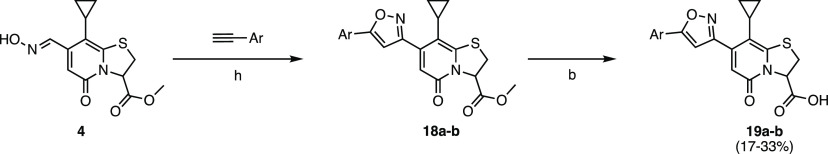
Synthesis of Ring-Fused 2-Pyridone Containing Isoxazoles **18a–b** from Oxime Intermediate 4 and Subsequent Hydrolysis Products **19a–b** Reagents and conditions: (a) TBAB, NaOH, DCM/H_2_O (1:1), rt, 16 h. (b) LiOH (1 M aq), THF, rt, 2 h. (c) *o*-Phenylenediamine, ethanol, 16 h, reflux. (d) 2-Aminophenol, ethanol, 16 h, reflux. (e) PhI(OAc)_2_, CH_3_CN, air, 24 h, rt. (f) NaN_3_, CuSO_4_·5H_2_O, sodium ascorbate, MeOH/H_2_O (1:1), rt, 24 h (for **14a** and **14b**). (g) CuSO_4_·5H_2_O, sodium ascorbate, ^*t*^BuOH/H_2_O (1:1), 40 °C, 24 h (for **14c** and **14d**). (h) CuSO_4_·5H_2_O, Cu, chloramine-T·3H_2_O, ^*t*^BuOH/H_2_O (1:1), rt, 15 h.

These functional groups served as a foundation for the synthesis of five-membered heterocycles with different heteroatom connectivity (Figure 2). From there, we initiated the synthesis of the first branch of our diversified C7 substituent library with 1,3,4-oxadiazole-2-thiol derivatives (**8a** and **8b**). These were accessible via the chloromethylene intermediate (**1**) and 5-substituted 1,3,4-oxadiazole 2-thiols via direct nucleophilic substitution (Scheme 2). Similarly, with the other C7-functionalized intermediates in hand, we prepared two rigid analogues (one rotatable bond): a benzo[*d*]imidazole (**10**) and a benzo[*d*]oxazole (**13**). These analogues were synthesized via condensation between the aldehyde (**2**) and the corresponding *o*-phenylenediamine and 2-aminophenol, respectively (Scheme 3). Triazoles **15a** and **15b** were synthesized using “click” chemistry between azides and the acetylene-substituted intermediate (**3**) (Scheme 4). Finally, we synthesized two 3-substituted isoxazole analogues **17a** and **17b** from the acetylene (**3**) (Scheme 5), and two regioisomeric 5-substituted isoxazole analogues **19a** and **19b** from the oxime (**4**) (Scheme 6). Based on the initial screening results (described below) of our diversified primary library of compounds, we focused on the substitution pattern and expanded the library further on the most accessible oxadiazoles (Scheme 2, Figure 2) and the complementary isoxazoles (Scheme 5, Figure 2).

### Synthesis of **C7** Chloromethyl-Ring-Fused Thiazolo-2-pyridone and Its Modification on C7

The chloromethyl group on compound **1** was oxidized to the C7 formyl-ring-fused thiazolo-2-pyridone **2** in an 84% yield in the presence of *N*-methylmorpholine-*N*-oxide (NMO) and a catalytic amount of KI in dry THF under refluxing conditions (Scheme 1). Next, the key intermediate **2** was transformed into two different functional groups at the C7 position of the ring-fused thiazolo-2-pyridone. In the first transformation, a terminal alkyne group was synthesized in one step by the Seyferth–Gilbert homologation using the Bestmann–Ohira reagent to obtain the C7 alkyne-ring-fused thiazolo-2-pyridone (**3**) in an 85% yield (Scheme 1). The Bestmann–Ohira reagent was prepared in the lab by following the standard method of synthesis as described in Scheme S1 (Supporting Information). In another transformation, the C7 oxime-ring-fused thiazolo-2-pyridone **4** was synthesized by condensation of the C7 formyl group and NH_2_OH·HCl in the presence of NaOAc in MeOH (Scheme 1).

### Synthesis of 5-Substituted 1,3,4-Oxadiazole-2-thiols

The synthetic route to obtain oxadiazole intermediates (**6a–l**) is described in Scheme S2 (Supporting Information). The acyl hydrazides were synthesized from the corresponding carboxylic acids through the pre-formation of esters followed by a nucleophilic substitution by the hydrazine hydrate. The syntheses of 5-substituted-1,3,4-oxadiazole-2-thiols (**6a–l**) were carried out with acyl hydrazides and carbon disulfide under refluxing conditions in ethanol in the presence of a base. The products thus obtained were used for the subsequent reactions.

### Synthesis of Ring-Fused Thiazolo-2-pyridone-5-benzylsulfanyl-1,3,4-oxadiazole Derivatives

The synthesis of ring-fused thiazolo-2-pyridone-oxadiazole derivatives over two steps is shown in Scheme 2. The nucleophilic substitution of C7 chloromethyl-ring-fused thiazolo-2-pyridone (**1**) with oxadiazoles (**6a–l**) was carried out using the phase transfer catalyst tetrabutylammonium bromide (TBAB). The resulting ring-fused thiazolo-2-pyridone-oxadiazole methyl ester derivatives (**7a–l**) were hydrolyzed to obtain the final products (**8a–l**) in 76–93% yields.

### Synthesis of Benzo[*d*]imidazole and Benzo[*d*]oxazole Ring-Fused Thiazolo-2-pyridone

The condensation reaction between *o*-phenylenediamine and C7 formyl-ring-fused thiazolo-2-pyridone (**2**) was carried out in absolute EtOH under refluxing conditions to yield 83% of the benzo[*d*]imidazole fused ring-fused thiazolo-2-pyridone (**9**) (Scheme 3). The final product **10** was obtained in an 88% yield from the hydrolysis of the intermediate **9** following the general procedure of hydrolysis. However, by following the same condensation procedure but with 2-aminophenol and C7 formyl-ring-fused thiazolo-2-pyridone (**2**), only the ring-opened product **11** was formed in an 88% yield. Thus, to obtain the benzo[*d*]oxazole fused ring-fused thiazolo-2-pyridone (**12**), intermediate **11** was oxidized in the presence of PhI(OAc)_2_ in acetonitrile (Scheme 3). The final compound **13** was obtained in a 91% yield after hydrolysis.

### Synthesis of 1,2,3-Triazole Ring-fused Thiazolo-2-pyridone

The triazole heterocycles were synthesized using intermediate **3** and substituted aromatic azides. The synthesis of **14a-b** was carried out by in situ transformation of 1 or 2-naphthyl boronic acid to azide followed by the Cu-catalyzed click coupling with **3** in one-pot (Scheme 4). Compounds **14c-d** were obtained from the corresponding aromatic azides synthesized from the corresponding amines (Supporting Information Scheme S3) via coupling with the terminal alkyne intermediate **3** via Cu-catalyzed azide/alkyne click chemistry. The final triazole products were obtained after hydrolysis in moderate yields (37–47%).

### Synthesis of Isoxazole-Substituted Ring-Fused Thiazolo-2-pyridone

Compounds **16a–p** were synthesized from **3**, and the corresponding substituted aromatic/aliphatic oximes are shown in Scheme 5. The oximes **Ia–p** were synthesized from commercially available aldehydes in the presence of NH_2_OH·HCl and NaOAc as a base in MeOH (Supporting Information Scheme S4). The isoxazole compounds **16a–p** were obtained by the activation of oxime intermediates using chloramine-T·3H_2_O in a ^*t*^BuOH/H_2_O (1:1) mixture followed by copper-mediated [3 + 2] cycloaddition of the terminal alkyne intermediate **3** (Scheme 5). In the next step, compounds **16a–p** were hydrolyzed to the final compounds **17a–p** (Scheme 5) in 28–69% yields.

### Synthesis of Regioisomer Isoxazole-Substituted Ring-Fused Thiazolo-2-pyridone

Compounds **18a-b** were synthesized via intermediate **4** and its condensation with terminal alkynes in the presence of chloramine-T·3H_2_O in a mixture of ^*t*^BuOH/H_2_O (1:1) (Scheme 6). The final products **19a-b** were obtained in 17–33% yields after hydrolysis.

## Biological Evaluation

### Structure–Activity Relationship Analysis of the Derivatives of the Key Intermediate

The goal of the work presented here was to identify a more potent anti-*Mtb* ring-fused thiazolo-2-pyridone than **C10** while maintaining its INH-potentiating behaviors that we described in our previous report.^16^ We used the microplate Alamar Blue assay (MABA)^21^ to analyze the efficacy of our second-generation ring-fused thiazolo-2-pyridone library with the goal of identifying compounds that are more potent inhibitors of *Mtb* respiration and growth than **C10**. The MABA uses the dye resazurin, which is blue in its oxidized form and gets reduced by cellular metabolism to resorufin, a pink, fluorescent product. Thus, the MABA serves as a measure of *Mtb* metabolism, respiration, and growth, and is commonly used to evaluate the efficacy of anti-mycobacterial compounds. To improve compound solubility in the assay medium, we tested all compounds in our second-generation C7 library as imidazole salts (IMD) and included **C10** as an IMD salt in every assay as the comparator. We treated wild-type (WT) *Mtb* Erdman with a range of concentrations for each compound and calculated the concentration that inhibits *Mtb* by 50% (IC_50_) for each compound (Tables 1–4).

**Table 1:** MABA IC_50_ Values for C10-IMD and Key Derivatives from C7 Functional Group Diversification (Figure 2)^a^

compound	mean IC_50_ ± SD (μM)	*n*	*p* value
**C10**	14.44 ± 1.32	3	N/A
**8a**	12.62 ± 2.16	3	0.7656
**8b**	NS	N/A	N/A
**10**	>25	3	<0.0001
**13**	>25	3	<0.0001
**15a**	>25	2	<0.0001
**15b**	>25	2	<0.0001
**17a**	1.86 ± 1.13	3	0.0428
**17b**	3.04 ± 0.58	3	0.0658
**19a**	3.90 ± 1.43	3	0.0881
**19b**	5.09 ± 1.30	3	0.1295

a NS = not soluble. *p*-Values for comparison to C10 using ordinary one-way ANOVA with Fisher’s LSD Test performed in Prism.

**Table 2:**
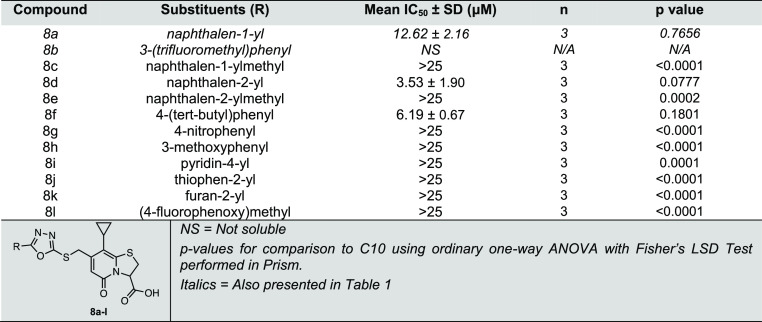
MABA IC_50_ Values for 1,3,4-Oxadiazole Hydrolysis Products, with the General Structure of **8a–l** (Scheme 2)

**Table 3:**
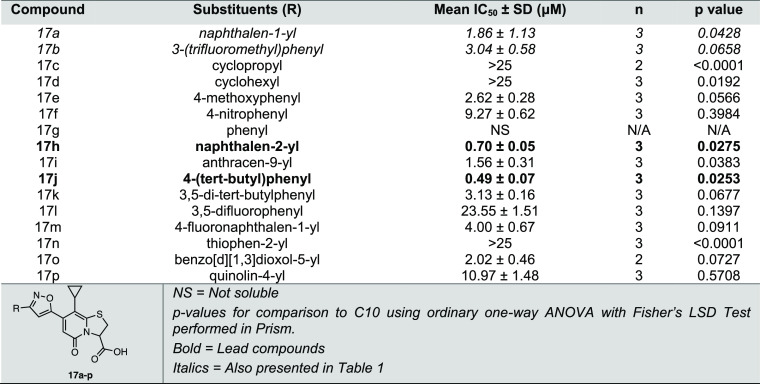
MABA IC_50_ Values for Isoxazole Hydrolysis Products, with the General Structure of **17a–p** (Scheme 5)

**Table 4:**
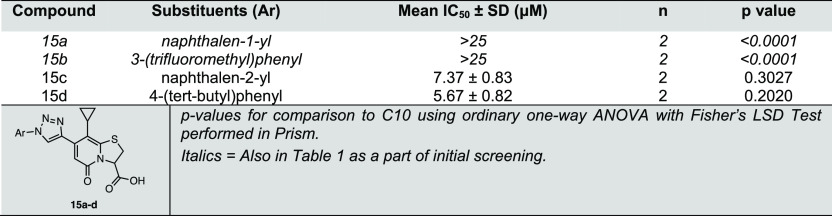
MABA IC_50_ Values for Ring-Fused Thiazolo-2-Pyridone Containing Triazoles Hydrolysis Products, with the General Structure of **15a–d** (Scheme 4)

An α-naphthyl group is the main C7 substituent on **C10**;^16^ therefore, we initiated our first round of MABA screens with a more flexible C7 oxadiazole containing an α-naphthyl group (**8a**) (Table 1). Analogue **8a** and **C10** had comparable MABA IC_50_ values (12.62 and 14.44 μM, respectively), indicating that increased C7 flexibility and oxadiazole scaffold do not significantly alter activity. In contrast, the other oxadiazole analogue containing 3-(trifluoromethyl)phenyl (**8b**) faced solubility limitations in the MABA, and thus, an IC_50_ could not be determined (Table 1). However, the more rigid compounds benzo[*d*]imidazole (**10**) and benzo[*d*]oxazole (**13**) exhibited significantly less activity (**10**: IC_50_ > 25 μM and **13**: IC_50_ > 25 μM) (Table 1). The corresponding α-naphthyl and 3-(trifluoromethyl)phenyl-substituted 1,2,3-triazole analogues demonstrated a similarly significant drop in inhibitory activity (**15a**: IC_50_ > 25 μM and **15b**: IC_50_ > 25 μM), while the corresponding isoxazole analogues (**17a**: IC_50_ = 1.86 μM and **17b**: IC_50_ = 3.04 μM) exhibited improved inhibitory activity compared to **C10** (Table 1). The regioisomeric isoxazole analogues (**19a**: IC_50_ = 3.90 μM and **19b**: IC_50_ = 5.09 μM) did not significantly alter the inhibitory activity compared to **17a** and **17b** (Table 1). Additionally, **19a** and **19b**, synthesized from oxime intermediate (**4**) (Figure 2; Scheme 6), produced poor yields likely due to the low solubility of intermediate **4** under the reaction conditions.

### Anti-*Mtb* Activity of Oxadiazole, Isoxazole, and Triazole Derivatives

Based on these initial results, we focused on fine-tuning the substitution pattern to further expand the library by making substitutions to the most accessible oxadiazoles (Figure 2; Scheme 2). We introduced a range of substitutions including aryls, naphthyls, and heteroaryls and synthesized and evaluated 10 additional oxadiazoles (Scheme 2, Table 2). The 1,3,4-oxadiazoles were straightforward to synthesize and highly scalable; thus, we introduced a few 5-substituted 1,3,4-oxadiazole 2-thiols.

A more flexible naphthalen-1-ylmethyl (**8c**) substitution showed no improved activity (IC_50_ > 25 μM, Table 2). A β-naphthyl substitution (**8d**) improved *Mtb* inhibition (IC_50_ = 3.5 μM, Table 2) compared to **C10**. The introduction of a methylene linker between the β-naphthyl and oxadiazole substituents in **8e** significantly reduced the inhibitory activity (IC_50_ > 25 μM, Table 2). These results indicate that a methylene extension significantly reduced activity, while a naphthalene directly attached to oxadiazole trended toward improving activity. The bulky 4-(*tert*-butyl)phenyl (**8f**) retained activity (IC_50_ = 6.2 μM, Table 2), and its IC_50_ improved over that of **C10**. The addition of either an electron-withdrawing [4-nitrophenyl (**8g**)] or an electron-donating substituent [3-methoxyphenyl (**8h**)] significantly decreased *Mtb* respiration inhibition (**8g** and **8h** IC_50_ > 25 μM, Table 2). Similarly, heterocyclic substituents, such as 4-pyridinyl (**8i**), 2-thiophenyl (**8j**), and 2-furanyl (**8k**), and a (4-fluorophenoxy)methyl-substituted oxadiazole (**8l**) had poor activity against *Mtb* (IC_50_ > 25 μM for each **8i**–**8l**, Table 2). This expanded library of oxadiazoles provided important insight into the substituents that exhibited improved *Mtb* inhibition activity and assisted in designing other classes of compounds.

Among the different classes of compounds, the initial screening results showed a great improvement in anti-*Mtb* activity with isoxazole analogues **17a** and **17b**. Also, with the understanding of SAR from oxadiazoles, we were eager to expand the isoxazole library. Based on the superior activity and synthetic accessibility, we chose to expand 3-substituted isoxazole analogues over 5-substituted isoxazole analogues. In order to access the isoxazole library, we used our formyl intermediate (**2**) (Scheme 1) to perform Seyferth–Gilbert homologation to synthesize the terminal alkyne (**3**) (Scheme 1), and then coupled substituted oxime intermediates to access isoxazole via [3 + 2] cycloaddition (Scheme 5). We tested several conditions for isoxazole synthesis and found that the conditions reported in Scheme 5 were most effective to work with our ring-fused thiazolo-2-pyridone scaffold; this approach also provided us with regio-control over isoxazole formation. Following the improvement in the activity of isoxazole derivatives **17a** and **17b** (Figure 2, Scheme 5, and Table 1), we screened an extended panel of analogues with substituents at the C3 isoxazole position. First, we introduced a shorter cyclopropyl group to synthesize **17c** and a larger cyclohexyl group to make **17d**, both of which significantly lost activity relative to **C10** (**17c** and **17d** IC_50_ > 25 μM, Table 3), indicating that introduction of an aliphatic substitution is unfavorable to *Mtb* respiration inhibition. Addition of an electron-donating *para-*methoxyphenyl group in **17e** (IC_50_ = 2.62 μM, Table 3) significantly improved anti-*Mtb* activity over **C10**, to a level comparable to that of **17a**. However, substitution with an electron-withdrawing *para*-nitrophenyl in **17f** (IC_50_ = 9.27 μM) retained similar activity to **C10** (Table 3). Inconsistent data were observed for the unsubstituted phenyl (**17g**) due to solubility issues (Table 3). Collectively, these trends in SAR indicate that an electronically neutral aromatic substitution or an electron-donating substitution at the C3 position of the isoxazole is more favorable than an electron-withdrawing or an aliphatic substitution.

In further exploration of aromatic substitutions, a β-naphthyl substitution (**17h**) (IC_50_ = 0.70 μM, Table 3) led to a more than 20-fold enhanced potency compared to **C10**. A subtle change in the position of ring attachment from α- to β-naphthyl had a significant impact on activity. However, a larger 9-anthracene substitution in **17i** (IC_50_ = 1.56 μM, Table 3) was threefold less potent than the β-naphthyl analogue **17h** but had similar activity to the α-naphthyl analogue (**17a**) and better activity than **C10**. Next, we systematically examined the effect of substitutions on the phenyl group attached to the isoxazole spacer. The addition of 4-*tert*-butyl phenyl (**17j**) (IC_50_ = 0.49 μM, Table 3) improved activity more than 29-fold over **C10**. The 3,5-di-*tert*-butylphenyl (**17k**) (IC_50_ = 3.13 μM, Table 3) substituted isoxazole had improved potency over **C10**, and the 3,5-difluorophenyl substitution (**17l**) (IC_50_ = 23.55 μM, Table 3) decreased activity when compared to **C10**. Next, we shifted from phenyl group substitutions to a 4-fluoro substitution on α-naphthyl (**17m**) (IC_50_ = 4.0 μM, Table 3), which was better than **C10** but inferior compared to **17a**, suggesting that electron-withdrawing groups such as the fluorine functionality do not improve activity. Subsequent SAR investigation was directed toward the introduction of a heteroaromatic substituent. Compound **17n** with a 2-thiophenyl (IC_50_ > 25 μM, Table 3) had significantly less activity than **C10**. However, **17o** with a benzo[*d*][1,3]dioxol-5-yl group (IC_50_ = 2.02 μM, Table 3) had significantly improved activity and **17p** with a 4-quinolone substitution (IC_50_ = 10.97 μM, Table 3) retained activity close to that of **C10**. Among the substituted isoxazole analogues screened, we observed that many of the substituents showed significantly improved activity against *Mtb*. In particular, compounds **17h** and **17j** substituted with β-naphthyl and 4-(*tert*-butyl)phenyl showed 20-fold and 29-fold increased activity, respectively, when compared to **C10**.

**Figure 3:**
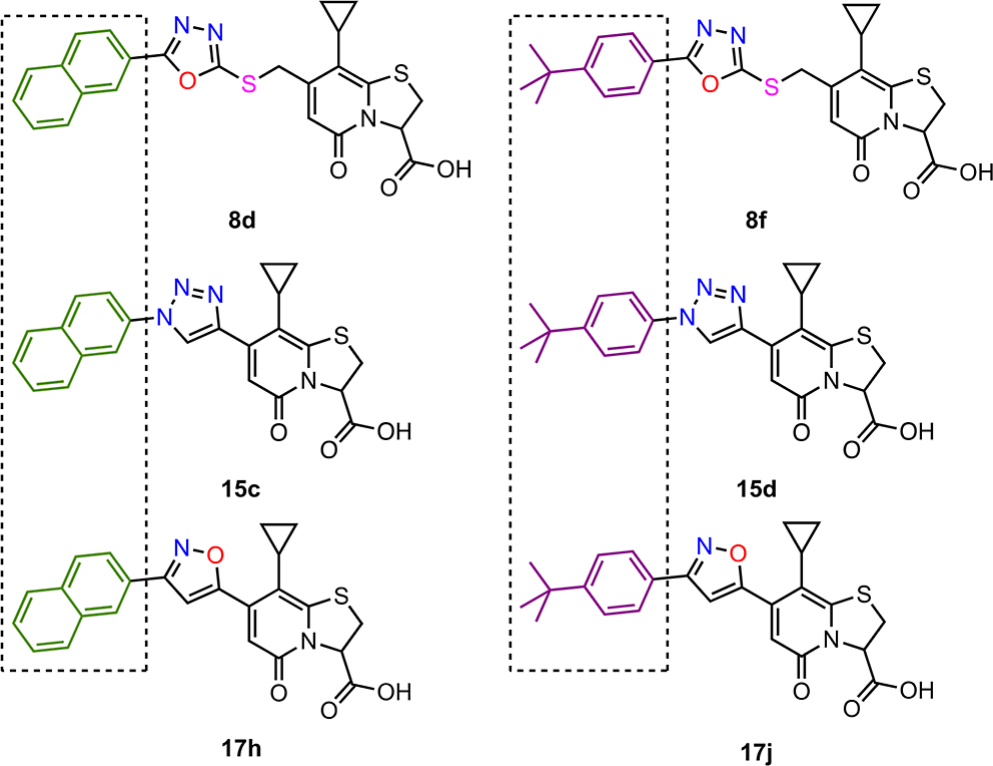
Structures of the two most active analogues from oxadiazole, triazole, and isoxazole class of compounds. The two new best substituents identified are naphthalen-2-yl (green) and 4-(*tert*-butyl)phenyl(purple).

Even though we did not observe any improved activity of the initial pair of 1,2,3-triazoles tested (Table 1), we synthesized and tested two additional triazole analogues because of the heteroaromatic resemblance of a triazole with isoxazole (Figure 2, Scheme 4). To directly compare the activity of triazole and isoxazole spacers, we synthesized triazole analogues with substituents similar to our most potent isoxazole analogues, **17h** and **17j** (Figure 3). The β-naphthyl analogue (**15c**) (IC_50_ = 7.37 μM, Figure 3 and Table 4) had activity comparable to **C10** but significantly lower than that of its parallel isoxazole **17h** (Figure 3, Table 3). Similarly, the activity of 4-*tert*-butyl triazole (**15d**) (IC_50_ = 5.67 μM, Figure 3, Table 4) was significantly lower than that of its corresponding isoxazole analogue **17j** (Figure 3, Table 3). Thus, the observations corroborate the initial comparison of a triazole with isoxazole analogues, demonstrating that the isoxazole heterocyclic spacer is favorable over triazole at the C7 position of peptidomimetic ring-fused thiazolo-2-pyridone for anti-*Mtb* activity.

### Permeability and CYP Inhibition of the Most Active Compounds from Three Different Classes

The most active analogues from the oxadiazole, triazole, and isoxazole class of compounds contained a naphthalen-2-yl or a 4-(*tert*-butyl)phenyl substituent at the terminal of the heterocyclic spacers (Figure 3). Among the heterocyclic spacers, isoxazole analogues (**17h** and **17j**) showed favorable anti-*Mtb* activity over the oxadiazole (**8d** and **8f**) and the triazole analogues (**15c** and **15d**) when compared with similar terminal substituents (Figure 3; Tables 2–4). In order to start to gain a comprehensive understanding of the compounds and anticipate their future development with a specific focus on bioavailability, we examined the membrane permeability of selected compounds **8f**, **15d**, and **17j**. Investigating membrane permeability is a critical step in the preliminary assessment of potential drug candidates aimed at oral administration. The ability of a compound to diffuse across the cell membrane significantly impacts its absorption within the gastrointestinal tract, distribution throughout the body, metabolic processes, and excretion patterns. By conducting a human cell monolayer study and measuring the apparent permeability (Papp), valuable insights into these crucial properties can be obtained. We tested all three selected compounds for their permeability in Caco-2 cells. The permeability of the tested compounds showed apical to basolateral Papp coefficient in the range of 0.88–3.3 cm/s and an efflux ratio in the span of 1.5–7.7 (Table S1). The Papp coefficient values indicate a low to moderate permeability of selected compounds with a moderate to high efflux ratio.

The therapeutic regimen for TB typically involves the simultaneous administration of multiple drugs, making drug–drug interactions a critical consideration during drug metabolism. In order to expand our understanding of the selected compounds, we sought to also investigate possible CYP inhibition that then could impact future drug–drug interactions. The cytochrome P450 (CYP450, CYP) inhibition assay aims at investigating the drug–drug interaction potential of new molecular entities. The CYP450 enzyme family is widely recognized as the primary group of enzymes involved in drug metabolism. Consequently, it is crucial to evaluate CYP450 inhibition in early discovery stages for potential drug candidates, as this helps to prevent later-phase failures caused by toxicity concerns. Here, we tested the selected compounds for the inhibition of two representative enzymes from the cytochrome P450 (CYP) family, CYP3A4 and CYP2C9. All three representative compounds showed very low CYP3A4 inhibition with approximate IC_50_ above 100 μM (Table S2), approximately 16-fold or higher than their respective IC_50_ in MABA, indicating a low risk of drug–drug interaction during concurrent drug delivery. However, all three tested compounds inhibited CYP2C9 at significantly lower concentrations, with approximate IC_50_ values between 10 and 100 μM for **8f** and 1–10 μM for **15d**, and a value equal to or lower than 1 μM for **17j** (Table S3), indicating a possible risk of higher-order drug–drug interactions. These parameters will be pursued in more depth in follow-up studies, particularly examining the carboxylic acid functionality, which others have shown can affect permeability and efflux.^22,23^ Since the differences in these parameters between the analogues in this current study were quite small and since we are at an early stage of anti-*Mtb* drug development, we prioritized following up in more detail on anti-*Mtb* activity and cytotoxicity in different cell lines with the two most potent active compounds **17h** and **17j** (*in vitro*).

### Lead Compounds Are Nontoxic to Mammalian Cells at Concentrations That Inhibit *Mtb*

**Figure 4:**
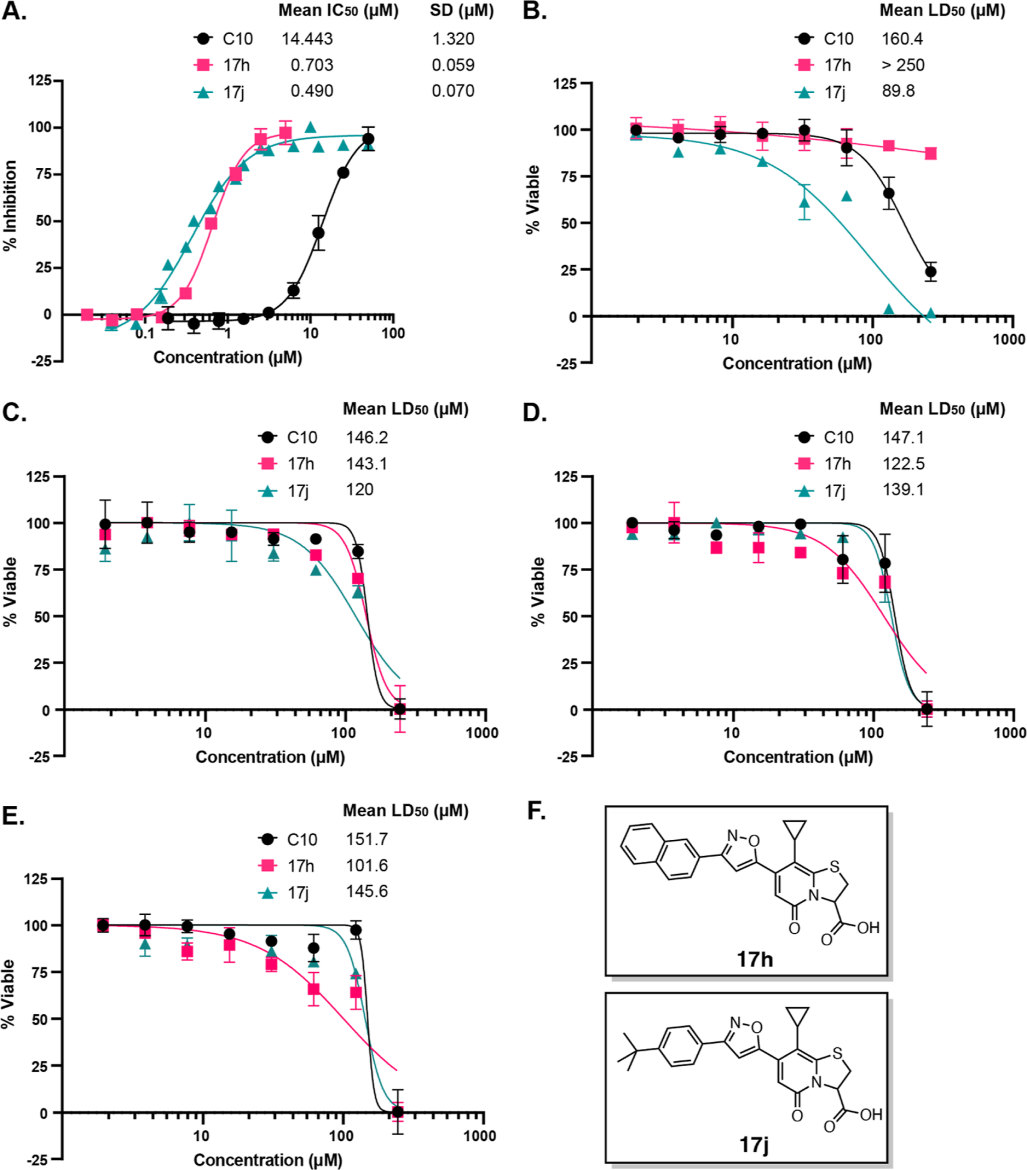
**17h** and **17j** are potent, nontoxic inhibitors of *Mtb*. (A) *Mtb* was treated with increasing concentrations of **C10**, **17h**, or **17j** and monitored in the MABA. Using GraphPad Prism, the IC_50_ ± SD was calculated from a nonlinear regression for each compound. (B) Cytotoxicity in Calu-3 cells was quantified by treating cells with increasing concentrations of each compound and measuring cellular ATP production as luminescence with the CellTiter-Glo kit after 72 h of compound treatment. LD_50_ ± SD was calculated with GraphPad Prism. All three compounds were prepared as IMD salts and dissolved in DMSO. IMD and DMSO were included in the untreated controls. Data points represent mean ± SD. Cytotoxicity in (C) BEAS-2B, (D) IMR-90, and (E) HEK 293 cell lines was quantified by treating cells with increasing concentrations of **C10**, **17h**, or **17j** and performing an MTT assay. LD_50_ ± SD was calculated with GraphPad Prism. *n* = 3 for each compound in each panel. (F) Structures of **17h** and **17j**.

The most potent compounds from the MABA analysis contain a β-naphthalene or a 4-(*tert*-butyl)phenyl substituent on the isoxazoles of **17h** and **17j**, respectively. They represent a suitable substituent, where both bulkier and smaller substitutions had inferior activity, and compounds containing more strongly electron-donating or electron-withdrawing heteroaromatics performed more poorly in the MABA. **17h** and **17j** were 20-fold and 29-fold more potent than **C10** in the MABA, respectively (Figure 4A). Before proceeding with these two lead compounds to test their activity in additional *Mtb* assays, we wished to rule out the possibility of toxicity to eukaryotic cells. Using Promega’s CellTiter-Glo assay to assess cellular ATP levels as a read-out of viability, we tested a range of concentrations of **C10**, **17h**, and **17j** on the viability of Calu-3 cells, a human lung epithelial cell line, over a 72 h of incubation. We determined the concentration of the compound that resulted in a 50% decrease in cellular ATP (LD_50_) (Figure 4B).

To estimate a therapeutic index for each compound, we calculated the ratio of the LD_50_ in Calu-3 cells to the MABA IC_50_ in *Mtb* for each compound, where the larger the index, the safer the compound is in eukaryotic cells relative to its effective dose. The therapeutic indices for **C10**, **17h**, and **17j** were 11.1, >355, and 182.5, respectively (Table 5), indicating that both **17h** and **17j** (Figure 4F) have larger therapeutic indexes in Calu-3 cells than **C10**. Furthermore, we examined the toxicity of these three compounds against human non-tumorigenic lung epithelial cells (BEAS-2B), normal lung fibroblasts (IMR-90), and human embryonic kidney (HEK 293) cell lines over a 48 h incubation period. Using the 3-(4,5-dimethylthiazol-2-yl)-2,5-diphenyltetrazolium bromide (MTT) assay, we identified the compound concentration that caused a 50% reduction in cell viability (Figure 4C–E). The ratio of the LD_50_ in each of these three cell lines to the MABA IC_50_ in *Mtb* for each compound was used to determine a therapeutic index. **C10**, **17h**, and **17j** had comparable therapeutic indexes in BEAS-2B, IMR-90, and HEK 293 cell lines as observed in Calu-3 cells (Figure 4C–E, Table 5). Thus, in addition to **17h** and **17j** having greater potency in *Mtb* than **C10**, they also have an improved safety profile in relevant human cell lines.

**Table 5:** Compound Therapeutic Index as the Ratio of Toxicity (LD_50_) in Each of Four Mammalian Cell Lines to Anti-*Mtb* MABA Activity (IC_50_)

compound	Calu-3^a^	BEAS-2B^b^	IMR-90^b^	HEK 293^b^
**C10**	11.1	10.1	10.1	10.5
**17h**	>355	203.5	167.8	144.5
**17j**	182.5	243.9	282.7	295.9

a Therapeutic index calculated based on LD_50_ of 72 h compound treatment of cells followed by CTG assay.

b Therapeutic index calculated based on LD_50_ of 48 h compound treatment of cells followed by MTT assay.

### Lead Compounds Inhibit *Mtb* Biofilm Formation

**Figure 5:**
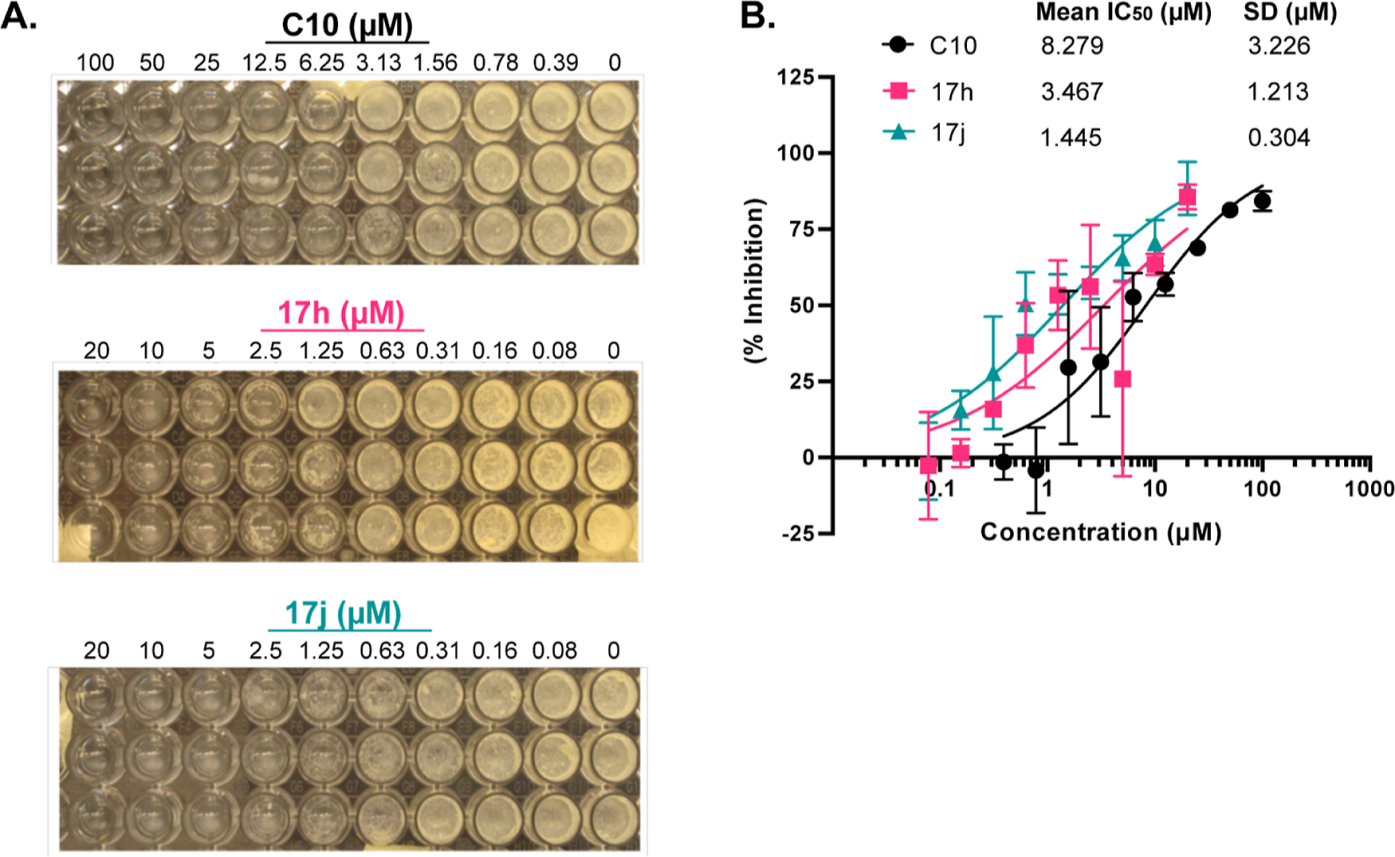
**17h** and **17j** inhibit *Mtb* biofilm formation. *Mtb* was incubated in the presence of increasing doses of each compound for 3 weeks under hypoxic conditions, followed by 3 weeks of incubation under aerobic conditions. After 6 weeks total of incubation, (A) pictures of biofilm pellicles for three replicates of each compound are shown and (B) OD_600_ through the pellicle biofilm at the air/liquid interface in each well was measured. Percent inhibition was calculated compared to DMSO/IMD control wells and plotted by compound concentration. IC_50_ (relative to no treatment) was calculated for each compound using GraphPad Prism. All three compounds were prepared as IMD salts and dissolved in DMSO. IMD and DMSO were included in the untreated controls. *n* = 3 for each compound. Data points represent mean ± SD.

**C10** was originally discovered as an inhibitor of *Mtb* biofilm formation, with an IC_50_ for biofilm inhibition that is similar to the IC_50_ in the MABA.^16^ To test whether our two new lead compounds maintained this activity, we performed biofilm inhibition assays by treating *Mtb* with a range of compound dilutions over the course of 3 weeks under hypoxic conditions followed by 3 weeks of incubation under aerobic conditions (Figure 5A). We assessed the inhibition of biofilm formation by measuring OD_600_ through the pellicle at the air/liquid interface and calculating an IC_50_ (Figure 5B). Visually, both **17h** and **17j** inhibited biofilm pellicle formation at concentrations lower than **C10** (Figure 5A). When we quantified biofilm inhibition by OD_600_, we found that both compounds were more potent than **C10**, with **17j** being more potent at inhibiting biofilm pellicle formation than **17h** (Figure 5B). We had previously shown that the inhibition of biofilm pellicle formation by **C10** occurs at concentrations that are not sufficient to inhibit *Mtb* growth, demonstrating that **C10** inhibits a physiological process required for biofilm formation.^16^ Since **17h** and **17j** were more potent than **C10**, we confirmed that the effects on OD_600_ in the biofilm cultures were specific for the inhibition of biofilm formation and not reflective of changes in bacterial survival. We performed similar dose range experiments in the presence of 0.05% tyloxapol, a detergent that will prevent pellicle biofilm formation by *Mtb*. At their respective IC_50_ concentrations for biofilm inhibition, neither **17h** nor **17j** inhibited the OD_600_ of *Mtb* cultures in the presence of tyloxapol (Supporting Information Figure S5), supporting that at concentrations that are subinhibitory for growth inhibition, **17h** and **17j** can inhibit biofilm formation. These data demonstrate that the improved activity we observed for **17j** in the MABA correlated with increased potency for biofilm inhibition.

### **17h** and **17j** More Potently Resensitize an *Mtb katG* Mutant to Inhibition by INH than C10

One of **C10**’s most exciting anti-*Mtb* properties is its resensitization of INH-resistant *Mtb* to INH. To assess whether our new, more potent compounds maintained this key phenotype, we treated an INH-resistant mutant that has a frameshift mutation in *katG* at amino acid six (*katG*^FS^)^16^ with INH or 1 μM of **C10**, **17h**, or **17j** alone or in combination with INH. The MABA IC_50_ values for **C10**, **17h**, and **17j** are 14.4, 0.703, and 0.492 μM, respectively. Therefore, the 1 μM ring-fused thiazolo-2-pyridone dose used in this study is lower than the **C10** IC_50_ value, higher than the **17h** and **17j** IC_50_ values, and lower than the concentration of **C10** previously used to resensitize *Mtb katG*^FS^ to INH, which was 25 μM.^16^ However, by using 1 μM here, we are able to directly compare **C10**, **17h**, and **17j** efficacy at a single concentration in promoting INH activity in an *Mtb katG* mutant. For the INH-treated samples, we used a concentration of INH (0.25 μg/mL) that alone completely inhibits WT *Mtb* growth, but only partially inhibits growth of *Mtb katG*^FS^.^16^

**Figure 6:**
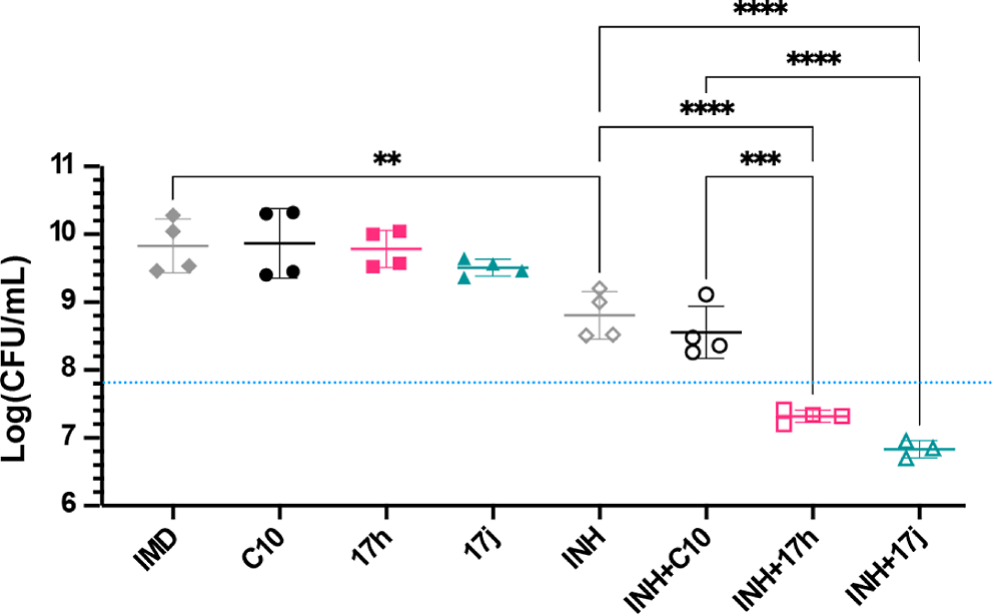
**17h** and **17j** potentiate killing of an INH-resistant *Mtb katG* mutant by INH. *Mtb katG*^*FS*^ was grown to log-phase in Sauton’s media, diluted to 5 × 10^7^ CFU/mL, and treated with 1 μM **C10**, **17h**, or **17j** ± 0.25 μg/mL INH. **C10**, **17h**, and **17j** were prepared as IMD salts and dissolved in DMSO. IMD and DMSO were included in the untreated controls, where IMD in the graph designates the untreated control. Two experiments were performed, each with duplicate samples per condition. Graphed is the mean CFU/mL ± SD after 13 days of treatment for the combined two experiments. The dashed blue line denotes the inoculum CFU/mL. Statistical comparisons were performed in GraphPad Prism with an ordinary one-way ANOVA with Tukey’s correction; only significant differences are shown. **p < 0.01; ***p < 0.001; and ****p < 0.0001.

We monitored *Mtb katG*^FS^ survival by enumerating colony-forming units (CFU) after 13 days of treatment (Figure 6). Treatment with 1 μM **C10**, **17h**, or **17j** alone resulted in no difference in CFU (Figure 6) compared with the control. Treatment with INH alone significantly decreased the number of surviving bacteria after 13 days of treatment by an order of magnitude (8.09 × 10^8^ CFU/mL compared to 9.07 × 10^9^ CFU/mL in control cultures) (Figure 6). Next, we compared the effect of INH alone to its combination treatment with **C10**, **17h**, or **17j**. We found that INH plus 1 μM **C10** did not inhibit the survival of *Mtb katG*^FS^ beyond the effect of INH alone (Figure 6). However, the combination treatment of INH + **17h** or INH + **17j** resulted in significantly less surviving *Mtb katG*^FS^ when compared to INH alone or INH + **C10**, where the CFU following 13 days of treatment with either INH + **17h** or INH + **17j** were below the input dose (Figure 6), demonstrating that these combinations were bactericidal over a 13-day treatment period. Together, these data show that both second-generation lead compounds reproduced the original **C10** phenotype^16^ of enhancing *Mtb katG*^FS^ sensitivity to INH at concentrations that do not elicit a survival defect in the absence of INH and at a lower effective concentration than **C10** (Figure 6).

### Lead Ring-Fused Thiazolo-2-pyridones Inhibit *Mtb* Intracellular Replication in Macrophages

**Figure 7:**
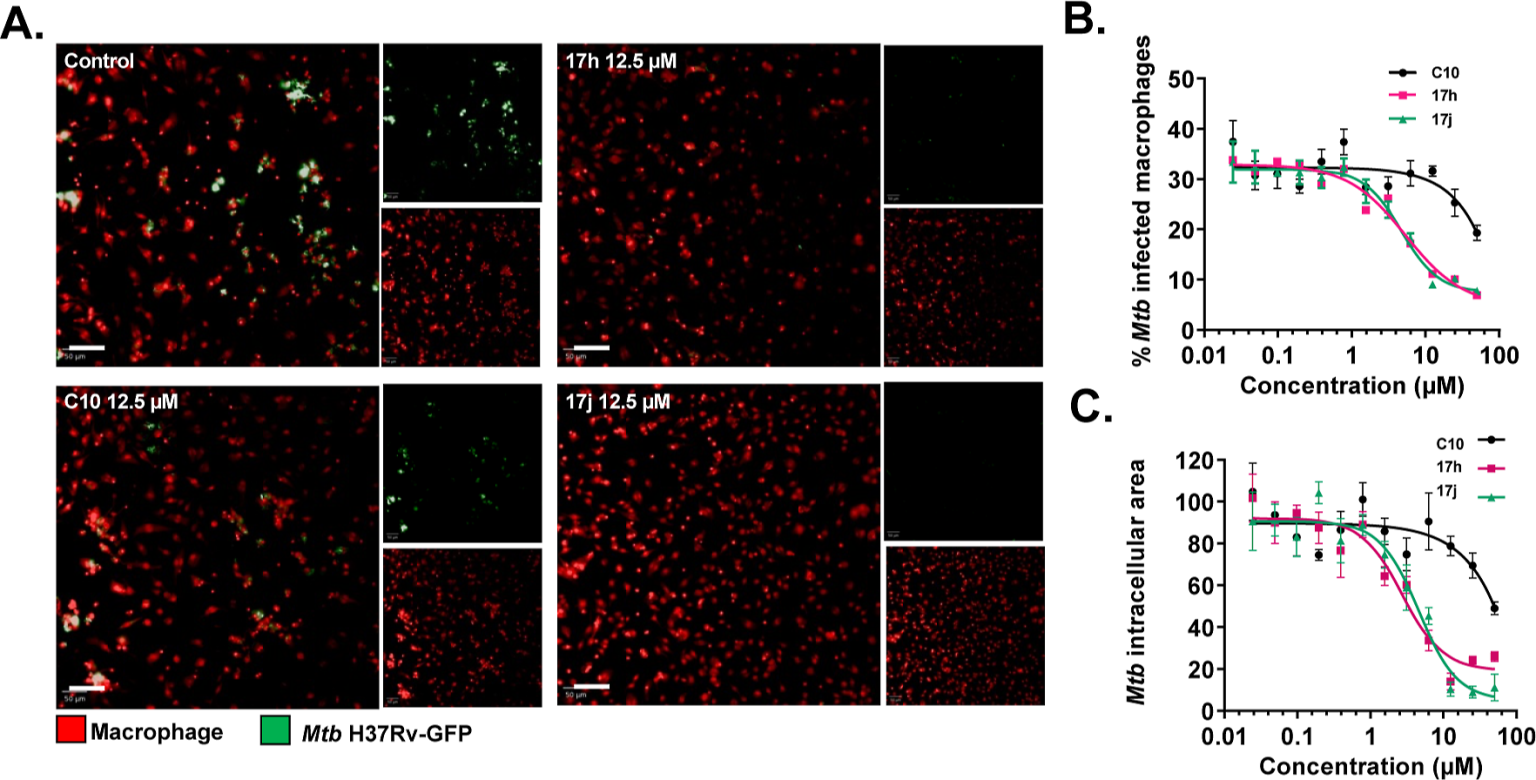
Inhibition of *Mtb* replication within macrophages by compounds **C10**, **17h**, and **17j**. (A) Efficacy of the compounds was assessed using a phenotypic cell-based quantification of intracellular mycobacteria assay. Murine RAW 264.7 macrophages were infected with H37Rv-GFP *Mtb* and treated with either DMSO (control) or individual lead compounds at a concentration of 12.5 μM. Representative images of infected cells (red nuclei) are shown. Scale bar: 50 μM. (B,C) Dose–response analysis was performed for all three compounds, measuring the percentage of infected cells and the bacterial area per infected cell. Active compounds were identified by a percentage of infected cells below 10 (B) and an intracellular bacterial area below 40 (C). The IC_50_ (relative to no treatment) was calculated for each compound using GraphPad Prism. All compounds were dissolved in DMSO. The experiment was performed with four replicates for each compound, and the data points represent mean ± SD.

Macrophages serve as crucial reservoirs for mycobacterium replication during TB infections. Within the host’s lungs, *Mtb* infects and multiplies within macrophages.^24^ Hence, the development of the potential drug candidates targeting *Mtb* necessitates their ability to hinder *Mtb* replication specifically within macrophages without significant toxicity. We examined the activity of **C10**, **17h**, and **17j** against intracellular *Mtb* replication in macrophages using image-based assays that were conducted by treating murine RAW 264.7 macrophages infected with *Mtb* with varying dilutions of the compounds over a 5 day infection period (Figure 7A). The inhibition of intracellular mycobacterial growth was quantified by determining the percentage of infected cells (Figure 7B) and the bacterial area per infected cell (Figure 7C). The results showed that both **17h** and **17j** inhibited intracellular mycobacteria at concentrations lower than **C10**. Moreover, the compounds did not show any cytotoxicity for uninfected murine RAW 264.7 macrophages under similar conditions where compounds **17h** and **17j** inhibited intracellular mycobacteria (Supporting Information Figure S6).

### **17j** Inhibitory Effect on *Mtb* Is Enantioselective

**Figure 8:**
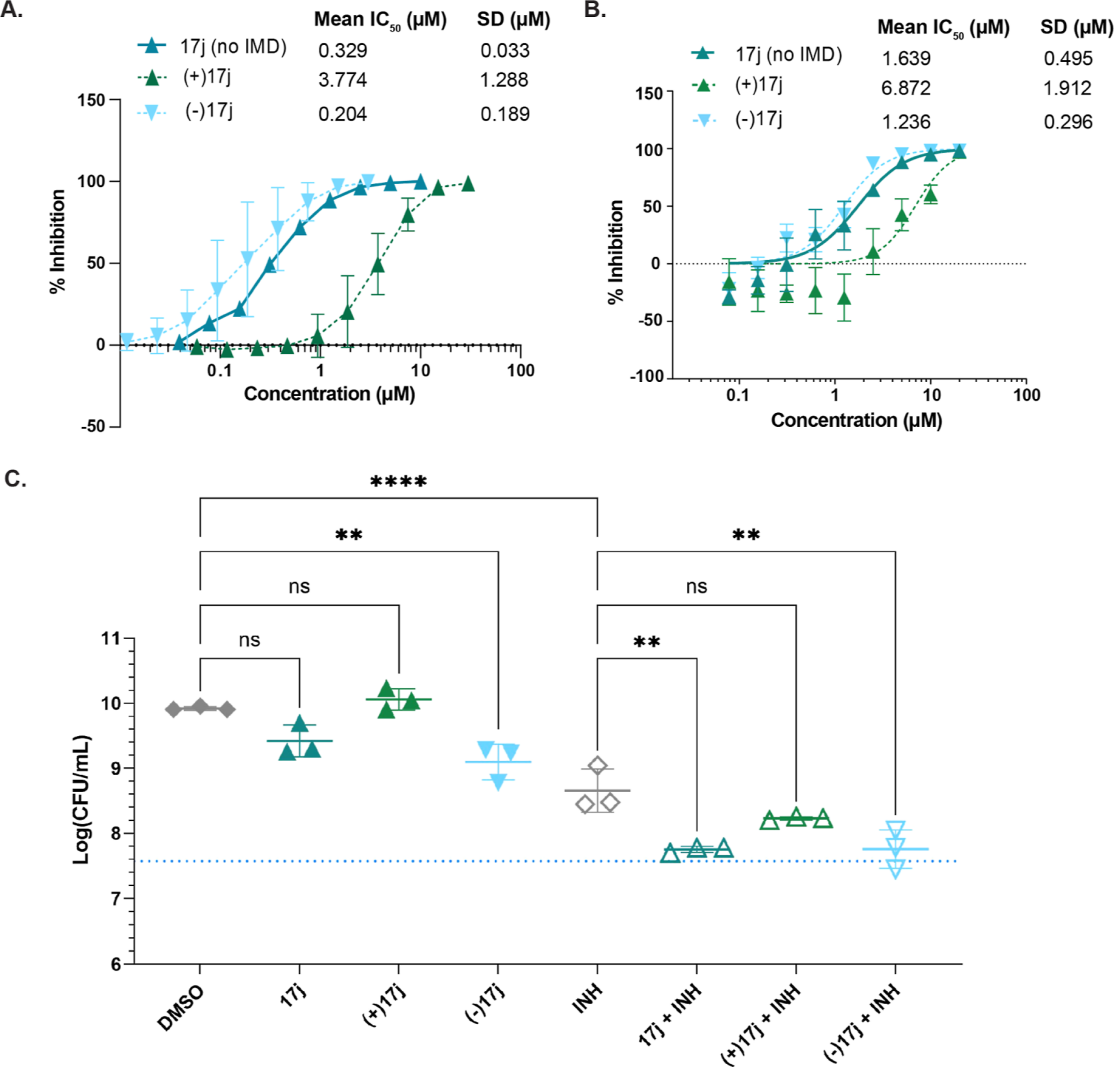
Anti-*Mtb* activity of **17j** is stereoselective. (A) *Mtb* was treated with increasing concentrations of **17j** (without IMD) or the enantiomers of **17j**, **(+)17j**, or **(−)17j**, and monitored for inhibitory activity in the MABA. Using GraphPad Prism, IC_50_ ± SD was calculated from a nonlinear regression for each compound. **17j**, *n* = 2; **(+)17j**, *n* = 3; **(−)17j**, *n* = 3. (B) *Mtb* was incubated in the presence of increasing doses of each compound for 3 weeks under hypoxic conditions, followed by 3 weeks of incubation under aerobic conditions. After 6 weeks total of incubation, the OD_600_ across the pellicle biofilm at the air/liquid interface of each well was measured and plotted by concentration, whereby an IC_50_ (relative to no treatment) was calculated for each compound using GraphPad Prism. (C) *Mtb katG*^*FS*^ was grown to log-phase in Sauton’s media, diluted to 5 × 10^7^ CFU/mL, and treated with 1 μM **17j**, (+)**17j**, or **(−)17j** ± 0.25 μg/mL INH, with triplicate samples per condition. Graphed is the mean CFU/mL ± SD after 10 days of treatment. The dashed blue line denotes the inoculum CFU/mL. Statistical comparisons were performed in GraphPad Prism with an ordinary one-way ANOVA with Tukey’s correction; only comparisons between single treatments and DMSO or INH-cotreatments versus INH alone are shown. **p < 0.01; ****p < 0.0001; ns = not significant. For all experiments, DMSO was included in the untreated controls.

**17j** is the most potent compound from our second-generation ring-fused thiazolo-2-pyridone SAR library in the MABA (Table 3, Figure 4A), the biofilm inhibition assay (Figure 5), and for INH-potentiation in the *Mtb katG*^FS^ mutant (Figure 6). In addition, **17j** exhibited minimal toxicity in a range of relevant cell lines (Figure 4B–E, Table 5). **17j** is a racemic mixture; therefore, we expected one of the enantiomers to have higher activity than the other enantiomer and the racemic mixture. To test this, we separated **17j** into its pure enantiomers using chiral HPLC (Supporting Information Scheme S5, Figures S7–S10) and tested the individual enantiomers for anti-*Mtb* activity in the MABA. It should be noted that the enantiomers were tested in the absence of IMD; therefore, we included **17j** (as a racemic mixture) without IMD as an additional control compound in these assays. We discovered that **(−)17j** (IC_50_ = 0.20 μM) was over 10 times more active than **(+)17j** (IC_50_ = 3.77 μM), and the activity of **17j** (IC_50_ = 0.54 μM) was between that of each of the two enantiomers (Figure 8A). In addition, **(−)17j** was also more potent at inhibiting *Mtb* pellicle biofilm formation (Figure 8B) and at potentiating INH-mediated inhibition of the *Mtb katG*^FS^ strain (Figure 8C) when compared to **(+)17j**, further supporting that **(−)17j** is the more potent **17j** enantiomer. These results showed that the increased potency of racemic **17j** is primarily driven by the **(−)17j** enantiomer.

## Conclusions

The current standard of care for TB relies on long antibiotic regimens that provide the opportunity for the emergence and selection of drug-resistant *Mtb* mutants, which pose a serious threat to the control of TB worldwide.^25^ Despite increased efforts toward TB drug development, there is an unmet medical need for new treatments for TB. Herein, we performed strategic synthesis based on a SAR analysis around the ring-fused thiazolo-2-pyridone scaffold of **C10**,^16^ a compound we previously discovered to inhibit stress-tolerant *Mtb.* The goal of the present study was to enhance the potency of the peptidomimetic ring-fused thiazolo-2-pyridone for its anti-*Mtb* effects through the modification of substituents. By exploring the C7 position of the ring-fused thiazolo-2-pyridone scaffold, we were able to identify compounds **17h** and **17j**, which significantly improved the potency of all previous analogues. In addition, **17h** and **17j** have favorable *in vitro* therapeutic indexes, inhibit biofilm formation, and potently resensitize an *Mtb katG* mutant to inhibition by INH. The isoxazole heterocyclic linker at the C7 position was important for the improved activity of **17h** and **17j**. Moreover, our findings show that **17h** and **17j** possess the capability to inhibit the growth of intracellular *Mtb* within macrophages at lower concentrations compared to **C10**. The isolated **(−)17j** enantiomer showed further improved activity inhibiting *Mtb* growth and biofilm formation compared to the racemic **17j**, while the other **(+)17j** enantiomer still showed improved activity in comparison to **C10**. Collectively, this work shows that an improvement in anti-*Mtb* activity is achievable by fine-tuning the substitution pattern on the peptidomimetic ring-fused thiazolo-2-pyridone scaffold. Our results indicate that **(−)17j** is a promising candidate for further development towards a new class of drugs to treat drug-sensitive and drug-resistant *Mtb* and for use in combination therapy. The distinctive ability of the ring-fused thiazolo-2-pyridone compounds to resensitize INH-resistant mutants to killing by INH highlights a unique strategy to develop a new treatment regimen for *Mtb*. Future studies will also focus on dissecting the precise mode of action of the **(−)17j** enantiomer and testing our lead compounds for in vivo efficacy, both alone and in combination with INH and other antibiotics as well as against both drug-sensitive *Mtb* and drug-resistant *Mtb*. These studies will contribute to new strategies for TB drug development.

## Experimental Section

### General Chemical Procedures

All reagents and solvents were used as received from commercial suppliers without further purification. Microwave reactions were performed in sealed vessels using a Biotage Initiator microwave synthesizer; temperatures were monitored by an internal IR probe. All the necessary reactions were carried out in dry solvents under an inert atmosphere. The reaction progress was monitored on aluminum-based silica gel TLC plates (median pore size 60 Å, fluorescent indicator 254 nm) and detected with UV light at 254 and 366 nm. Flash column chromatography was performed using silica gel (0.063–0.200 mesh). Automated flash column chromatography was performed using a Biotage Isolera One system and purchased prepacked silica gel cartridges (Biotage Sfar, duo 60 μm). Preparative HPLC was performed with a Gilson instrument using a Phenomenex Gemini NX-C18 HTec column (250 mm × 21.5 mm; particle size 5 μm) supported by Phenomenex Security Guard cartridge C18 (15 mm × 21.2 mm). The purity of all final compounds studied in the biological evaluation was 95% or higher as determined by analytical HPLC (Waters Acquity H-Class QS.M). Chiral HPLC separation of **17j** was performed with a Gilson instrument column and a Phenomenex Lux i-Amylose-1 (150 mm × 21.2 mm; particle size 5 μm). Analytical chiral HPLC separation of compound 15i was performed on a Waters Acquity H-Class QSM: column and Phenomenex Lux i-Amylose-1 (250 mm × 4.6 mm; particle size 5 μm) column [eluent A, water; eluent B, acetonitrile with 0.15% trifluoroacetic acid, isocratic (45:55) with a flow rate of 18 mL min^–1^ and detection at 254 nm; column temperature of 25 °C]. ^1^H, ^13^C, and ^19^F NMR spectra were recorded on a Bruker AVANCE III 400 MHz spectrometer with a BBO-F/H Smart probe and a Bruker AVANCE III HD 600 MHz spectrometer with a CP BBO-H/F, 5 mm cryoprobe at 298 K. All spectrometers were operated by Topspin 3.5.7. LC–MS was conducted on a Micromass ZQ mass spectrometer using ES + ionization unless otherwise stated. HRMS was performed on an Agilent mass spectrometer with ESI-TOF (ES+).

### Oxidative Transformation of Benzyl Halide into an Aldehyde

#### Methyl 8-Cyclopropyl-7-formyl-5-oxo-2,3-dihydro-5*H*-thiazolo[3,2-*a*]pyridine-3-carboxylate (**2**)

*N*-Methylmorpholine-*N*-oxide (30.0 mmol, 3.0 equiv) and potassium iodide (3.0 mmol, 0.3 equiv) were added portion-wise to a solution of compound **1** (10.0 mmol, 1.0 equiv) in dry THF (8 mL/mmol) at 0 °C under nitrogen atmosphere. The reaction mixture was stirred for 4 h at reflux under a nitrogen atmosphere. The reaction mixture was allowed to cool down to room temperature, and the solvent was removed under reduced pressure. The reaction mixture was dissolved in H_2_O (50 mL) and extracted with EtOAc (3× 40 mL). The combined organic layer was washed with H_2_O and brine, dried with Na_2_SO_4_, and concentrated in vacuo. The crude product was purified by flash chromatography (heptane/EtOAc 1:1 → 2:8). The pure product was collected as a bright-yellow solid (2.34 g, 8.39 mmol, 84%).^18^^1^H NMR (400 MHz, chloroform-*d*): δ 10.45 (s, 1H), 6.67 (s, 1H), 5.65 (dd, *J* = 8.6, 2.3 Hz, 1H), 3.84 (s, 3H), 3.75 (dd, *J* = 11.8, 8.7 Hz, 1H), 3.59 (dd, *J* = 11.8, 2.3 Hz, 1H), 1.88–1.81 (m, 1H), 1.09–0.98 (m, 2H), 0.68–0.58 (m, 2H). ^13^C NMR (100 MHz, chloroform-*d*): δ 191.17, 168.03, 161.08, 150.65, 146.41, 116.67, 112.23, 63.17, 53.46, 31.81, 9.67, 8.02, 8.00. LCMS (ES+) *m*/*z* calcd for C_13_H_14_NO_4_S^+^ [M + H]^+^: 280.3, found, 280.1.

#### Synthesis of Alkyne from Aldehyde Using the Bestmann–Ohira Reagent

##### Methyl (±)-8-Cyclopropyl-7-ethynyl-5-oxo-2,3-dihydro-5*H*-thiazolo[3,2-*a*]pyridine-3-Carboxylate (**3**)

To a solution of aldehyde **2** (8.0 mmol, 1.0 equiv) in anhydrous methanol (20 mL) were added potassium carbonate (8.0 mmol, 1.0 equiv) and Bestmann–Ohira reagent (9.6 mmol, 1.2 equiv) (Supporting Information Scheme S1). The mixture was stirred at room temperature for 3 h under nitrogen. The reaction was monitored by TLC. Upon completion of the reaction, the solvent was evaporated under reduced pressure. The crude material was diluted with a saturated NH_4_Cl solution (20 mL) and extracted with dichloromethane (3× 20 mL). The organic layer was separated, washed with water and brine, dried over Na_2_SO_4_, and concentrated under reduced pressure. The crude product was purified by flash chromatography (heptane/EtOAc 1:1 → 2:8). The pure product was collected as a bright-yellow solid (1.88 g, 6.84 mmol, 85%). ^1^H NMR (400 MHz, chloroform-*d*): δ 6.44 (s, 1H), 5.58 (dd, *J* = 8.6, 2.3 Hz, 1H), 3.79 (s, 3H), 3.70–3.65 (m, 1H), 3.58–3.39 (m, 2H), 1.68–1.61 (m, 1H), 0.95–0.84 (m, 2H), 0.75–0.61 (m, 2H). ^13^C NMR (100 MHz, chloroform-*d*): δ 168.31, 160.33, 148.48, 137.73, 119.62, 114.37, 86.62, 79.64, 63.01, 53.32, 31.59, 11.69, 7.45, 7.42. LCMS (ES+) *m*/*z* calcd for C_14_H_14_NO_3_S^+^ [M + H]^+^: 276.3; found, 276.1.

##### (±) Methyl 8-Cyclopropyl-7-((hydroxyimino)methyl)-5-oxo-2,3-dihydro-5*H*-thiazolo[3,2-*a*]pyridine-3-carboxylate (**4**)

NH_2_OH·HCl (2.4 mmol, 1.2 equiv) was added to a mixture of aldehyde **2** (2.0 mmol, 1.0 equiv) and NaOAc (3.0 mmol, 1.5 equiv) in MeOH at room temperature. The mixture was stirred at the same temperature for 4 h. The reaction mixture was acidified with 1 N HCl solution to attain neutral pH. The aqueous layer was separated, and the organic layer was filtered off to obtain a yellow solid product (520 mg, 1.76 mmol, 88%). ^1^H NMR (400 MHz, DMSO-*d*_6_): δ 11.77 (s, 1H), 8.25 (s, 1H), 6.21 (s, 1H), 5.41 (dd, *J* = 9.3, 2.3 Hz, 1H), 3.71 (dd, *J* = 12.0, 9.3 Hz, 1H), 3.58 (s, 3H), 3.43 (dd, *J* = 11.9, 2.4 Hz, 1H), 1.57–1.50 (m, 1H), 0.81–0.74 (m, 2H), 0.43–0.29 (m, 2H). ^13^C NMR (100 MHz, DMSO-*d*_6_): δ 169.11, 160.11, 150.36, 146.42, 145.42, 111.25, 109.64, 63.15, 53.34, 31.34, 10.52, 8.23, 8.13. LCMS (ES+) *m*/*z* calcd for C_14_H_14_NO_3_S^+^ [M + H]^+^: 295.3; found, 296.1.

#### General Procedure for the Condensation of Ring-Fused Thiazolo-2-pyridone and 5-Substituted-1,3,4-oxadiazole-2-thiols (**7a–l**)

A solution of 7-(chloromethyl)-ring-fused thiazolo-2-pyridone (0.2 mmol, 1.0 equiv) and tetrabutylammonium bromide (TBAB) (0.01 mmol, 0.05 equiv) in CH_2_Cl_2_ (5 mL) was added to a solution of the corresponding 5-substituted-1,3,4-oxadiazole-2-thiol (**6a–l**) (0.22 mmol, 1.1 equiv) (Supporting Information Scheme S2) with sodium hydroxide (0.25 mmol, 1.25 equiv) in H_2_O (5 mL). The reaction mixture was stirred at room temperature overnight. Upon completion, the reaction mixture was acidified with 1M HCl solution (2× 10 mL), and the organic layer was collected after brine wash (2× 10 mL) and dried over anhydrous sodium sulfate. The organic solvent was removed under reduced pressure, and the compound was purified with flash column chromatography (heptane/EtOAc 1:1 → 1:9).

#### General Procedure of Ester Hydrolysis

The ester was dissolved in THF (2 mL), and LiOH (0.1 M aq; 1.4 equiv) was added while stirring. The reaction mixture was left stirring at room temperature and monitored by TLC (EtOAc). Upon completion, the reaction was quenched with HCl (1 M; 1.5 equiv). The mixture was diluted with EtOAc (15 mL) and washed with brine (10 mL). The aqueous phase was extracted with EtOAc (10 mL). The organic phases were combined, dried, filtered, and evaporated. The residue was dissolved in DMSO (1 mL) and purified with preparative HPLC (H_2_O/MeCN + 0.75% HCOOH; 40–100% in 30 min, 100% for 10 min). The fraction containing the desired product was diluted with H_2_O and freeze-dried.

##### (±) 8-Cyclopropyl-7-(((5-(naphthalen-1-yl)-1,3,4-oxadiazol-2-yl)thio)methyl)-5-oxo-2,3-dihydro-5*H*-thiazolo[3,2-*a*]pyridine-3-carboxylic Acid (**8a**)

Compound **8a** was obtained as a white solid (112 mg, 0.235 mmol, 92%). ^1^H NMR (600 MHz, chloroform-*d*): δ 9.19 (d, *J* = 8.6 Hz, 1H), 8.14 (d, *J* = 7.4 Hz, 1H), 8.06 (d, *J* = 8.2 Hz, 1H), 7.95 (d, *J* = 8.1 Hz, 1H), 7.71 (dd, *J* = 8.4, 6.4 Hz, 1H), 7.61 (dt, *J* = 15.1, 7.6 Hz, 2H), 6.75 (s, 1H), 5.68 (s, 1H), 4.73 (d, *J* = 13.9 Hz, 1H), 4.57 (d, *J* = 13.8 Hz, 1H), 4.03 (d, *J* = 10.5 Hz, 1H), 3.67 (s, 1H), 1.85 (s, 1H), 1.08 (d, *J* = 64.6 Hz, 2H), 0.75 (d, *J* = 25.8 Hz, 2H). ^13^C NMR (151 MHz, chloroform-*d*): δ 167.26, 166.25, 162.78, 162.58, 153.34, 150.53, 133.82, 132.82, 129.80, 128.72, 128.39, 128.26, 126.81, 126.08, 124.90, 119.85, 116.49, 114.51, 64.71, 33.26, 30.03, 11.01, 8.07, 7.36. HRMS (ESI-TOF) *m*/*z* [M + H]^+^ calcd for C_24_H_19_N_3_O_4_S_2_: 478.0890; found, 478.0888.

##### (±) 8-Cyclopropyl-5-oxo-7-(((5-(3-(trifluoromethyl)phenyl)-1,3,4-oxadiazol-2-yl)thio)methyl)-2,3-dihydro-5*H*-thiazolo[3,2-*a*]pyridine-3-carboxylic Acid (**8b**)

Compound **8b** was obtained as a white solid (129 mg, 0.260 mmol, 87%). ^1^H NMR (600 MHz, DMSO-*d*_6_): δ 8.28 (dd, *J* = 7.9, 1.6 Hz, 1H), 8.24 (d, *J* = 1.8 Hz, 1H), 8.03 (dd, *J* = 8.0, 1.7 Hz, 1H), 7.86 (t, *J* = 7.9 Hz, 1H), 6.28 (s, 1H), 5.40 (dd, *J* = 9.1, 1.8 Hz, 1H), 4.65–4.59 (m, 2H), 3.81 (dd, *J* = 11.9, 9.1 Hz, 1H), 3.53 (dd, *J* = 11.9, 1.8 Hz, 1H), 1.74–1.69 (m, 1H), 0.99–0.90 (m, 2H), 0.72–0.58 (m, 2H). ^13^C NMR (151 MHz, DMSO-*d*_6_): δ 169.90, 164.86, 164.05, 160.14, 151.46, 149.83, 131.33, 130.94, 130.56 (q, *J* = 32 Hz), 128.98 (d, *J* = 4.5 Hz), 124.56, 124.05 (q, *J* = 271.8 Hz), 123.33 (q, *J* = 4.5 Hz), 114.69, 111.50, 63.03, 33.66, 31.72, 10.88, 7.80, 7.71. ^19^F NMR (565 MHz, DMSO-*d*_6_): δ −61.48. HRMS (ESI-TOF) *m*/*z* [M + H]^+^ calcd for C_21_H_16_F_3_N_3_O_4_S_2_: 496.0607; found, 496.0616.

##### (±) 8-Cyclopropyl-7-(((5-(naphthalen-1-ylmethyl)-1,3,4-oxadiazol-2-yl)thio)methyl)-5-oxo-2,3-dihydro-5*H*-thiazolo[3,2-*a*]pyridine-3-carboxylic Acid (**8c**)

Compound **8c** was obtained as a white solid (75 mg, 0.153 mmol, 76%). ^1^H NMR (600 MHz, chloroform-*d*): δ 8.11–8.10 (m, 1H), 7.90 (dd, *J* = 8.0, 1.4 Hz, 1H), 7.85 (dd, *J* = 6.2, 3.3 Hz, 1H), 7.59–7.56 (m, 1H), 7.55–7.52 (m, 1H), 7.49–7.46 (m, 2H), 6.55 (s, 1H), 5.65 (dd, *J* = 8.8, 1.5 Hz, 1H), 4.66–4.60 (m, 2H), 4.48 (d, *J* = 14.2 Hz, 1H), 4.32 (d, *J* = 14.2 Hz, 1H), 3.92 (dd, *J* = 11.6, 1.5 Hz, 1H), 3.65 (dd, *J* = 11.6, 8.8 Hz, 1H), 1.69–1.65 (m, 1H), 1.01–0.89 (m, 2H), 0.68–0.59 (m, 2H). ^13^C NMR ^13^C NMR (151 MHz, chloroform-*d*): δ 167.63, 166.87, 163.16, 162.45, 153.00, 150.32, 133.88, 131.59, 129.45, 128.85, 128.74, 127.81, 126.85, 126.12, 125.54, 123.39, 116.02, 114.42, 64.39, 33.10, 30.27, 29.61, 10.81, 7.84, 7.23. HRMS (ESI-TOF) *m*/*z* [M + H]^+^ calcd for C_25_H_21_N_3_O_4_S_2_: 492.1046; found, 492.1053.

##### (±) 8-Cyclopropyl-7-(((5-(naphthalen-2-yl)-1,3,4-oxadiazol-2-yl)thio)methyl)-5-oxo-2,3-dihydro-5*H*-thiazolo[3,2-*a*]pyridine-3-carboxylic Acid (**8d**)

Compound **8d** was obtained as a white solid (93 mg, 0.194 mmol, 93%). ^1^H NMR (600 MHz, DMSO-*d*_6_): δ 8.59 (d, *J* = 1.7 Hz, 1H), 8.17–8.12 (m, 2H), 8.06–8.03 (m, 2H), 7.69–7.64 (m, 2H), 6.33 (s, 1H), 5.42 (dd, *J* = 9.1, 1.7 Hz, 1H), 4.65–4.59 (m, 2H), 3.82 (dd, *J* = 11.9, 9.1 Hz, 1H), 3.54 (dd, *J* = 11.9, 1.8 Hz, 1H), 1.75–1.71 (m, 1H), 1.00–0.91 (m, 2H), 0.72–0.59 (m, 2H). ^13^C NMR (151 MHz, DMSO-*d*_6_): δ 169.94, 166.13, 163.42, 160.20, 151.64, 149.86, 134.67, 132.93, 129.72, 129.42, 128.76, 128.34, 127.83, 127.45, 123.09, 120.70, 114.85, 111.55, 63.07, 33.78, 31.74, 10.92, 7.81, 7.73. HRMS (ESI-TOF) *m*/*z* [M + H]^+^ calcd for C_24_H_19_N_3_O_4_S_2_: 478.0890; found, 478.0887.

##### (±) 8-Cyclopropyl-7-(((5-(naphthalen-2-ylmethyl)-1,3,4-oxadiazol-2-yl)thio)methyl)-5-oxo-2,3-dihydro-5*H*-thiazolo[3,2-*a*]pyridine-3-carboxylic Acid (**8e**)

Compound **8e** was obtained as a white solid (72 mg, 0.146 mmol, 85%). ^1^H NMR (600 MHz, DMSO-*d*_6_): δ 7.92–7.90 (m, 3H), 7.85 (s, 1H), 7.53–7.51 (m, 2H), 7.44 (dd, *J* = 8.5, 1.8 Hz, 1H), 6.19 (s, 1H), 5.39 (dd, *J* = 9.2, 1.8 Hz, 1H), 4.51–4.43 (m, 4H), 3.79 (dd, *J* = 11.9, 9.1 Hz, 1H), 3.52 (dd, *J* = 11.9, 1.9 Hz, 1H), 1.61–1.57 (m, 1H), 0.88–0.79 (m, 2H), 0.62–0.49 (m, 2H). ^13^C NMR (151 MHz, DMSO-*d*_6_): δ 169.91, 167.51, 163.18, 160.12, 151.42, 149.75, 133.44, 132.53, 132.20, 128.83, 128.03 (2C), 127.94, 127.48, 126.88, 126.54, 114.50, 111.44, 63.04, 33.53, 31.72, 31.43, 10.77, 7.72, 7.61. HRMS (ESI-TOF) *m*/*z* [M + H]^+^ calcd for C_25_H_21_N_3_O_4_S_2_: 492.1046; found, 492.1057.

##### (±) 7-(((5-(4-(*tert*-Butyl)phenyl)-1,3,4-oxadiazol-2-yl)thio)methyl)-8-cyclopropyl-5-oxo-2,3-dihydro-5*H*-thiazolo[3,2-*a*]pyridine-3-carboxylic Acid (**8f**)

Compound **8f** was obtained as a white solid (100 mg, 0.206 mmol, 86%). ^1^H NMR (600 MHz, DMSO-*d*_6_): δ 7.92–7.90 (m, 2H), 7.63–7.61 (m, 2H), 6.26 (s, 1H), 5.40 (dd, *J* = 9.2, 1.8 Hz, 1H), 4.61–4.55 (m, 2H), 3.81 (dd, *J* = 11.9, 9.2 Hz, 1H), 3.53 (dd, *J* = 11.9, 1.8 Hz, 1H), 1.73–1.68 (m, 1H), 1.33 (s, 9H)0.98–0.89 (m, 2H), 0.71–0.57 (m, 2H). ^13^C NMR (151 MHz, DMSO-*d*_6_): δ 169.89, 165.97, 162.94, 160.14, 155.53, 151.58, 149.82, 126.81 (2C), 126.75 (2C), 120.71, 114.68, 111.51, 63.03, 35.33, 33.73, 31.73, 31.27 (3C), 10.89, 7.80, 7.69. HRMS (ESI-TOF) *m*/*z* [M + H]^+^ calcd for C_24_H_25_N_3_O_4_S_2_: 484.1359; found, 484.1362.

##### (±) 8-Cyclopropyl-7-(((5-(4-nitrophenyl)-1,3,4-oxadiazol-2-yl)thio)methyl)-5-oxo-2,3-dihydro-5*H*-thiazolo[3,2-*a*]pyridine-3-carboxylic Acid (**8g**)

Compound **8g** was obtained as a white solid (63 mg, 0.133 mmol, 85%). ^1^H NMR (600 MHz, DMSO-*d*_6_): δ 8.41 (d, *J* = 8.9 Hz, 2H), 8.23 (d, *J* = 8.8 Hz, 2H), 6.28 (s, 1H), 5.40 (dd, *J* = 9.1, 1.8 Hz, 1H), 4.64–4.59 (m, 2H), 3.80 (dd, *J* = 11.9, 9.1 Hz, 1H), 3.53 (dd, *J* = 11.9, 1.8 Hz, 1H), 1.73–1.68 (m, 1H), 0.98–0.89 (m, 2H), 0.71–0.57 (m, 2H). ^13^C NMR (151 MHz, DMSO-*d*_6_): δ 169.91, 164.71, 164.62, 160.15, 151.40, 149.86, 149.64, 128.97, 128.32 (2C), 125.06 (2C), 114.74, 111.48, 63.11, 33.72, 31.78, 10.88, 7.80, 7.75. HRMS (ESI-TOF) *m*/*z* [M + H]^+^ calcd for C_20_H_16_N_4_O_6_S_2_: 473.0584; found, 473.0584.

##### (±) 8-Cyclopropyl-7-(((5-(3-methoxyphenyl)-1,3,4-oxadiazol-2-yl)thio)methyl)-5-oxo-2,3-dihydro-5*H*-thiazolo[3,2-*a*]pyridine-3-carboxylic Acid (**8h**)

Compound **8h** was obtained as a white solid (51 mg, 0.111 mmol, 91%). ^1^H NMR (600 MHz, DMSO-*d*_6_): δ 7.56 (dd, *J* = 7.7, 1.4 Hz, 1H), 7.51 (t, *J* = 7.9 Hz, 1H), 7.47 (t, *J* = 2.0 Hz, 1H), 7.21 (dd, *J* = 8.3, 2.6 Hz, 1H), 6.26 (s, 1H), 5.39 (dd, *J* = 9.1, 1.8 Hz, 1H), 4.62–4.56 (m, 2H), 3.85 (s, 3H), 3.80 (dd, *J* = 11.9, 9.2 Hz, 1H), 3.52 (dd, *J* = 11.8, 1.8 Hz, 1H), 1.73–1.68 (m, 1H), 0.99–0.89 (m, 2H), 0.71–0.57 (m, 2H). ^13^C NMR (151 MHz, DMSO-*d*_6_): δ 169.91, 165.82, 163.34, 160.14, 160.12, 151.54, 149.84, 131.24, 124.56, 119.25, 118.71, 114.67, 111.58, 111.49, 63.07, 55.93, 33.69, 31.75, 10.90, 7.80, 7.68. HRMS (ESI-TOF) *m*/*z* [M + H]^+^ calcd for C_21_H_19_N_3_O_5_S_2_: 458.0839; found, 458.0844.

##### (±) 8-Cyclopropyl-5-oxo-7-(((5-(pyridin-4-yl)-1,3,4-oxadiazol-2-yl)thio)methyl)-2,3-dihydro-5*H*-thiazolo[3,2-*a*]pyridine-3-carboxylic Acid (**8i**)

Compound **8i** was obtained as a white solid (118 mg, 0.275 mmol, 87%). ^1^H NMR (600 MHz, DMSO-*d*_6_): δ 8.83–8.82 (m, 2H), 7.91–7.90 (m, 2H), 6.29 (s, 1H), 5.41 (dd, *J* = 9.1, 1.8 Hz, 1H), 4.65–4.59 (m, 2H), 3.82 (dd, *J* = 11.9, 9.1 Hz, 1H), 3.53 (dd, *J* = 11.9, 1.8 Hz, 1H), 1.73–1.69 (m, 1H), 0.98–0.91 (m, 2H), 0.71–0.58 (m, 2H). ^13^C NMR (151 MHz, DMSO-*d*_6_): δ 169.94, 164.75, 164.44, 160.15, 151.43 (2C), 151.41 (2C), 149.84, 130.51, 120.52, 114.75, 111.51, 63.03, 33.69, 31.73, 10.89, 7.81, 7.74. HRMS (ESI-TOF) *m*/*z* [M + H]^+^ calcd for C_19_H_16_N_4_O_4_S_2_: 429.0686; found, 429.0684.

##### (±) 8-Cyclopropyl-5-oxo-7-(((5-(thiophen-2-yl)-1,3,4-oxadiazol-2-yl)thio)methyl)-2,3-dihydro-5*H*-thiazolo[3,2-*a*]pyridine-3-carboxylic Acid (**8j**)

Compound **8j** was obtained as a white solid (34 mg, 0.078 mmol, 92%). ^1^H NMR (600 MHz, chloroform-*d*): δ 7.74 (d, *J* = 3.6 Hz, 1H), 7.58 (d, *J* = 4.8 Hz, 1H), 7.19–7.18 (m, 1H), 6.65 (s, 1H), 5.67 (s, 1H), 4.65 (d, *J* = 13.4 Hz, 1H), 4.49 (d, *J* = 13.6 Hz, 1H), 3.97 (d, *J* = 10.5 Hz, 1H), 3.68 (d, *J* = 9.8 Hz, 1H), 1.81 (s, 1H), 1.10–1.00 (m, 2H), 0.73 (d, *J* = 27.3 Hz, 2H). ^13^C NMR (151 MHz, chloroform-*d*): δ 167.48, 162.49, 162.43, 162.18, 153.30, 150.72, 130.42, 129.99, 128.26, 124.46, 116.51, 114.43, 64.64, 33.38, 30.42, 10.98, 8.03, 7.36. HRMS (ESI-TOF) *m*/*z* [M + H]^+^ calcd for C_18_H_15_N_3_O_4_S_3_: 434.0297; found, 434.0296.

##### (±) 8-Cyclopropyl-7-(((5-(furan-2-yl)-1,3,4-oxadiazol-2-yl)thio)methyl)-5-oxo-2,3-dihydro-5*H*-thiazolo[3,2-*a*]pyridine-3-carboxylic Acid (**8k**)

Compound **8k** was obtained as a white solid (65 mg, 0.155 mmol, 90%). ^1^H NMR (600 MHz, chloroform-*d*): δ 7.66 (d, *J* = 1.7 Hz, 1H), 7.16 (d, *J* = 3.5 Hz, 1H), 6.64–6.61 (m, 2H), 5.68 (dd, *J* = 8.8, 1.6 Hz, 1H), 4.65 (d, *J* = 14.0 Hz, 1H), 4.50 (d, *J* = 14.0 Hz, 1H), 3.90 (d, *J* = 11.2 Hz, 1H), 3.69 (dd, *J* = 11.7, 8.8 Hz, 1H), 1.82–1.77 (m, 1H), 1.11–0.97 (m, 2H), 0.77–0.68 (m, 2H). ^13^C NMR (151 MHz, chloroform-*d*): δ 167.77, 162.37, 162.34, 162.25, 158.92, 153.06, 150.77, 145.91, 138.78, 116.31, 114.38, 112.26, 64.42, 33.36, 30.65, 10.91, 7.91, 7.31. HRMS (ESI-TOF) *m*/*z* [M + H]^+^ calcd for C_18_H_15_N_3_O_5_S_2_: 418.0526; found, 418.0526.

##### (±) 8-Cyclopropyl-7-(((5-((4-fluorophenoxy)methyl)-1,3,4-oxadiazol-2-yl)thio)methyl)-5-oxo-2,3-dihydro-5*H*-thiazolo[3,2-*a*]pyridine-3-carboxylic Acid (**8l**)

Compound **8l** was obtained as a white solid (130 mg, 0.273 mmol, 83%). ^1^H NMR (600 MHz, DMSO-*d*_6_): δ 7.18–7.14 (m, 2H), 7.11–7.08 (m, 2H), 6.22 (s, 1H), 5.40 (dd, *J* = 9.1, 1.8 Hz, 1H), 5.38 (s, 2H), 4.57–4.50 (m, 2H), 3.81 (dd, *J* = 11.9, 9.2 Hz, 1H), 3.53 (dd, *J* = 11.9, 1.8 Hz, 1H), 1.68–1.64 (m, 1H), 0.95–0.86 (m, 2H), 0.68–0.54 (m, 2H). ^13^C NMR (151 MHz, DMSO-*d*_6_): δ 169.90, 164.39, 164.34, 160.12, 158.43, 156.86, 153.98, 151.26, 149.82, 116.93 (d, *J* = 8.2 Hz), 116.57, 116.42, 114.56, 111.44, 63.05, 60.55, 33.55, 31.74, 10.82, 7.77, 7.66. ^19^F NMR (565 MHz, DMSO-*d*_6_): δ −122.35. HRMS (ESI-TOF) *m*/*z* [M + H]^+^ calcd for C_21_H_18_FN_3_O_5_S_2_: 476.0645; found, 476.0758.

##### (±) Methyl 7-(1*H*-benzo[*d*]imidazole-2-yl)-8-cyclopropyl-5-oxo-2,3-dihydro-5*H*-thiazolo[3,2-*a*]pyridine-3-carboxylate (**9**)

To a solution of aldehyde **2** (0.10 mmol, 1.0 equiv) in 1 mL of ethanol was added *o*-phenylenediamine (0.10 mmol, 1.0 equiv). The reaction mixture was refluxed for 24 h. Completion of the reaction was monitored by TLC. After completion of the reaction, the solvent from the reaction mixture was removed under reduced pressure. The crude material was purified by silica gel chromatography (heptane/EtOAc 1:1 → 3:7) to afford the pure compound as a white solid (33 mg, 0.09 mmol, 83%).^1^H NMR (400 MHz, chloroform-*d*): δ 7.62 (dd, *J* = 6.0, 3.3 Hz, 2H), 7.22–7.19 (m, 2H), 6.44 (s, 1H), 5.63 (dd, *J* = 9.0, 2.4 Hz, 1H), 3.80 (dd, *J* = 11.7, 8.9 Hz, 1H), 3.45 (s, 3H), 3.39 (dd, *J* = 11.7, 2.2 Hz, 1H), 1.70–1.64 (m, 1H), 0.51–0.48 (m, 2H), 0.20 to −0.04 (m, 2H). ^13^C NMR (100 MHz, chloroform-*d*): δ 168.24, 160.95, 160.81, 151.36, 151.20, 148.60, 148.46, 145.94, 123.17, 115.15, 115.03, 114.34, 114.10, 63.86, 53.26, 31.65, 12.03, 8.16, 7.99. LCMS (ES+) *m*/*z* calcd for C_19_H_18_N_3_O_3_S^+^ [M + H]^+^: 368.4; found, 368.2.

##### (±) 7-(1*H*-benzo[*d*]imidazole-2-yl)-8-cyclopropyl-5-oxo-2,3-dihydro-5*H*-thiazolo[3,2-*a*]pyridine-3-carboxylic Acid (**10**)

The product was obtained following the general method of ester hydrolysis as a white solid (28 mg, 0.08 mmol, 88%). ^1^H NMR (600 MHz, methanol-*d*_4_): δ 7.95 (dd, *J* = 6.2, 3.1 Hz, 2H), 7.74 (dd, *J* = 6.2, 3.1 Hz, 2H), 6.72 (s, 1H), 5.79 (dd, *J* = 9.1, 1.6 Hz, 1H), 4.03 (dd, *J* = 12.0, 9.1 Hz, 1H), 3.79 (dd, *J* = 12.0, 1.7 Hz, 1H), 2.03–2.00 (m, 1H), 0.80–0.77 (m, 2H), 0.33–0.31 (m, 2H). ^13^C NMR (150 MHz, methanol-*d*_4_): δ 168.84, 160.20, 154.84, 146.22, 138.14, 131.29, 127.10 (2C), 116.33, 114.04 (2C), 111.33, 64.03, 39.06, 31.62, 10.31, 7.10, 6.82. HRMS (ES+) *m*/*z* calcd for C_18_H_16_N_3_O_3_S^+^ [M + H]^+^: 354.0907; found, 354.0913.

##### (±) Methyl 8-Cyclopropyl-7-(((2-hydroxyphenyl)imino)methyl)-5-oxo-2,3-dihydro-5*H*-thiazolo[3,2-*a*]pyridine-3-carboxylate (**11**)

To a solution of aldehyde **2** (0.17 mmol, 1.0 equiv) in ethanol was added 2-aminophenol (0.25 mmol, 1.5 equiv). The reaction mixture was refluxed for 24 h. Completion of the reaction was monitored by TLC. After completion of the reaction, the solvent from the reaction mixture was removed under reduced pressure. The crude material was purified by silica gel chromatography (heptane/EtOAc 1:1 → 3:7) to afford the pure compound as an orange solid (55 mg, 0.15 mmol, 88%). ^1^H NMR (400 MHz, chloroform-*d*): δ 9.14 (s, 1H), 7.33–7.25 (m, 2H), 7.05–7.03 (m, 2H), 6.93 (t, *J* = 7.7 Hz, 1H), 5.70 (dd, *J* = 8.7, 2.1 Hz, 1H), 3.83 (s, 3H), 3.75 (dd, *J* = 11.8, 8.6 Hz, 1H), 3.58 (dd, *J* = 11.8, 2.2 Hz, 1H), 1.84–1.76 (m, 1H), 1.09–0.99 (m, 2H), 0.72–0.62 (m, 2H). ^13^C NMR (100 MHz, chloroform-*d*): δ 168.34, 161.20, 153.16, 152.71, 149.48, 147.79, 134.76, 130.41, 120.18, 116.00, 115.70, 113.14, 112.87, 63.13, 53.39, 31.85, 10.17, 8.08, 8.06. LCMS (ES+) *m*/*z* calcd for C_19_H_19_N_2_O_4_S^+^ [M + H]^+^: 371.4; found, 371.1.

##### (±) Methyl 7-(Benzo[*d*]oxazol-2-yl)-8-cyclopropyl-5-oxo-2,3-dihydro-5*H*-thiazolo[3,2-*a*]pyridine-3-carboxylate (**12**)

To a solution of compound **11** (0.10 mmol, 1.0 equiv) in 1 mL methanol was added iodosobenzene diacetate (0.20 mmol, 2.0 equiv), and the mixture was stirred for 16 h. Completion of the reaction was monitored by TLC. After completion of the reaction, the solvent from the reaction mixture was removed under reduced pressure. The crude material was purified by silica gel chromatography (heptane/EtOAc 1:1 → 3:7) to afford the pure compound as a white solid (32 mg, 0.08 mmol, 80%). ^1^H NMR (600 MHz, chloroform-*d*): δ 7.90–7.73 (m, 1H), 7.61 (dd, *J* = 7.4, 1.6 Hz, 1H), 7.44–7.39 (m, 2H), 6.92 (s, 1H), 5.67 (d, *J* = 7.4 Hz, 1H), 3.83 (s, 3H), 3.74 (d, *J* = 9.3 Hz, 1H), 3.58 (d, *J* = 10.9 Hz, 1H), 2.08–2.03 (m, 1H), 0.84–0.78 (m, 2H), 0.41–0.37 (m, 2H). ^13^C NMR (151 MHz, chloroform-*d*): δ 168.25, 160.37, 150.79, 150.30, 141.77, 141.61, 126.05, 124.84 (2C), 120.80, 117.32, 112.51, 110.87, 63.40, 53.44, 31.85, 12.31, 8.36, 8.31. LCMS (ES+) *m*/*z* calcd for C_19_H_17_N_2_O_4_S^+^ [M + H]^+^: 369.4; found, 369.1.

##### (±) 7-(Benzo[*d*]oxazol-2-yl)-8-cyclopropyl-5-oxo-2,3-dihydro-5*H*-thiazolo[3,2-*a*]pyridine-3-carboxylic Acid (**13**)

The final product was obtained following the general method of ester hydrolysis as a white solid (28 mg, 0.08 mmol, 91%). ^1^H NMR (400 MHz, methanol-*d*_4_): δ 7.59–7.57 (m, 1H), 7.49–7.47 (m, 1H), 7.29–7.20 (m, 2H), 6.57 (s, 1H), 5.48 (dd, *J* = 8.9, 1.7 Hz, 1H), 3.70 (dd, *J* = 11.9, 8.9 Hz, 1H), 3.47 (dd, *J* = 11.9, 1.8 Hz, 1H), 1.83–1.79 (m, 1H), 0.59–0.55 (m, 2H), 0.15–0.10 (m, 2H). ^13^C NMR (100 MHz, methanol-*d*_4_): δ 169.21, 161.13, 160.45, 152.67, 150.83, 141.91, 140.99, 126.25, 124.93, 120.01, 115.25, 113.00, 110.68, 63.85, 31.43, 11.72, 7.52, 7.48. HRMS (ES+) *m*/*z* calcd for C_18_H_15_N_2_O_4_S^+^ [M + H]^+^: 355.0747; found, 355.0757.

#### General Procedure for the One-Pot Synthesis of Triazoles (**14a** and **14c**)

NaN_3_ (0.76 mmol, 1.7 equiv), CuSO_4_·5H_2_O (0.04 mmol, 0.1 equiv), and naphthyl boronic acids (0.45 mmol, 1.0 equiv) were added to a flame-dried round bottom flask and stirred in methanol (3 mL) for 16 h. The reaction mixture was diluted with water (3 mL). Sodium ascorbate (0.22 mmol, 0.5 equiv) and compound **3** (0.45 mmol, 1.0 equiv) were added to the reaction mixture and stirred at room temperature for another 4 h. The reaction mixture was diluted with water (20 mL) and extracted with ethyl acetate (3× 20 mL). The organic layer was separated, washed with water and brine, dried over Na_2_SO_4_, and concentrated under reduced pressure. The crude product was purified by flash chromatography (heptane/EtOAc 1:1 → 100% EtOAc). The pure product was collected as a white solid.

##### (±) Methyl 8-Cyclopropyl-7-(1-(naphthalen-1-yl)-1*H*-1,2,3-triazol-4-yl)-5-oxo-2,3-dihydro-5*H*-thiazolo[3,2-*a*]pyridine-3-carboxylate (**14a**)

The product was obtained as a white solid (100 mg, 0.23 mmol, 50%). ^1^H NMR (600 MHz, chloroform-*d*): δ 8.26 (s, 1H), 8.07 (d, *J* = 8.0 Hz, 1H), 8.00 (d, *J* = 8.0 Hz, 1H), 7.66–7.57 (m, 5H), 7.00 (s, 1H), 5.7 (dd, *J* = 8.7, 2.4 Hz, 1H), 3.84 (s, 3H), 3.77 (dd, *J* = 11.7, 8.6 Hz, 1H), 3.58 (dd, *J* = 11.7, 2.3 Hz, 1H), 1.88–1.84 (m, 1H), 0.94–0.88 (m, 2H), 0.56–0.54 (m, 2H). ^13^C NMR (151 MHz, chloroform-*d*): δ 168.52, 160.89, 149.91, 144.71, 143.77, 134.17, 133.35, 130.70, 128.54, 128.42, 128.08, 127.21, 126.38, 125.06, 123.73, 122.04, 114.27, 112.03, 63.29, 53.35, 31.76, 12.14, 9.54, 9.45. LCMS (ES+) *m*/*z* calcd for C_24_H_21_N_4_O_3_S [M + H]^+^: 445.51; found, 445.10.

##### (±) Methyl 8-Cyclopropyl-7-(1-(naphthalen-2-yl)-1*H*-1,2,3-triazol-4-yl)-5-oxo-2,3-dihydro-5*H*-thiazolo[3,2-*a*]pyridine-3-carboxylate (**14c**)

The product was obtained as a white solid (144 mg, 0.32 mmol, 72%). ^1^H NMR (600 MHz, chloroform-*d*): δ 8.41 (s, 1H), 8.24 (d, *J* = 2.1 Hz, 1H), 8.06 (d, *J* = 8.8 Hz, 1H), 7.99–7.95 (m, 3H), 7.64–7.60 (m, 2H), 6.91 (s, 1H), 5.71 (dd, *J* = 8.6, 2.2 Hz, 1H), 3.85 (s, 3H), 3.77 (dd, *J* = 11.6, 2.3 Hz, 1H), 3.59 (dd, *J* = 11.6, 2.3 Hz, 1H), 1.97–1.93 (m, 1H), 0.94–0.89 (m, 2H), 0.52–0.48 (m, 2H). ^13^C NMR (151 MHz, chloroform-*d*): δ 168.52, 160.89, 149.81, 144.65, 134.15, 133.21, 133.00, 130.21, 128.50, 128.31, 128.00, 127.62, 127.20, 121.61, 118.85, 118.59, 114.44, 112.10, 63.26, 53.36, 31.77, 12.17, 9.45, 9.40. LCMS (ES+) *m*/*z* calcd for C_24_H_21_N_4_O_3_S [M + H]^+^: 445.51; found, 445.10.

#### Procedure for the Synthesis of Triazoles (**14b** and **14d**)

Compound **3** (0.35 mmol, 1.0 equiv) and the crude aromatic azide (0.35 mmol, 1.0 equiv) (Supporting Information Scheme S3) were suspended in a mixture of ^*t*^BuOH/H_2_O (1:1). Freshly prepared sodium ascorbate (0.035 mmol, 0.1 equiv) was added, followed by the addition of CuSO_4_·5H_2_O (0.035 mmol, 0.1 equiv). The resulting mixture was heated to 40 °C and vigorously stirred for 24 h. The reaction mixture was diluted with water (20 mL) and extracted with ethyl acetate (3× 20 mL). The organic layer was separated, washed with water and brine, dried over Na_2_SO_4_, and concentrated under reduced pressure. The crude product was purified by flash chromatography (heptane/EtOAc 1:1 → 100% EtOAc). The pure product was collected as a white solid.

##### (±) Methyl 8-Cyclopropyl-5-oxo-7-(1-(3-(trifluoromethyl)phenyl)-1*H*-1,2,3-triazol-4-yl)-2,3-dihydro-5*H*-thiazolo[3,2-*a*]pyridine-3-carboxylate (**14b**)

The product was obtained as a white solid 83 mg (0.18 mmol, 52%). ^1^H NMR (600 MHz, chloroform-*d*): δ 8.43 (s, 1H), 8.10 (s, 1H), 8.04 (dd, *J* = 7.1, 2.1 Hz, 1H), 7.75–7.71 (m, 2H), 6.80 (s, 1H), 5.67 (dd, *J* = 8.7, 2.3 Hz, 1H), 3.81 (s, 3H), 3.77 (dd, *J* = 11.7, 8.7 Hz, 1H), 3.56 (dd, *J* = 11.7, 2.2 Hz, 1H), 1.91–1.85 (m, 1H), 0.88–0.84 (m, 2H), 0.45–0.40 (m, 2H). ^13^C NMR (151 MHz, chloroform-*d*): δ 168.50, 160.75, 150.04, 145.06, 144.23, 137.10, 132.48 (q, *J* = 33.3 Hz), 130.74, 125.59 (q, *J* = 3.7 Hz), 123.59, 123.28 (q, *J* = 273.3 Hz), 121.49, 117.44 (q, *J* = 3.9 Hz), 114.37, 111.88, 63.24, 53.30, 31.71, 12.03, 9.40, 9.34. ^19^F NMR (565 MHz, chloroform-*d*): δ −62.82. LCMS (ES+) *m*/*z* calcd for C_21_H_18_F_3_N_4_O_3_S [M + H]^+^: 463.45; found, 463.20.

##### (±) Methyl 7-(1-(4-(*tert*-Butyl)phenyl)-1*H*-1,2,3-triazol-4-yl)-8-cyclopropyl-5-oxo-2,3-dihydro-5*H*-thiazolo[3,2-*a*]pyridine-3-carboxylate (**14d**)

The product was obtained as a white solid (92 mg, 0.20 mmol, 58%). ^1^H NMR (600 MHz, chloroform-*d*): δ 8.30 (s, 1H), 7.69 (d, *J* = 8.7 Hz, 2H), 7.54 (d, *J* = 8.6 Hz, 2H), 6.83 (s, 1H), 5.65 (dd, *J* = 8.7, 2.4 Hz, 1H), 3.79 (s, 3H), 3.75 (dd, *J* = 11.7, 8.6 Hz, 1H), 3.53 (dd, *J* = 11.7, 2.3 Hz, 1H), 1.87–1.83 (m, 1H), 1.35 (s, 9H), 0.85–0.82 (m, 2H), 0.43–0.40 (m, 2H). ^13^C NMR (151 MHz, chloroform-*d*): δ 168.56, 160.83, 152.39, 149.79, 144.65, 144.37, 134.32, 126.73, 121.66, 121.63, 120.21, 114.16, 114.12, 111.87, 63.22, 53.26, 34.80, 31.70, 31.24 (3C), 12.07, 9.43, 9.39. LCMS (ES+) *m*/*z* calcd for C_24_H_27_N_4_O_3_S [M + H]^+^: 451.56; found, 451.10.

##### (±) 8-Cyclopropyl-7-(1-(naphthalen-1-yl)-1*H*-1,2,3-triazol-4-yl)-5-oxo-2,3-dihydro-5*H*-thiazolo[3,2-*a*]pyridine-3-carboxylic Acid (**15a**)

Hydrolyzed compound **15a** was obtained as a white solid (42 mg, 0.098 mmol, 44%) after HPLC purification. ^1^H NMR (600 MHz, DMSO-*d*_6_): δ 9.12 (s, 1H), 8.24 (d, *J* = 8.3 Hz, 1H), 8.16 (dd, *J* = 7.6, 1.7 Hz, 1H), 7.83 (d, *J* = 7.2 Hz, 1H), 7.75 (t, *J* = 7.8 Hz, 1H), 7.70–7.66 (m, 2H), 7.56–7.55 (m, 1H), 6.67 (s, 1H), 5.53 (dd, *J* = 9.2, 1.8 Hz, 1H), 3.89 (dd, *J* = 11.9, 9.2 Hz, 1H), 3.59 (dd, *J* = 11.9, 1.8 Hz, 1H), 1.97–1.93 (m, 1H), 0.90–0.84 (m, 2H), 0.43–0.34 (m, 2H). ^13^C NMR (151 MHz, DMSO-*d*_6_): δ 170.02, 160.21, 151.18, 144.74, 143.28, 134.12, 133.49, 130.98, 128.86, 128.63, 128.52, 127.70, 125.94, 124.69, 122.35, 112.80, 110.73, 99.98, 63.40, 31.77, 12.04, 9.81, 9.64. HRMS (ES+) *m*/*z* calcd for C_23_H_19_N_4_O_3_S^+^ [M + H]^+^: 431.1172; found, 431.1171.

##### (±) 8-Cyclopropyl-5-oxo-7-(1-(3-(trifluoromethyl)phenyl)-1*H*-1,2,3-triazol-4-yl)-2,3-dihydro-5*H*-thiazolo[3,2-*a*]pyridine-3-carboxylic Acid (**15b**)

Hydrolyzed compound **15b** was obtained as a white solid (38 mg, 0.087 mmol, 47%) after HPLC purification. ^1^H NMR (600 MHz, DMSO-*d*_6_): δ 9.38 (s, 1H), 8.36–8.34 (m, 2H), 7.93–7.89 (m, 2H), 6.60 (s, 1H), 5.52 (dd, *J* = 9.1, 1.7 Hz, 1H), 3.89 (dd, *J* = 11.8, 9.2 Hz, 1H), 3.59 (dd, *J* = 11.8, 1.8 Hz, 1H), 2.01–1.97 (m, 1H), 0.91–0.84 (m, 2H), 0.34–0.26 (m, 2H). ^13^C NMR (151 MHz, DMSO-*d*_6_): δ 169.99, 160.13, 151.24, 144.46, 144.39, 137.37, 131.05 (q, *J* = 32.5 Hz), 125.95 (q, *J* = 3.8 Hz), 124.74, 124.13, 124.05 (q, *J* = 273.3 Hz), 117.40 (q, *J* = 4.5 Hz), 117.36, 112.95, 110.63, 63.41, 31.77, 12.04, 9.68, 9.54. ^19^F NMR (565 MHz, DMSO-*d*_6_): δ −61.15. HRMS (ES+) *m*/*z* calcd for C_20_H_16_F_3_N_4_O_3_S^+^ [M + H]^+^: 449.0890; found, 449.0891.

##### (±) 8-Cyclopropyl-7-(1-(naphthalen-2-yl)-1*H*-1,2,3-triazol-4-yl)-5-oxo-2,3-dihydro-5*H*-thiazolo[3,2-*a*]pyridine-3-carboxylic Acid (**15c**)

Hydrolyzed compound **15c** was obtained as a white solid (48 mg, 0.111 mmol, 39%) after HPLC purification. ^1^H NMR (600 MHz, DMSO-*d*_6_): δ 9.35 (s, 1H), 8.56 (d, *J* = 2.2 Hz, 1H), 8.21 (d, *J* = 8.9 Hz, 1H), 8.15 (dd, *J* = 8.8, 2.2 Hz, 1H), 8.10 (d, *J* = 8.0 Hz, 1H), 8.06 (d, *J* = 7.9 Hz, 1H), 7.72–7.68 (m, 2H), 6.63 (s, 1H), 5.52 (dd, *J* = 9.2, 1.7 Hz, 1H), 3.89 (dd, *J* = 11.8, 9.1 Hz, 1H), 3.59 (dd, *J* = 11.8, 1.8 Hz, 1H), 2.05–2.01 (m, 1H), 0.93–0.88 (m, 2H), 0.37–0.28 (m, 2H). ^13^C NMR (151 MHz, DMSO-*d*_6_): δ 170.01, 160.17, 151.17, 144.60, 144.23, 134.35, 133.31, 132.93, 130.48, 128.76, 128.39, 128.02, 127.56, 124.05, 119.31, 118.60, 112.82, 110.67, 63.39, 31.76, 12.07, 9.75, 9.60. HRMS (ES+) *m*/*z* calcd for C_23_H_19_N_4_O_3_S^+^ [M + H]^+^: 431.1172; found, 431.1171.

##### (±) 7-(1-(4-(*tert*-Butyl)phenyl)-1*H*-1,2,3-triazol-4-yl)-8-cyclopropyl-5-oxo-2,3-dihydro-5*H*-thiazolo[3,2-*a*]pyridine-3-carboxylic Acid (**15d**)

Hydrolyzed compound **15d** was obtained as a white solid (32 mg, 0.073 mmol, 37%) after HPLC purification. ^1^H NMR (600 MHz, DMSO-*d*_6_): δ 9.17 (s, 1H), 7.88 (d, *J* = 8.7 Hz, 2H), 7.65 (d, *J* = 8.7 Hz, 2H), 6.60 (s, 1H), 5.52 (dd, *J* = 9.2, 1.7 Hz, 1H), 3.88 (dd, *J* = 11.8, 9.2 Hz, 1H), 3.59 (dd, *J* = 11.8, 1.8 Hz, 1H), 2.01–1.97 (m, 1H), 1.35 (s, 9H), 0.88–0.84 (m, 2H), 0.34–0.26 (m, 2H). ^13^C NMR (151 MHz, DMSO-*d*_6_): δ 170.01, 160.17, 152.10, 151.12, 144.66, 143.99, 134.59, 127.12 (2C), 123.87, 120.55 (2C), 112.73, 110.67, 63.38, 35.01, 31.75, 31.46 (3C), 12.05, 9.72, 9.57. HRMS (ES+) *m*/*z* calcd for C_23_H_25_N_4_O_3_S^+^ [M + H]^+^: 437.1642; found, 437.1645.

#### General Synthesis of Isoxazoles by Copper Catalysis

The oxime (0.315 mmol, 1.05 equiv) (Supporting Information Scheme S3) was suspended in ^*t*^BuOH/H_2_O (1:1, v/v) (1.5 mL) under a nitrogen atmosphere, and chloramine-T·3H_2_O (0.315 mmol, 1.05 equiv) was added in small portions over 10 min, followed by the addition of CuSO_4_·5H_2_O (0.09 mmol, 0.30 equiv) and copper turnings (0.15 mmol, 0.50 equiv). A suspension of the alkyne (0.3 mmol, 1.0 equiv) in ^*t*^BuOH/H_2_O (1:1, v/v) (1 mL) was added to the reaction mixture and stirred overnight under a nitrogen atmosphere. The reaction mixture was quenched with NH_4_Cl (1 M) and extracted with water and CH_2_Cl_2_ (3× 10 mL). The combined organic layer was washed with brine and dried over Na_2_SO_4_ and concentrated under reduced pressure. The crude material was purified by silica gel chromatography (heptane/EtOAc 1:1 → 1:9) to afford the pure compound.

##### (±) 8-Cyclopropyl-7-(3-(naphthalen-1-yl)isoxazol-5-yl)-5-oxo-2,3-dihydro-5*H*-thiazolo[3,2-*a*]pyridine-3-carboxylic Acid (**17a**)

The product was obtained following the two consecutive steps of general synthesis of isoxazole and ester hydrolysis as a white solid (54 mg, 0.13 mmol, 31%). ^1^H NMR (600 MHz, DMSO-*d*_6_): δ 8.49–8.48 (m, 1H), 8.14 (d, *J* = 8.2 Hz, 1H), 8.08 (dd, *J* = 7.7, 1.7 Hz, 1H), 7.91 (dd, *J* = 7.1, 1.2 Hz, 1H), 7.70–7.63 (m, 3H), 7.56 (s, 1H), 6.58 (s, 1H), 5.55 (dd, *J* = 9.2, 1.7 Hz, 1H), 3.91 (dd, *J* = 11.9, 9.2 Hz, 1H), 3.63 (dd, *J* = 11.8, 1.8 Hz, 1H), 2.00–1.95 (m, 1H), 0.94–0.87 (m, 2H), 0.47–0.37 (m, 2H). ^13^C NMR (151 MHz, DMSO-*d*_6_): δ 167.64, 164.37, 160.82, 157.66, 150.28, 138.86, 131.81, 128.90, 128.49, 127.01, 126.65, 125.80, 124.86, 124.08, 123.84, 123.64, 111.74, 108.00, 105.74, 61.48, 29.75, 9.80, 7.09, 6.89. HRMS (ES+) *m*/*z* calcd for C_24_H_19_N_2_O_4_S^+^ [M + H]^+^: 431.1060; found, 431.1059.

##### (±) 8-Cyclopropyl-5-oxo-7-(3-(3-(trifluoromethyl)phenyl)isoxazol-5-yl)-2,3-dihydro-5*H*-thiazolo[3,2-*a*]pyridine-3-carboxylic Acid (**17b**)

The product was obtained following the two consecutive steps of general synthesis of isoxazole and ester hydrolysis as a white solid (185 mg, 0.41 mmol, 48%). ^1^H NMR (600 MHz, DMSO-*d*_6_): δ 8.30–8.29 (m, 2H), 7.93 (d, *J* = 7.8 Hz, 1H), 7.82 (t, *J* = 7.9 Hz, 1H), 7.78 (s, 1H), 6.51 (s, 1H), 5.54 (dd, *J* = 9.2, 1.8 Hz, 1H), 3.91 (dd, *J* = 11.9, 9.2 Hz, 1H), 3.62 (dd, *J* = 11.9, 1.8 Hz, 1H), 1.96–1.91 (m, 1H), 0.91–0.85 (m, 2H), 0.40–0.31 (m, 2H). ^13^C NMR (151 MHz, DMSO-*d*_6_): δ 169.77, 167.78, 161.64, 159.72, 152.46, 140.82, 131.11, 130.97, 130.50 (q, *J* = 32.1 Hz), 129.79, 127.54 (d, *J* = 3.8 Hz), 124.38 (q, *J* = 272.5 Hz), 123.69 (q, *J* = 3.8 Hz), 113.85, 110.00, 104.87, 63.57, 31.84, 11.86, 9.14, 8.97. ^19^F NMR (565 MHz, methanol-*d*_4_): δ −61.19. HRMS (ES+) *m*/*z* calcd for C_21_H_16_F_3_N_2_O_4_S^+^ [M + H]^+^: 449.0777; found, 449.0784.

##### (±) 8-Cyclopropyl-7-(3-cyclopropylisoxazol-5-yl)-5-oxo-2,3-dihydro-5*H*-thiazolo[3,2-*a*]pyridine-3-carboxylic Acid (**17c**)

The product was obtained following the two consecutive steps of general synthesis of isoxazole and ester hydrolysis as a white solid (138 mg, 0.40 mmol, 68%). ^1^H NMR (600 MHz, methanol-*d*_4_): δ 6.67 (s, 1H), 6.53 (s, 1H), 5.69 (dd, *J* = 8.9, 1.7 Hz, 1H), 3.91 (dd, *J* = 11.9, 8.9 Hz, 1H), 3.68 (dd, *J* = 11.9, 1.7 Hz, 1H), 2.12–2.05 (m, 1H), 1.88–1.83 (m, 1H), 1.14–1.11 (m, 2H), 0.93–0.88 (m, 4H), 0.45–0.39 (m, 2H). ^13^C NMR (150 MHz, methanol-*d*_4_): δ 169.27, 167.12, 165.94, 161.33, 152.43, 141.96, 112.75, 112.17, 103.40, 63.75, 31.34, 11.26, 8.17, 8.12, 7.30, 7.28, 6.70. HRMS (ES+) *m*/*z* calcd for C_17_H_17_N_2_O_4_S^+^ [M + H]^+^: 345.0904; found, 345.0904.

##### (±) 7-(3-Cyclohexylisoxazol-5-yl)-8-cyclopropyl-5-oxo-2,3-dihydro-5*H*-thiazolo[3,2-*a*]pyridine-3-carboxylic Acid (**17d**)

The product was obtained following the two consecutive steps of general synthesis of isoxazole and ester hydrolysis as a white solid (95 mg, 0.25 mmol, 52%). ^1^H NMR (600 MHz, methanol-*d*_4_): δ 6.86 (d, *J* = 1.4 Hz, 1H), 6.55 (d, *J* = 1.4 Hz, 1H), 5.69 (dd, *J* = 9.1, 1.7 Hz, 1H), 3.93–3.89 (m, 1H), 3.68 (dd, *J* = 11.8, 1.7 Hz, 1H), 2.86–2.81 (m, 1H), 2.05–2.02 (m, 2H), 1.90–1.85 (m, 3H), 1.80–1.77 (m, 1H), 1.60–1.44 (m, 4H), 1.40–1.32 (m, 1H), 0.91–0.88 (m, 2H), 0.44–0.39 (m, 2H). ^13^C NMR (150 MHz, methanol-*d*_4_): δ 169.34, 168.87, 165.70, 161.36, 152.44, 142.06, 112.68, 112.18, 104.58, 63.81, 53.39, 48.16, 35.88, 31.67, 31.36, 25.62, 25.53, 11.28, 8.19, 8.15. HRMS (ES+) *m*/*z* calcd for C_20_H_23_N_2_O_4_S^+^ [M + H]^+^: 387.1373; found, 387.1378.

##### (±) 8-Cyclopropyl-7-(3-(4-methoxyphenyl)isoxazol-5-yl)-5-oxo-2,3-dihydro-5*H*-thiazolo[3,2-*a*]pyridine-3-carboxylic Acid (**17e**)

The product was obtained following the two consecutive steps of general synthesis of isoxazole and ester hydrolysis as a white solid (62 mg, 0.15 mmol, 39%). ^1^H NMR (600 MHz, DMSO-*d*_6_): δ 13.59 (s, 1H), 7.91–7.88 (m, 2H), 7.54 (s, 1H), 7.12–7.10 (m, 2H), 6.47 (s, 1H), 5.52 (dd, *J* = 9.2, 1.8 Hz, 1H), 3.89 (dd, *J* = 11.9, 9.2 Hz, 1H), 3.84 (s, 3H), 3.61 (dd, *J* = 11.9, 1.8 Hz, 1H), 1.95–1.90 (m, 1H), 0.89–0.84 (m, 2H), 0.40–0.30 (m, 2H). ^13^C NMR (150 MHz, DMSO-*d*_6_): δ 169.77, 166.90, 162.28, 161.35, 159.76, 152.31, 141.08, 128.69 (2C), 121.06, 115.04 (2C), 113.65, 110.03, 104.46, 63.60, 55.82, 31.86, 11.91, 9.18, 9.01. HRMS (ES+) *m*/*z* calcd for C_21_H_19_N_2_O_5_S^+^ [M + H]^+^: 411.1009; found, 411.1012.

##### (±) 8-Cyclopropyl-7-(3-(4-nitrophenyl)isoxazol-5-yl)-5-oxo-2,3-dihydro-5*H*-thiazolo[3,2-*a*]pyridine-3-carboxylic Acid (**17f**)

The product was obtained following the two consecutive steps of general synthesis of isoxazole and ester hydrolysis as a bright yellow solid (66 mg, 0.15 mmol, 34%). ^1^H NMR (600 MHz, DMSO-*d*_6_): δ 13.59 (s, 1H), 8.43–8.41 (m, 2H), 8.27–8.25 (m, 2H), 7.79 (s, 1H), 6.52 (s, 1H), 5.54 (dd, *J* = 9.2, 1.8 Hz, 1H), 3.91 (dd, *J* = 11.9, 9.2 Hz, 1H), 3.62 (dd, *J* = 11.9, 1.8 Hz, 1H), 1.96–1.91 (m, 1H), 0.91–0.85 (m, 2H), 0.41–0.31 (m, 2H). ^13^C NMR (150 MHz, DMSO-*d*_6_): δ 169.77, 168.06, 161.29, 159.69, 152.52, 148.99, 140.68, 134.73, 128.51 (2C), 124.90 (2C), 113.92, 109.97, 105.16, 63.58, 31.86, 11.86, 9.16, 8.98. HRMS (ES+) *m*/*z* calcd for C_20_H_16_N_3_O_6_S^+^ [M + H]^+^: 426.0754; found, 426.0748.

##### (±) 8-Cyclopropyl-5-oxo-7-(3-phenylisoxazol-5-yl)-2,3-dihydro-5*H*-thiazolo[3,2-*a*]pyridine-3-carboxylic Acid (**17g**)

The product was obtained following the two consecutive steps of general synthesis of isoxazole and ester hydrolysis as a white solid (53 mg, 0.139 mmol, 68%). ^1^H NMR (600 MHz, DMSO-*d*_6_): δ 13.59 (s, 1H), 7.96 (dd, *J* = 7.7, 1.9 Hz, 2H), 7.61 (s, 1H), 7.57–7.55 (m, 3H), 6.49 (s, 1H), 5.53 (dd, *J* = 9.2, 1.8 Hz, 1H), 3.89 (dd, *J* = 11.8, 9.2 Hz, 1H), 3.62 (dd, *J* = 11.8, 1.8 Hz, 1H), 1.95–1.91 (m, 1H), 0.89–0.85 (m, 2H), 0.40–0.30 (m, 2H). ^13^C NMR (150 MHz, DMSO-*d*_6_): δ 167.63, 165.12, 160.55, 157.63, 150.25, 138.85, 128.81, 127.54, 126.58, 125.05, 111.61, 107.89, 102.57, 61.52, 29.76, 9.77, 7.04, 6.87. HRMS (ES+) *m*/*z* calcd for C_20_H_17_N_2_O_4_S^+^ [M + H]^+^: 381.0904; found, 381.0912.

##### (±) 8-Cyclopropyl-7-(3-(naphthalen-2-yl)isoxazol-5-yl)-5-oxo-2,3-dihydro-5*H*-thiazolo[3,2-*a*]pyridine-3-carboxylic Acid (**17h**)

The product was obtained following the two consecutive steps of general synthesis of isoxazole and ester hydrolysis as a white solid (141 mg, 0.33 mmol, 50%). ^1^H NMR (600 MHz, DMSO-*d*_6_): δ 8.57 (s, 1H), 8.11–8.01 (m, 4H), 7.75 (d, *J* = 1.3 Hz, 1H), 7.68–7.60 (m, 2H), 6.53 (d, *J* = 1.3 Hz, 1H), 5.53 (d, *J* = 9.0 Hz, 1H), 3.92–3.89 (m, 1H), 3.62 (dd, *J* = 11.8, 1.9 Hz, 1H), 1.98–1.95 (m, 1H), 0.93–0.89 (m, 2H), 0.43–0.34 (m, 2H). ^13^C NMR (100 MHz, DMSO-*d*_6_): δ 169.77, 167.29, 162.74, 159.76, 152.40, 141.01, 134.15, 133.30, 129.38, 128.90, 128.30, 127.83, 127.47, 127.18, 126.16, 124.06, 113.76, 110.05, 104.89, 63.59, 31.86, 11.94, 9.22, 9.05. HRMS (ES+) *m*/*z* calcd for C_24_H_19_N_2_O_4_S^+^ [M + H]^+^: 431.1060; found, 431.1058.

##### (±) 7-(3-(Anthracen-9-yl)isoxazol-5-yl)-8-cyclopropyl-5-oxo-2,3-dihydro-5*H*-thiazolo[3,2-*a*]pyridine-3-carboxylic Acid (**17i**)

The product was obtained following the two consecutive steps of general synthesis of isoxazole and ester hydrolysis as a white solid (51 mg, 0.11 mmol, 42%). ^1^H NMR (400 MHz, DMSO-*d*_6_): δ 8.87 (s, 1H), 8.25–8.22 (m, 2H), 7.86–7.83 (m, 2H), 7.63–7.58 (m, 4H), 7.44 (s, 1H), 6.65 (s, 1H), 5.57 (dd, *J* = 9.1, 1.8 Hz, 1H), 3.92 (dd, *J* = 11.9, 9.2 Hz, 1H), 3.64 (dd, *J* = 11.9, 1.8 Hz, 1H), 1.98–92 (m, 1H), 0.94–0.89 (m, 2H), 0.57–0.44 (m, 2H). ^13^C NMR (150 MHz, DMSO-*d*_6_): δ 169.78, 167.14, 161.07, 159.82, 152.49, 141.04, 131.15 (2C), 130.43 (2C), 129.57, 129.13 (2C), 127.58 (2C), 126.21 (2C), 125.50 (2C), 122.89, 114.06, 110.15, 110.02, 63.72, 31.93, 11.91, 9.27, 9.02. HRMS (ES+) *m*/*z* calcd for C_28_H_21_N_2_O_4_S^+^ [M + H]^+^: 481.1217; found, 481.1220.

##### (±) 7-(3-(4-(*tert*-Butyl)phenyl)isoxazol-5-yl)-8-cyclopropyl-5-oxo-2,3-dihydro-5*H*-thiazolo[3,2-*a*]pyridine-3-carboxylic Acid (**17j**)

The product was obtained following the two consecutive steps of general synthesis of isoxazole and ester hydrolysis as a white solid (83 mg, 0.19 mmol, 53%). ^1^H NMR (600 MHz, DMSO-*d*_6_): δ 13.58 (s, 1H), 7.88 (d, *J* = 8.4 Hz, 2H), 7.58–7.57 (m, 3H), 6.49 (s, 1H), 5.53 (dd, *J* = 9.2, 1.7 Hz, 1H), 3.90 (dd, *J* = 11.9, 9.2 Hz, 1H), 3.61 (dd, *J* = 11.9, 1.8 Hz, 1H), 1.96–1.91 (m, 1H), 1.33 (s, 9H), 0.90–0.83 (m, 2H), 0.40–0.30 (m, 2H). ^13^C NMR (150 MHz, DMSO-*d*_6_): δ 169.79, 167.02, 162.52, 159.75, 153.64, 152.34, 141.04, 126.97, 126.43, 125.95, 113.68, 110.03, 104.70, 63.57, 40.52, 35.10, 31.84, 31.51, 31.42 (3C), 11.90, 9.18, 9.00. HRMS (ES+) *m*/*z* calcd for C_24_H_25_N_2_O_4_S^+^ [M + H]^+^: 437.1530; found, 437.1533.

##### (±) 8-Cyclopropyl-7-(3-(3,5-di-*tert*-butylphenyl)isoxazol-5-yl)-5-oxo-2,3-dihydro-5*H*-thiazolo[3,2-*a*]pyridine-3-carboxylic Acid (**17k**)

The product was obtained following the two consecutive steps of general synthesis of isoxazole and ester hydrolysis as a white solid (55 mg, 0.11 mmol, 29%). ^1^H NMR (600 MHz, DMSO-*d*_6_): δ 7.75 (s, 2H), 7.66 (s, 1H), 7.56 (t, *J* = 1.8 Hz, 1H), 6.51 (s, 1H), 5.54 (dd, *J* = 9.2, 1.8 Hz, 1H), 3.91 (dd, *J* = 11.9, 9.2 Hz, 1H), 3.62 (dd, *J* = 11.9, 1.8 Hz, 1H), 1.97–1.93 (m, 1H), 1.36 (s, 18H), 0.87–0.84 (m, 2H), 0.40–0.30 (m, 2H). ^13^C NMR (151 MHz, DMSO-*d*_6_): δ 169.80, 167.31, 163.28, 159.78, 152.33, 151.78 (2C), 141.19, 128.11, 124.56, 121.31 (2C), 113.86, 110.06, 104.73, 63.55, 35.19 (2C), 31.83, 31.62 (6C), 11.92, 9.05, 8.89. HRMS (ES+) *m*/*z* calcd for C_28_H_33_N_2_O_4_S^+^ [M + H]^+^: 493.2156; found, 493.2166.

##### (±) 8-Cyclopropyl-7-(3-(3,5-difluorophenyl)isoxazol-5-yl)-5-oxo-2,3-dihydro-5*H*-thiazolo[3,2-*a*]pyridine-3-carboxylic Acid (**17l**)

The product was obtained following the two consecutive steps of general synthesis of isoxazole and ester hydrolysis as a white solid (48 mg, 0.12 mmol, 40%). ^1^H NMR (600 MHz, DMSO-*d*_6_): δ 13.59 (s, 1H), 7.74–7.71 (m, 3H), 7.51–7.47 (m, 1H), 6.49 (s, 1H), 5.54 (dd, *J* = 9.2, 1.7 Hz, 1H), 3.90 (dd, *J* = 11.9, 9.2 Hz, 1H), 3.62 (dd, *J* = 11.9, 1.8 Hz, 1H), 1.93–1.88 (s, 1H), 0.90–0.88 (m, 2H), 0.39–0.31 (m, 2H). ^13^C NMR (150 MHz, DMSO-*d*_6_): δ 169.77, 167.78, 164.13 (d, *J* = 13.5 Hz), 162.50 (d, *J* = 13.5 Hz), 161.11, 159.69 (2C), 152.52, 140.67, 131.92 (d, *J* = 10.6 Hz), 113.76, 110.65–110.47 (m), 109.93, 106.42 (t, *J* = 25.8 Hz), 105.03, 63.57, 31.85, 11.85, 9.20, 9.02. ^19^F NMR (565 MHz, DMSO-*d*_6_): δ −108.32, −108.34, −108.35. HRMS (ES+) *m*/*z* calcd for C_20_H_15_F_2_N_2_O_4_S^+^ [M + H]^+^: 417.0715; found, 417.0723.

##### (±) 8-Cyclopropyl-7-(3-(4-fluoronaphthalen-1-yl)isoxazol-5-yl)-5-oxo-2,3-dihydro-5*H*-thiazolo[3,2-*a*]pyridine-3-carboxylic Acid (**17m**)

The product was obtained following the two consecutive steps of general synthesis of isoxazole and ester hydrolysis as a white solid (238 mg, 0.53 mmol, 67%). ^1^H NMR (600 MHz, DMSO-*d*_6_): δ 13.59 (s, 1H), 8.57 (dd, *J* = 7.7, 1.8 Hz, 1H), 8.21 (dd, *J* = 7.7, 1.8 Hz, 1H), 7.94 (dd, *J* = 8.0, 5.4 Hz, 1H), 7.81–7.75 (m, 2H), 7.57–7.53 (m, 2H), 6.58 (s, 1H), 5.56 (dd, *J* = 9.2, 1.7 Hz, 1H), 3.91 (dd, *J* = 11.9, 9.2 Hz, 1H), 3.63 (dd, *J* = 11.9, 1.7 Hz, 1H), 1.98–1.95 (m, 1H), 0.92–0.88 (m, 2H), 0.46–0.37 (m, 2H). ^13^C NMR (151 MHz, DMSO-*d*_6_): δ 167.68, 164.48, 160.26, 158.19, 157.64, 156.51, 150.30, 138.81, 130.00 (d, *J* = 4.9 Hz), 127.17 (d, *J* = 9.3 Hz), 127.04, 125.70, 124.02 (d, *J* = 2.3 Hz), 121.51 (d, *J* = 16.4 Hz), 120.67 (d, *J* = 4.3 Hz), 118.87 (d, *J* = 5.7 Hz), 111.76, 107.99, 105.66, 61.46, 29.73, 9.78, 7.07, 6.88. ^19^F NMR (565 MHz, DMSO-*d*_6_): δ −119.57 (dd, *J* = 10.6, 5.4 Hz). HRMS (ES+) *m*/*z* calcd for C_24_H_18_FN_2_O_4_S^+^ [M + H]^+^: 449.0966; found, 449.0973.

##### (±) 8-Cyclopropyl-5-oxo-7-(3-(thiophen-2-yl)isoxazol-5-yl)-2,3-dihydro-5*H*-thiazolo[3,2-*a*]pyridine-3-carboxylic Acid (**17n**)

The product was obtained following the two consecutive steps of general synthesis of isoxazole and ester hydrolysis as a pale yellow solid (48 mg, 0.12 mmol, 49%). ^1^H NMR (600 MHz, DMSO-*d*_6_): δ 13.58 (s, 1H), 7.80–7.78 (m, 2H), 7.55 (s, 1H), 7.26 (dd, *J* = 5.0, 3.6 Hz, 1H), 6.48 (s, 1H), 5.53 (dd, *J* = 9.2, 1.8 Hz, 1H), 3.90 (dd, *J* = 11.9, 9.2 Hz, 1H), 3.61 (dd, *J* = 11.9, 1.8 Hz, 1H), 1.92–1.87 (m, 1H), 0.90–0.83 (m, 2H), 0.40–0.30 (m, 2H). ^13^C NMR (150 MHz, DMSO-*d*_6_): δ 169.76, 167.14, 159.71, 158.20, 152.42, 140.79, 129.96, 129.85, 129.52, 128.59, 113.85, 109.99, 104.50, 63.57, 31.84, 11.87, 9.13, 8.95. HRMS (ES+) *m*/*z* calcd for C_18_H_15_N_2_O_4_S_2_^+^ [M + H]^+^: 387.0468; found, 387.0477.

##### (±) 7-(3-(Benzo[*d*][1,3]dioxol-5-yl)isoxazol-5-yl)-8-cyclopropyl-5-oxo-2,3-dihydro-5*H*-thiazolo[3,2-*a*]pyridine-3-carboxylic Acid (**17o**)

The product was obtained following the two consecutive steps of general synthesis of isoxazole and ester hydrolysis as a white solid (53 mg, 0.13 mmol, 69%). ^1^H NMR (600 MHz, DMSO-*d*_6_): δ 13.57 (s, 1H), 7.54 (s, 1H), 7.50 (dd, *J* = 5.9, 1.6 Hz, 2H), 7.10 (d, *J* = 8.3 Hz, 1H), 6.47 (s, 1H), 6.13 (s, 2H), 5.53 (dd, *J* = 9.1, 1.5 Hz, 1H), 3.89 (dd, *J* = 11.8, 9.2 Hz, 1H), 3.61 (dd, *J* = 11.8, 1.8 Hz, 1H), 1.94–1.89 (m, 1H), 0.90–0.84 (m, 2H), 0.39–0.30 (m, 2H). ^13^C NMR (150 MHz, DMSO-*d*_6_) 169.78, 166.93, 162.31, 159.75, 152.32, 149.52, 148.50, 141.02, 122.52, 121.71, 113.62, 110.01, 109.37, 107.06, 104.66, 102.12, 63.56, 31.83, 11.90, 9.19, 9.03. HRMS (ES+) *m*/*z* calcd for C_21_H_17_N_2_O_6_S^+^ [M + H]^+^: 425.0802; found, 425.0787.

##### (±) 8-Cyclopropyl-5-oxo-7-(3-(quinolin-4-yl)isoxazol-5-yl)-2,3-dihydro-5*H*-thiazolo[3,2-*a*]pyridine-3-carboxylic Acid (**17p**)

The product was obtained following the two consecutive steps of general synthesis of isoxazole and ester hydrolysis as a white solid (35 mg, 0.08 mmol, 28%). ^1^H NMR (600 MHz, DMSO-*d*_6_): δ 9.10 (d, *J* = 4.4 Hz, 1H), 8.59 (dd, *J* = 8.5, 1.3 Hz, 1H), 8.19 (dd, *J* = 8.5, 1.3 Hz, 1H), 7.93 (d, *J* = 4.4 Hz, 1H), 7.90 (ddd, *J* = 8.3, 6.8, 1.4 Hz, 1H), 7.78 (ddd, *J* = 8.3, 6.8, 1.3 Hz, 1H), 7.71 (s, 1H), 6.60 (s, 1H), 5.56 (dd, *J* = 9.2, 1.8 Hz, 1H), 3.92 (dd, *J* = 11.9, 9.2 Hz, 1H), 3.63 (dd, *J* = 11.9, 1.8 Hz, 1H), 1.99–1.94 (m, 1H), 0.92–0.87 (m, 2H), 0.47–0.36 (m, 2H). ^13^C NMR (151 MHz, DMSO-*d*_6_): δ 169.77, 167.28, 161.38, 159.73, 152.55, 150.88, 148.75, 140.69, 134.09, 130.56, 130.26, 128.52, 126.16, 124.99, 122.47, 114.06, 110.07, 107.54, 63.62, 31.89, 11.88, 9.17, 8.97. HRMS (ES+) *m*/*z* calcd for C_23_H_18_N_3_O_4_S^+^ [M + H]^+^: 432.0940; found, 432.0991.

##### (±) Methyl 8-Cyclopropyl-7-(5-(naphthalen-1-yl)isoxazol-3-yl)-5-oxo-2,3-dihydro-5*H*-thiazolo[3,2-*a*]pyridine-3-carboxylate (**18a**)

The product was obtained following the general synthesis of isoxazole from compound **4** as a white solid (42 mg, 0.09 mmol, 37%). ^1^H NMR (600 MHz, chloroform-*d*): δ 8.25 (d, *J* = 8.3 Hz, 1H), 7.93 (d, *J* = 8.2 Hz, 1H), 7.88 (d, *J* = 7.9 Hz, 1H), 7.84–7.74 (m, 1H), 7.61–7.46 (m, 3H), 6.73 (s, 1H), 6.55 (s, 1H), 5.62 (dd, *J* = 8.7, 2.1 Hz, 1H), 3.78 (s, 3H), 3.69 (dd, *J* = 11.7, 8.9 Hz, 1H), 3.52 (dd, *J* = 11.7, 2.2 Hz, 1H), 1.78–1.74 (m, 1H), 0.79–0.75 (m, 2H), 0.41 (d, *J* = 5.3 Hz, 2H). ^13^C NMR (151 MHz, chloroform-*d*): δ 169.98, 168.37, 160.89, 160.65, 150.03, 144.44, 133.82, 131.16, 130.28, 128.80, 127.84, 127.58, 126.54, 125.18, 124.79, 124.75, 116.12, 112.50, 104.30, 63.30, 53.42, 31.80, 11.93, 8.72, 8.64. LCMS (ES+) *m*/*z* calcd for C_25_H_21_N_2_O_4_S^+^ [M + H]^+^: 445.1; found, 445.7.

##### (±) Methyl 8-Cyclopropyl-5-oxo-7-(5-(3-(trifluoromethyl)phenyl)isoxazol-3-yl)-2,3-dihydro-5*H*-thiazolo[3,2-*a*]pyridine-3-carboxylate (**18b**)

The product was obtained following the general synthesis of isoxazole from compound **4** as a white solid (45 mg, 0.09 mmol, 41%). ^1^H NMR (600 MHz, chloroform-*d*): δ 8.10 (s, 1H), 8.04 (d, *J* = 7.8 Hz, 1H), 7.76 (d, *J* = 7.8 Hz, 1H), 7.67 (t, *J* = 7.8 Hz, 1H), 6.81 (s, 1H), 6.54 (s, 1H), 5.69 (dd, *J* = 8.7, 2.3 Hz, 1H), 3.86 (s, 3H), 3.77 (dd, *J* = 11.8, 8.7 Hz, 1H), 3.60 (dd, *J* = 11.9, 1.9 Hz, 1H), 1.82–1.79 (m, 1H), 0.83–0.79 (m, 2H), 0.45–0.41 (m, 2H). ^13^C NMR (151 MHz, chloroform-*d*): δ 168.31, 161.40, 160.51, 150.14, 144.00, 146.41, 131.76 (q, *J* = 33.0 Hz), 129.80, 128.99, 127.82, 126.98 (q, *J* = 3.6 Hz), 123.62 (q, *J* = 273.3 Hz), 122.74 (q, *J* = 3.8 Hz), 116.12, 112.30, 101.10, 63.27, 53.42, 31.77, 11.81, 8.66, 8.57. ^19^F NMR (565 MHz, chloroform-*d*): δ −62.95. LCMS (ES+) *m*/*z* calcd for C_22_H_18_F_3_N_2_O_4_S^+^ [M + H]^+^: 463.08; found, 464.1.

##### (±) 8-Cyclopropyl-7-(5-(naphthalen-1-yl)isoxazol-3-yl)-5-oxo-2,3-dihydro-5*H*-thiazolo[3,2-*a*]pyridine-3-carboxylic Acid (**19a**)

The product was obtained following the general ester hydrolysis as a white solid (19 mg, 0.04 mmol, 47%, 17% yield over two consecutive steps). ^1^H NMR (600 MHz, methanol-*d*_4_): δ 8.34 (d, *J* = 8.5 Hz, 1H), 8.10 (d, *J* = 8.2 Hz, 1H), 8.04 (d, *J* = 8.1 Hz, 1H), 7.94 (d, *J* = 7.1 Hz, 1H), 7.69–7.62 (m, 3H), 7.19 (d, *J* = 1.7 Hz, 1H), 6.57 (d, *J* = 1.7 Hz, 1H), 5.70 (d, *J* = 8.8 Hz, 1H), 3.94 (dd, *J* = 11.7, 1.2 Hz, 1H), 3.72 (d, *J* = 11.7 Hz, 1H), 1.96–1.92 (m, 1H), 0.91–0.86 (q, *J* = 9.0, 8.4 Hz, 2H), 0.52–0.49 (m, 2H). ^13^C NMR (150 MHz, DMSO-*d*_6_): δ 169.60, 169.46, 159.63, 156.30, 149.59, 142.08, 133.89, 131.75, 129.97, 129.35, 128.48, 128.45, 127.19, 125.99, 124.78, 124.44, 119.45, 111.76, 105.86, 64.67, 32.26, 12.51, 8.16, 8.05. HRMS (ES+) *m*/*z* calcd for C_24_H_19_N_2_O_4_S^+^ [M + H]^+^: 431.1060; found, 431.1071.

##### (±) 8-Cyclopropyl-5-oxo-7-(5-(3-(trifluoromethyl)phenyl)isoxazol-3-yl)-2,3-dihydro-5*H*-thiazolo[3,2-*a*]pyridine-3-carboxylic Acid (**19b**)

The product was obtained following the general ester hydrolysis as a white solid (35 mg, 0.08 mmol, 90%, 33% yield over two consecutive steps). ^1^H NMR (600 MHz, methanol-*d*_4_): δ 8.23–8.20 (m, 2H), 7.84 (d, *J* = 7.9 Hz, 1H), 7.78 (t, *J* = 7.8 Hz, 1H), 7.36 (s, 1H), 6.50 (s, 1H), 5.72 (d, *J* = 8.9 Hz, 1H), 3.95 (dd, *J* = 11.8, 9.1 Hz, 1H), 3.71 (d, *J* = 11.9 Hz, 1H), 1.92–1.88 (m, 1H), 0.84–0.81 (m, 2H), 0.43–0.41 (m, 2H). ^13^C NMR (151 MHz, methanol-*d*_4_): δ 169.39, 168.28, 161.67, 161.43, 152.10, 144.67, 131.33 (q, *J* = 32.6 Hz), 129.98, 129.05, 127.99, 126.65 (q, *J* = 3.5 Hz), 123.85 (q, *J* = 271.7 Hz), 122.11 (q, *J* = 3.7 Hz), 114.10, 113.14, 101.96, 63.82, 31.43, 11.20, 7.96, 7.91. ^19^F NMR (565 MHz, methanol-*d*_4_): δ −64.38. HRMS (ES+) *m*/*z* calcd for C_21_H_16_F_3_N_2_O_4_S^+^ [M + H]^+^: 449.0777; found, 449.0792.

### Bacterial Strains and Growth Conditions

*Mtb* Erdman was thawed from a freezer stock and inoculated into Middlebrook 7H9 liquid medium supplemented with 1.7 g/L NaCl, 10 g/L BSA, 4 g/L dextrose, 0.006 g/L catalase (ADC), 0.5% glycerol, and 0.05% Tween-80. *Mtb* growing logarithmically was then inoculated into Sauton liquid medium [0.5 g/L KH_2_PO_4_, 0.5 g MgSO_4_, 4.0 g/L l-asparagine, 6% glycerol, 0.05 g/L ferric ammonium citrate, 2.0 g/L citric acid, and 0.01% (wt/vol) ZnSO_4_; adjusted to a pH of 7; supplemented with 0.05% Tween-80] and used for experiments. Viable CFUs were enumerated on Middlebrook 7H11 agar medium supplemented with oleic acid, ADC, and 0.5% glycerol. The *katG* frameshift mutant of *Mtb* was isolated as described previously.^16^

### Microplate Alamar Blue Assay

Logarithmically growing *Mtb* was inoculated into Tween-free Sauton’s medium in 96-well plates with wells containing decreasing doses of each compound of interest. **C10** with imidazole was included as a performance reference in all biological assays. Initial MABAs were performed using a nine-point dose curve ranging from 50 to 0.195 μM. Potent compounds (e.g., **17h** and **17j**) were retested at lower doses to achieve a complete dose–response curve and IC_50_ values. After one week of incubation at 37 °C with 5% CO_2_, 0.052 mM resazurin and 1.1% Tween-80 were added to each well, and the plate was incubated for an additional 24 h at 37 °C with 5% CO_2_. Conversion of resazurin to fluorescent resorufin was measured on a BioTek H1 Synergy Plate reader with λ_ex_ = 530 nm and λ_em_ = 590 nm. The fluorescent measurements (RFU) for the wells containing bacteria and IMD/DMSO were set to 0% inhibition. The fluorescent measurements for the wells containing IMD/DMSO with no bacteria were set to 100% inhibition. The percent inhibition for each compound was calculated relative to the mean fluorescence of untreated *Mtb* wells using the following formula
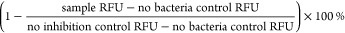


IC_50_ values were calculated as described in the Statistical Analysis section of the Experimental Section.

### Permeability and Efflux in Caco-2 Cells

The Caco-2 study was performed according to a published protocol.^26^ Caco-2 cell monolayers were grown on permeable filter support and used for transport study on day 21 after seeding. Prior to the experiment, a drug solution of 2 μM was prepared and warmed to 37 °C. The Caco-2 filters were washed with prewarmed HBSS (Hanks’ Balanced Salt Solution) prior to the experiment, and thereafter, the experiment was started by applying the donor solution on the apical or basolateral side. The transport experiments were carried out at pH 7.4 in both the apical and basolateral chambers. 5 μM enalaprilat was used as membrane integrity control in each filter. The Papp of enalaprilat for a tight monolayer has been determined to be <1 × 10^–6^ cm/s. The experiments were performed at 37 °C and with a stirring rate of 500 rpm. The receiver compartment was sampled at 30 min, and at 30 min also, a final sample from the donor chamber was taken in order to calculate the mass balance of the compound. The samples (100 μL) were transferred to a 96-well plate containing 100 μL of methanol and warfarin as the internal standard and were sealed until LC–MS/MS analysis.

### CYP Inhibition

The inhibition assay utilized human liver microsomes (HLM) at a final concentration of 0.2 mg/mL in 100 mM phosphate buffer (pH 7.4). CYP marker substrate and concentrations were as follows: CYP3A4: midazolam 5 μM and testosterone 50 μM; CYP2C9: diclofenac 5 μM. The incubation time was 30 min for all except midazolam (10 min). The representative compounds were added to a 96-well plate (1 μL) to achieve final concentrations of 1, 10, and 100 μM from 100× stocks prepared in DMSO. Control wells contained only DMSO (1% final concentration). The incubation volume was 100 μL. The reaction was started by addition of 50 μL 2 mM NADPH (in buffer) and stopped by addition of 100 μL ice-cold acetonitrile containing warfarin/ BCS as the internal standard. After the assay, the plate was sealed and centrifuged, and analyzed by LC–MS/MS (Waters TQs Micro coupled to an Acquity UPLC). Chromatographic separation was performed using formic acid and acetonitrile-based mobile phases on a BEH C18 from Waters.

### Inhibition of Biofilm Pellicle Formation

Biofilm pellicle formation assays were performed as described previously.^16^ For the initial screening assay, compounds were tested at the concentrations of 50, 25, 10, 5, and 2.5 μM for inhibition of biofilm pellicle formation, which was evaluated by eye following 3 weeks of incubation under low oxygen and 2 weeks of incubation under aerated conditions. For the assays involving the determination of the IC_50_ for biofilm inhibition by **C10**, **17h**, and **17j**, Sauton’s medium was inoculated with stationary-phase *Mtb* with and without two-fold dilutions of **C10** ranging from 0.39–100 to 0.08–20 μM for the more potent compounds, **17h** and **17j**; untreated controls were included in every row of the assay. The plates were sealed in airtight containers and incubated at 37 °C with 5% CO_2_. After 3 weeks, the lid of the container was removed, and the plates were incubated for an additional 3 weeks. At the end of the 6-week treatment, the plates were photographed and the OD_600_ through the pellicle at the air/liquid interface in each well was quantified using a BioTek Synergy H1 Plate reader to determine pellicle density. In control wells containing tyloxapol, the procedure was the same but 0.05% tyloxapol was added to each well.

### Aerobic Liquid Media Growth Assays

Sauton’s liquid medium was inoculated with the *Mtb katG*^*FS*^ mutant at an OD_600_ of 0.1. **C10**, **17h**, and/or **17j** were added at a 1 μM concentration, and INH was added at 0.25 μg/mL. IMD and DMSO were used as a vehicle control in otherwise untreated bottles and in INH-only treatments. Bottles were incubated, rolling, at 37 °C, and OD_600_ was monitored over time. Viable CFUs were enumerated after the designated time by plating serial dilutions of the cultures on 7H11 agar medium plus ADC. This was performed in duplicate in each of the two experiments.

### Cell-Based Cytotoxicity Assays

Calu-3 cells (ATCC-HTB55), immortalized human lung epithelial cells, were cultured and maintained in Dulbecco’s Modified Eagle Medium (DMEM) with 10% fetal bovine serum (FBS), 1% HEPES, and 1% penicillin/streptomycin at 37 °C with 5% CO_2_. To assess compound toxicity to eukaryotic cells, 10,000 Calu-3 cells/well were plated on a white-walled 96-well plate and incubated at 37 °C with 5% CO_2_ for 18 h. Media were then replaced with the same media mixture but with 2% FBS instead of 10% FBS. Compounds were then added by performing two-fold serial dilutions over eight doses ranging from 250 μM down to 0.977 μM. After a 72 h incubation, plates were examined visually on a microscope and 25 μL of CellTiter-Glo (Promega) was added per well to measure cellular ATP concentrations via luminescence using a BioTek Synergy H1 Plate reader with a gain setting of 170, as a proxy for cell viability. Percent viability was calculated as luminescence relative to untreated cells. To further assess the potential cytotoxicity of the compounds against human embryonic kidney (HEK 293) cells, human non-tumorigenic lung epithelial cells (BEAS-2B), and normal human lung fibroblasts (IMR-90), the 3-(4,5-dimethylthiazol-2-yl)-2,5-diphenyltetrazolium bromide (MTT) test was used.^27^ Compounds **C10**, **17h**, and **17j** were added in twofold serial dilutions ranging from 250 to 1.97 μM to a sterile 96-well microtiter plate containing 1 × 10^4^ cells/well and cultured for 48 h at 37 °C with 5% CO_2_. The medium was then removed, and 10 μL of MTT reagent (5 mg/mL) was added to the plate and incubated for another 3 h. The MTT reagent was then removed, and 100 μL of DMSO was added to each well. DMSO dissolves the formazan crystals that have formed in the wells. The absorbance was measured at 580 nm against a blank using the Thermo Scientific Varioskan Lux microplate reader, and the LD_50_ values were calculated using GraphPad Prism. Cytotoxicity experiments were performed in triplicate.

### Macrophage Infection and Quantification of Intracellular Mycobacterial Replication

Macrophage infection and quantification of intracellular mycobacterial replication were performed using *Mtb* H37Rv strain (*Mtb*, ATCC-27294) constitutively expressing the green fluorescent protein (H37Rv-GFP) as a reporter for the replication assay.^28^ The bacteria were cultured at 37 °C in complete 7H9 medium containing 0.5% glycerol (50405, Euromedex), 10% Middlebrook oleic acid-albumin-dextrose-catalase (OADC, 211886, Becton Dickinson), 0.05% Tween 80 (P4780, Sigma-Aldrich), and 50 μg/mL hygromycin B (10687010, Invitrogen). On the day of the experiment, *Mtb* was washed three times with D-PBS and resuspended in RPMI-1640 + GlutaMAX (61870044, Life Technologies). Mouse macrophage RAW 264.7 cells (ATCC # TIB-71) were maintained in RPMI-1640 + GlutaMAX containing 10% heat-inactivated fetal bovine serum (FBS, 10270106, Gibco). Macrophages were harvested using Versene (15040033, LifeTechnologies).

For assay plate preparation, the lead compounds were diluted in DMSO (Sigma-Aldrich) to 10 mg/mL and dispensed in Echo-qualified 384-well low dead volume source plates using the Echo 550 Series Liquid Handler (Labcyte). The compounds were transferred to 384-well clear-bottom polystyrene assay plates (781091, Greiner Bio-One) using acoustic liquid handling technology. All samples were backfilled with DMSO.

For the intracellular assay, bacteria were mixed with RAW 264.7 macrophages to prepare a suspension, and the infected cells were incubated for 3 h at 37 °C with shaking. After infection, the cells were washed by centrifugation at 1400 rpm for 5 min to remove extracellular bacteria. The cellular suspension (50 μL containing 20,000 cells) was added to the 384-well assay plate and incubated for 5 days at 37 °C, 5% CO_2_. Macrophages were then stained with SYTO 60 dye (S11342, Molecular probes), followed by image acquisition and data analysis. Confocal images were recorded on an automated high-content fluorescent microscope (InCell 6500, Cytiva). A series of four pictures of each well were taken, and each image was then analyzed using a Columbus system as previously described^29^ to infer the percentage of infected cells and the intracellular bacterial area. Dose–response plots were generated for each compound using GraphPad Prism software to determine the IC_50_.

### Cytotoxicity Assay on Murine Macrophages

Mouse macrophage RAW 264.7 cells (ATCC # TIB-71) were maintained in RPMI-1640 + GlutaMAX containing 10% heat-inactivated fetal bovine serum (FBS, 10270106, Gibco). Macrophages were harvested using Versene (15040033, LifeTechnologies).

For assay plate preparation, the lead compounds were diluted in DMSO (Sigma-Aldrich) to 10 mg/mL and dispensed in Echo-qualified 384-well low dead volume source plates using the Echo 550 Series Liquid Handler (Labcyte). The compounds were transferred to 384-well clear-bottom polystyrene assay plates (781091, Greiner Bio-One) using acoustic liquid handling technology. All samples were backfilled with DMSO.

The cellular suspension (50 μL containing 20,000 cells) was added to the 384-well assay plate and incubated for 5 days at 37 °C, 5% CO_2_. Macrophages were then stained with SYTO 60 dye (S11342, Molecular probes), followed by image acquisition and data analysis. Confocal images were recorded on an automated high-content fluorescence microscope (InCell 6500, Cytiva). A series of four pictures of each well were taken, and each image was then analyzed using the Columbus system to infer the total number of cells. Dose–response plots were generated for each compound using GraphPad Prism software.

### Statistical Analysis

#### MABAs and Biofilm Inhibition

IC_50_ values were calculated by GraphPad Prism using a nonlinear regression, with an [inhibitor] vs response – variable slope equation, fit to each dose curve. Average IC_50_ and SD values were calculated for either two or three MABA replicates for each compound (as defined in Tables 1–4). Replicate values were compared to **C10** with an ordinary one-way ANOVA with Fisher’s LSD Test in Prism, which produced the *p* values listed in Tables 1–4. For consistency, we used a maximum IC_50_ cutoff of 25 μM in Tables 1–4. However, in many of our MABAs for less potent compounds, we tested concentrations higher than 25 μM. For those compounds, we used the calculated IC_50_ values in our ANOVA tests. For a given replicate of a compound, if the highest dose tested achieved <50% inhibition and no IC_50_ was calculated, we assigned the highest dose tested as the IC_50_ for the purpose of performing statistical analysis. For example, if the highest concentration tested in a MABA was 100 μM, and the IC_50_ was higher than that, we assigned 100 μM as the IC_50_ value. Thus, for compounds with IC_50_ values >25 μM, we used the most conservative IC_50_ estimate possible to perform statistical testing. Biofilm IC_50_ values were calculated based on OD_600_ using the same method described for MABA IC_50_.

#### *Mtb katG*^*FS*^ Inhibition and INH Potentiation

Each OD_600_ over the course of the 13 day experiment was graphed and fit by Prism with a nonlinear regression using an exponential growth equation to calculate doubling time as a best-fit value. Doubling times for each treatment as well as day 13 CFU/mL concentrations for each treatment were compared using an ordinary one-way ANOVA with Tukey’s correction for multiple comparisons.

## Supporting Information Available

Synthesis of Bestmann–Ohira reagent, acyl hydrazides (**5a–l**), 5-substituted-1,3,4-oxadiazole-2-thiols (**6a–l**), oxadiazole intermediates (**7a–l**) data, azide intermediates (**IIa-b**), oximes (**Ia-p**), structure and biofilm inhibition activity of ring-fused thiazolo-2-pyridone library with a variation of substituents, permeability and CYP inhibition of selected compounds, inhibition of **17h** and **17j** growth and survival in the presence of tyloxapol, cytotoxicity of **C10**, **17h**, and **17j** in murine macrophages, separation of enantiomers of **17j**, separation of racemic **17j**, chromatogram of racemic **17j**, chromatogram of **(+)17j**, chromatogram of **(−)17j**, and NMR-analyzed and peak-assigned spectra of synthesized compounds (^1^H, ^13^C, and ^19^F NMR) (PDF)
Molecular formula strings and assay data (CSV)

## Abbreviations

CFUs: colony-forming units
dd: double doublet
DMEM: Dulbecco’s Modified Eagle Medium
DR: drug-resistant
FBS: fetal bovine serum
HBSS: Hanks’ balanced salt solution
HLM: human liver microsomes
IMD: imidazole salt
INH: isoniazid
MABA: microplate Alamar blue assay
Mtb: *Mycobacterium tuberculosis*
MTT: 3-(4,5-dimethylthiazol-2-yl)-2,5-diphenyltetrazolium bromide
Papp: apparent permeability
RIF: rifampicin
SAR: structure–activity relationship
XDR: extensively drug-resistant
