# Supplementary material for: Design, Synthesis, and Evaluation of Novel Δ2-Thiazolino 2-Pyridone Derivatives That Potentiate Isoniazid Activity in an Isoniazid-Resistant Mycobacterium tuberculosis Mutant
Source: J Med Chem. 2023 Jul 24;66(16):11056–77. doi: 10.1021/acs.jmedchem.3c00358 (PMC10461229; doi:10.1021/acs.jmedchem.3c00358)

Supporting information

Design, synthesis, and evaluation of novel Δ^2-thiazolino 2-pyridone derivatives that potentiate isoniazid activity in an isoniazid-resistant *Mycobacterium tuberculosis* mutant

Souvik Sarkar[†#], Anne E. Mayer Bridwell[‡#], James A. D. Good[†], Erin R. Wang[‡], Samuel R. McKee[‡], Joy Valenta[‡], Gregory A. Harrison[‡], Kelly N. Flentie[‡], Frederick L. Henry[‡], Torbjörn Wixe[†], Peter Demirel[†], Siva K. Vagolu[§], Jonathan Chatagnon[^], Arnaud Machelart[^], Priscille Brodin[^], Tone Tønjum[§¶], Christina L. Stallings[‡*], Fredrik Almqvist[†*]

[†] Department of Chemistry, Umeå University, SE-90187 Umeå, Sweden

[‡] Department of Molecular Microbiology, Center for Women's Infectious Disease Research, Washington University School of Medicine, St. Louis, MO 63110, USA

[§] Department of Microbiology, University of Oslo, N-0316 Oslo, Norway

^ Univ. Lille, CNRS, INSERM, CHU Lille, Institut Pasteur de Lille, U1019-UMR 9017-CIIL-Center for Infection and Immunity of Lille, Lille, France.

[¶] Oslo University Hospital, N-0424 Oslo, Norway

[#]These authors contributed equally

*Correspondence to fredrik.almqvist@umu.se, stallings@wustl.edu

Table of Contents

1. Synthesis………………………………………………………………………………. S2
2. Structure and Biofilm inhibition activity of thiazolo ring-fused 2-pyridone library.... S9
3. Permeability and CYP inhibition of selected compounds……………………………. S11
4. Inhibition of **17h** and **17j** growth and survival in the presence of tyloxapol ……….. S12
5. Cytotoxicity of **C10**, **17h** and **17j** in murine macrophages…………………………… S12
6. Separation of enantiomers ……………………………………………………………….. S13
7. NMR data……………………………………………………………………………… S14

1. **Synthesis**

Scheme S1. **Synthesis of Bestmann-Ohira reagent.**

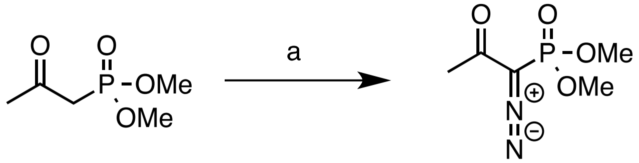

Reagents and conditions: (a) (i) NaH, toluene, 0 °C; (ii) 4-acetamidobenzenesulfonyl azide in THF, 16 h, rt.

A flame dried two-necked round bottom flask was cooled down under nitrogen atmosphere. The flask was charged with Dimethyl acetylmethylphosphonate (48.0 mmol, 1.0 equiv) and dissolved in toluene (120 mL) under N_2 atmosphere. The solution was cooled to 0 °C and NaH (60% in paraffin, 48.0 mmol, 1.0 equiv) was added over 10 min in portions when gas evolution started and reaction mixture become highly viscous suspension. After the gas evolution had ceased, a solution of 4-acetamidobenzenesulfonyl azide (48.0 mmol, 1.0 equiv) in THF (40 mL) was added dropwise; the highly viscous suspension slowly discolored to yellow-brown and stirring became easier. After 16 h the mixture was diluted with petroleum ether, filtered through a pad of Celite, rinsed thoroughly with Et_2O. The organic solvent layers were collected and solvents were removed under reduced pressure. The product obtained as crude pale-yellow oil 8.67 g (48.0 mmol, 94%). The crude oil was used for the next reaction without further purification.

General Procedure for the Synthesis of Acyl Hydrazides (5a-l): To a solution of the corresponding carboxylic acid derivative (2.0 mmol, 1.0 equiv) in 10 mL of ethanol was added concentrated H_2SO_4 (0.2 mmol, 0.1 equiv) and the mixture was refluxed until full consumption of starting material. Reaction was monitored by TLC. When full conversion of starting material was observed by TLC, 1 mL of hydrazine monohydrate (20 mmol, 10 equiv) was added to the same reaction mixture. The reaction mixture was maintained under reflux for 3 h, when TLC indicated the total consumption of the ester intermediate. The media was poured onto ice and the resulting precipitate was filtered out, affording the corresponding acyl hydrazide derivatives. Acyl hydrazides (**5a and 5c-e**) were used in further reactions without purification. Acyl hydrazides (**5b and 5f-l**) were obtained from commercial sources.

Scheme S2. Synthesis of oxadiazole intermediates 6a-l.

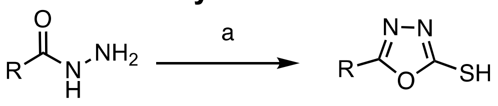

5a-l 6a-l

R = naphthalen-1-yl (6a)
= 3-(trifluoromethyl)phenyl (6b)
= naphthalen-1-ylmethyl (6c)
= naphthalen-2-yl (6d)
= naphthalen-2-ylmethyl (6e)
= 4-(*tert*-butyl)phenyl (6f)
= 4-nitrophenyl (6g)
= 3-methoxyphenyl (6h)
= pyridin-4-yl (6i)
= thiophen-2-yl (6j)
= furan-2-yl (6k)
= (4-fluorophenoxy)methyl (6l)

Reagents and conditions: (a) CS_2, KOH, EtOH, 16 h, reflux.

General Procedure for the Synthesis of 5-Substituted-1,3,4- oxadiazole-2-thiols (6a−l): Carbon disulfide (6.0 mmol, 3.0 equiv) was slowly added to a solution of acyl hydrazide (**5a-l**) (2.0 mmol, 1.0 equiv) and potassium hydroxide (2.0 mmol, 1.0 equiv) in EtOH (15 mL). The reaction mixture was refluxed for 6-18 h, depending on the substituent. Reaction was monitored by TLC. Upon completion, the solvent was removed under vacuo and the remaining solid was dissolved in water. The aqueous solution was acidified to pH 2 using 1M HCl solution to obtain solid precipitate. The product was filtered and washed with water to obtain a neutral pH. Substituted-1,3,4-oxadiazole-2-thiols (**6a-l**) were obtained with sufficient purity and used for further reactions without purification.

(±) Methyl 8-cyclopropyl-7-(((5-(naphthalen-1-yl)-1,3,4-oxadiazol-2-yl)thio)methyl)-5-oxo-2,3-dihydro-5H-thiazolo[3,2-a]pyridine-3-carboxylate (7a)

Prepared by following the general procedure. Final compound obtained as white solid 125 mg (0.254 mmol, 76%). **^{1}H NMR** (400 MHz, Chloroform-*d*) δ 9.10 (dd, *J* = 8.6, 1.1 Hz, 1H), 8.04 (dd, *J* = 7.3, 1.2 Hz, 1H), 7.95 (d, *J* = 8.3 Hz, 1H), 7.86 – 7.83 (m, 1H), 7.62 – 7.58 (m, 1H), 7.53 – 7.47 (m, 2H), 6.46 (s, 1H), 5.51 (dd, *J* = 8.6, 2.3 Hz, 1H), 4.61 – 4.45 (m, 2H), 3.71 (s, 3H), 3.59 (dd, *J* = 11.8, 8.6 Hz, 1H), 3.44 (dd, *J* = 11.8, 2.3 Hz, 1H), 1.73 – 1.66 (m, 1H), 0.99 – 0.87 (m, 2H), 0.769 – 0.61 (m, 2H). **^{13}C NMR** (100 MHz, Chloroform-*d*) δ 168.39, 166.08, 162.92, 160.84, 151.02, 148.73, 133.80, 132.68, 129.82, 128.67, 128.32, 128.19, 126.76, 126.15, 124.89, 119.98, 115.58, 112.91, 62.90, 53.33, 33.53, 31.75, 10.81, 7.72, 7.45. **LCMS** (ES+) m/z calcd for $C_{25}H_{21}N_3O_4S_2$ $[M+H]^+$ 492.58, found 492.2

(±) Methyl 8-cyclopropyl-5-oxo-7-(((5-(3-(trifluoromethyl)phenyl)-1,3,4-oxadiazol-2-yl)thio)methyl)-2,3-dihydro-5H-thiazolo[3,2-a]pyridine-3-carboxylate (7b)

Synthesized following the general procedure for the condensation of 2-pyridone and 5-Substituted-1,3,4-oxadiazole-2-thiols. Compound obtained as white solid, 152 mg (0.298 mmol, 92%). **^{1}H NMR** (400 MHz, Chloroform-*d*) δ 8.18 (s, 1H), 8.13 (d, *J* = 7.9 Hz, 1H), 7.73 – 7.71 (m, 1H), 7.61 – 7.57 (m, 1H), 6.40 (s, 1H), 5.51 (dd, *J* = 8.6, 2.3 Hz, 1H), 4.58 – 4.43 (m, 2H), 3.72 (s, 3H), 3.61 (dd, *J* = 11.8, 8.6 Hz, 1H), 3.44 (dd, *J* = 11.8, 2.3 Hz, 1H), 1.71 – 1.64 (m, 1H), 0.98 – 0.87 (m, 2H), 0.67– 0.63 (m, 2H). **^{13}C NMR** (100 MHz, Chloroform-*d*) δ 168.35, 164.86, 163.94,

160.77, 150.76, 148.83, 131.77 (q, *J* = 33.1 Hz), 129.82 (d, *J* = 2.9 Hz), 128.27 (d, *J* = 3.6 Hz), 124.81, 124.32, 123.56 (q, J = 3.9 Hz), 122.10, 115.55, 112.77, 62.89, 53.31, 33.57, 31.72, 10.75, 7.68, 7.43. **^{19}F NMR** (376 MHz, Chloroform-*d*) δ -62.99. **LCMS** (ES+) m/z calcd for $C_{22}H_{18}F_3N_3O_4S_2$ $[M+H]^+$ 510.52, found 510.10.

(±) Methyl 8-cyclopropyl-7-(((5-(naphthalen-1-ylmethyl)-1,3,4-oxadiazol-2-yl)thio)methyl)-5-oxo-2,3-dihydro-5H-thiazolo[3,2-a]pyridine-3-carboxylate (7c)

Prepared by following the general procedure. Compound obtained as transparent semi-solid 102 mg (0.202 mmol, 85%). **^{1}H NMR** (400 MHz, Chloroform-*d*) δ 8.02 (d, *J* = 8.6 Hz, 1H), 7.78 (d, *J* = 1.5 Hz, 1H), 7.76 – 7.73 (m, 1H), 7.50 – 7.39 (m, 2H), 7.38 – 7.36 (m, 2H), 6.29 (s, 1H), 5.47 (dd, *J* = 8.6, 2.3 Hz, 1H), 4.53 (s, 2H), 4.38 – 4.22 (m, 2H), 3.70 (s, 3H), 3.55 (dd, *J* = 11.8, 8.6 Hz, 1H), 3.40 (dd, *J* = 11.8, 2.3 Hz, 1H), 1.58 – 1.51 (m, 1H), 0.86 – 0.75 (m, 2H), 0.58 – 0.49 (m, 2H). **^{13}C NMR** (100 MHz, Chloroform-*d*) δ 168.38, 166.66, 163.44, 160.77, 150.94, 148.63, 133.87, 131.61, 129.56, 128.83, 128.69, 127.76, 126.81, 126.08, 125.55, 123.42, 115.44, 112.82, 62.86, 53.31, 33.42, 31.70, 29.62, 10.68, 7.60, 7.34. **LCMS** (ES+) m/z calcd for $C_{26}H_{23}N_3O_4S_2$ $[M+H]^+$ 506.60, found 506.2

(±) Methyl 8-cyclopropyl-7-(((5-(naphthalen-2-yl)-1,3,4-oxadiazol-2-yl)thio)methyl)-5-oxo-2,3-dihydro-5H-thiazolo[3,2-a]pyridine-3-carboxylate (7d)

Prepared by following the general procedure. Compound obtained as white solid 103 mg (0.209 mmol, 95%). **^{1}H NMR** (600 MHz, Chloroform-*d*) δ 8.26 – 8.25 (m, 1H), 7.87 – 7.83 (m, 1H), 7.74 – 7.72 (m, 2H), 7.66 (dd, *J* = 7.8, 1.7 Hz, 1H), 7.38 – 7.33 (m, 2H), 6.31 (s, 1H), 5.38 (dd, *J* = 8.6, 2.2 Hz, 1H), 4.52 – 4.20 (m, 2H), 3.56 (s, 3H), 3.45 (dd, *J* = 11.7, 8.6 Hz, 1H), 3.30 (dd, *J* = 11.7, 2.2 Hz, 1H), 1.57 – 1.53 (m, 1H), 0.83 – 0.73 (m, 2H), 0.52 – 0.47 (m, 2H). **^{13}C NMR** (151 MHz, Chloroform-*d*) δ 168.34, 166.33, 163.21, 160.93, 151.14, 148.81, 134.70, 132.81, 129.10, 128.91, 128.04, 127.95, 127.22, 127.14, 122.97, 120.70, 115.62, 113.11, 62.95, 53.35, 33.62, 31.76, 10.80, 7.70, 7.44. **LCMS** (ES+) m/z calcd for $C_{25}H_{21}N_3O_4S_2$ $[M+H]^+$ 492.58, found 492.1.

(±) Methyl 8-cyclopropyl-7-(((5-(naphthalen-2-ylmethyl)-1,3,4-oxadiazol-2-yl)thio)methyl)-5-oxo-2,3-dihydro-5H-thiazolo[3,2-a]pyridine-3-carboxylate (7e)

Prepared by following the general procedure. Compound obtained as white solid 87 mg (0.172 mmol, 83%). **^{1}H NMR** (600 MHz, Chloroform-*d*) δ 7.86 – 7.83 (m, 3H), 7.78 (d, *J* = 1.7 Hz, 1H), 7.52 – 7.48 (m, 2H), 7.42 (dd, *J* = 8.4, 1.8 Hz, 1H), 6.43 (s, 1H), 5.59 (dd, *J* = 8.6, 2.2 Hz, 1H), 4.52 – 4.38 (m, 2H), 4.36 (s, 2H), 3.80 (s, 3H), 3.65 (dd, *J* = 11.7, 8.6 Hz, 1H), 3.51 (dd, *J* = 11.7, 2.2 Hz, 1H), 1.70 – 1.66 (m, 1H), 0.99 – 0.89 (m, 2H), 0.68 – 0.64 (m, 2H). **^{13}C NMR** (151 MHz, Chloroform-*d*) δ 168.34, 166.75, 163.62, 160.89, 151.05, 148.70, 133.42, 132.63, 130.88, 128.80, 127.79, 127.73, 127.71, 126.63, 126.46, 126.18, 115.46, 113.01, 62.91, 53.34, 33.45, 32.09, 31.72, 10.70, 7.62, 7.34. **LCMS** (ES+) m/z calcd for $C_{26}H_{23}N_3O_4S_2$ $[M+H]^+$ 506.60, found 506.2.

(±) Methyl 7-(((5-(4-(tert-butyl)phenyl)-1,3,4-oxadiazol-2-yl)thio)methyl)-8-cyclopropyl-5-oxo-2,3-dihydro-5H-thiazolo[3,2-a]pyridine-3-carboxylate (7f)

Prepared by following the general procedure. Compound obtained as white solid 120 mg (0.241 mmol, 72%). **^{1}H NMR** (600 MHz, Chloroform-*d*) δ 7.93 (d, *J* = 8.5 Hz, 2H), 7.52 (d, *J* = 8.5 Hz, 2H), 6.48 (s, 1H), 5.59 (dd, *J* = 8.6, 2.3 Hz, 1H), 4.56 (dd, *J* = 75.2, 13.9 Hz, 2H), 3.80 (s, 3H), 3.68 (dd, *J* = 11.7, 8.6 Hz, 1H), 3.52 (dd, *J* = 11.7, 2.3 Hz, 1H), 1.79 – 1.74 (m, 1H), 1.37 (s, 9H),

1.05 – 0.95 (m, 2H), 0.75 – 0.69 (m, 2H). **^{13}C NMR** (151 MHz, Chloroform-*d*) δ 168.41, 166.22, 162.71, 160.80, 155.40, 150.98, 148.64, 126.56 (2C), 126.06 (2C), 120.64, 115.60, 112.80, 62.87, 53.30, 35.09, 33.61, 31.73, 31.11 (3C), 10.77, 7.68, 7.40. **LCMS** (ES+) m/z calcd for $C_{25}H_{27}N_3O_4S_2$ $[M+H]^+$ 498.63, found 498.2.

(±) Methyl 8-cyclopropyl-7-(((5-(4-nitrophenyl)-1,3,4-oxadiazol-2-yl)thio)methyl)-5-oxo-2,3-dihydro-5H-thiazolo[3,2-a]pyridine-3-carboxylate (7g)

Prepared by following the general procedure. Compound obtained as white solid 76 mg (0.156 mmol, 70%). **^{1}H NMR** (600 MHz, Chloroform-*d*) δ 8.39 (d, *J* = 8.9 Hz, 2H), 8.21 (d, *J* = 8.8 Hz, 2H), 6.50 (s, 1H), 5.60 (dd, *J* = 8.6, 2.2 Hz, 1H), 4.66 – 4.54 (m, 2H), 3.81 (s, 3H), 3.69 (dd, *J* = 11.8, 8.6 Hz, 1H), 3.54 (dd, *J* = 11.7, 2.3 Hz, 1H), 1.78 – 1.74 (m, 1H), 1.07 – 0.97 (m, 2H), 0.75 – 0.72 (m, 2H). **^{13}C NMR** (151 MHz, Chloroform-*d*) δ 168.32, 164.81, 164.34, 160.77, 150.66, 149.52, 148.90, 128.91, 127.61 (2C), 124.45 (2C), 115.57, 112.72, 62.90, 53.34, 33.57, 31.72, 10.74, 7.69, 7.49. **LCMS** (ES+) m/z calcd for $C_{21}H_{18}N_4O_6S_2$ $[M+H]^+$ 487.52, found 487.1.

(±) Methyl 8-cyclopropyl-7-(((5-(3-methoxyphenyl)-1,3,4-oxadiazol-2-yl)thio)methyl)-5-oxo-2,3-dihydro-5H-thiazolo[3,2-a]pyridine-3-carboxylate (7h)

Prepared by following the general procedure. Compound obtained as white solid 58 mg (0.123 mmol, 67%). **^{1}H NMR** (600 MHz, Chloroform-*d*) δ 7.59 – 7.57 (m, 1H), 7.54 (dd, *J* = 2.7, 1.5 Hz, 1H), 7.41 (t, *J* = 8.0 Hz, 1H), 7.09 – 7.07 (m, 1H), 6.48 (s, 1H), 5.59 (dd, *J* = 8.6, 2.2 Hz, 1H), 4.57 (dd, *J* = 74.1, 13.8 Hz, 2H), 3.89 (s, 3H), 3.80 (s, 3H), 3.68 (dd, *J* = 11.8, 8.6 Hz, 1H), 3.53 (dd, *J* = 11.7, 2.2 Hz, 1H), 1.78 – 1.74 (m, 1H), 1.05 – 0.95 (m, 2H), 0.74 – 0.71 (m, 2H). **^{13}C NMR** (151 MHz, Chloroform-*d*) δ 168.40, 166.06, 163.13, 160.81, 159.94, 150.93, 148.69, 130.24, 124.54, 119.13, 118.38, 115.59, 112.81, 111.20, 62.88, 55.53, 53.31, 33.57, 31.73, 10.77, 7.68, 7.40. **LCMS** (ES+) m/z calcd for $C_{22}H_{21}N_3O_5S_2$ $[M+H]^+$ 472.54, found 472.2.

(±) Methyl 8-cyclopropyl-5-oxo-7-(((5-(pyridin-4-yl)-1,3,4-oxadiazol-2-yl)thio)methyl)-2,3-dihydro-5H-thiazolo[3,2-a]pyridine-3-carboxylate (7i)

Prepared by following the general procedure. Compound obtained as white solid 140 mg (0.316 mmol, 79%). **^{1}H NMR** (400 MHz, Chloroform-d) δ 8.58 (d, *J* = 5.1 Hz, 1H), 7.63 (d, *J* = 6.1 Hz, 1H), 6.24 (s, 1H), 5.35 (dd, J = 8.7, 2.3 Hz, 1H), 4.43 – 4.29 (m, 2H), 3.56 (s, 3H), 3.47 (dd, J = 11.8, 8.7 Hz, 1H), 3.30 (dd, J = 11.8, 2.4 Hz, 1H), 1.55 – 1.48 (m, 1H), 0.83 – 0.71 (m, 2H), 0.53 – 0.46 (m, 2H). **^{13}C NMR** (100 MHz, Chloroform-*d*) δ 168.34, 164.77, 164.17, 160.70, 150.81 (2C), 150.57 (2C), 148.85, 130.52, 120.05, 115.54, 112.53, 62.84, 53.28, 33.51, 31.67, 10.71, 7.66, 7.45. **LCMS** (ES+) m/z calcd for $C_{20}H_{18}N_4O_4S_2$ $[M+H]^+$ 443.51, found 443.1.

(±) Methyl 8-cyclopropyl-5-oxo-7-(((5-(thiophen-2-yl)-1,3,4-oxadiazol-2-yl)thio)methyl)-2,3-dihydro-5H-thiazolo[3,2-a]pyridine-3-carboxylate (7j)

Prepared by following the general procedure. Compound obtained as white solid 48 mg (0.107 mmol, 64%). **^{1}H NMR** (600 MHz, Chloroform-d) δ 7.73 (dd, J = 3.8, 1.2 Hz, 1H), 7.56 (dd, J = 5.0, 1.2 Hz, 1H), 7.18 (dd, J = 5.0, 3.7 Hz, 1H), 6.51 (s, 1H), 5.61 (dd, J = 8.7, 2.2 Hz, 1H), 4.56 (dd, J = 78.6, 13.8 Hz, 2H), 3.81 (s, 3H), 3.70 (dd, J = 11.7, 8.6 Hz, 1H), 3.54 (dd, J = 11.7, 2.0 Hz, 1H), 1.79 – 1.75 (m, 1H), 1.06 – 0.96 (m, 2H), 0.75 – 0.70 (m, 2H). **^{13}C NMR** (151 MHz, Chloroform-*d*) δ 168.31, 162.44, 162.31, 160.83, 151.11, 148.86, 130.25, 129.82, 128.19, 124.63, 115.48, 113.15, 62.96, 53.35, 33.62, 31.77, 10.78, 7.70, 7.41. **LCMS** (ES+) m/z calcd for $C_{19}H_{17}N_3O_4S_3$ $[M+H]^+$ 448.55, found 448.1.

(±) Methyl 8-cyclopropyl-7-(((5-(furan-2-yl)-1,3,4-oxadiazol-2-yl)thio)methyl)-5-oxo-2,3-dihydro-5H-thiazolo[3,2-a]pyridine-3-carboxylate (7k)

Prepared by following the general procedure. Compound obtained as white solid 75 mg (0.173 mmol, 74%). **^{1}H NMR** (600 MHz, Chloroform-*d*) δ 7.65 (dd, *J* = 1.8, 0.8 Hz, 1H), 7.15 (dd, *J* = 3.6, 0.9 Hz, 1H), 6.61 (dd, *J* = 3.6, 1.8 Hz, 1H), 6.45 (s, 1H), 5.59 (dd, *J* = 8.7, 2.2 Hz, 1H), 4.62 – 4.48 (m, 2H), 3.80 (s, 3H), 3.69 (dd, *J* = 11.8, 8.6 Hz, 1H), 3.53 (dd, *J* = 11.7, 2.3 Hz, 1H), 1.77 – 1.73 (m, 1H), 1.05 – 0.95 (m, 2H), 0.74 – 0.70 (m, 2H). **^{13}C NMR** (151 MHz, Chloroform-*d*) δ 168.38, 162.58, 160.77, 158.82, 150.75, 148.76, 145.78, 138.93, 115.63, 114.16, 112.77, 112.19, 62.88, 53.32, 33.68, 31.73, 10.74, 7.66, 7.39. **LCMS** (ES+) m/z calcd for $C_{19}H_{17}N_3O_5S_2$ $[M+H]^+$ 432.48, found 432.4.

(±) Methyl 8-cyclopropyl-7-(((5-((4-fluorophenoxy)methyl)-1,3,4-oxadiazol-2-yl)thio)methyl)-5-oxo-2,3-dihydro-5H-thiazolo[3,2-a]pyridine-3-carboxylate (7l)

Prepared by following the general procedure. Compound obtained as brown solid 168 mg (0.343 mmol, 79%). **^{1}H NMR** (600 MHz, Chloroform-*d*) δ 7.01 – 6.94 (m, 4H), 6.41 (s, 1H), 5.57 (dd, *J* = 8.7, 2.3 Hz, 1H), 5.19 (s, 2H), 4.56 – 4.43 (m, 1H), 3.78 (s, 3H), 3.67 (dd, *J* = 11.8, 8.7 Hz, 1H), 3.50 (dd, *J* = 11.7, 2.3 Hz, 1H), 1.72 – 1.68 (m, 1H), 1.01 – 0.92 (m, 2H), 0.69 – 0.67 (m, 2H). **^{13}C NMR** (151 MHz, Chloroform-*d*) δ 168.34, 164.87, 163.52, 160.75, 158.84, 157.25, 153.55 (d, *J* = 2.4 Hz), 150.70, 148.82, 116.24, 116.20, 116.09, 115.48, 112.69, 62.87, 60.47, 53.30, 33.46, 31.69, 10.70, 7.64, 7.40. **^{19}F NMR** (565 MHz, Chloroform-*d*) δ -121.73. **LCMS** (ES+) m/z calcd for $C_{22}H_{20}FN_3O_5S_2$ $[M+H]^+$ 490.54, found 490.1.

***Procedure for the Synthesis of azide intermediates (IIa-b)*:**

Aniline (5.4 mmol, 1.0 equiv) was taken in a dried flask and dissolved in 5 mL EtOAc. The resulting mixture was cooled in an ice bath. Concentrated HCl (2.2 mL) was added dropwise, followed by the dropwise addition of a solution of $NaNO_2$ (6.5 mmol, 1.2 equiv) in 2.5 mL of water over the course of 15 min. The resulting mixture was stirred for 1 h. A solution of NaN_3 (6.5 mmol, 1.2 equiv) in 2.5 mL water was added dropwise to the reaction at 0 °C. The ice bath was removed and the reaction was carried out at room temperature for an additional 3 h. The reaction was quenched with cold water and extracted with EtOAc. The organic layer was separated, washed with water and brine, dried over Na_2SO_4 and concentrated under reduced pressure. The crude product was used for following reactions without further purification.

Scheme S3. Synthesis of oxime intermediates (IIa-b).

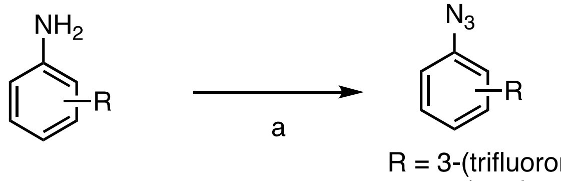

R = 3-(trifluoromethyl)phenyl (IIa)
= 4-(*tert*-butyl)phenyl (IIb)

Reagents and conditions: (a) $NaNO_2$, NaN_3, HCl, EtOAc, 0 °C to rt, 4 h.

General Synthesis of oximes (Ia-p)

To a mixture of aldehyde (3.0 mmol, 1.0 equiv) in MeOH (10 mL) was added hydroxylamine hydrochloride (3.6 mmol, 1.2 equiv) and then NaOAc (4.5 mmol, 1.5 equiv). The mixture is stirred for 4 hours at room temperature. The reaction was monitored through TLC. Upon completion, the reaction mixture was acidified to pH 6 by adding 1M HCl dropwise and extracted with EtOAc (3×40 mL). The combined organic layer was washed with H_2O and brine, dried with Na_2SO_4, and concentrated in vacuo to give the oxime products. The crude products (Ia-p) were used directly in the next reaction.

Scheme S4. Synthesis of oxime intermediates (Ia-p).

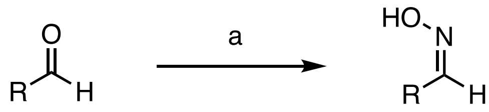

R = naphthalen-1-yl (Ia)
= 3-(trifluoromethyl)phenyl (Ib)
= cyclopropyl (Ic)
= cyclohexyl (Id)
= 4-methoxyphenyl (Ie)
= 4-nitrophenyl (If)
= phenyl (Ig)
= naphthalen-2-yl (Ih)
= anthracen-9-yl (Ii)
= 4-(*tert*-butyl)phenyl (Ij)
= 3,5-di-*tert*-butylphenyl (Ik)
= 3,5-difluorophenyl (Il)
= 4-fluoronaphthalen-1-yl (Im)
= thiophen-2-yl (In)
= benzo[*d*][1,3]dioxol-5-yl (Io)
= quinolin-4-yl (Ip)

Reagents and conditions: (a) $NH_2OH.HCl$, NaOAc, MeOH, rt, 4 h.

2. Structure and Biofilm inhibition activity of thiazolo ring-fused 2-pyridone library

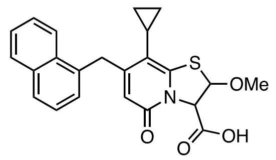

1 (>50μM)

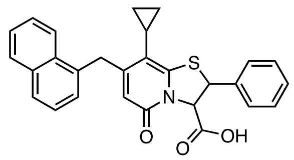

2 (>50μM)

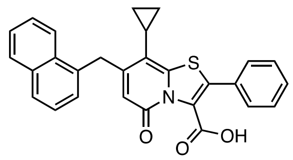

3 (50μM)

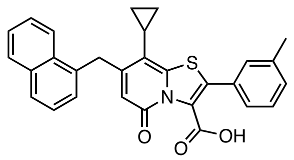

4 (50μM)

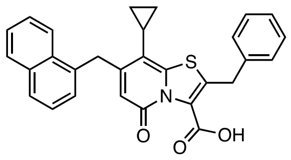

5 (50μM)

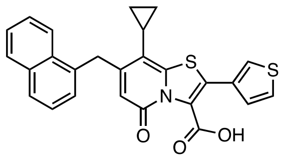

6 (50μM)

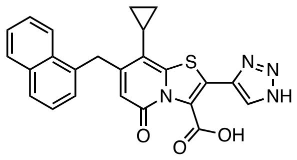

7 (>50μM)

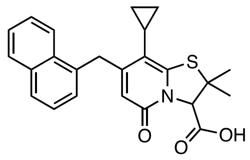

8 (>50μM)

Figure S1: Substituent variation at the C2 position of 2-pyridone library. Synthesized analogues were tested for *Mtb* biofilm formation inhibition. The minimum concentration found to inhibit biofilm formation for individual analogues are presented in parentheses. >50 μM indicates that no inhibition was detected at the highest concentration tested (50 μM).

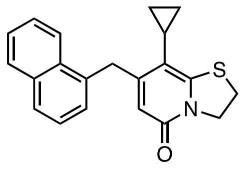

9 (50μM)

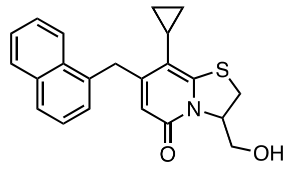

10 (50μM)

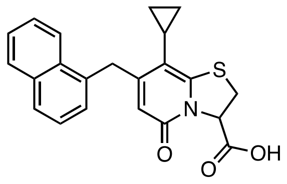

11 (**C10**; 2.5μM)

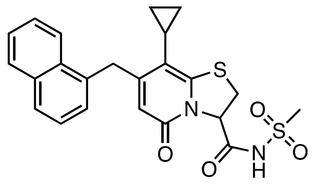

12 (50μM)

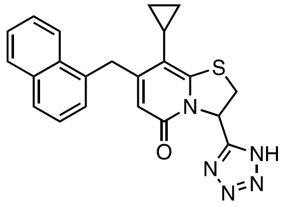

13 (50μM)

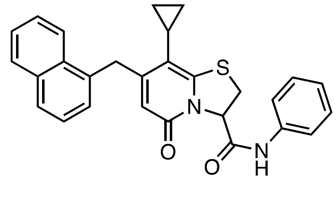

14 (>50μM)

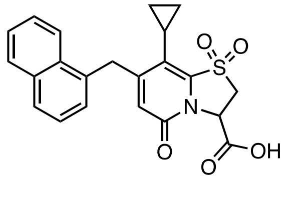

15 (>50μM)

Figure S2: Core scaffold modification and substituent variation at the C3 position of 2-pyridone library. Synthesized analogues were tested for *Mtb* biofilm formation inhibition. The minimum concentration found to inhibit biofilm formation for individual analogues are presented in parentheses. >50 μM indicates that no inhibition was detected at the highest concentration tested (50 μM).

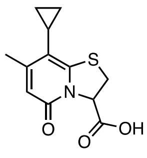
16 (>50μM)

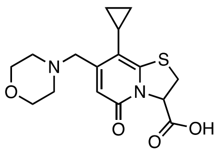
17 (>50μM)

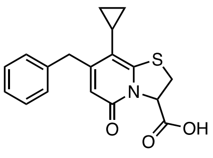
18 (>50μM)

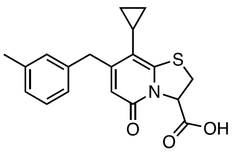
19 (25μM)

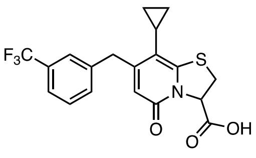
20 (5μM)

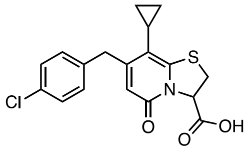
21 (>50μM)

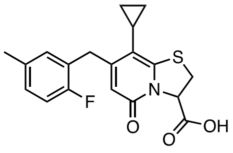
22 (50μM)

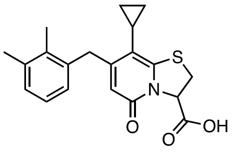
23 (50μM)

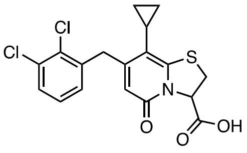
24 (25μM)

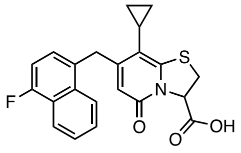
25 (5μM)

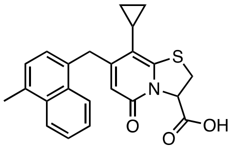
26 (5μM)

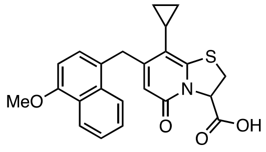
26 (25μM)

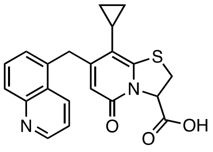
27 (>50μM)

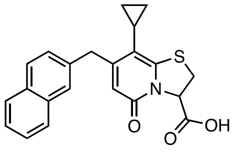
28 (10μM)

Figure S3: Substituent variation at the C7 position of 2-pyridone library. Synthesized analogues were tested for *Mtb* biofilm formation inhibition. The minimum concentration found to inhibit biofilm formation for individual analogues are presented in parentheses. >50 μM indicates that no inhibition was detected at the highest concentration tested (50 μM).

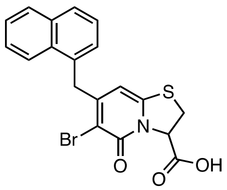
29 (50μM)

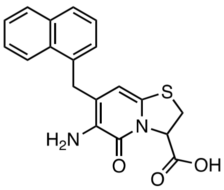
30 (>50μM)

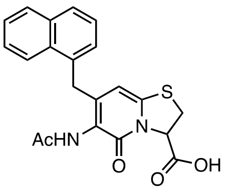
31 (>50μM)

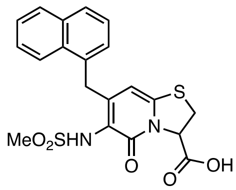
32 (>50μM)

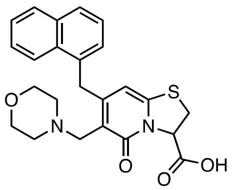
33 (>50μM)

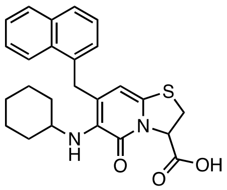
34 (>50μM)

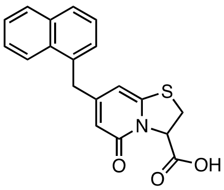
35 (>50μM)

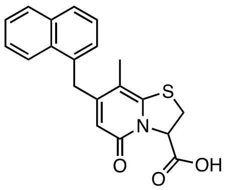
36 (25μM)

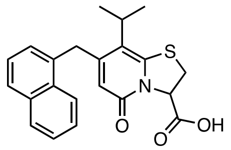
37 (>50μM)

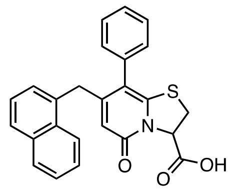
38 (>50μM)

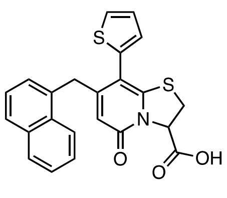
39 (50μM)

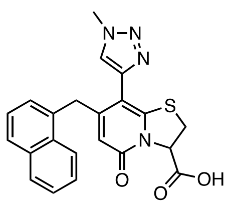
40 (>50μM)

Figure S4: Substituent variation at the C6 and C8 position of 2-pyridone library. Synthesized analogues were tested for *Mtb* biofilm formation inhibition. The minimum concentration found to inhibit biofilm formation for individual analogues are presented in parentheses. >50 μM indicates that no inhibition was detected at the highest concentration tested (50 μM).

3. Permeability and CYP inhibition of selected compounds

Table S1. Membrane permeability and efflux ratio of representative derivatives from oxadiazole, triazole and isoxazole class of heterocyclic spacers.

Compound	Papp A-B (10-6 cm/s)	Efflux ratio
**8f**	3.3	7.7
**15d**	0.88	1.5
**17j**	0.89	5.1

Table S2. CYP3A4 inhibition of representative derivatives from oxadiazole, triazole and isoxazole class of heterocyclic spacers.

Compound	concentration (μM)	% 1OH-midazolam activity	% 6OH-testosterone activity	n
**8f**	1	108 ± 4.1	98 ± 4.0	3
	10	117 ± 1.4	94 ± 5.8	
	100	84 ± 5.0	60 ± 1.9	
**15d**	1	102 ± 1.4	105 ± 1.0	3
	10	95 ± 2.7	101 ± 3.9	
	100	67 ± 1.5	75 ± 1.9	
**17j**	1	106 ± 7.2	100 ± 4.9	3
	10	99 ± 8.1	107 ± 4.5	
	100	56 ± 5.8	61 ± 8.3	
Ketoconazole	0.002	89 ± 1.8	102 ± 6.5	3
	0.02	31 ± 2.6	54 ± 4.2	
	0.2	3.6 ± 0.3	7 ± 1.1	

Table S3. CYP2C9 inhibition of representative derivatives from oxadiazole, triazole and isoxazole class of heterocyclic spacers.

Compound	concentration (μM)	% 4 OH-diclofenac activity	n
**8f**	1	100 ± 2.7	3
	10	93 ± 6.7	
	100	31 ± 3.6	
**15d**	1	83 ± 4.6	3
	10	32.9 ± 1.0	
	100	2.61 ± 0.29	
**17j**	1	42 ± 5.2	3
	10	7.1 ± 0.9	
	100	0.45 ± 0.04	
Sulfaphenazole	0.05	92 ± 5.6	3
	0.5	56 ± 2.5	
	5.0	14 ± 1.6	

4. Inhibition of 17h and 17j growth and survival in the presence of tyloxapol

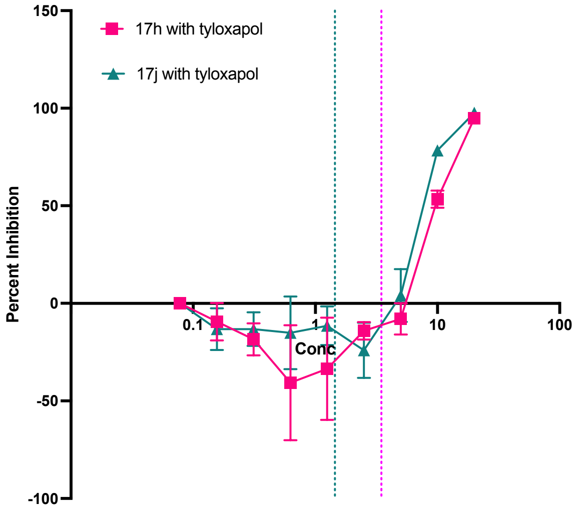

Figure S5. *Mtb* Erdman was incubated in Sauton's media with 0.05% tyloxapol in the presence of increasing doses of each compound for three weeks under hypoxic conditions, followed by three weeks of aerobic conditions. After six weeks total of incubation, the OD_{600} of each well was measured and plotted as percent inhibition compared to the lowest concentration of each compound. Both compounds were prepared as IMD salts and dissolved in DMSO. IMD and DMSO were included in the untreated controls. n = 3 for each compound. Data points represent mean ± SD and the vertical dashed lines designate the IC_{50} for biofilm inhibition for each compound (determined in the absence of tyloxapol).

5. Cytotoxicity of C10, 17h and 17j in murine macrophages

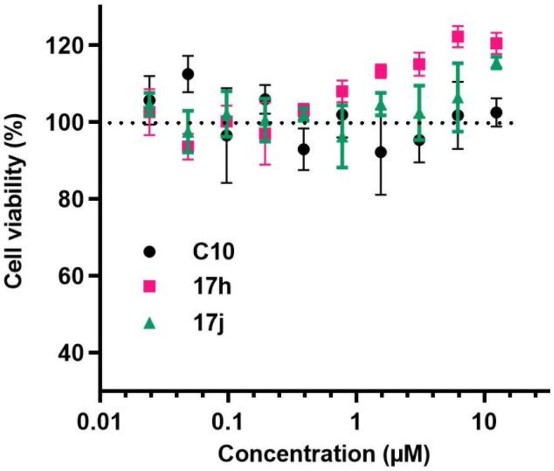

Figure S6: C10, 17h and 17j compounds are not cytotoxic in murine macrophages. A phenotypic-viability cell-based assay on murine Raw 264.7 macrophages was performed by dose-response analysis of the percentage of cell viability for all three compounds. Cell viability was measured by comparison to the average value (total cell number) obtained for the control (DMSO 1%). All three compounds were dissolved in DMSO. n = 4 for each compound. Data points represent mean ± SD.

6. Separation of enantiomers

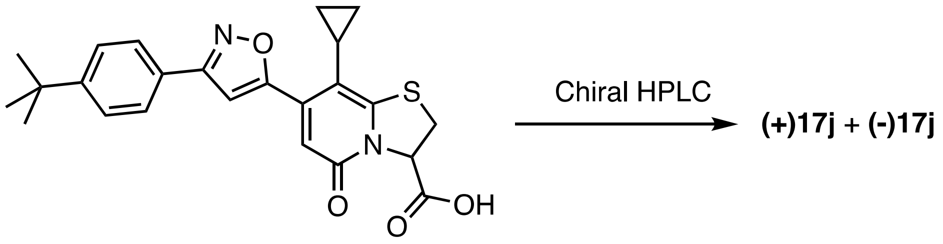

(±) 17j

Scheme S5: Separation of enantiomers of **17j** using chiral HPLC.

To establish the method of enantiomer separation, compound **17j** were injected under variable and isocratic gradient in Lux 5 µm *i*-Amylose-1 (150 x 21.2 mm) chiral column. 45 mg of compound **17j** was dissolved in 1.2 ml of DMSO and injected in four iterations. MeCN/H_2O with 0.15% TFA was used as mobile phase. An isocratic gradient of 55% MeCN (0.15% TFA) and 45% H_2O (0.15% TFA with was run for 55 min. at a constant flow rate of 18 ml/min. The first peak eluted after 26 min. followed by the second peak (**Figure S1**). Fractions were collected manually, concentrated under vacuo and freeze dried. Four separate injections of racemic **17j** were performed in order to get enough material for biological testing. Purity of the enantiomers were confirmed by analytical HPLC (Figure S5, S6 and S7).

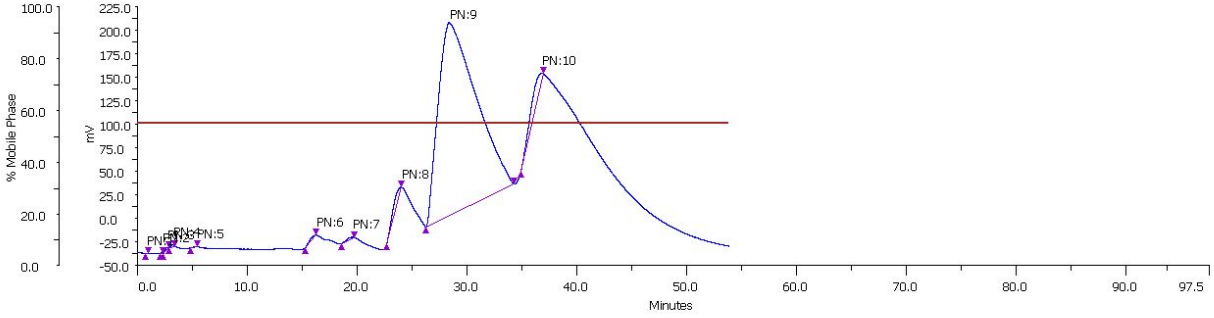

Figure S7. Separation of racemic 17j. The high-performance liquid chromatography (HPLC) of injected racemic **17j**, with Lux 5 µm *i*-Amylose-1(150 x 21.2 mm) chiral column, MeCN/H_2O (0.15% TFA) eluting-solvent system, 18 mL/min flow rate and 254 nm detection wavelength at ambient temperature.

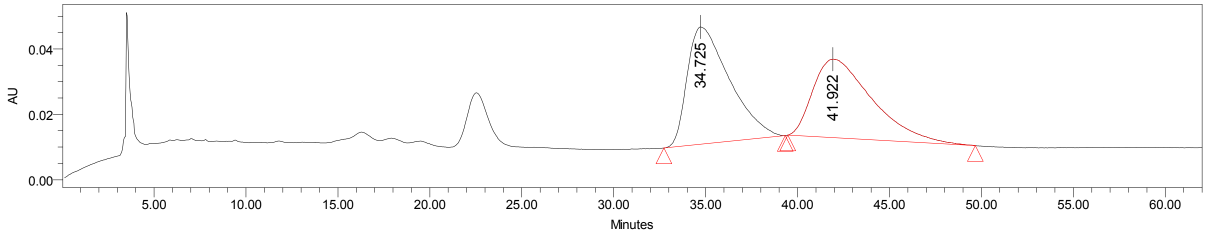

	Name	Retention Time	Area	% Area	Height	Int Type	Amount	Units	Peak Type	Peak Codes
1		34.725	5711545	51.55	35847	bb			Unknown	
2		41.922	5367248	48.45	23987	bb			Unknown	

Figure S8. Chromatogram of racemic 17j. The HPLC chromatogram of injected racemic **17j** (10 µL, 1 mg/mL in DMSO), with Lux 5 µm *i*-Amylose-1 (250 x 4.6 mm) chiral column, isocratic 55% MeCN + 45% H_2O (with 0.15% TFA) eluting solvent system, 0.8 mL/min flow rate and 254 nm detection wavelength at ambient temperature.

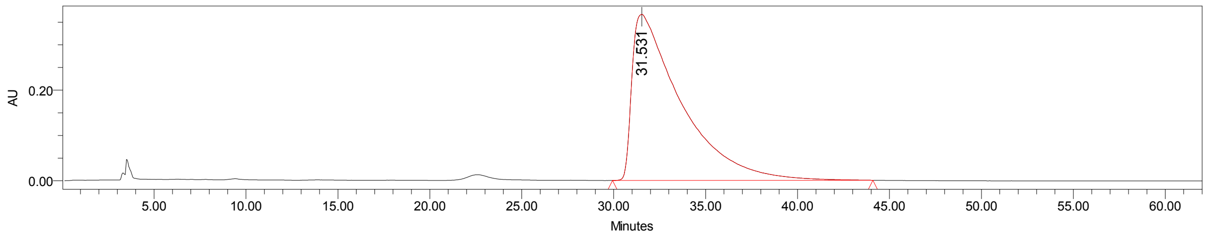

	Name	Retention Time	Area	% Area	Height	Int Type	Amount	Units	Peak Type	Peak Codes
1		31.531	68239798	100.00	366244	bb			Unknown	

Figure S9. **Chromatogram of (+)17j.** The HPLC chromatogram of injected pure enantiomer of **(+)17j** (10 µL, 1 mg/mL in DMSO), with Lux 5 µm *i*-Amylose-1 (250 x 4.6 mm) chiral column, isocratic 55% MeCN + 45% H_2O (with 0.15% TFA) eluting-solvent system, 0.8 mL/min flow rate and 254 nm detection wavelength at ambient temperature. (+)**17j**: $[\alpha]_D^{25}$ +180 (c 0.095, DMSO).

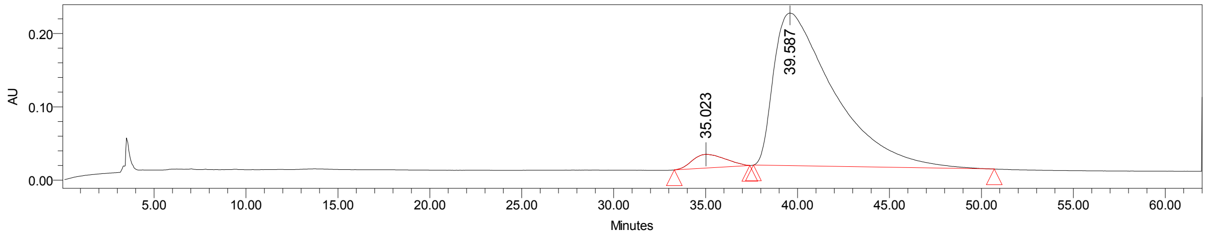

	Name	Retention Time	Area	% Area	Height	Int Type	Amount	Units	Peak Type	Peak Codes
1		35.023	2302872	4.61	18650	bb			Unknown	
2		39.587	47648560	95.39	207942	bb			Unknown	

Figure S10. Chromatogram of (-)17j. The HPLC chromatogram of injected pure enantiomer of **(-)17j** (10 µL, 1 mg/mL in DMSO), with Lux 5 µm *i*-Amylose-1 (250 x 4.6 mm) chiral column, isocratic 55% MeCN + 45% H_2O (with 0.15% TFA) eluting-solvent system, 0.8 mL/min flow rate and 254 nm detection wavelength at ambient temperature. (-)**17j**: $[\alpha]_D^{25}$ -178 (c 0.093, DMSO).

7. NMR Data

^{1}H NMR of **2**

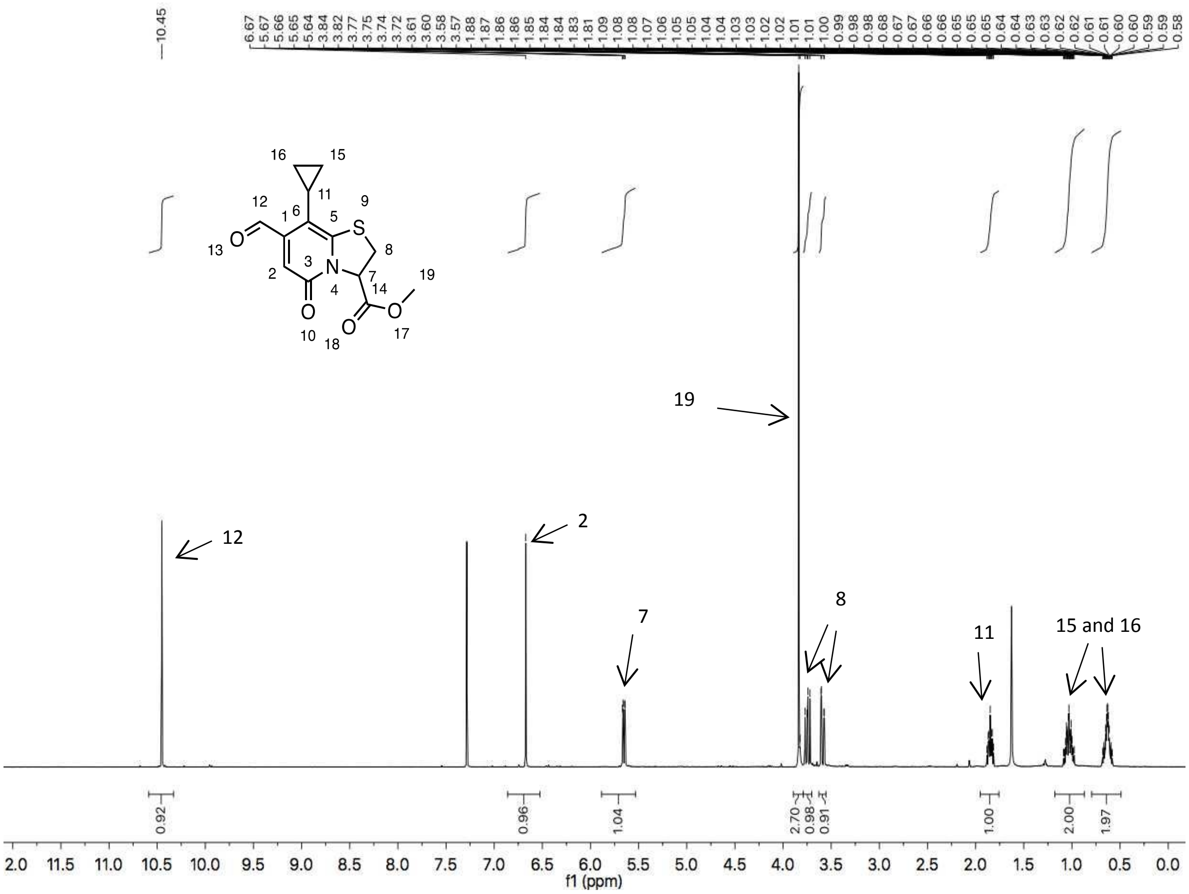

^{13}C NMR of **2**

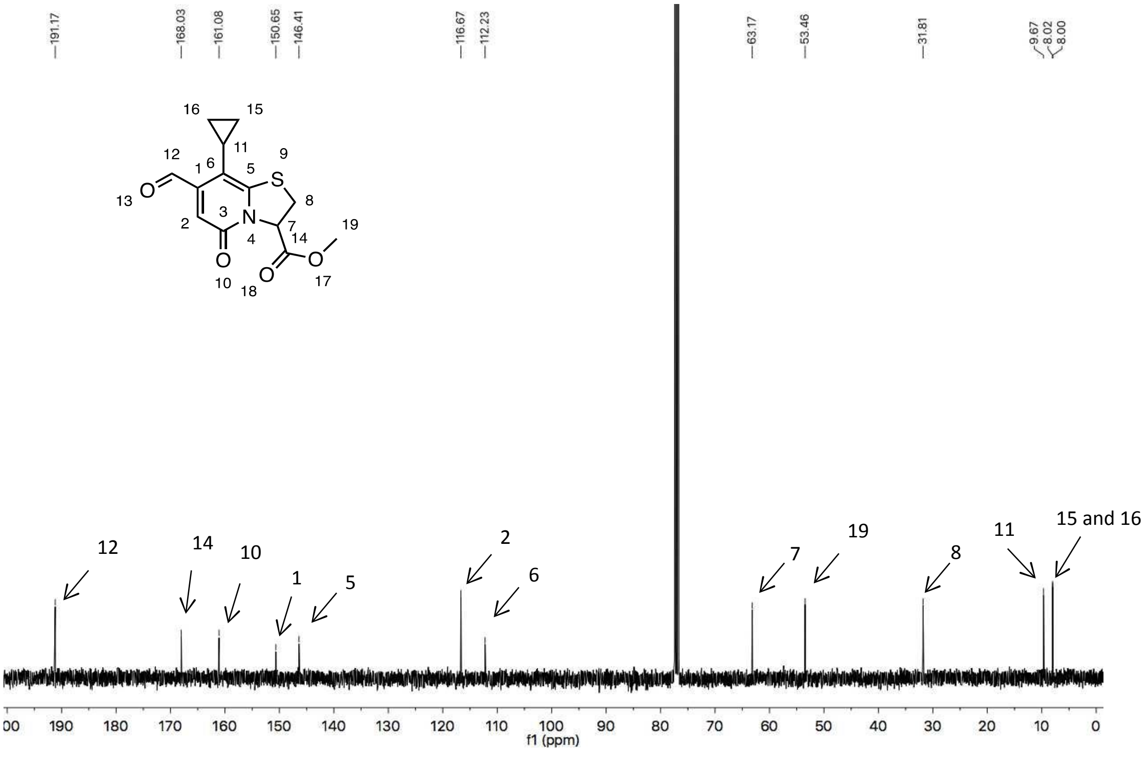

^{1}H NMR of **3**

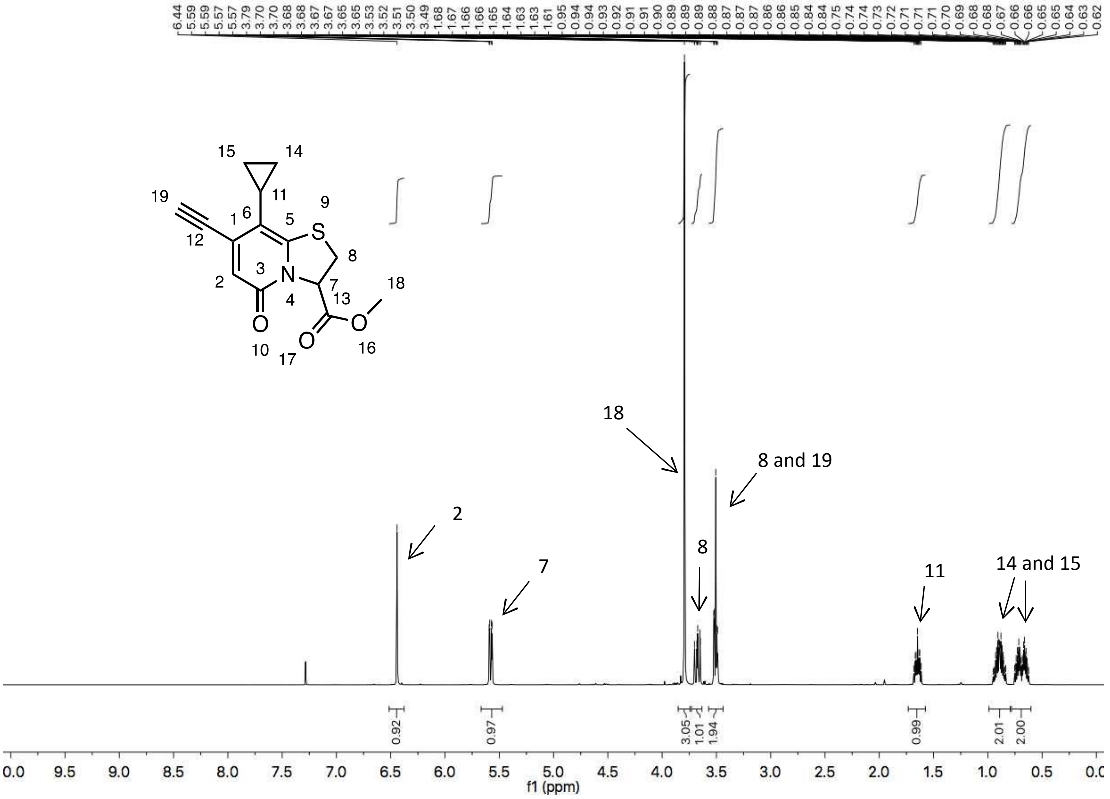

^{13}C NMR of **3**

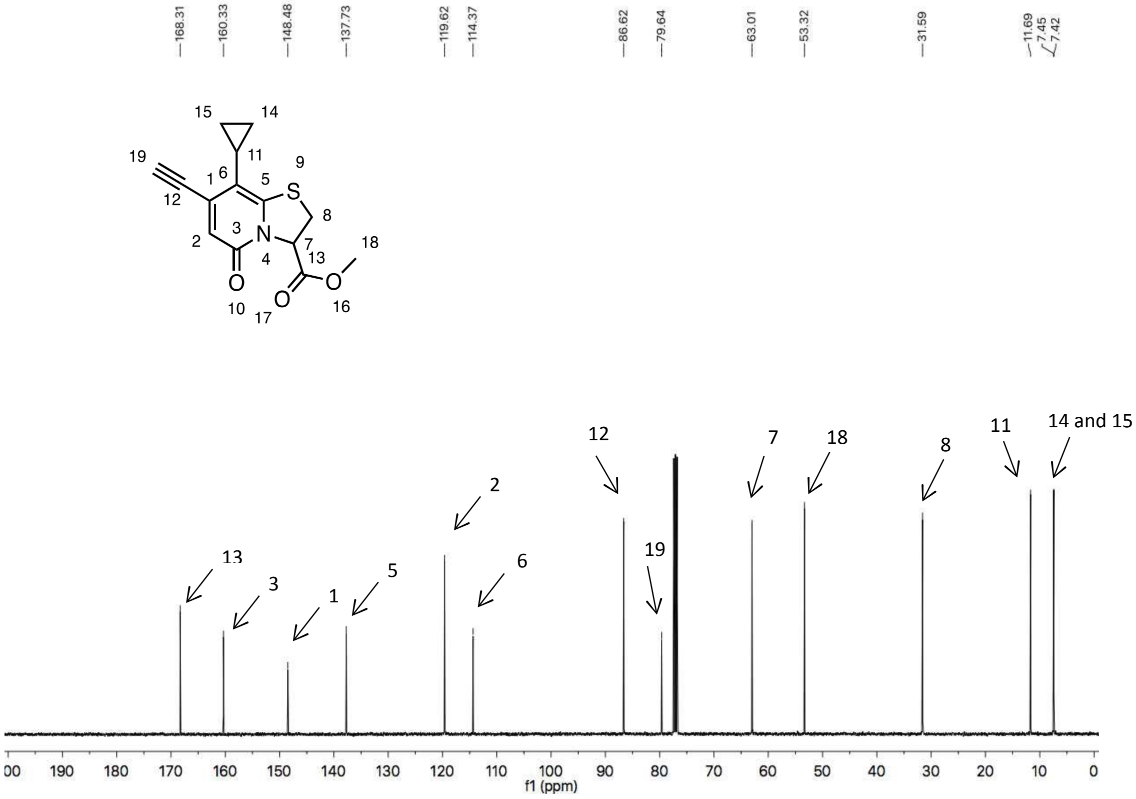

^{1}H NMR of **4**

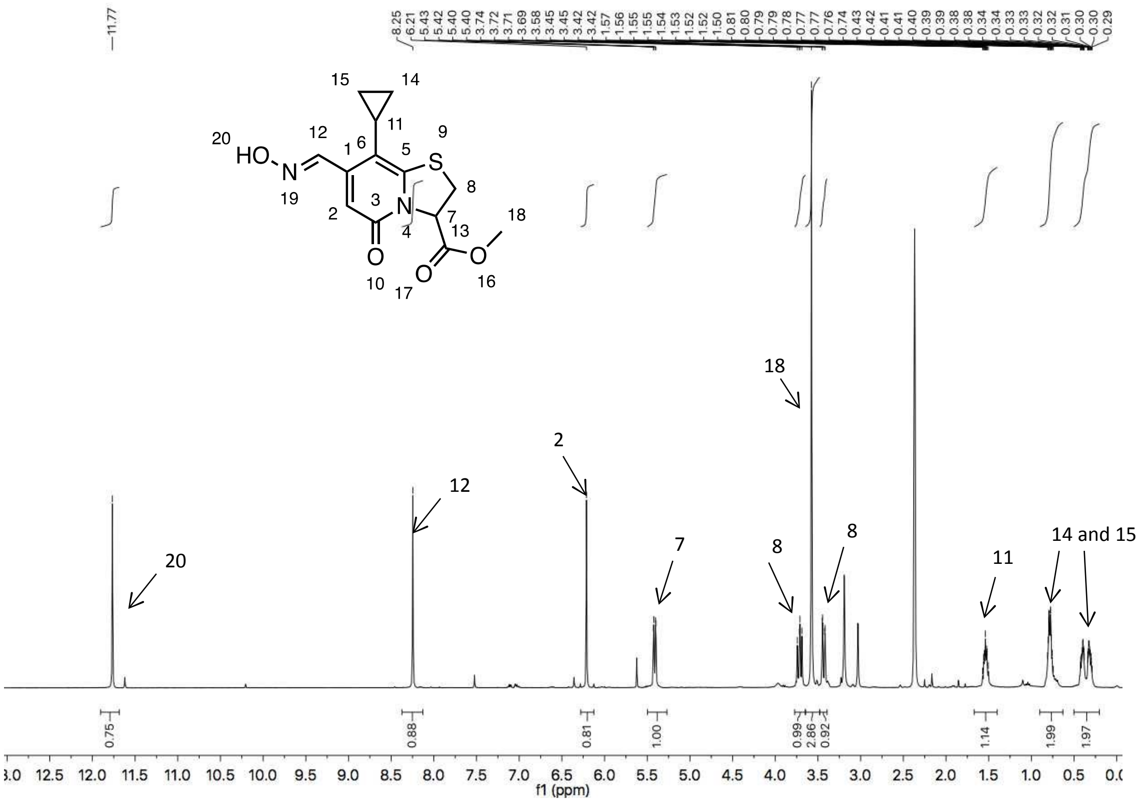

^{13}C NMR of **4**

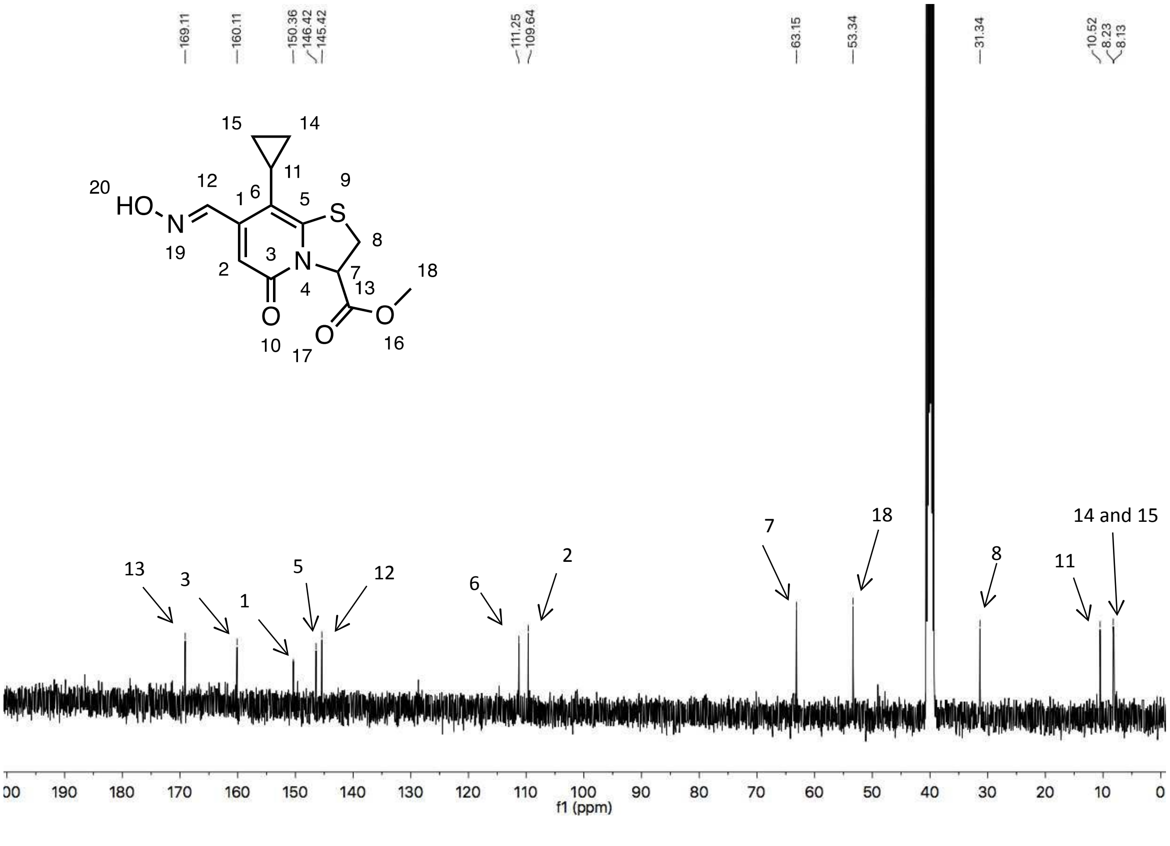

^{1}H NMR of **7a**

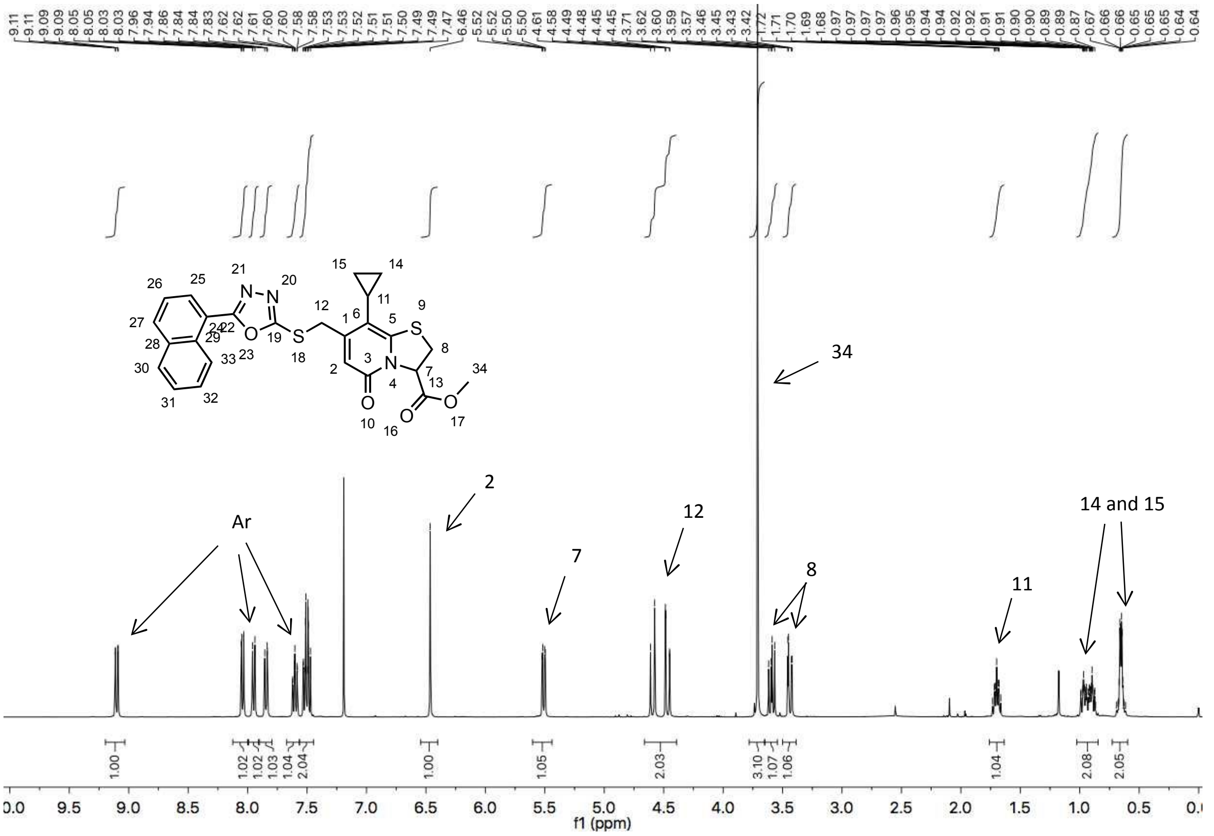

^{13}C NMR of **7a**

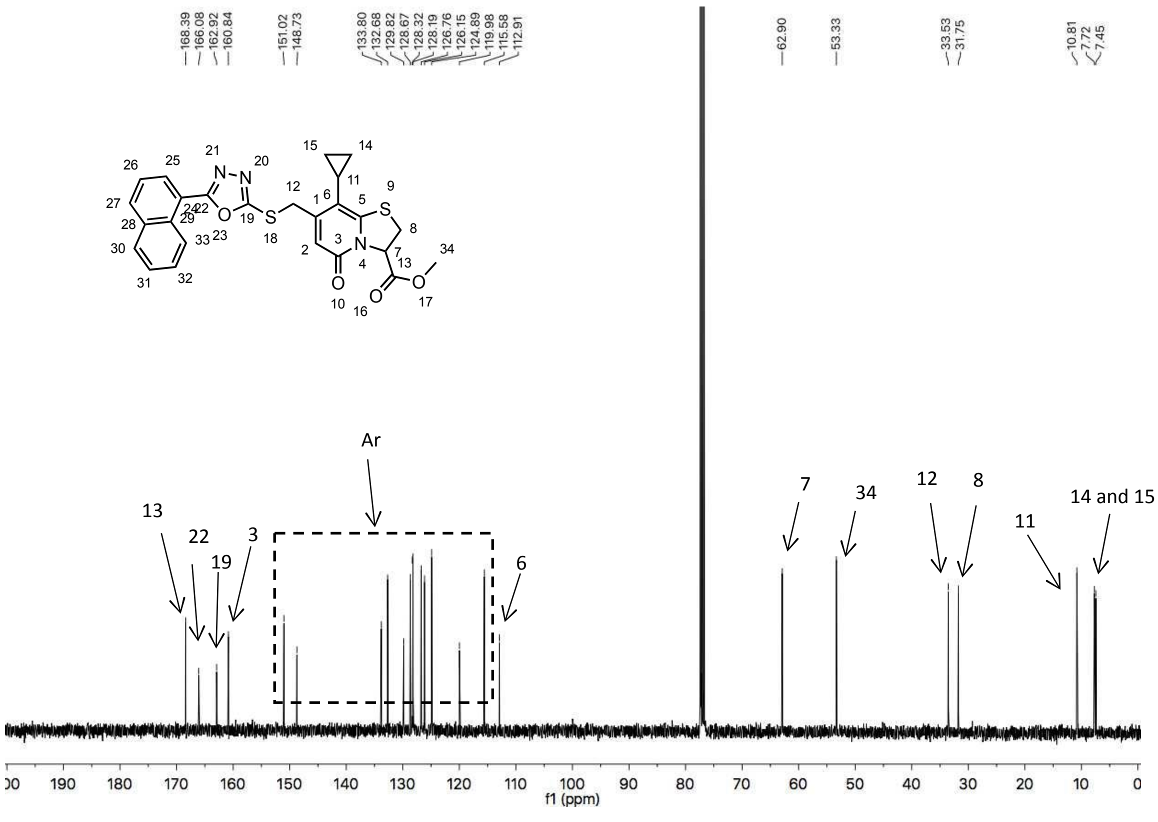

^{1}H NMR of **7b**

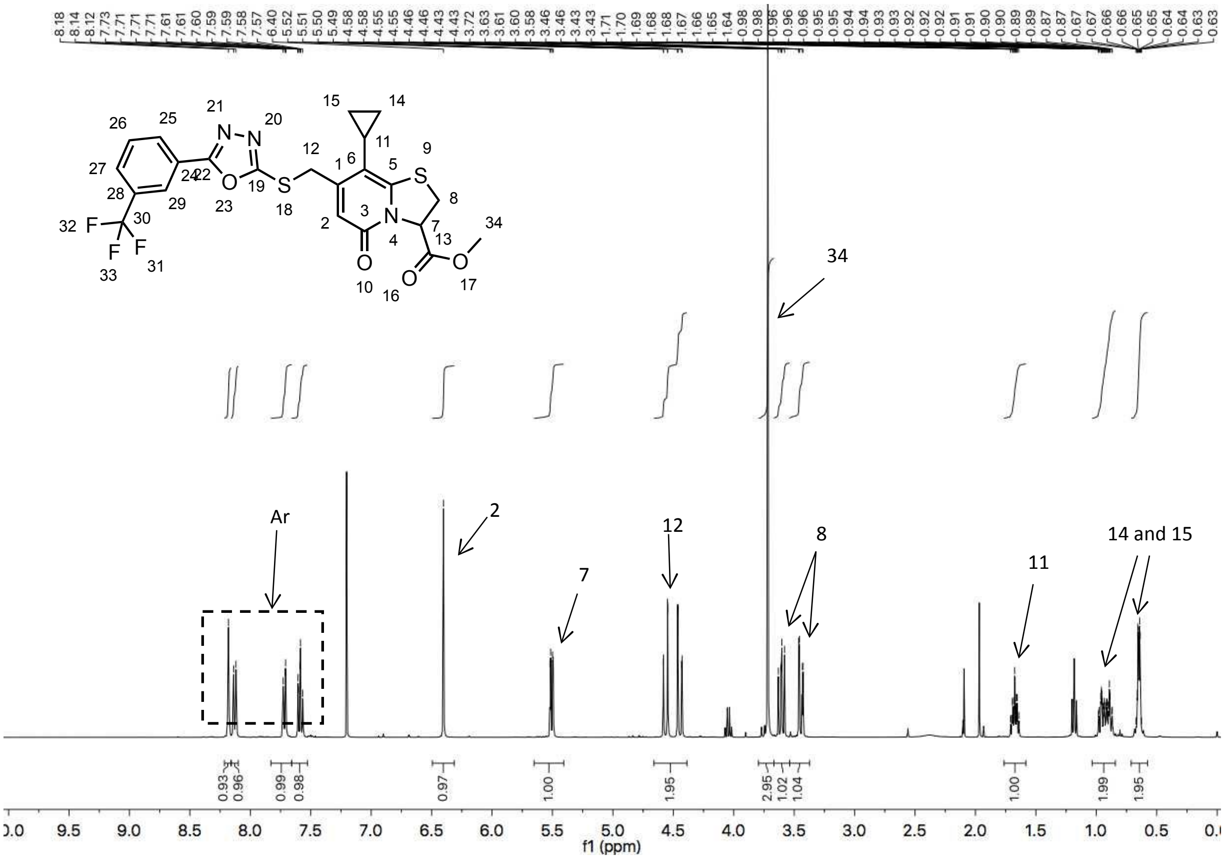

^{13}C NMR of **7b**

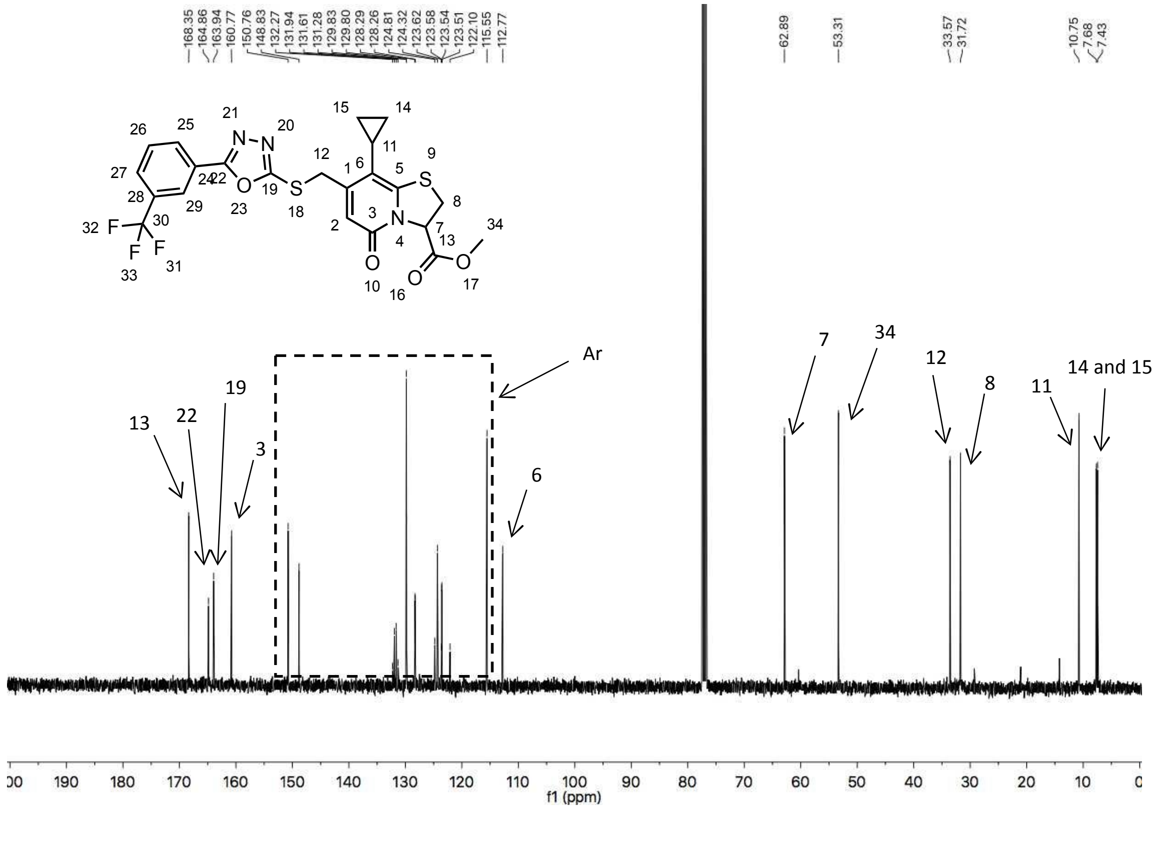

^{19}F NMR of **7b**

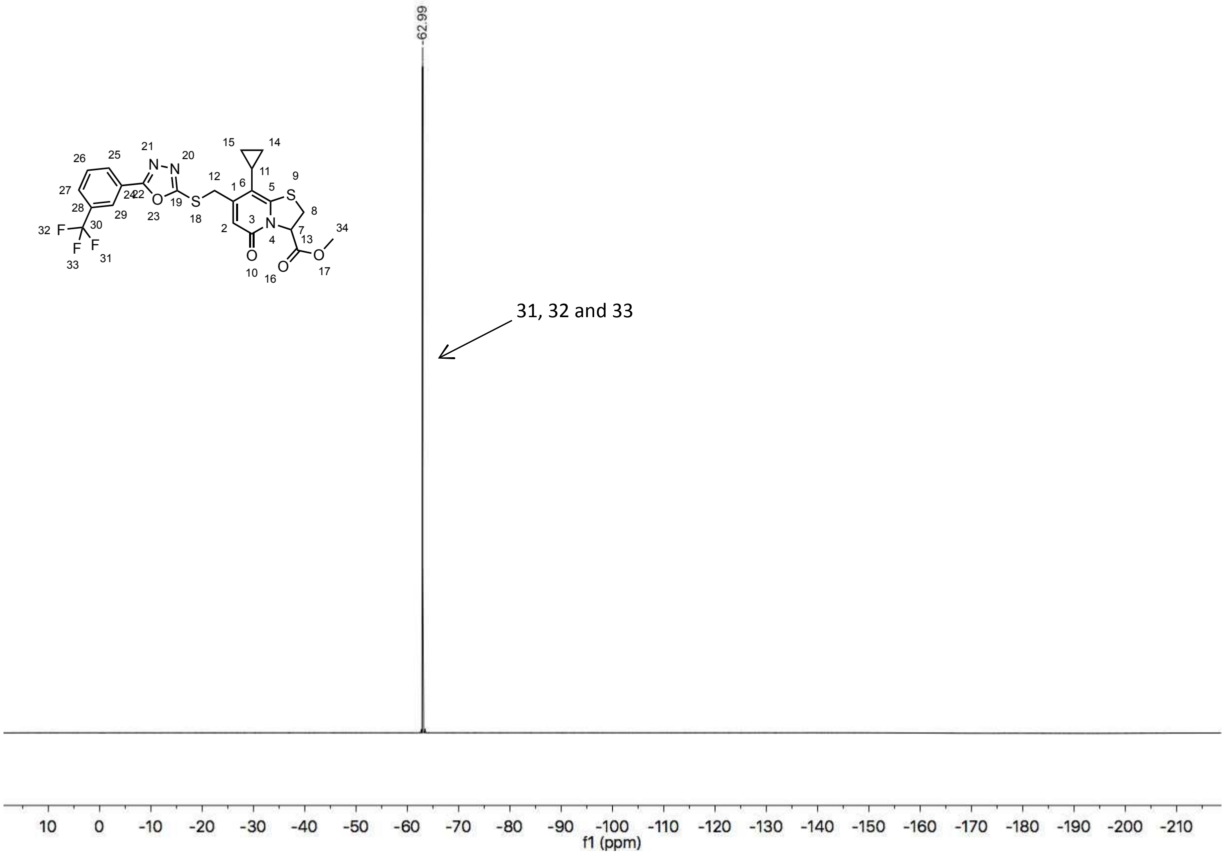
-62.99
31, 32 and 33
10 0 -10 -20 -30 -40 -50 -60 -70 -80 -90 -100 -110 -120 -130 -140 -150 -160 -170 -180 -190 -200 -210
f1 (ppm)

^{1}H NMR of **7c**

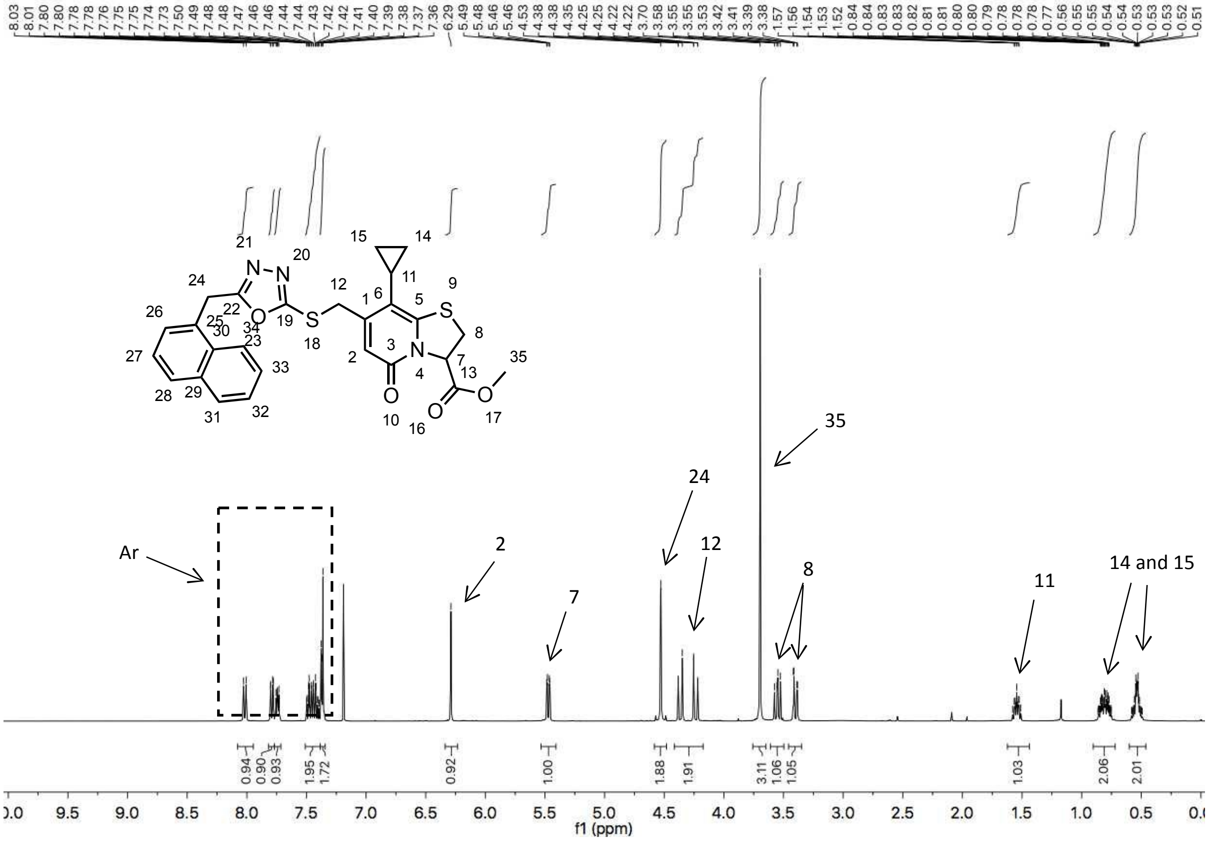

^{13}C NMR of **7c**

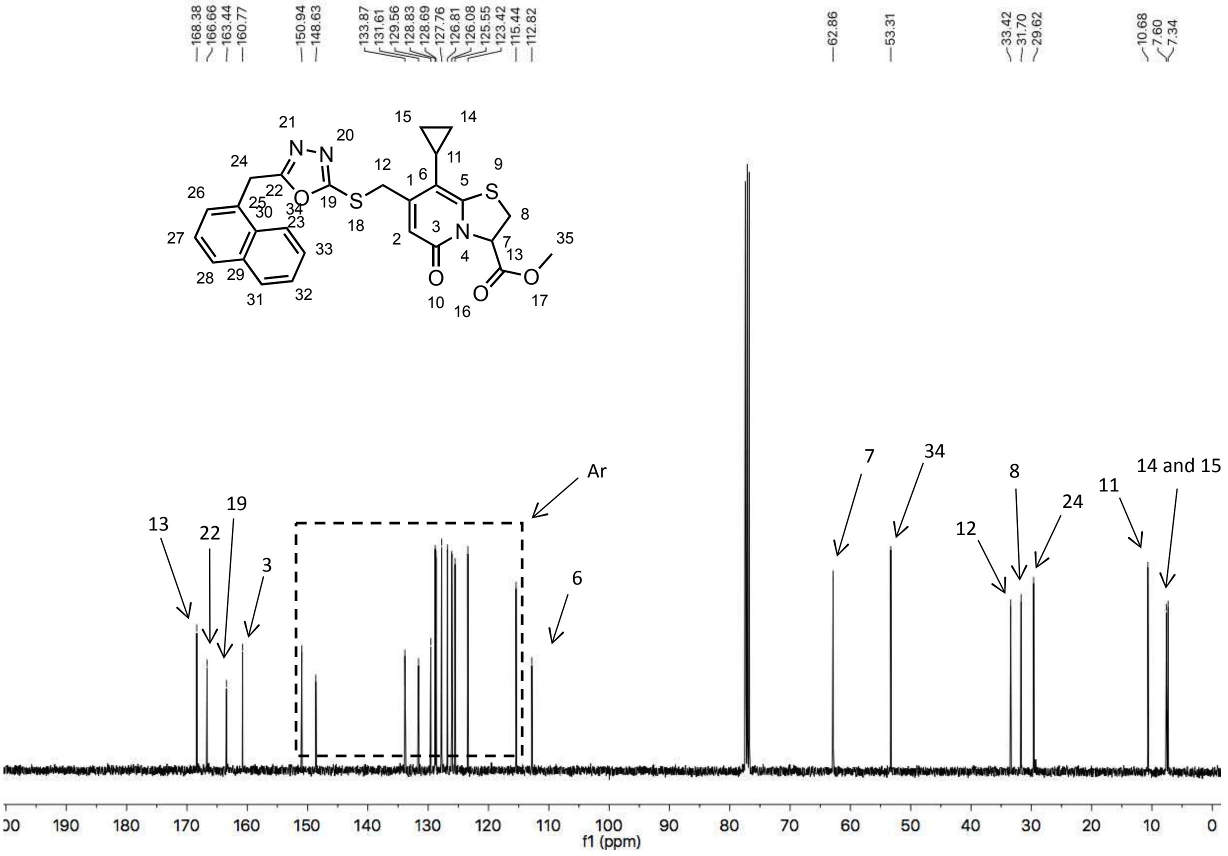

^{1}H NMR of **7d**

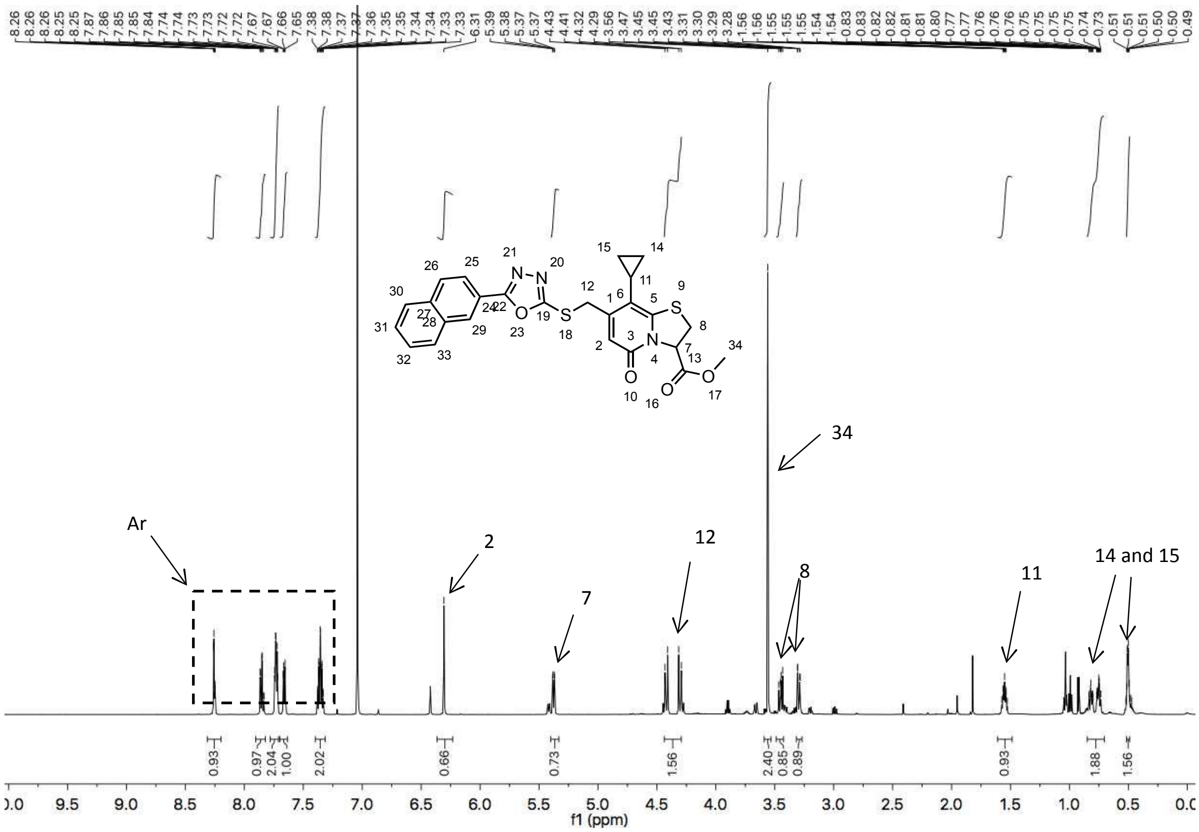

^{13}C NMR of **7d**

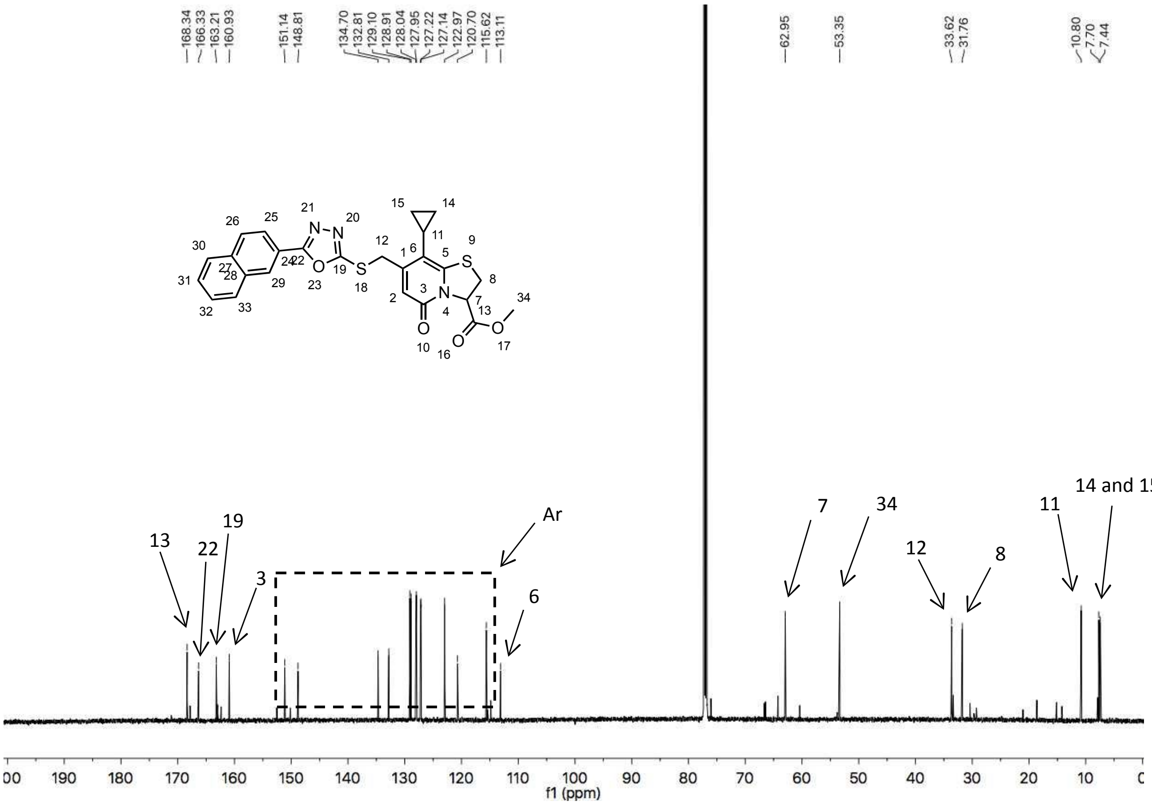

^{1}H NMR of **7e**

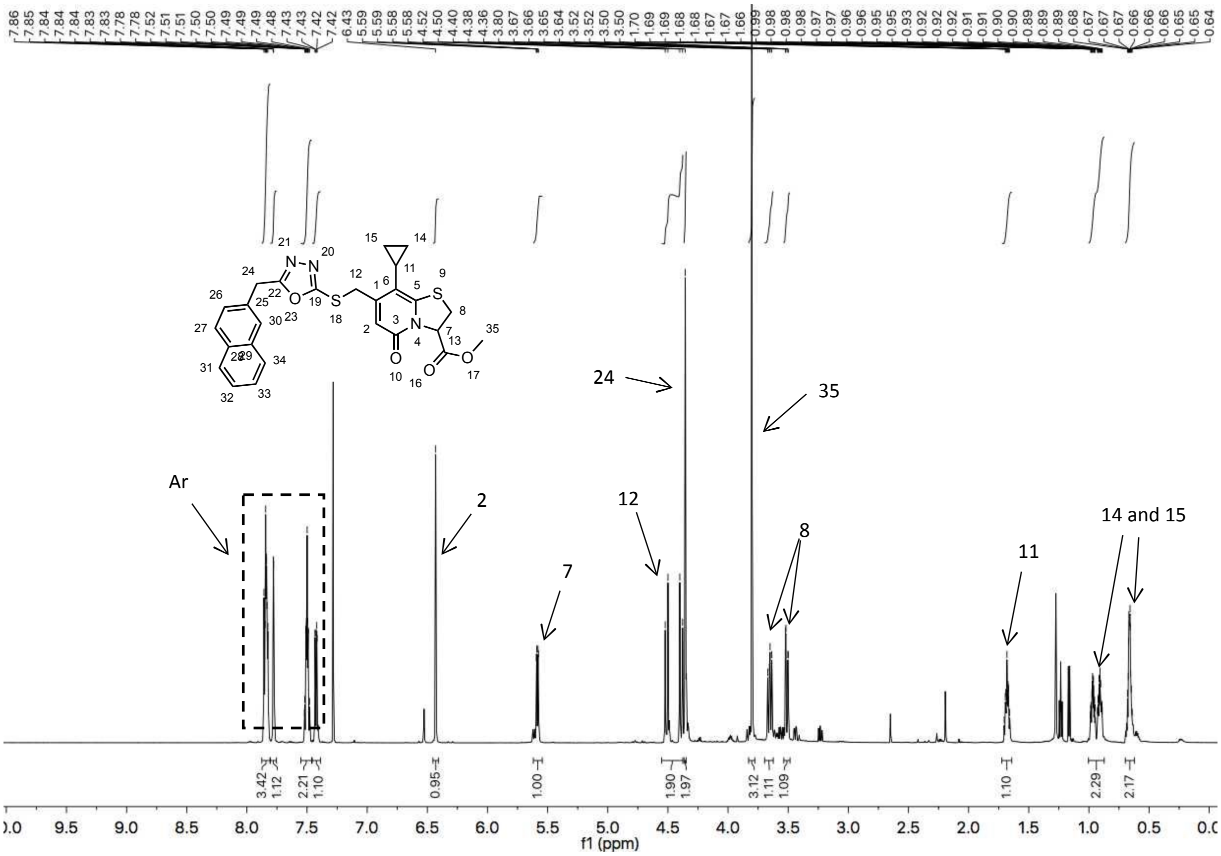

^{13}C NMR of **7e**

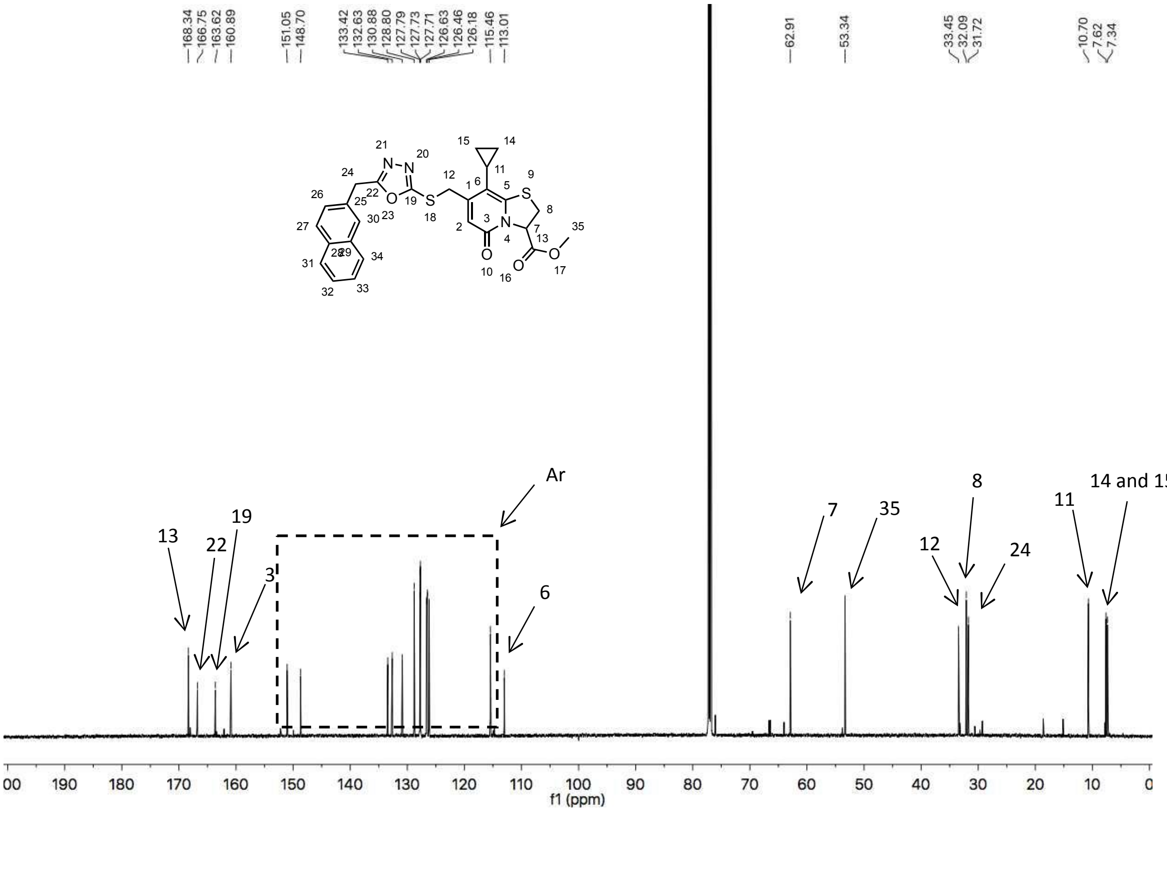

^{1}H NMR of **7f**

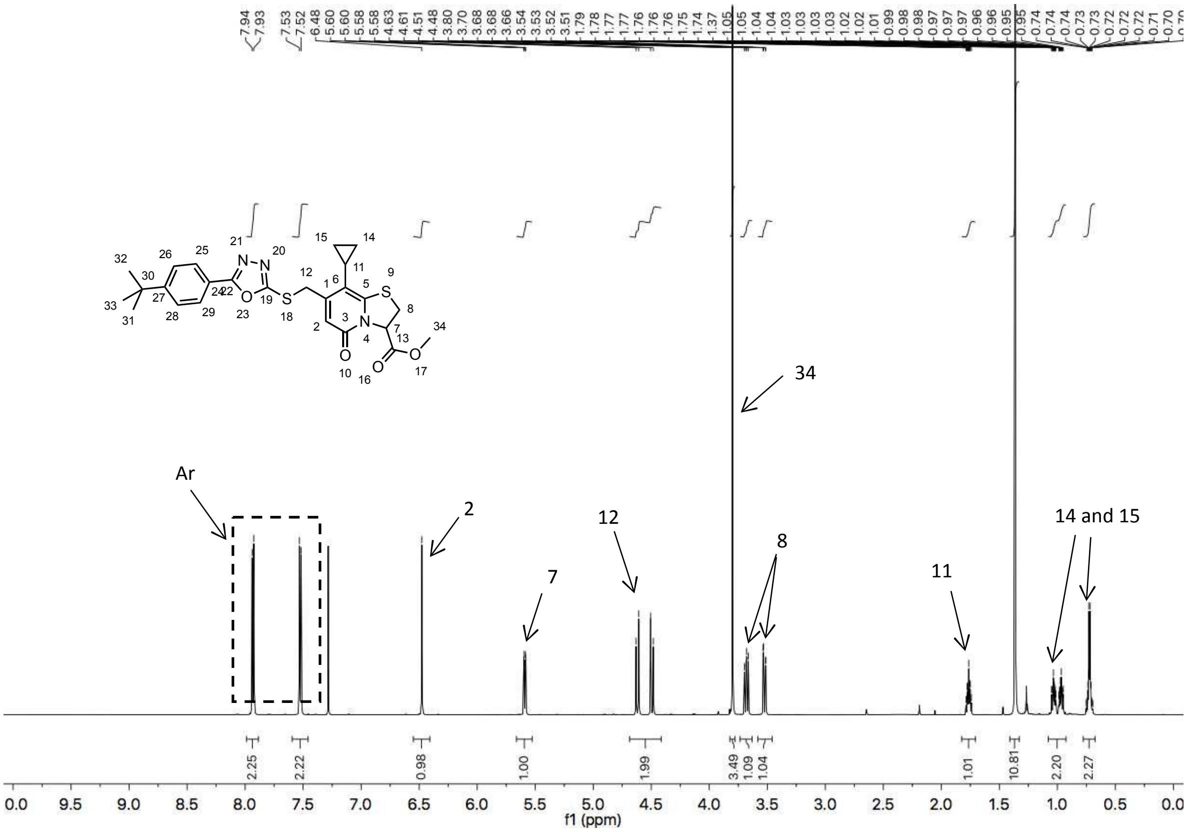

^{13}C NMR of **7f**

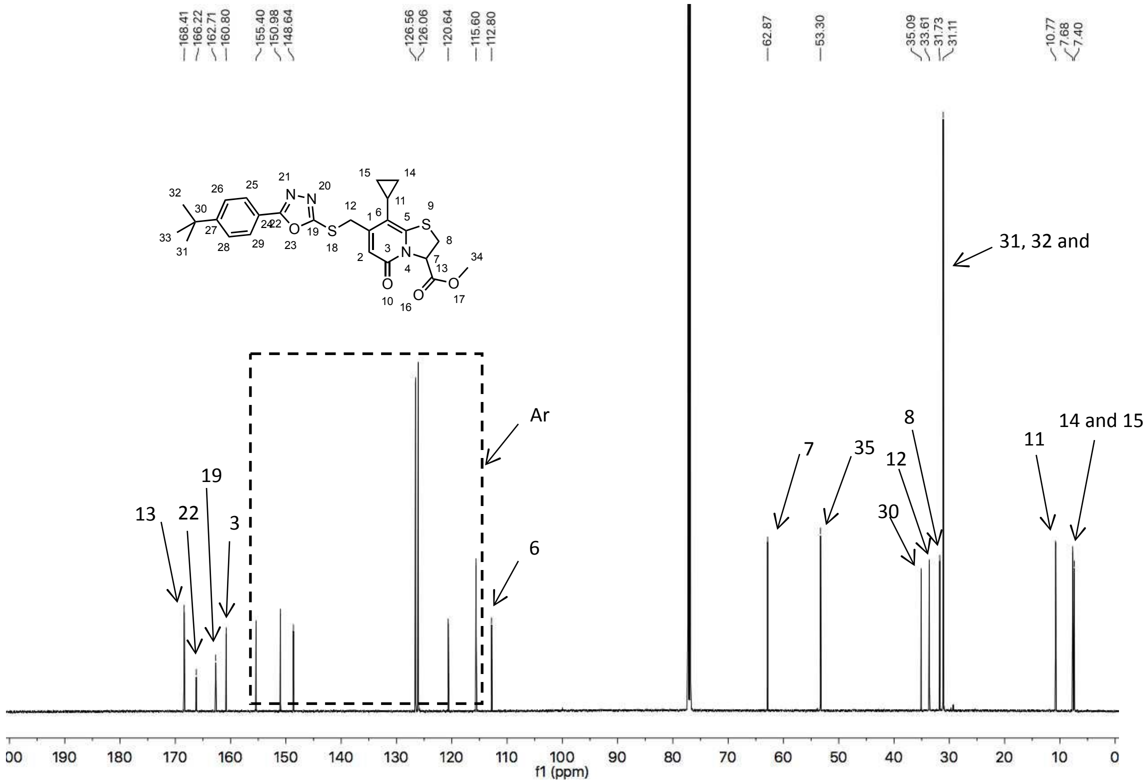

^{1}H NMR of **7g**

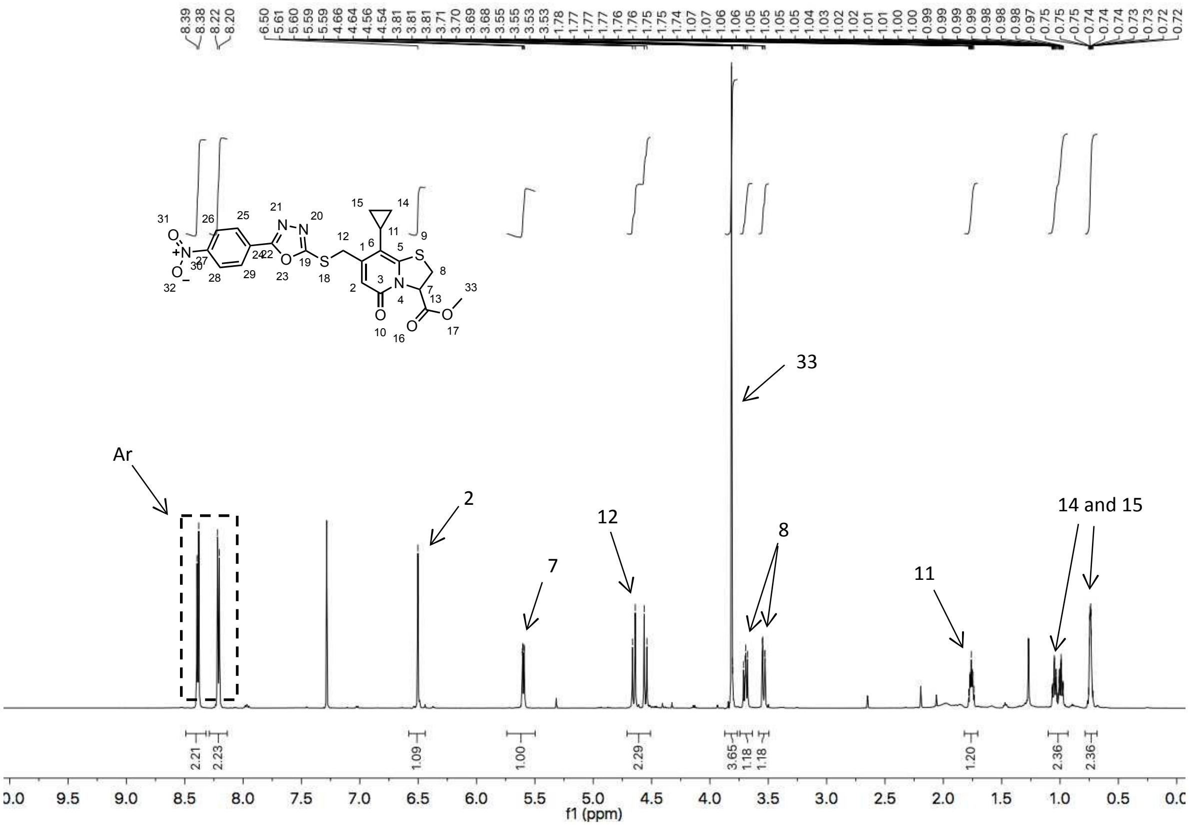

^{13}C NMR of **7g**

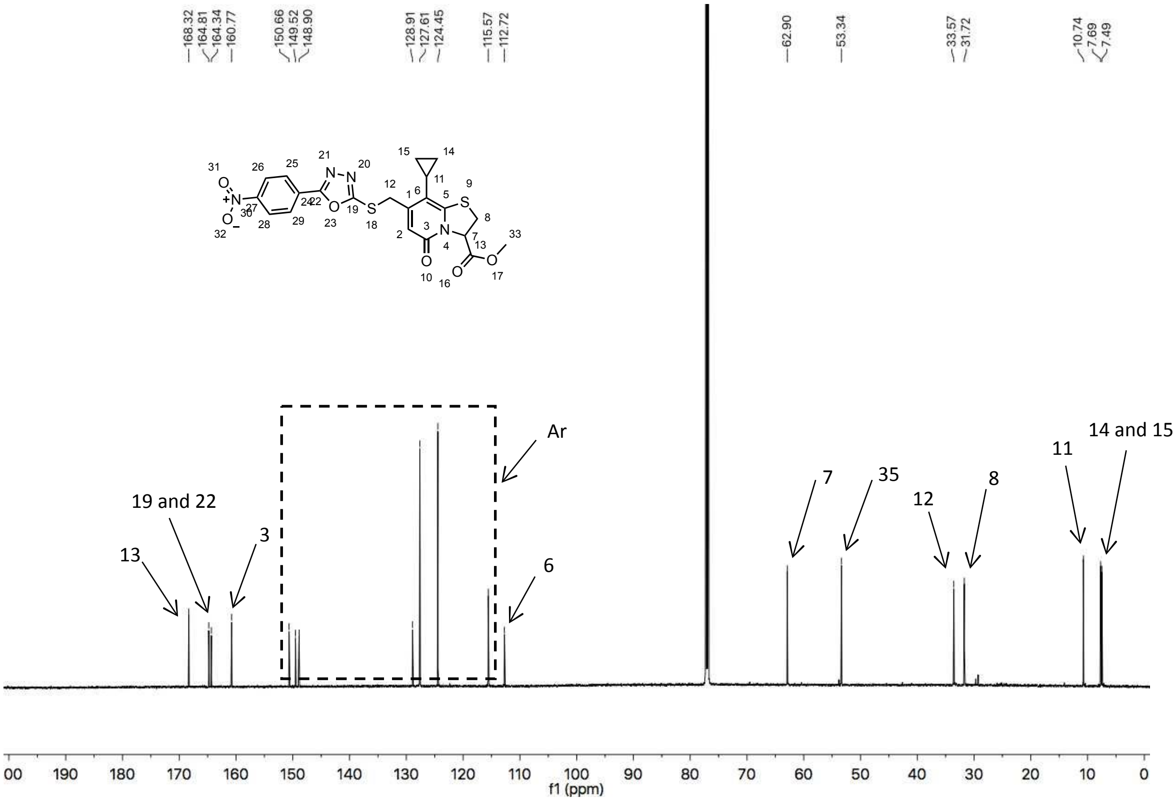

^{1}H NMR of **7h**

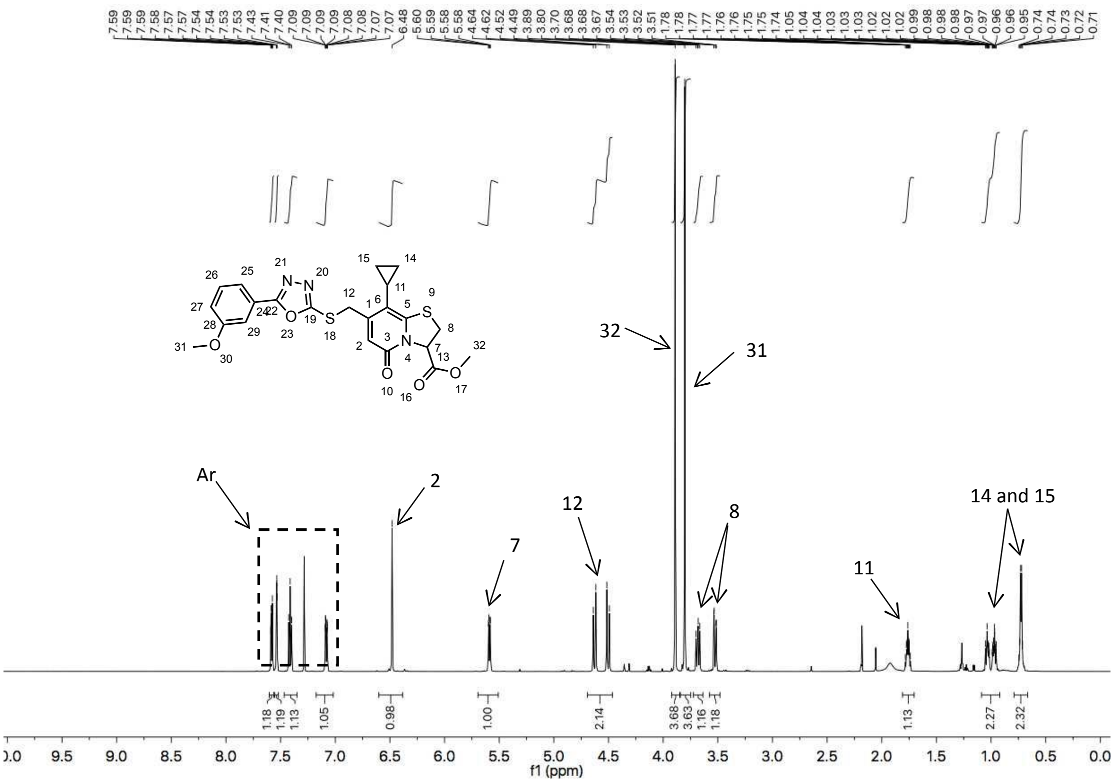

^{13}C NMR of **7h**

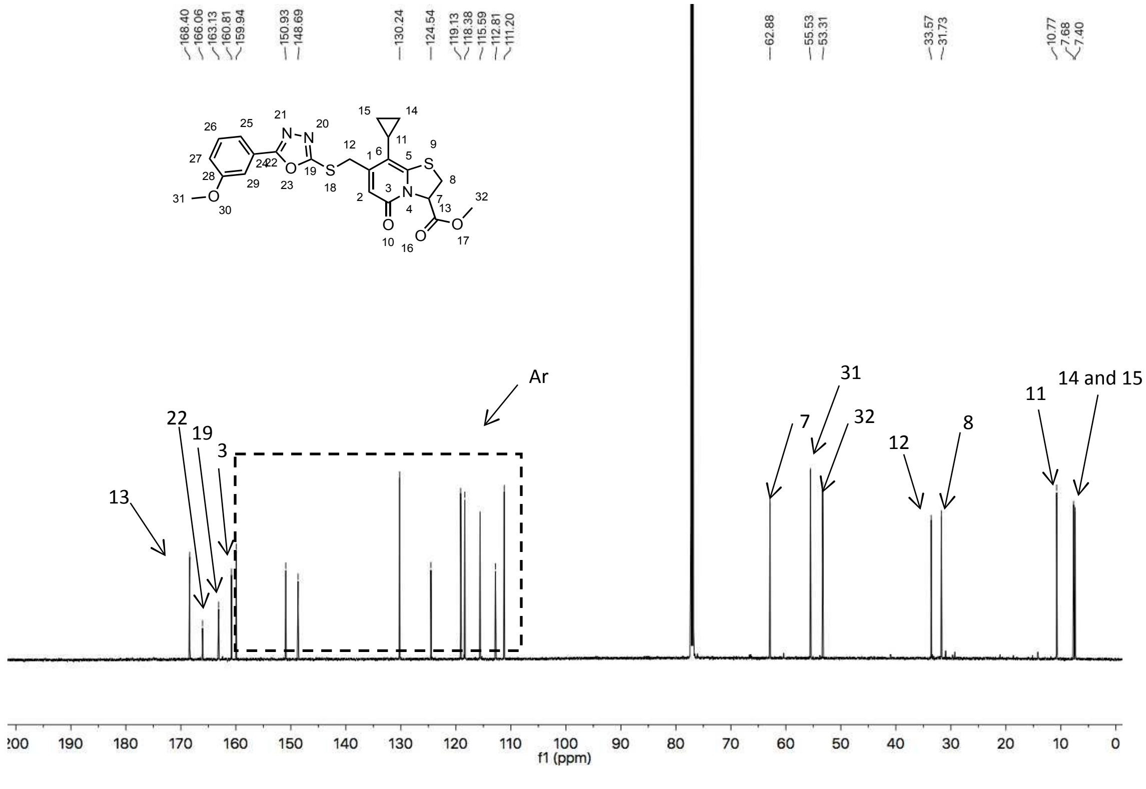

^{1}H NMR of **7i**

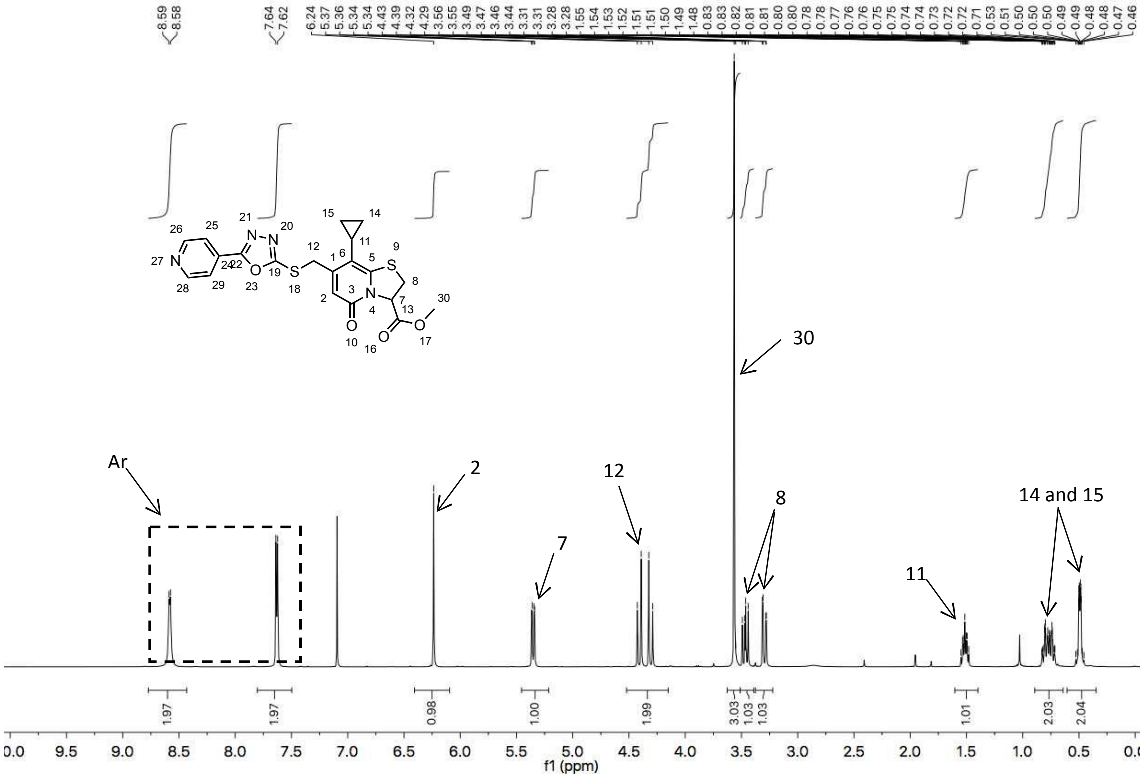

^{13}C NMR of **7i**

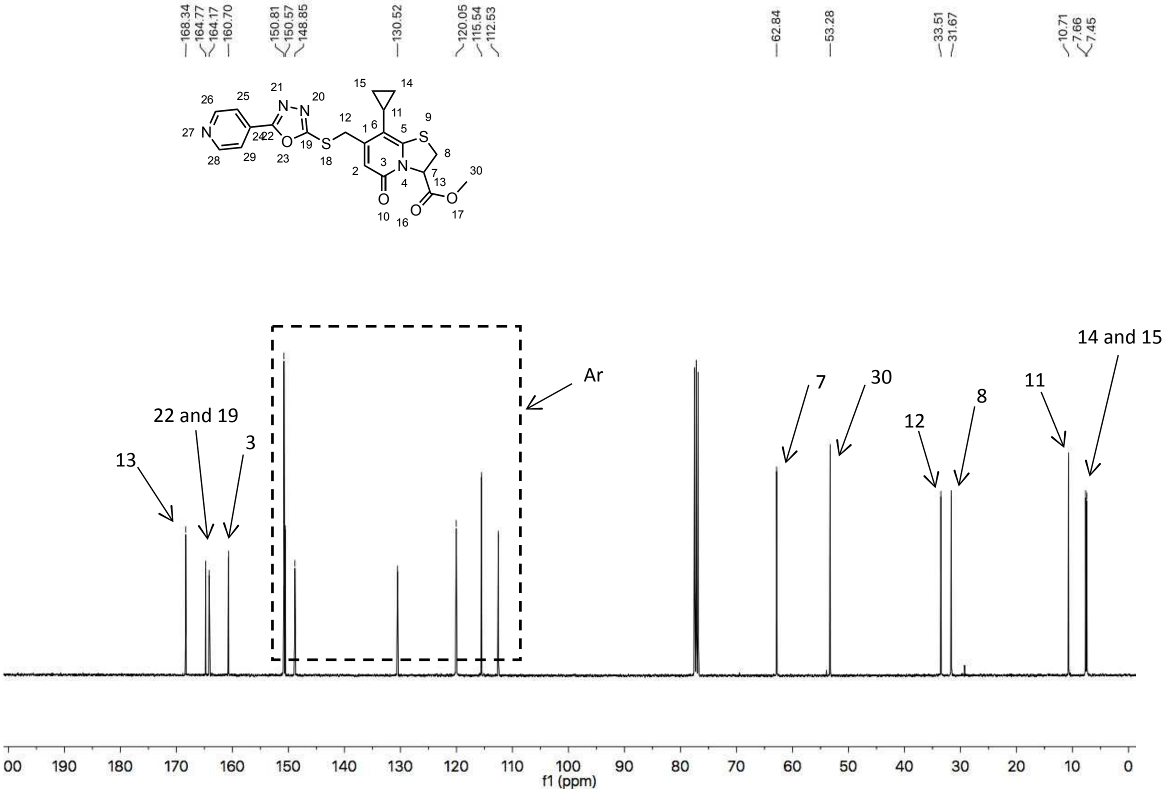

^{1}H NMR of **7j**

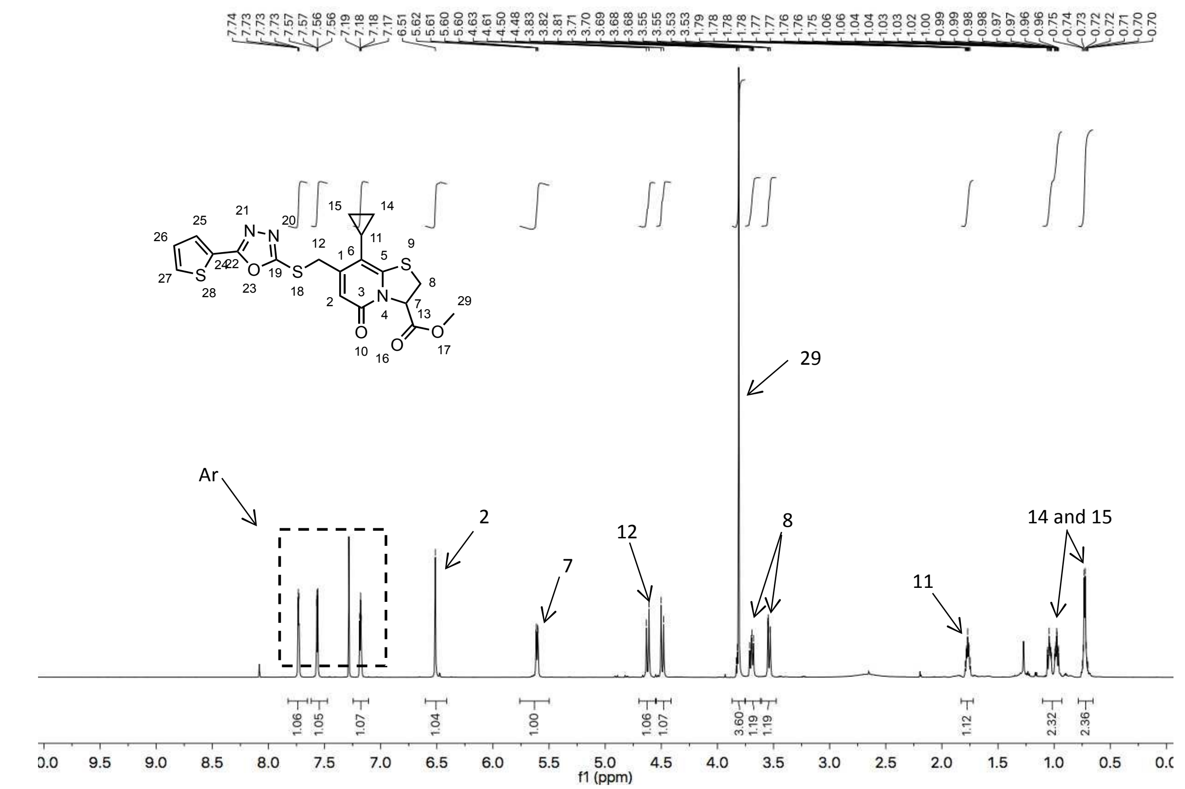

^{13}C NMR of **7j**

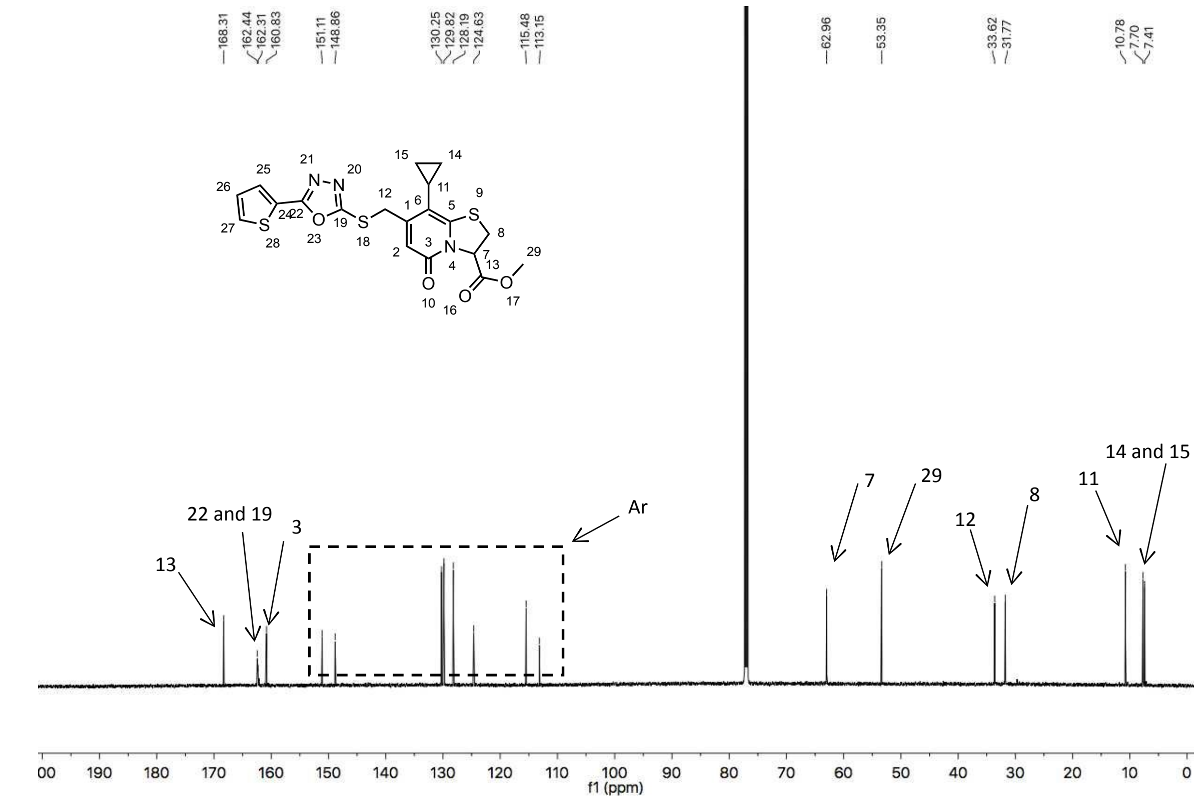

^{1}H NMR of **7k**

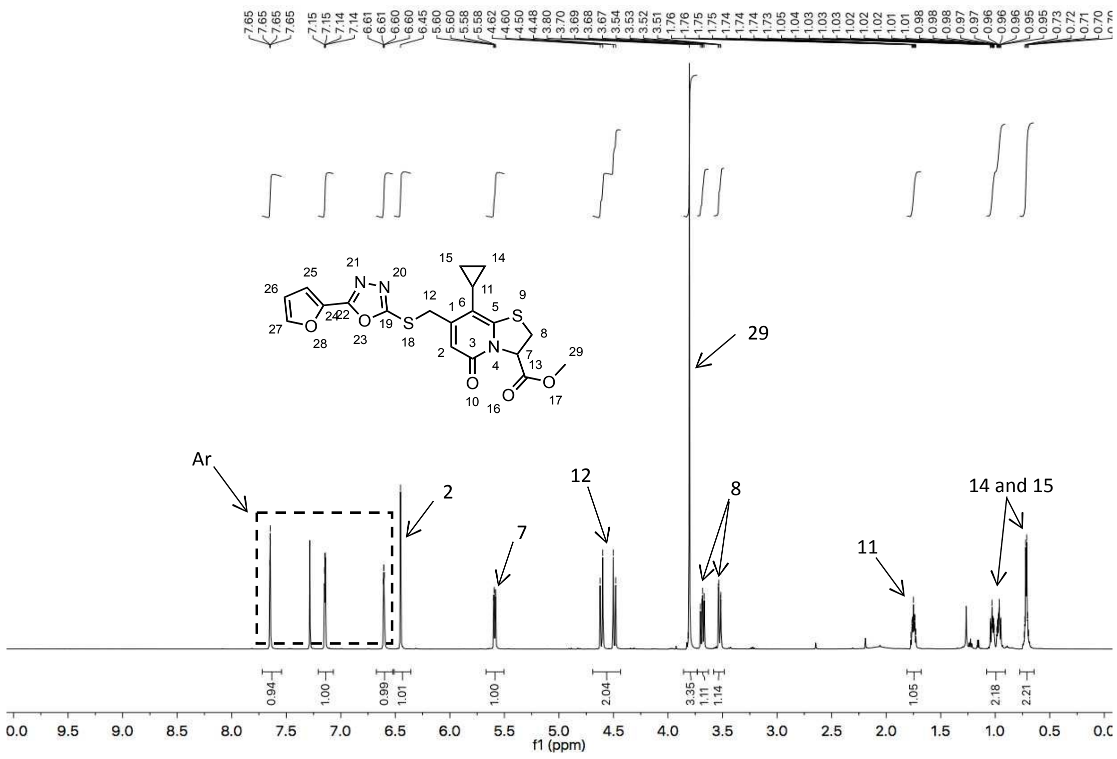

^{13}C NMR of **7k**

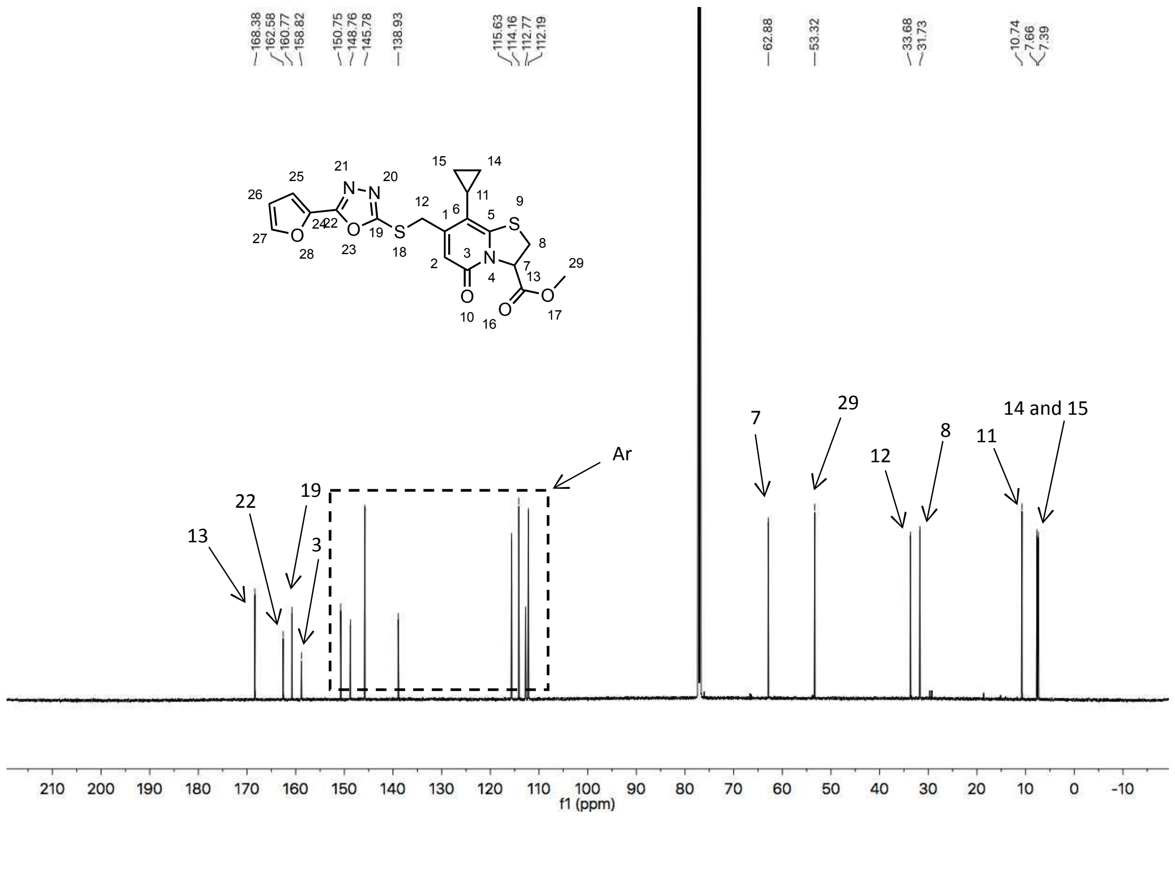

^1H NMR of **7l**

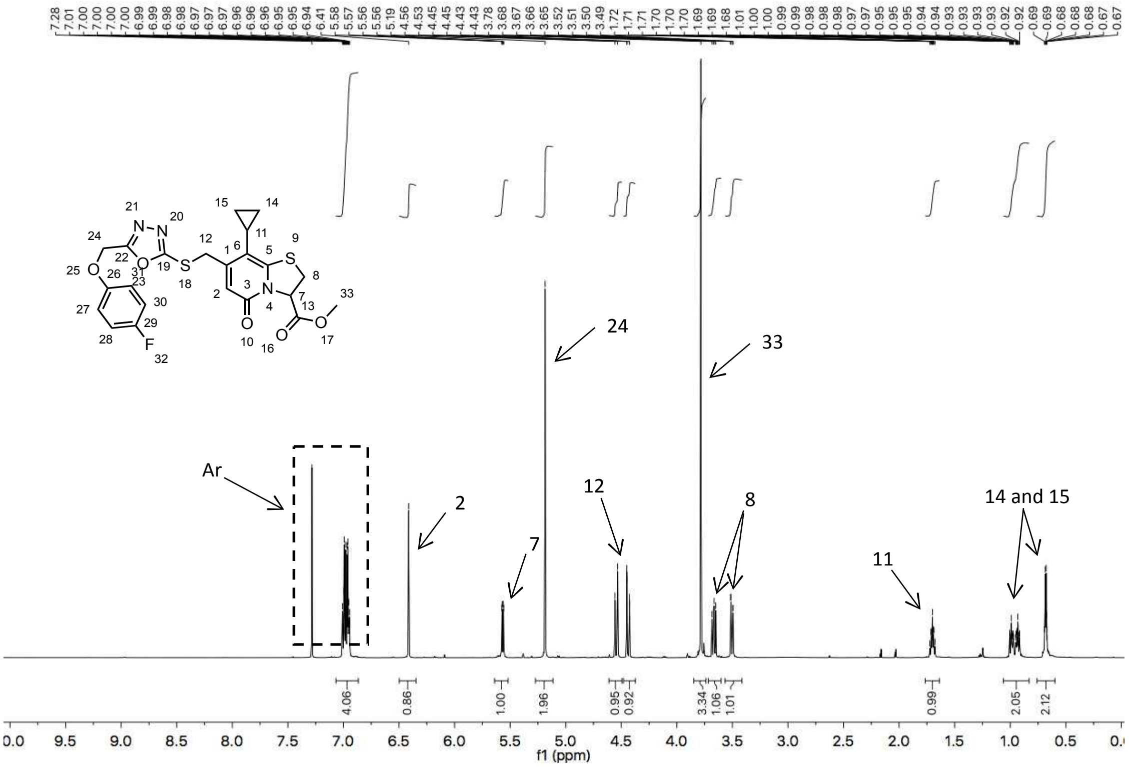

^{13}C NMR of **7l**

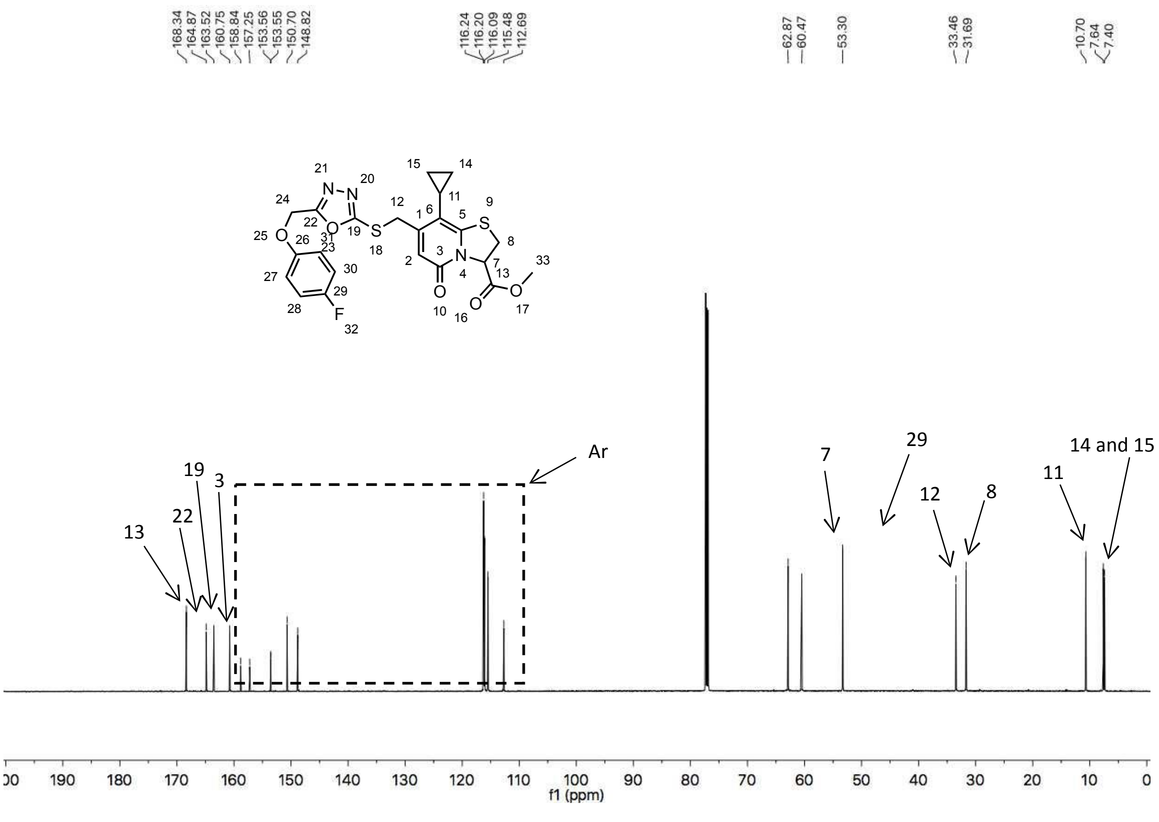

^{19}F NMR of **7l**

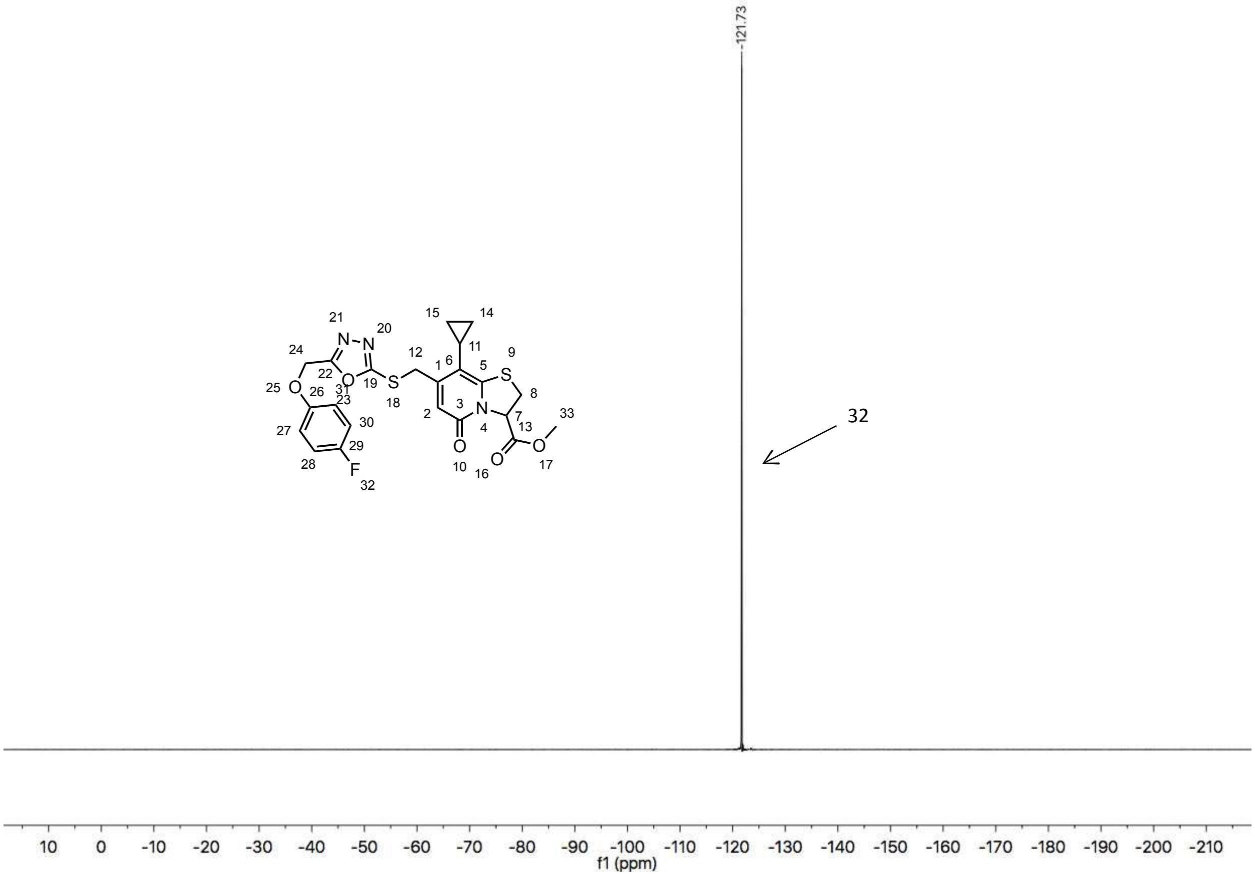
-121.73
21
20
15
14
24
12
11
9
1
6
22
5
25
26
31
19
8
23
18
3
2
30
4
7
13
33
27
29
28
10
16
17
32
32
10
0
-10
-20
-30
-40
-50
-60
-70
-80
-90
-100
-110
-120
-130
-140
-150
-160
-170
-180
-190
-200
-210
f1 (ppm)

^{1}H NMR of **8a**

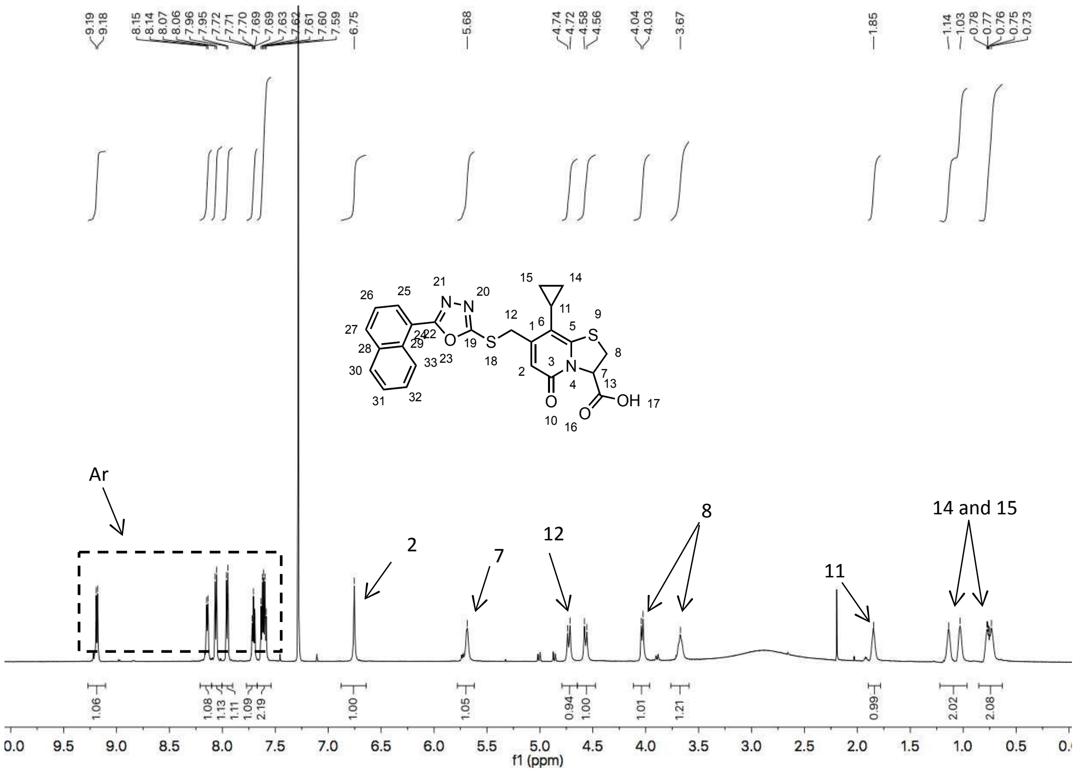

^{13}C NMR of **8a**

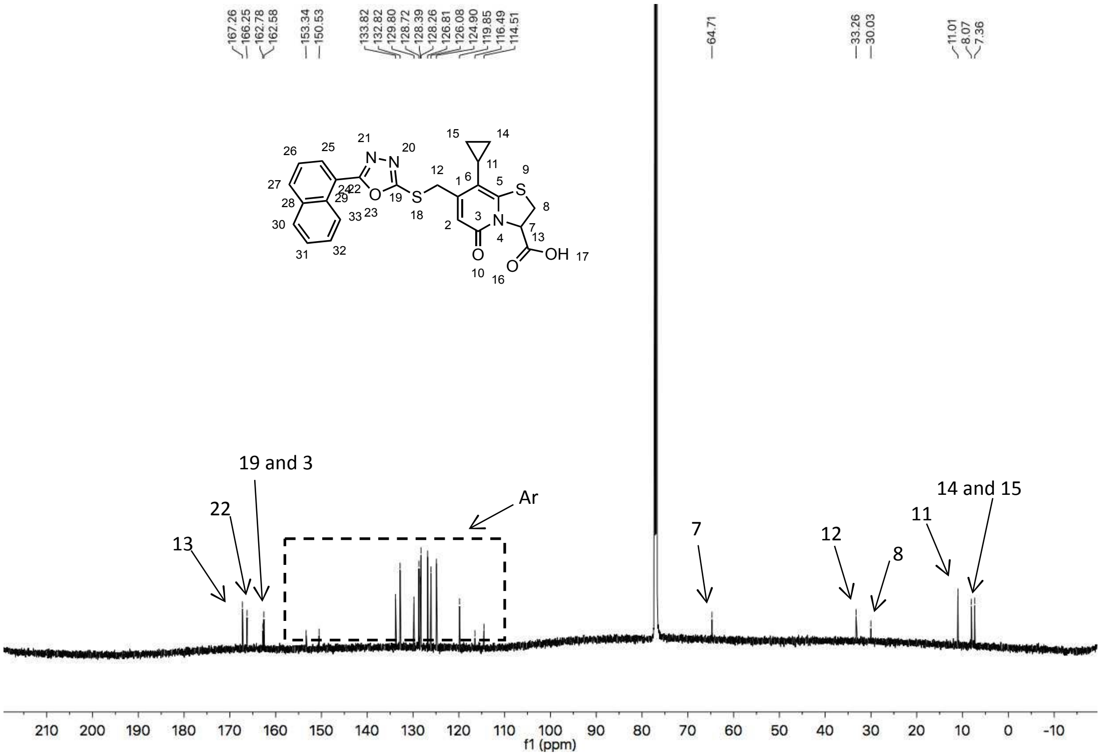

^{1}H NMR of **8b**

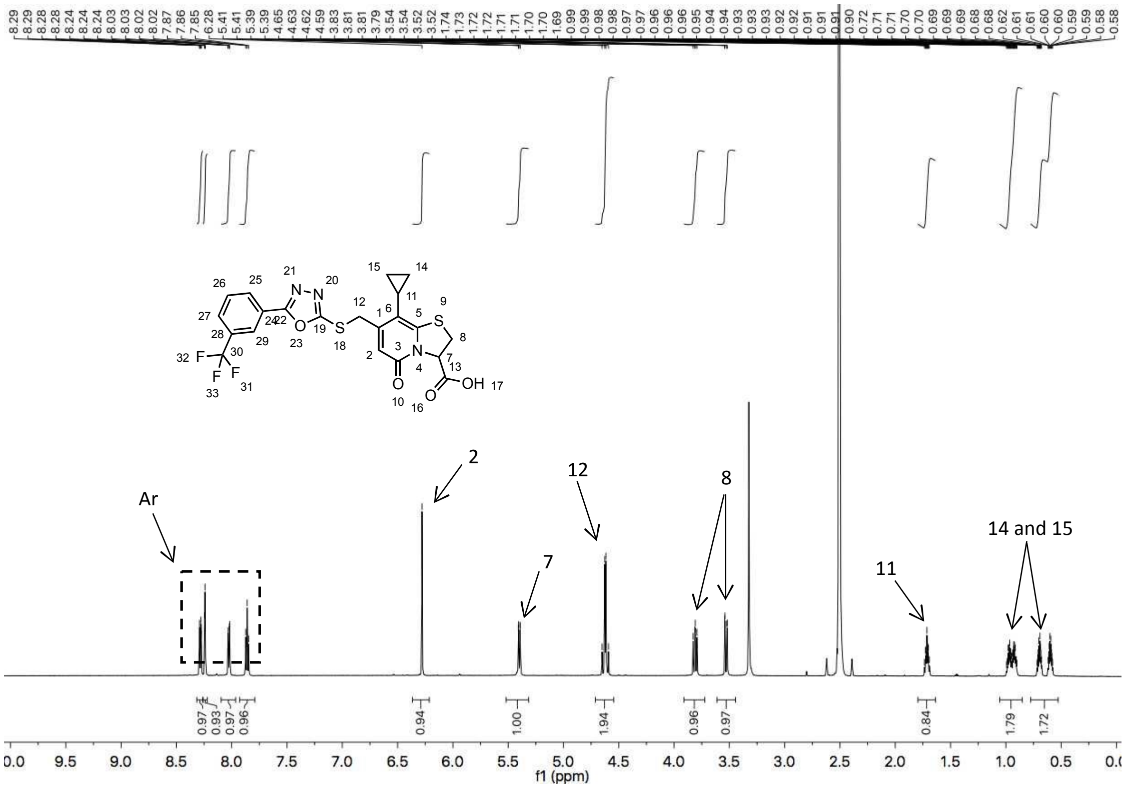

^{13}C NMR of **8b**

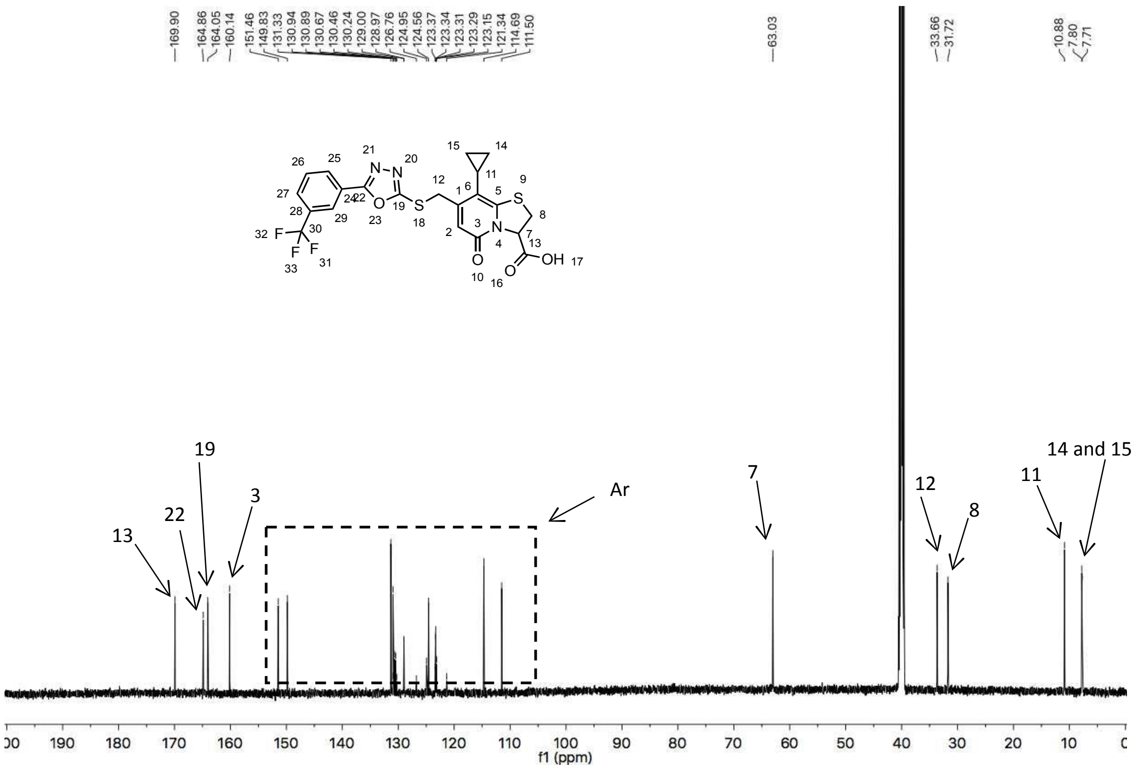

^{19}F NMR of **8b**

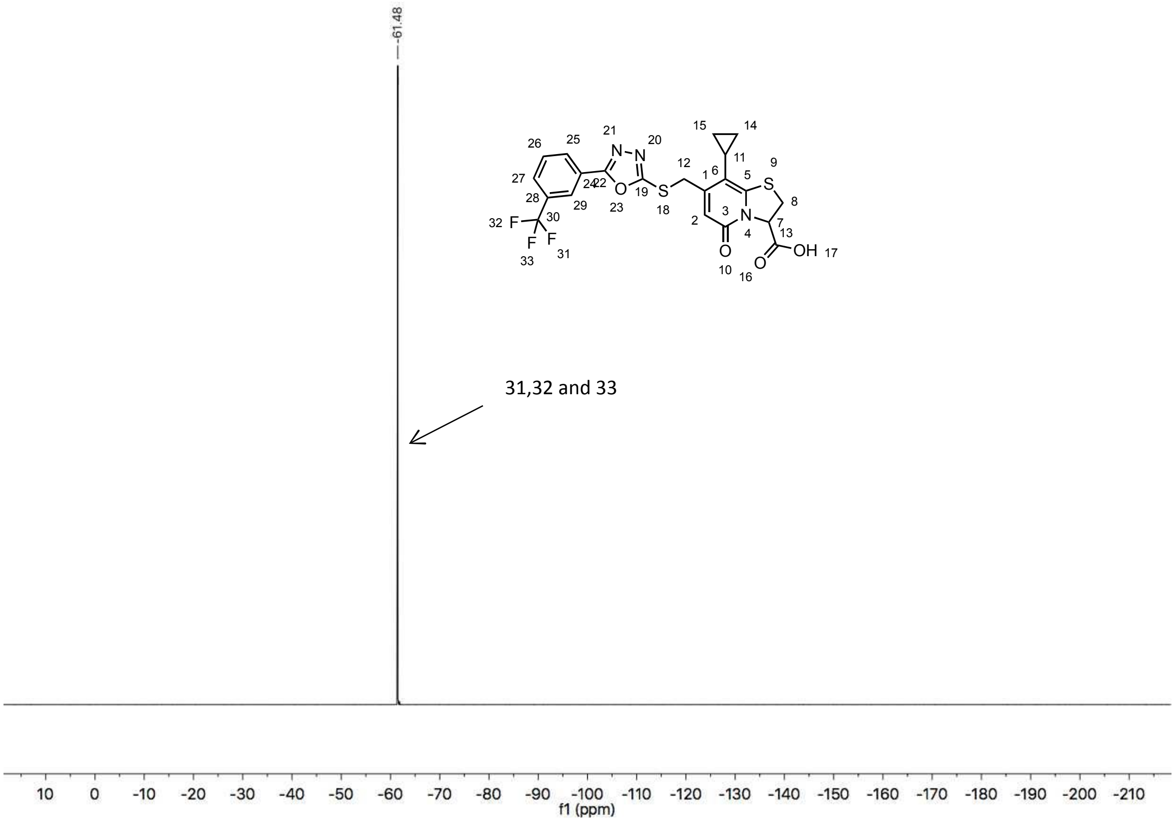

-61.48
31,32 and 33
10 0 -10 -20 -30 -40 -50 -60 -70 -80 -90 -100 -110 -120 -130 -140 -150 -160 -170 -180 -190 -200 -210
f1 (ppm)

^{1}H NMR of **8c**

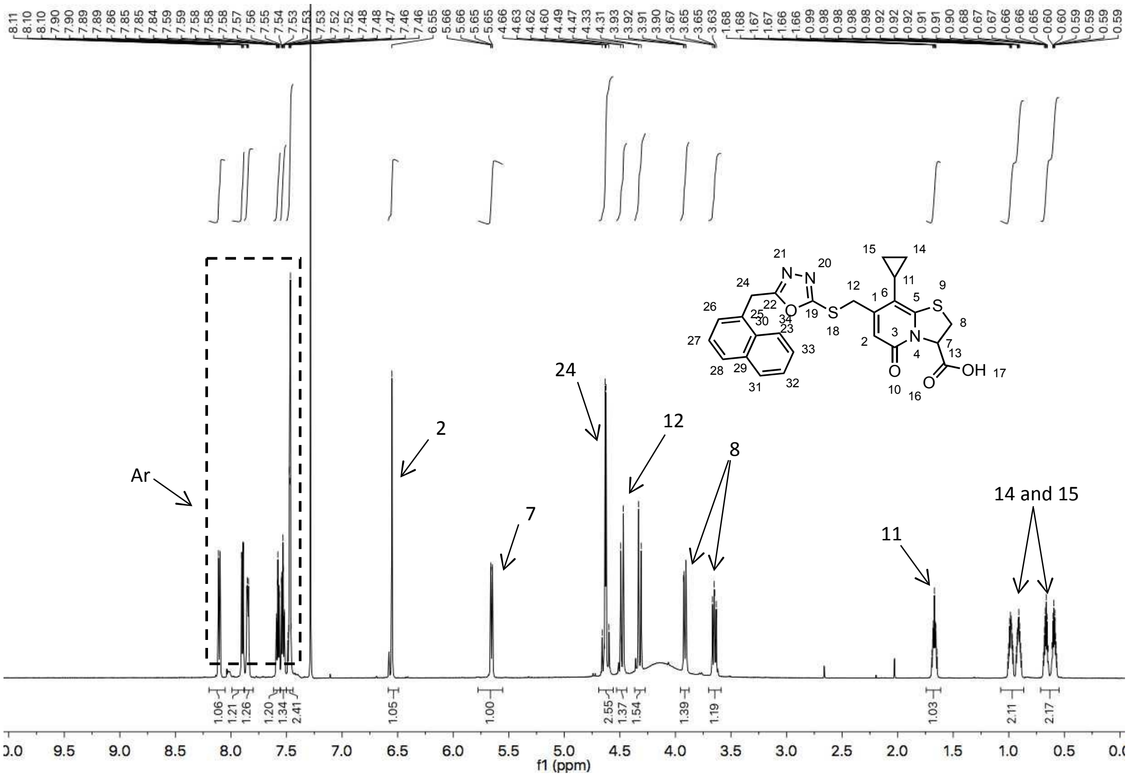

^{13}C NMR of **8c**

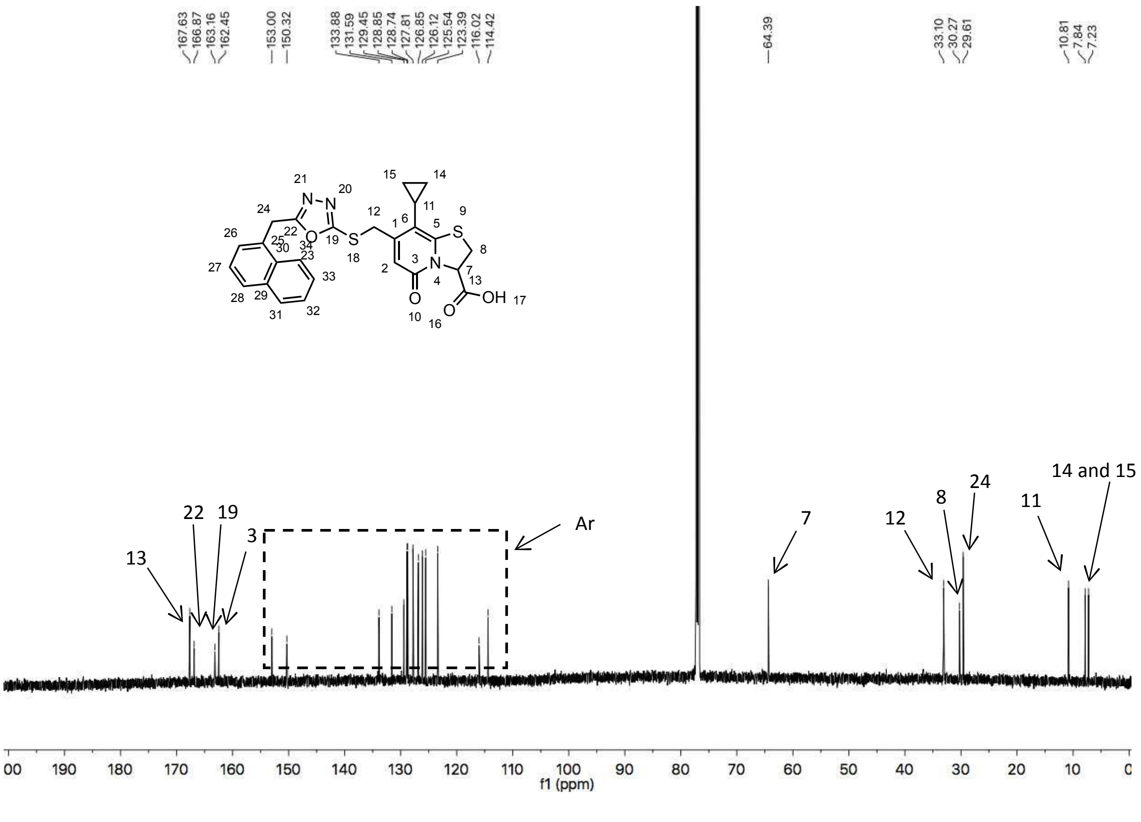

^{1}H NMR of **8d**

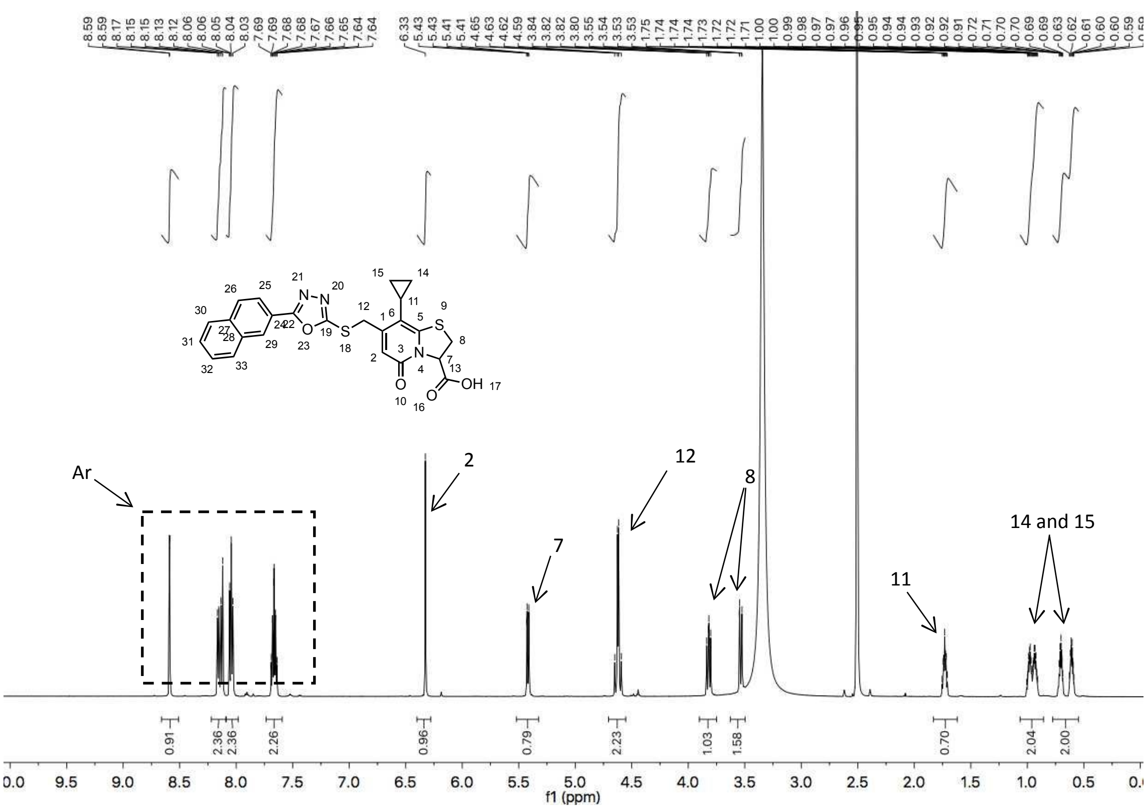

^{13}C NMR of **8d**

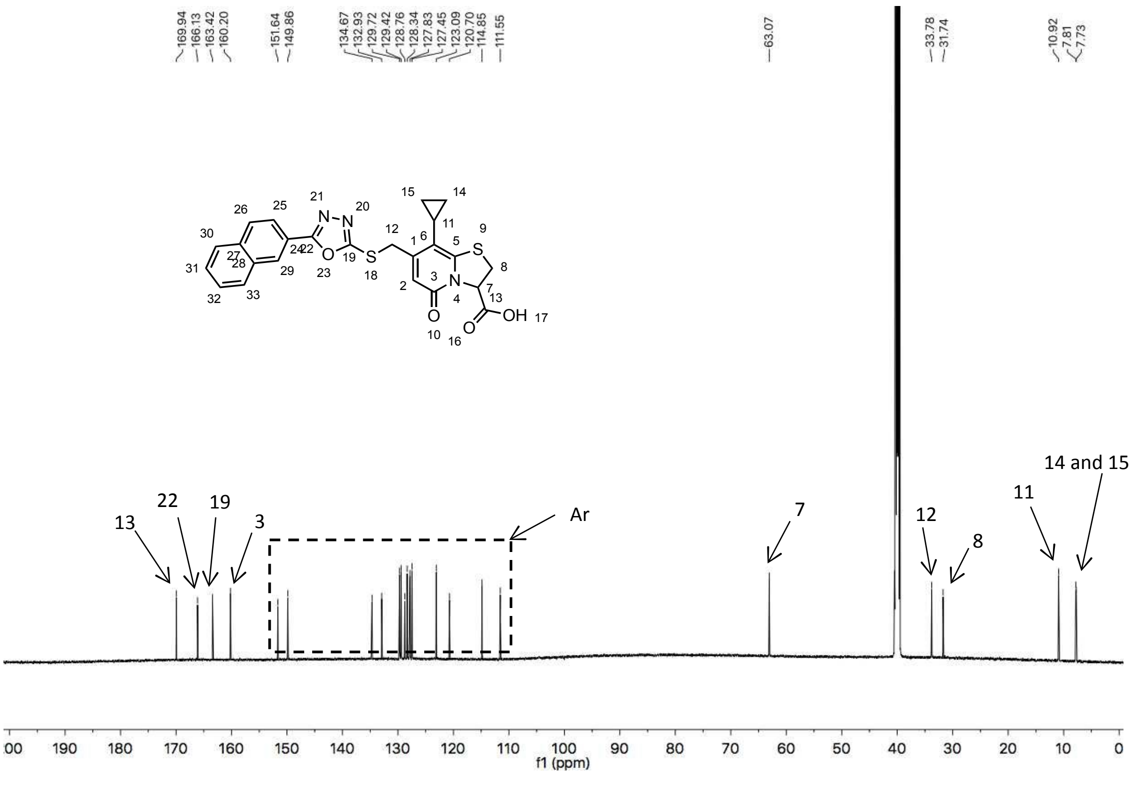

^{1}H NMR of **8e**

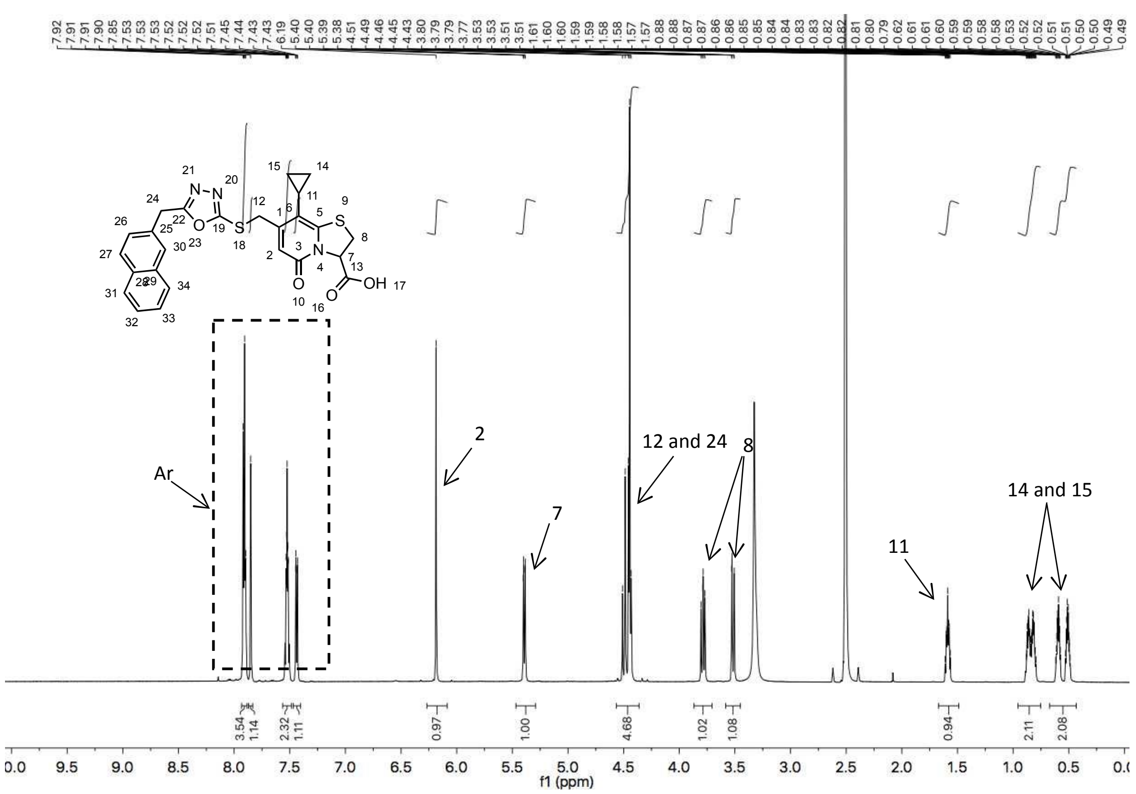

^{13}C NMR of **8e**

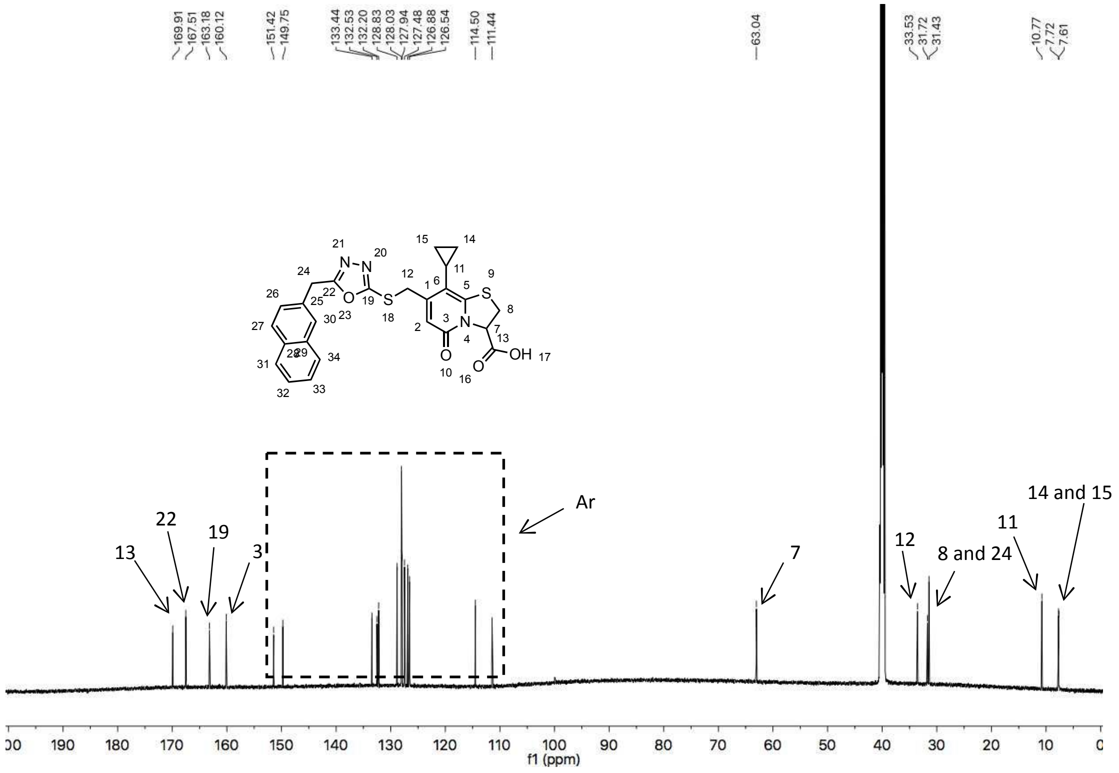

^{1}H NMR of **8f**

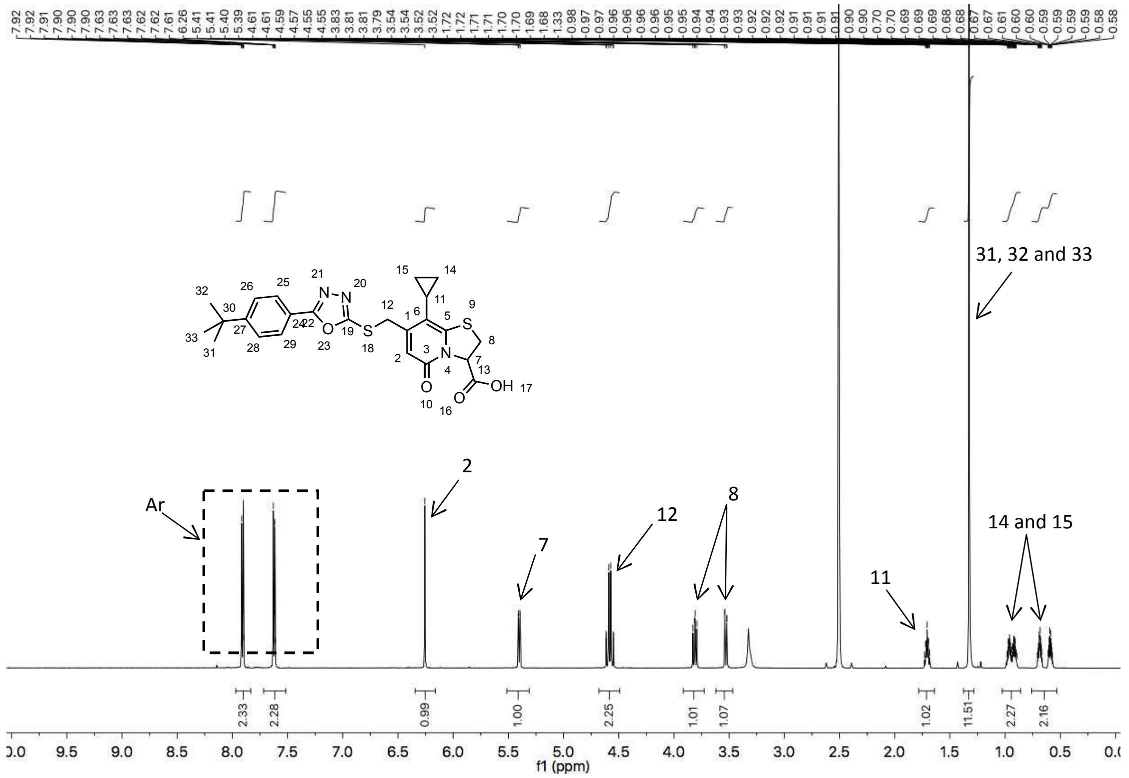

^{13}C NMR of **8f**

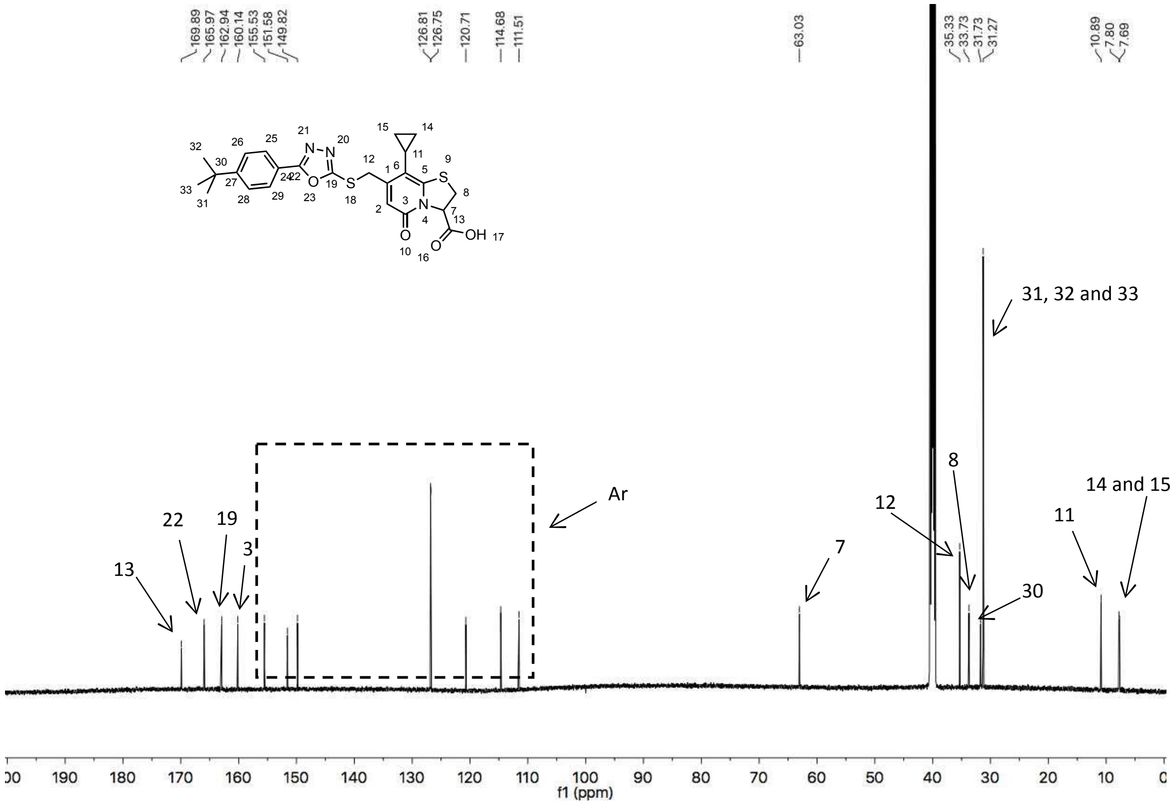

^{1}H NMR of **8g**

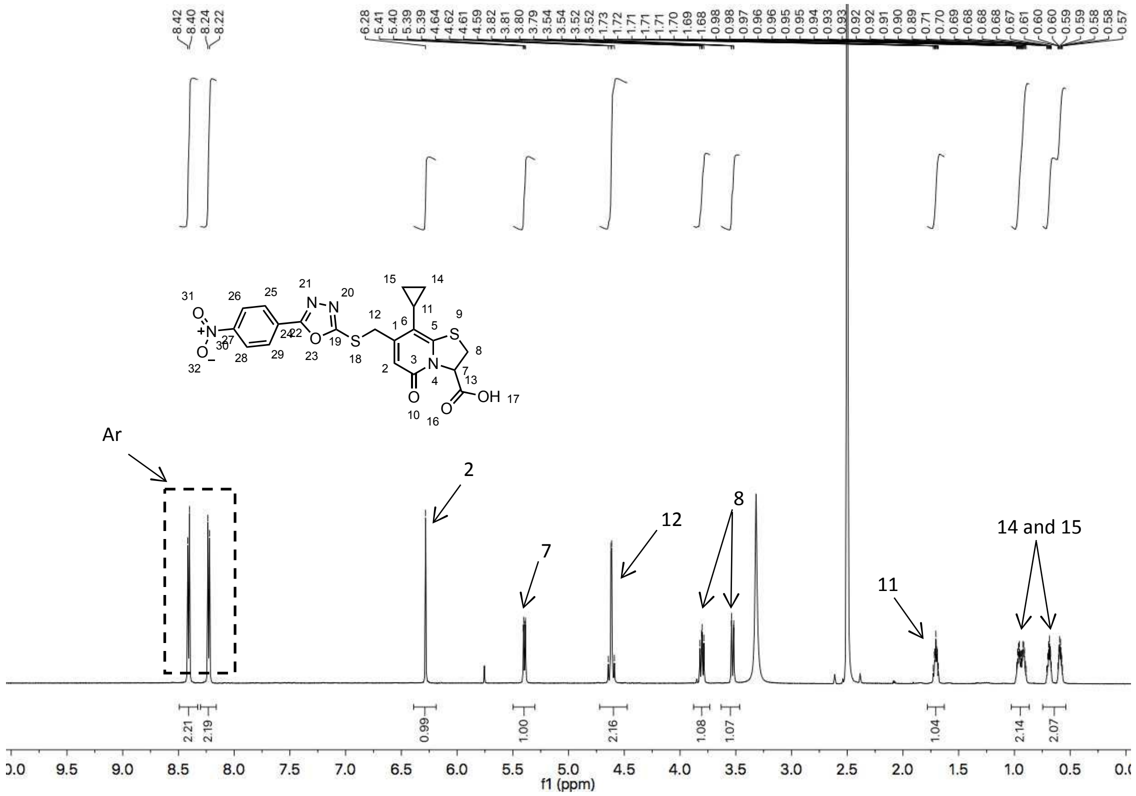

^{13}C NMR of **8g**

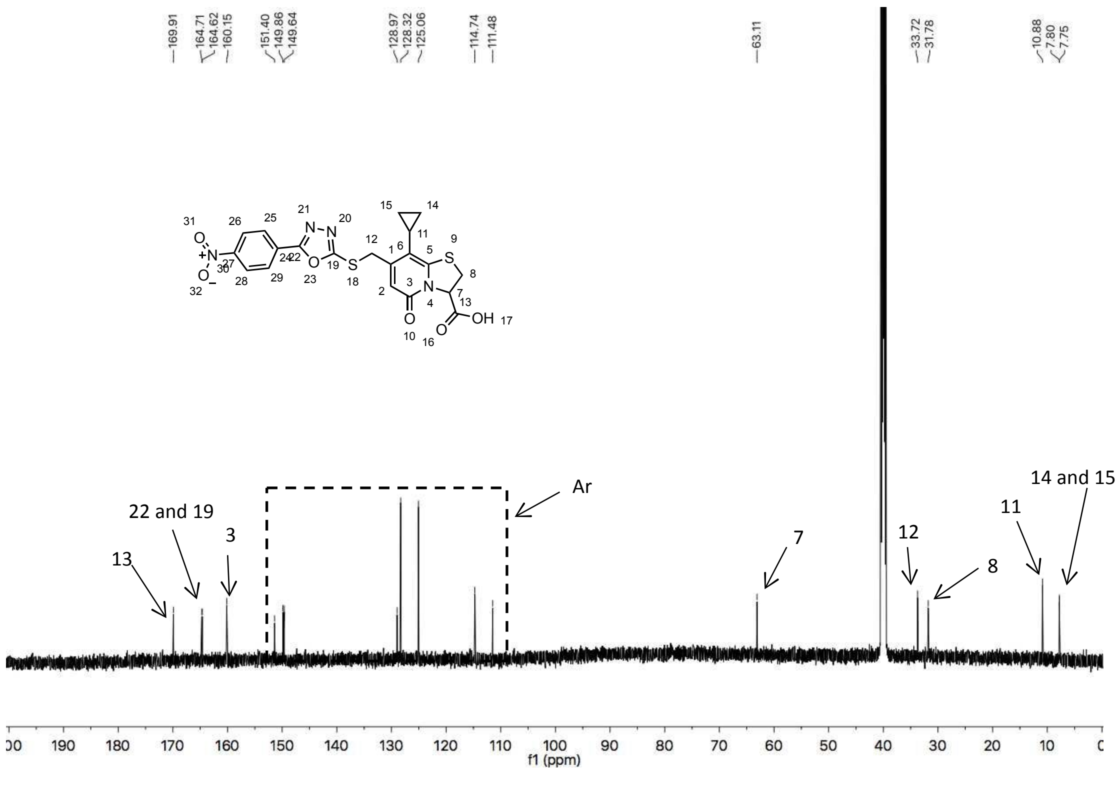

^{1}H NMR of **8h**

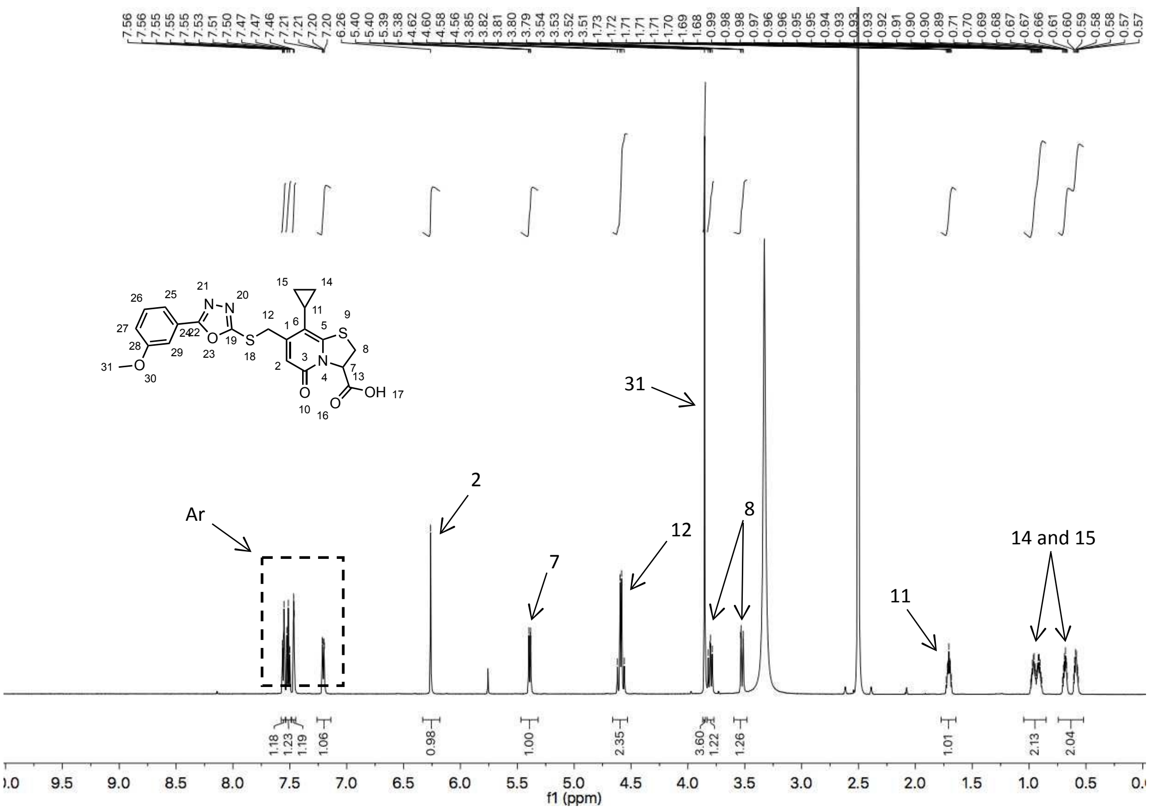

^{13}C NMR of **8h**

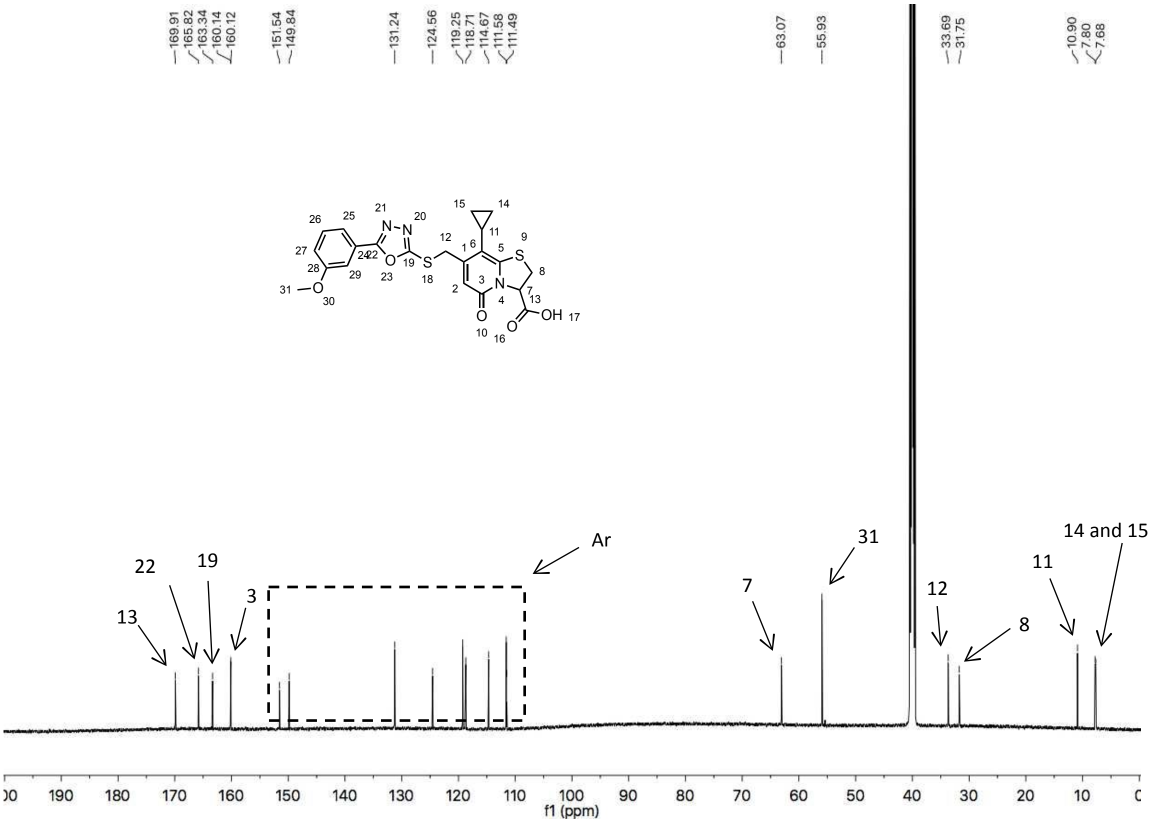

^{1}H NMR of **8i**

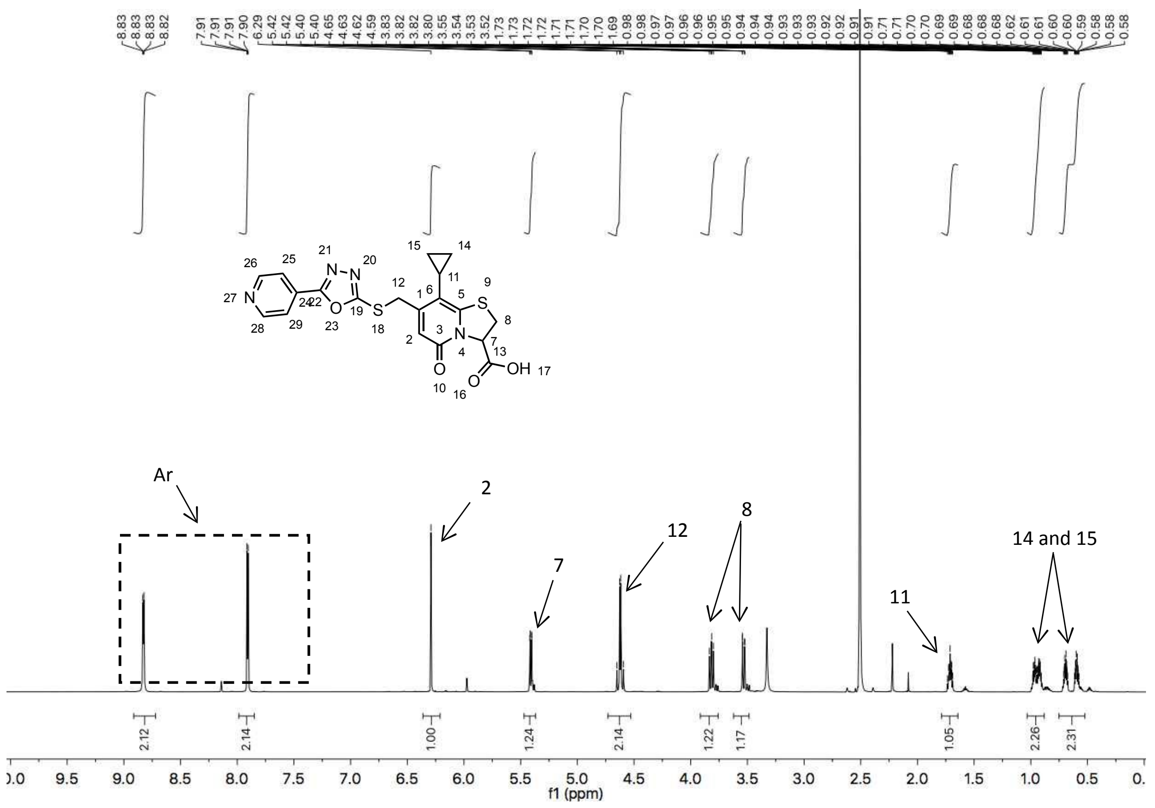

^{13}C NMR of **8i**

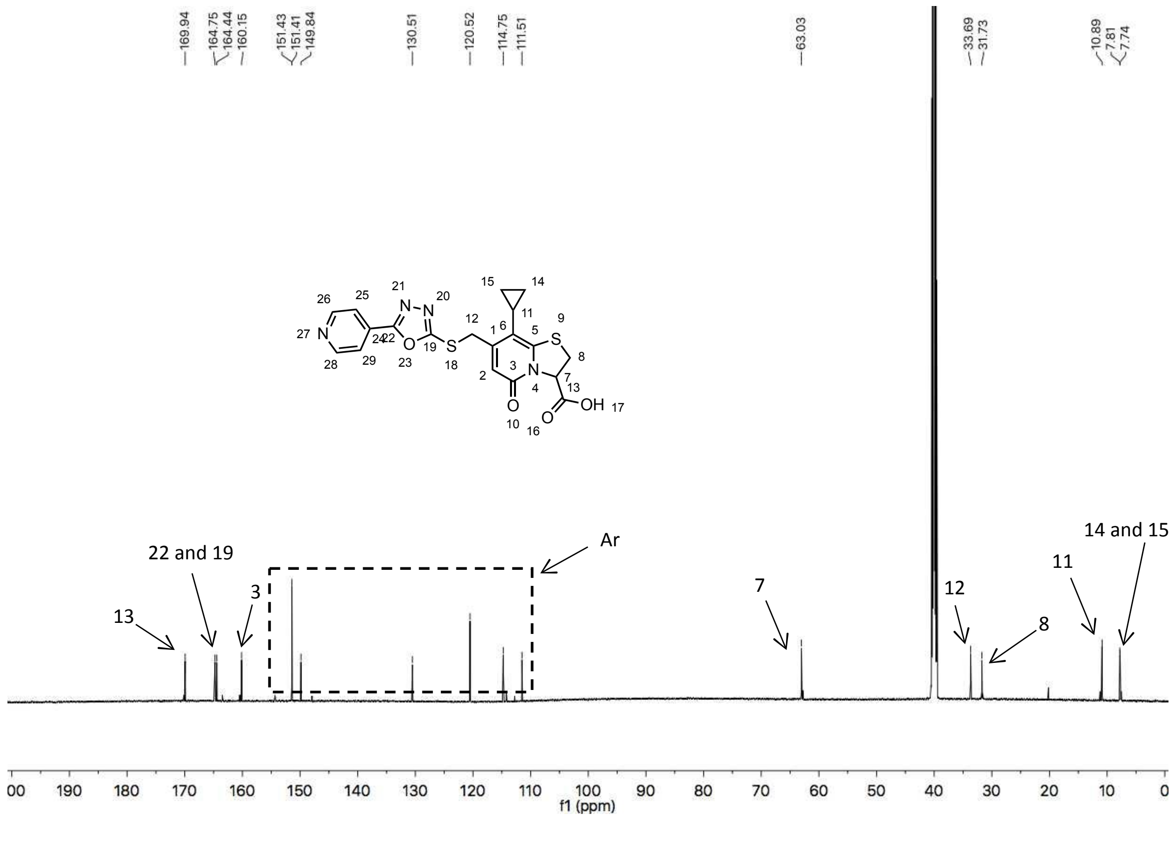

^{1}H NMR of **8j**

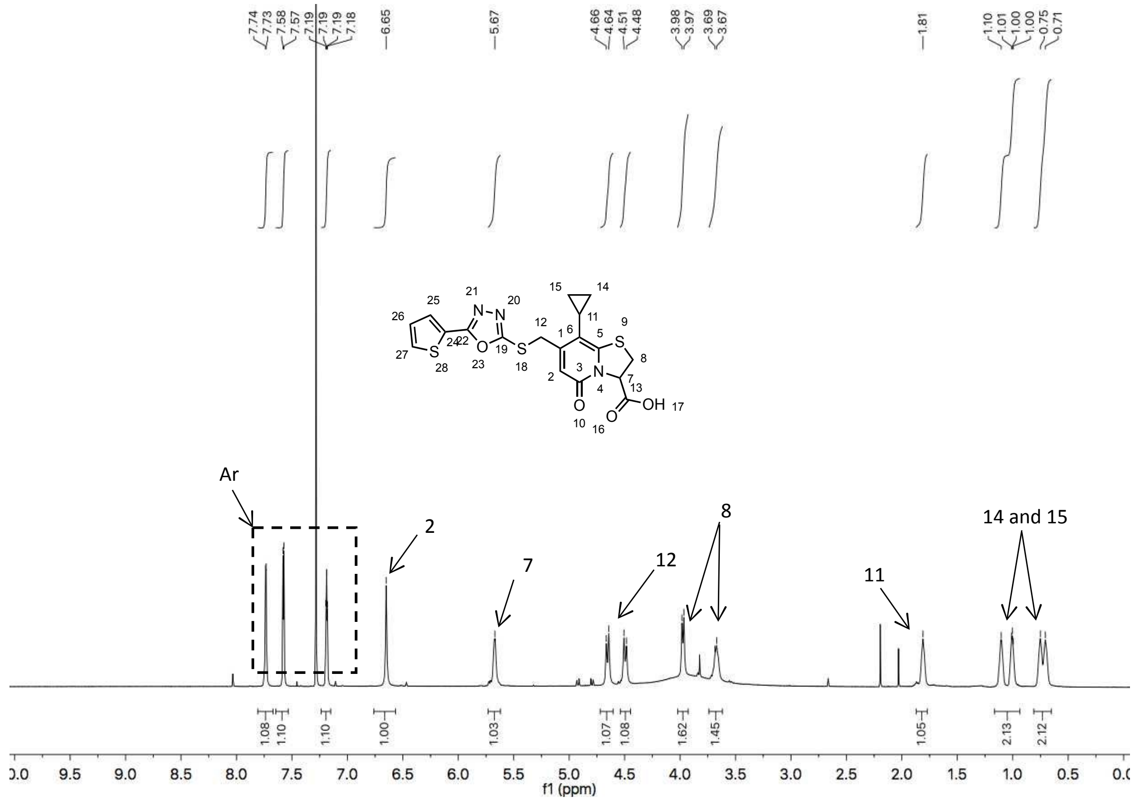

^{13}C NMR of **8j**

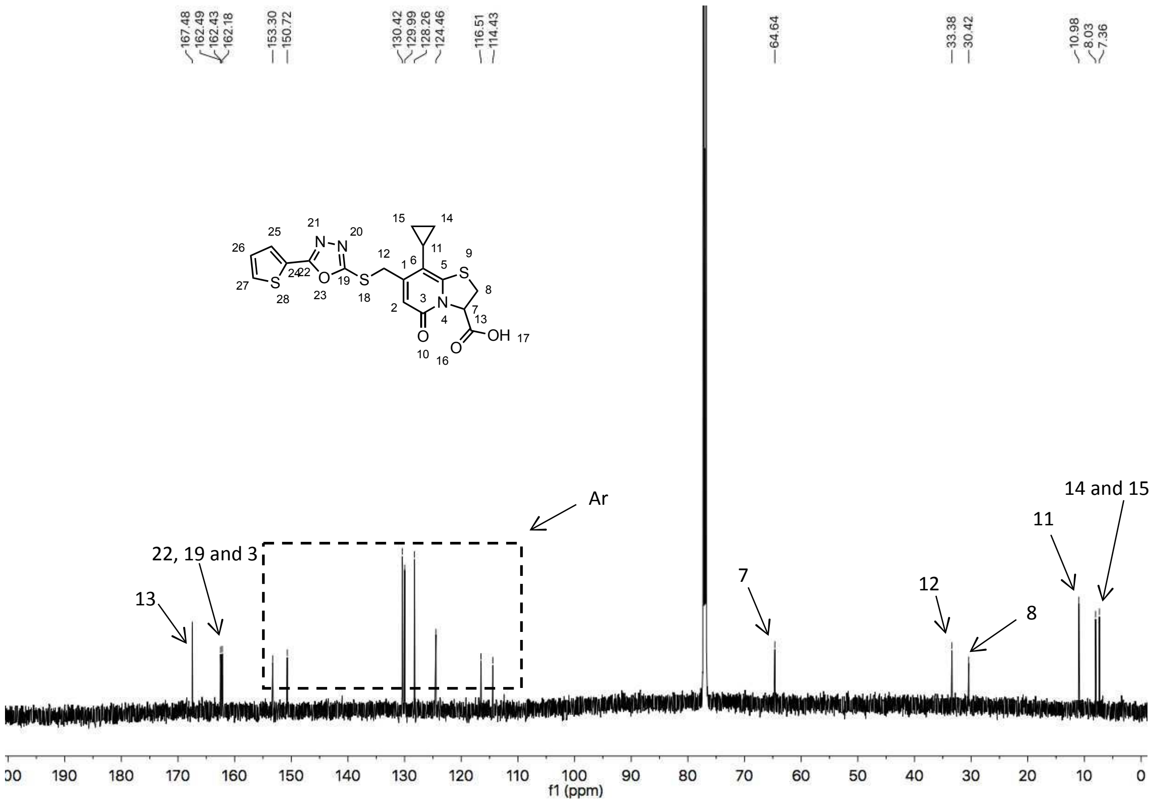

^{1}H NMR of **8k**

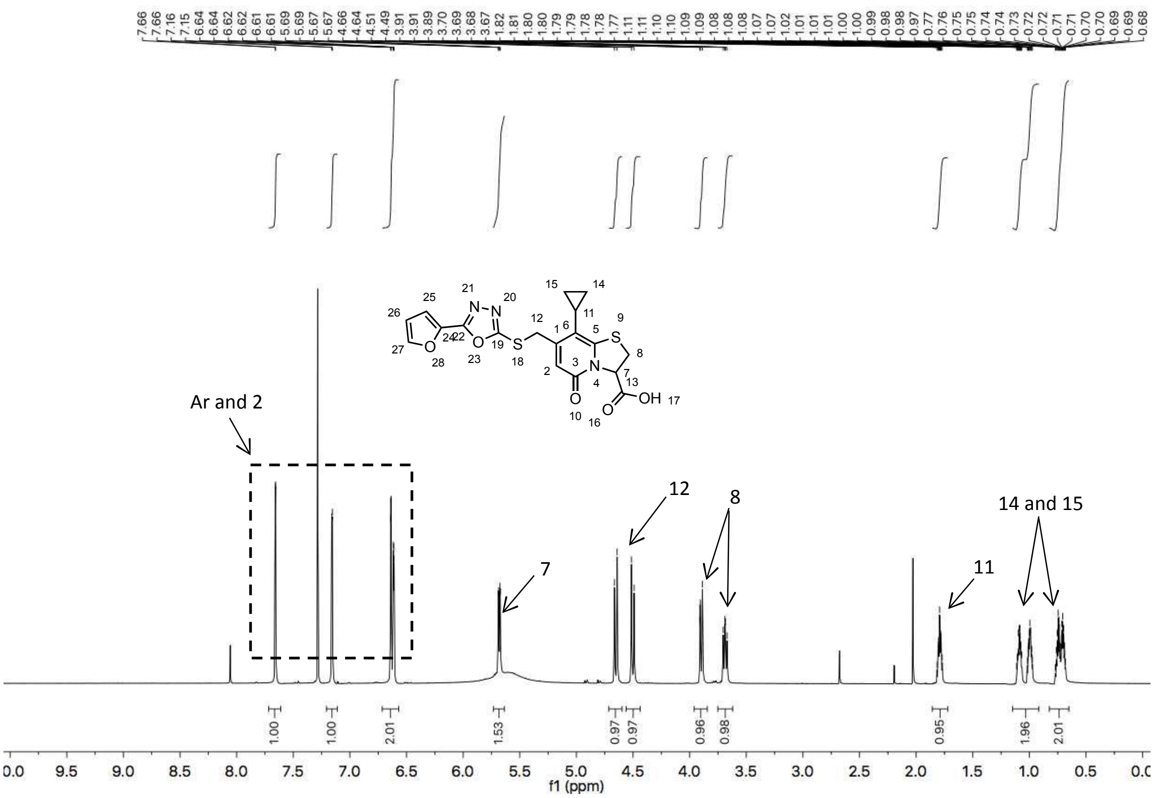

^{13}C NMR of **8k**

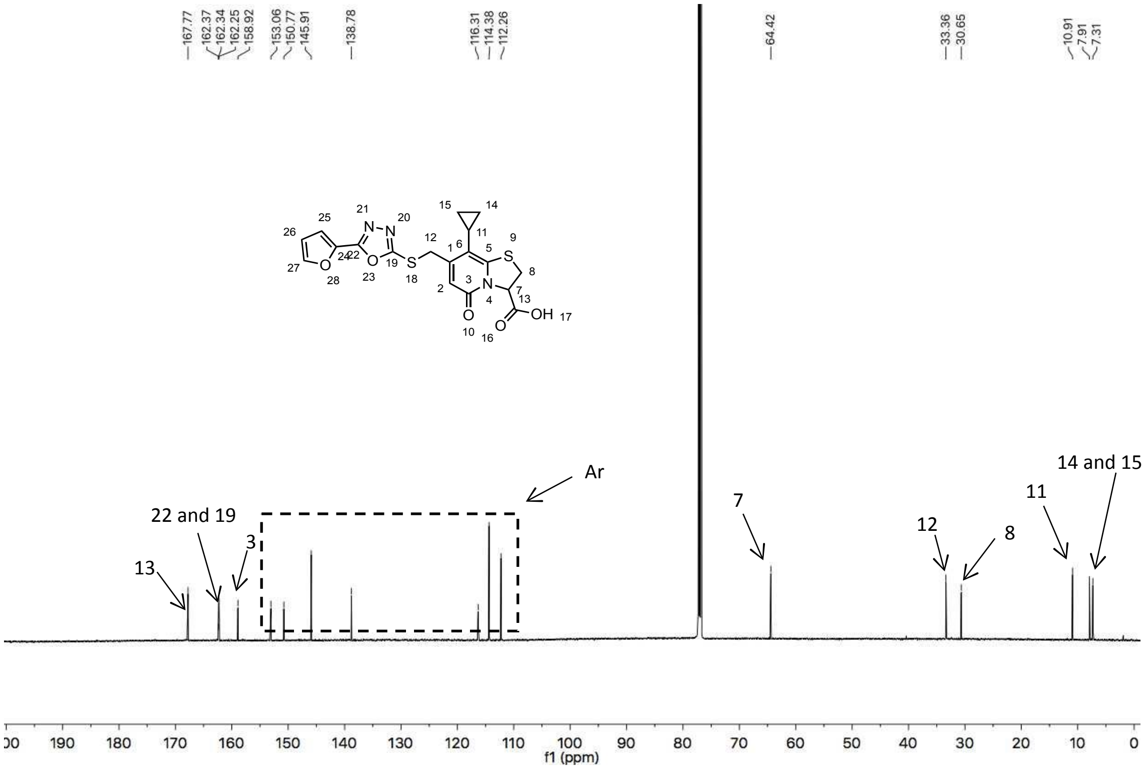

^{1}H NMR of **8l**

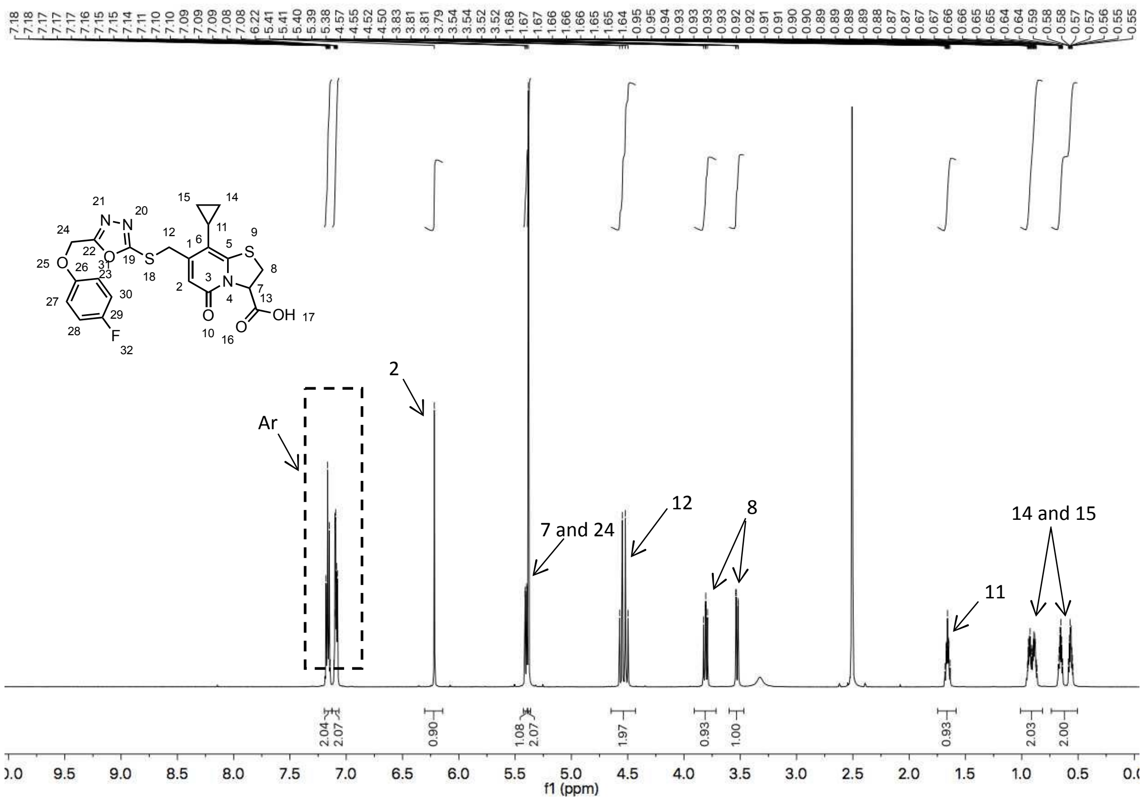

^{13}C NMR of **8l**

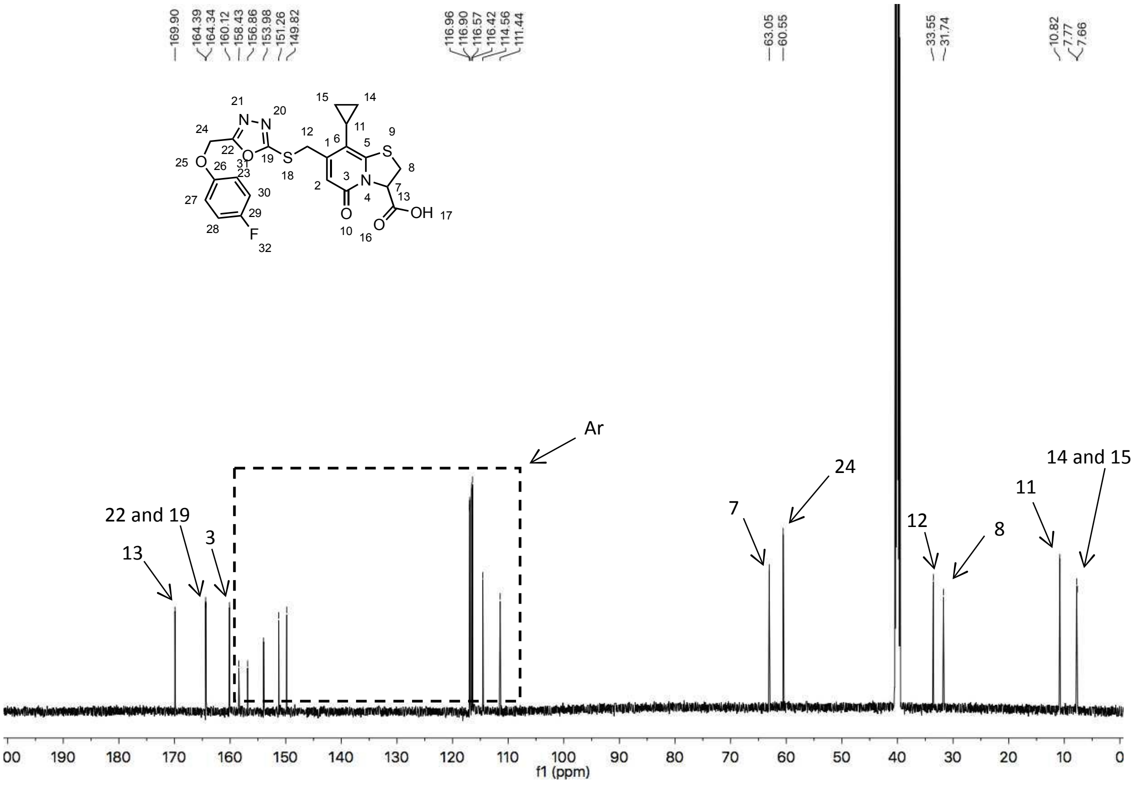

^{19}F NMR of **8l**

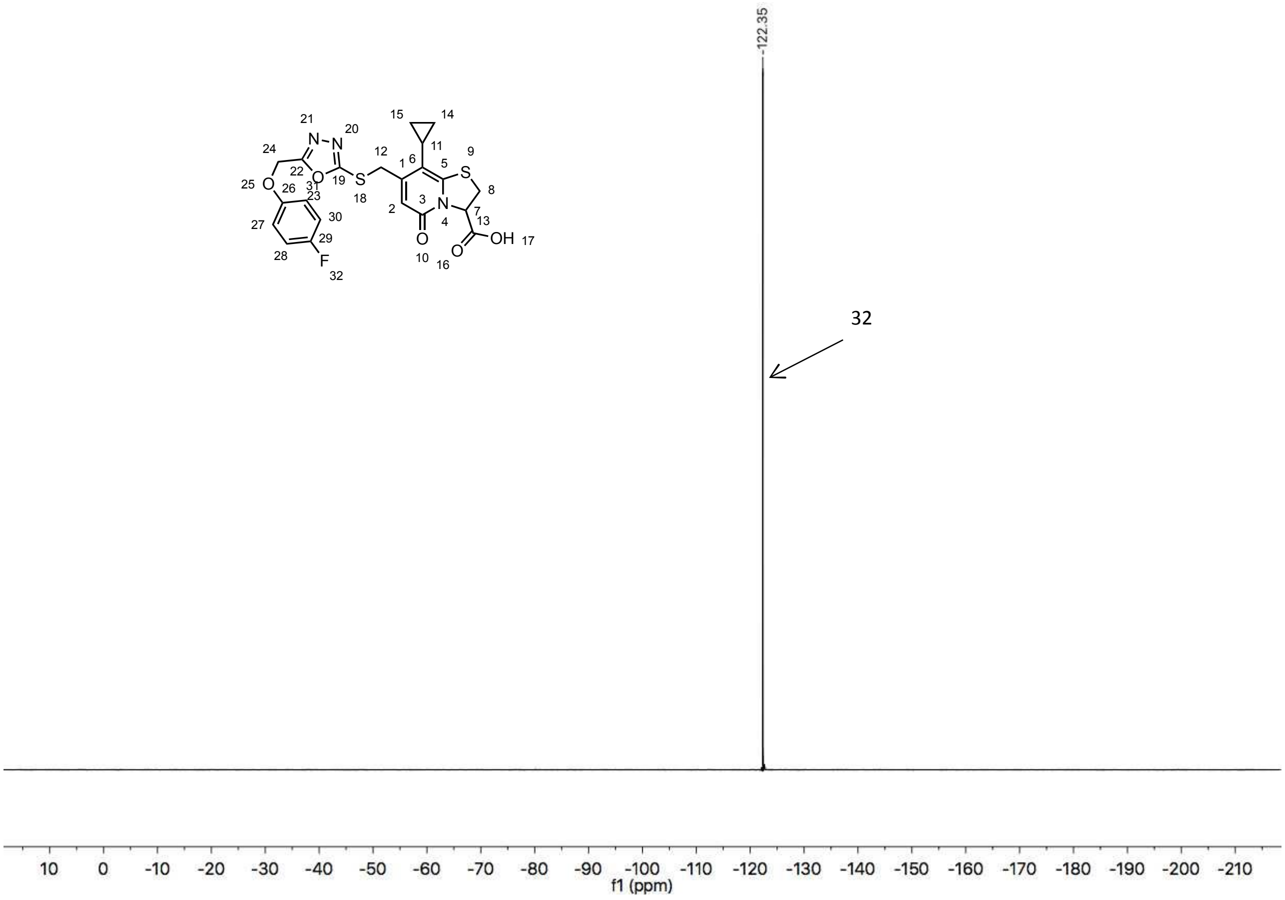
-122.35
32
10 0 -10 -20 -30 -40 -50 -60 -70 -80 -90 -100 -110 -120 -130 -140 -150 -160 -170 -180 -190 -200 -210
f1 (ppm)

^{1}H NMR of **9**

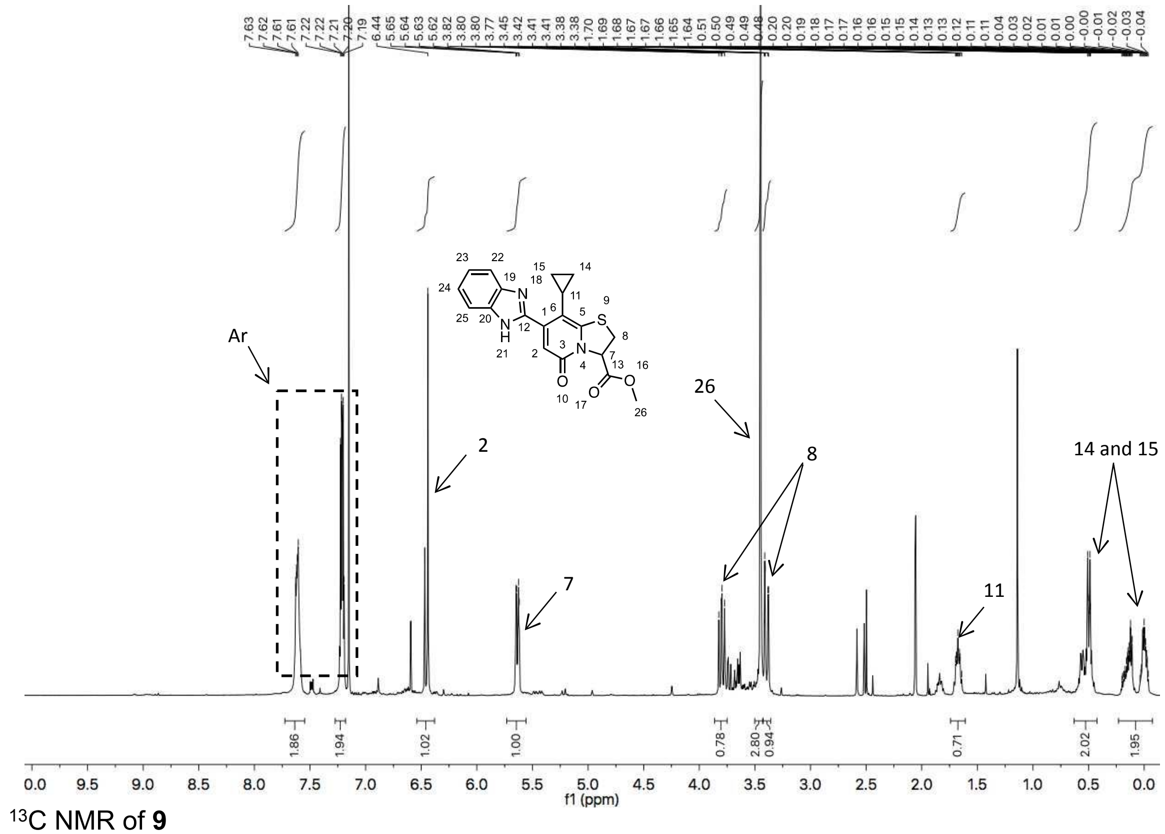

^{13}C NMR of **9**

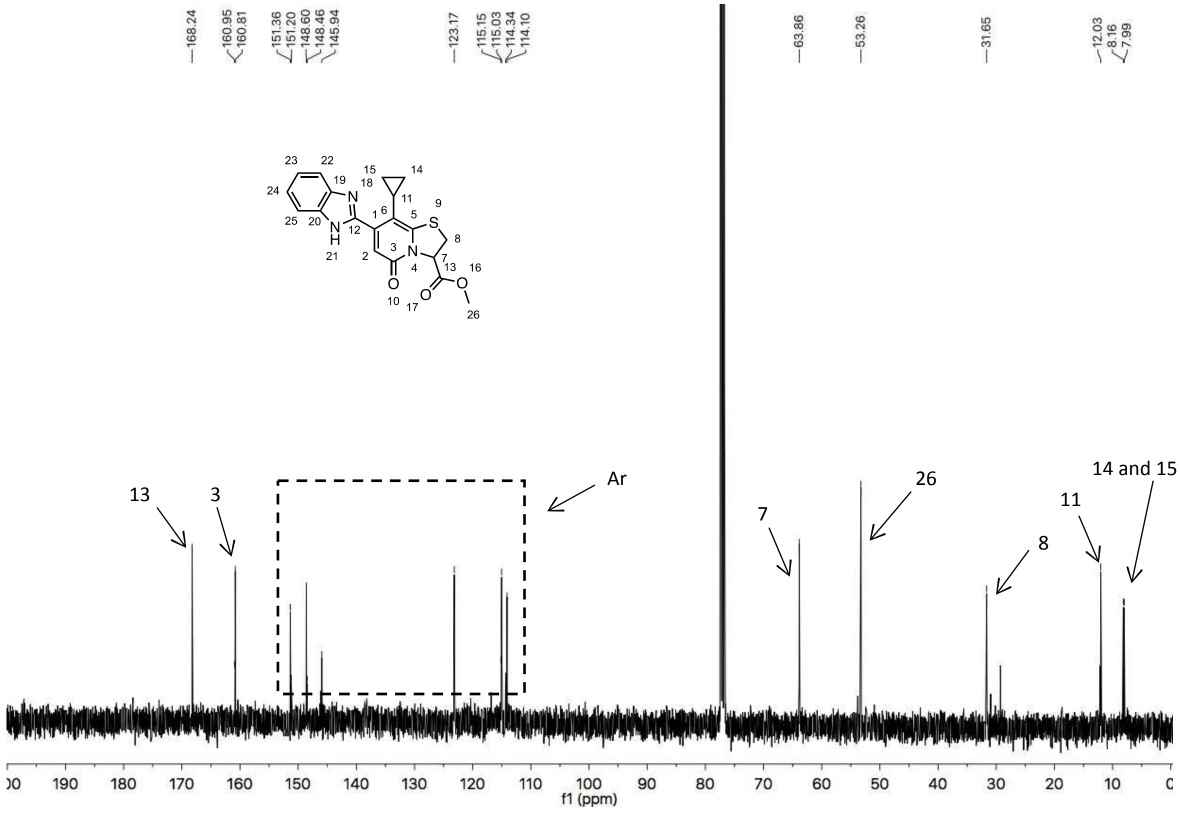

^{1}H NMR of **10**

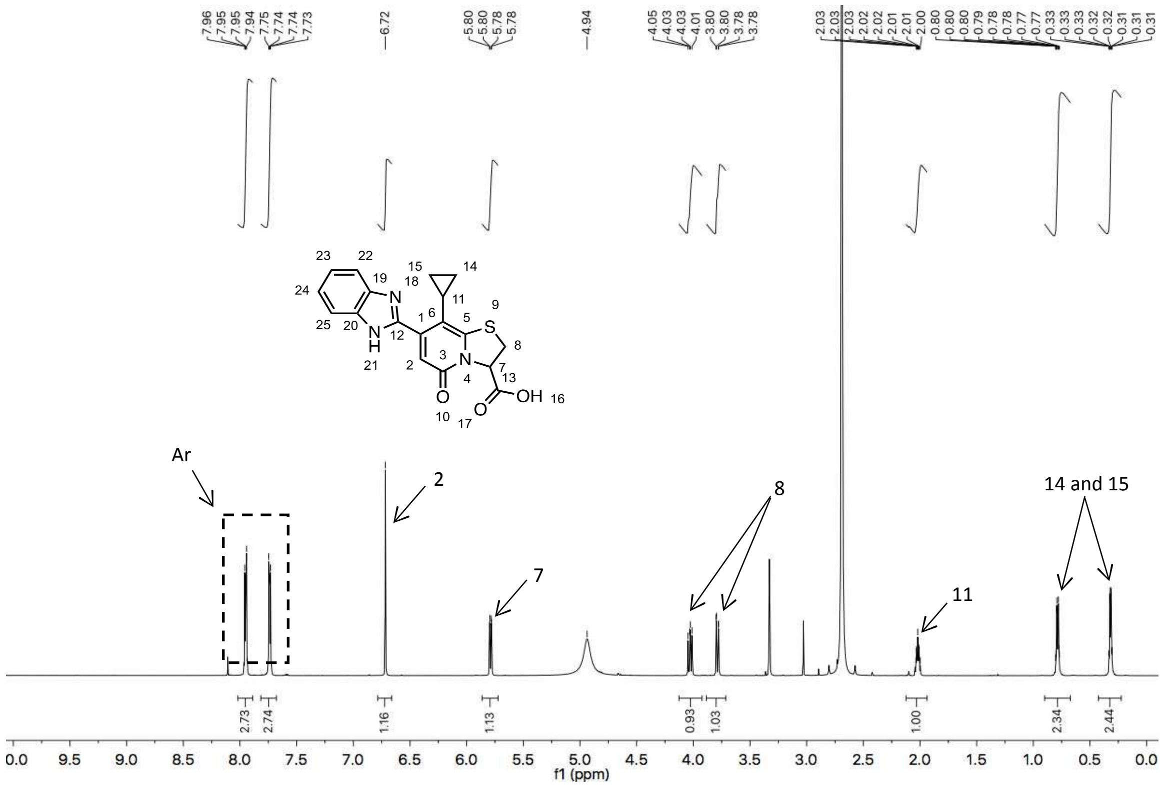

^{13}C NMR of **10**

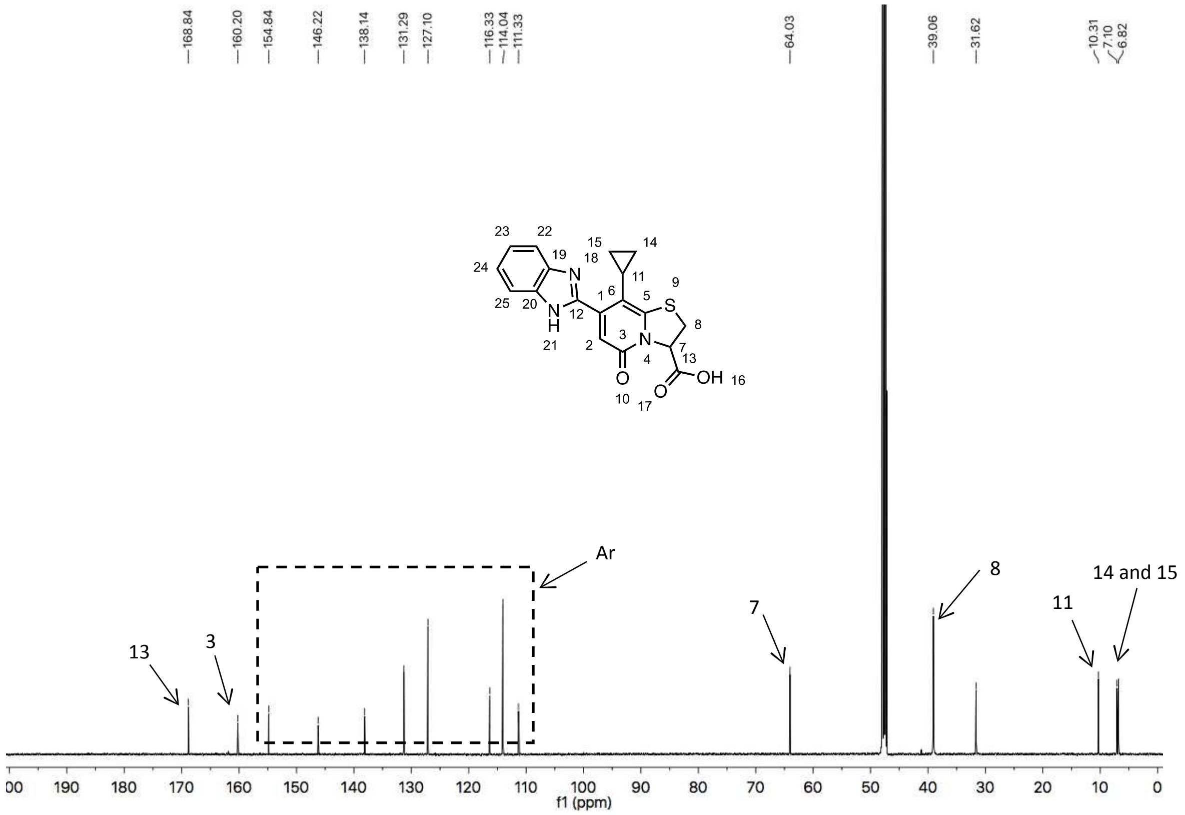

^{1}H NMR of **11**

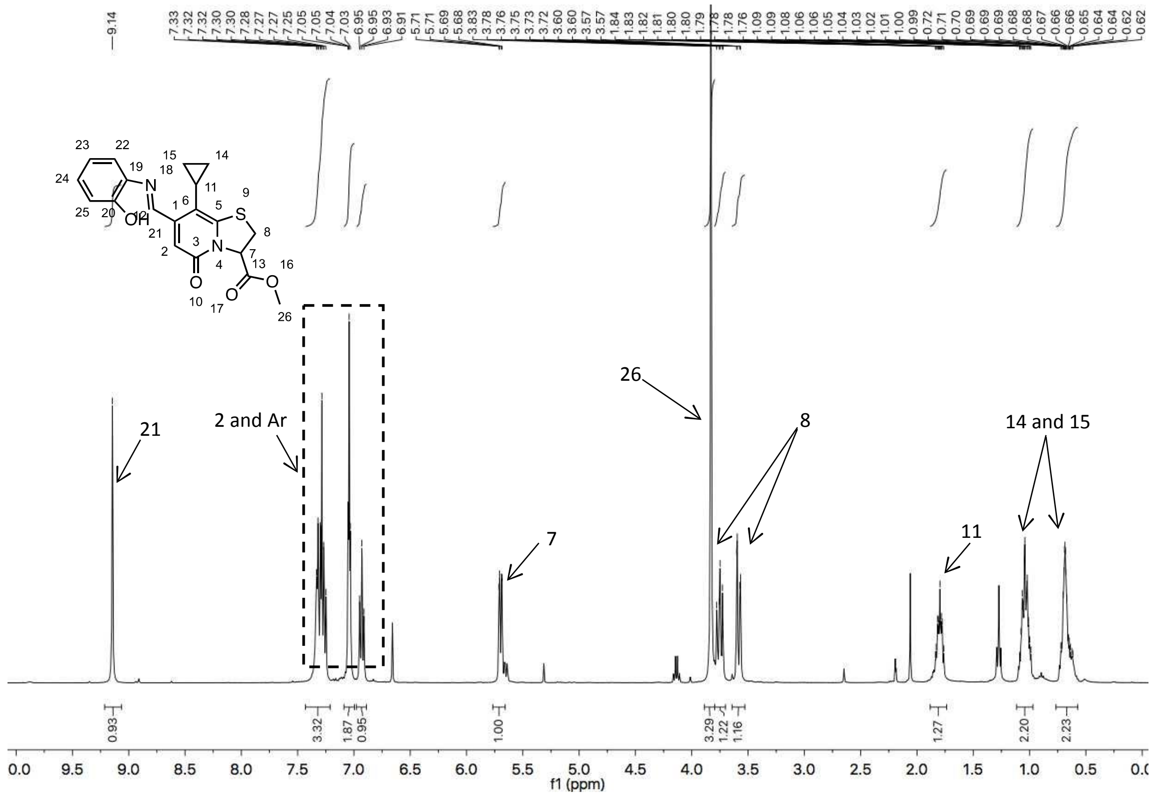

^{13}C NMR of **11**

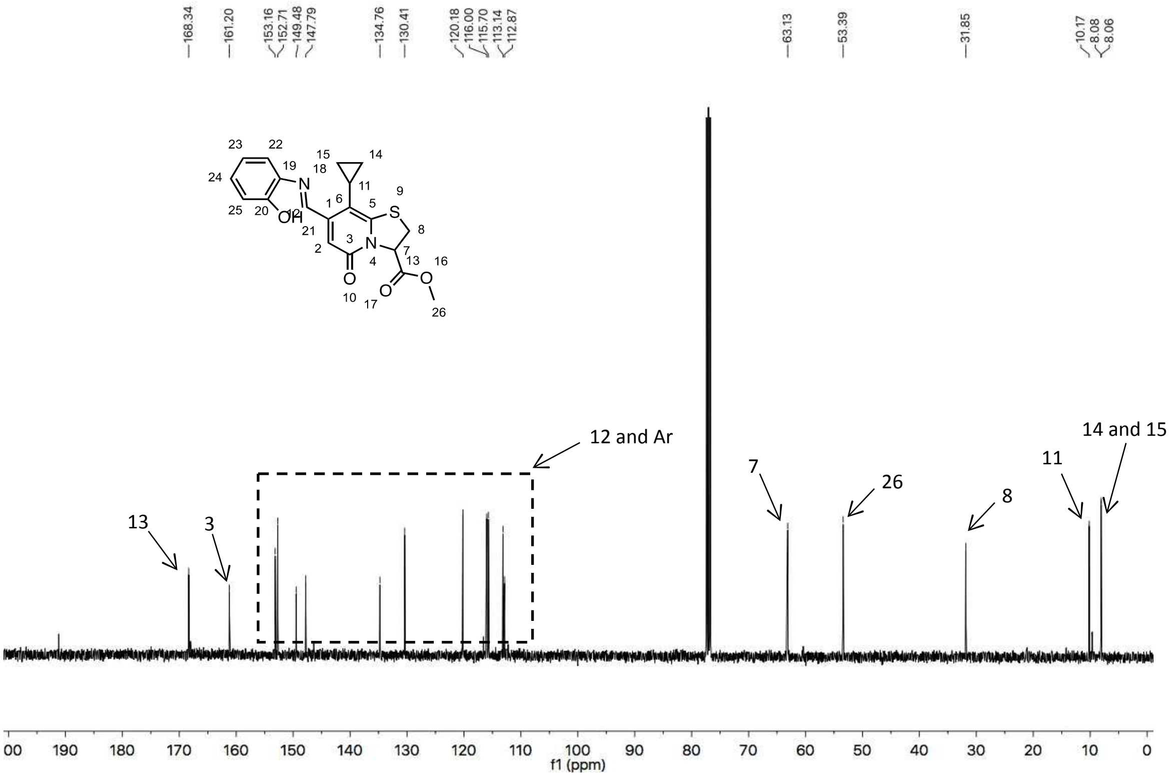

^{1}H NMR of **12**

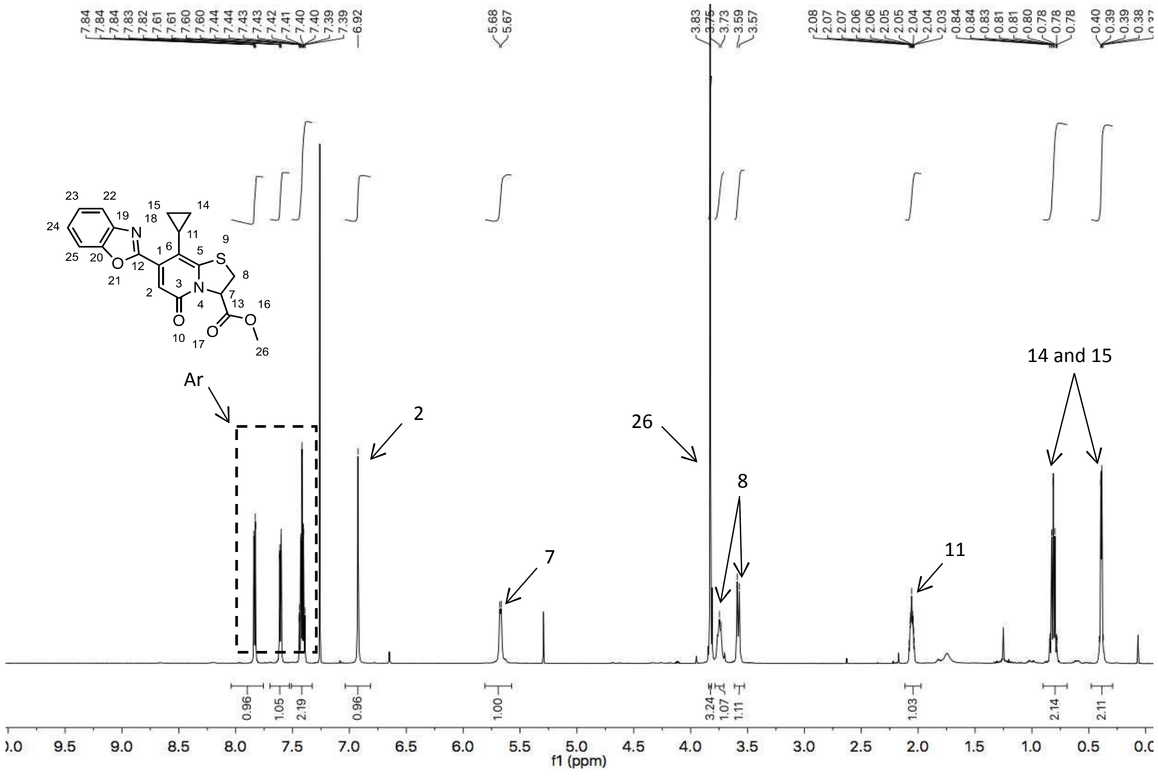

^{13}C NMR of **12**

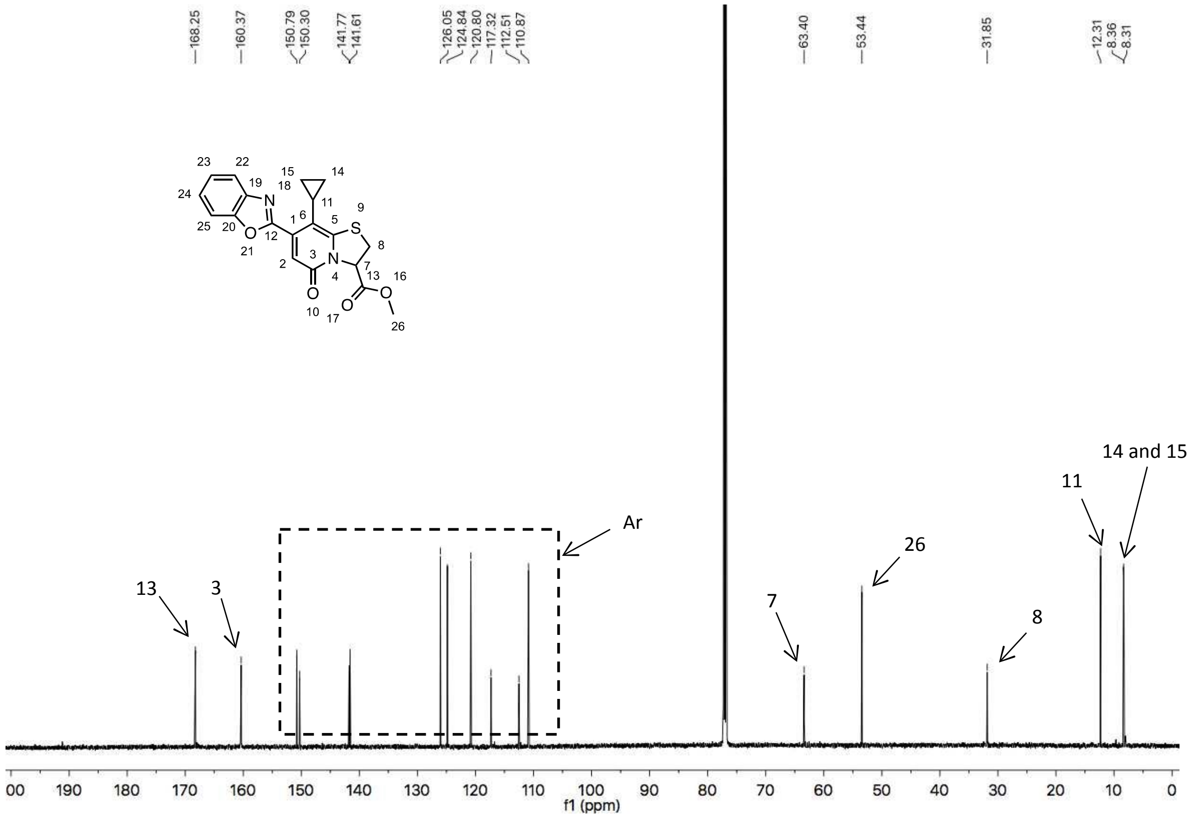

^{1}H NMR of **13**

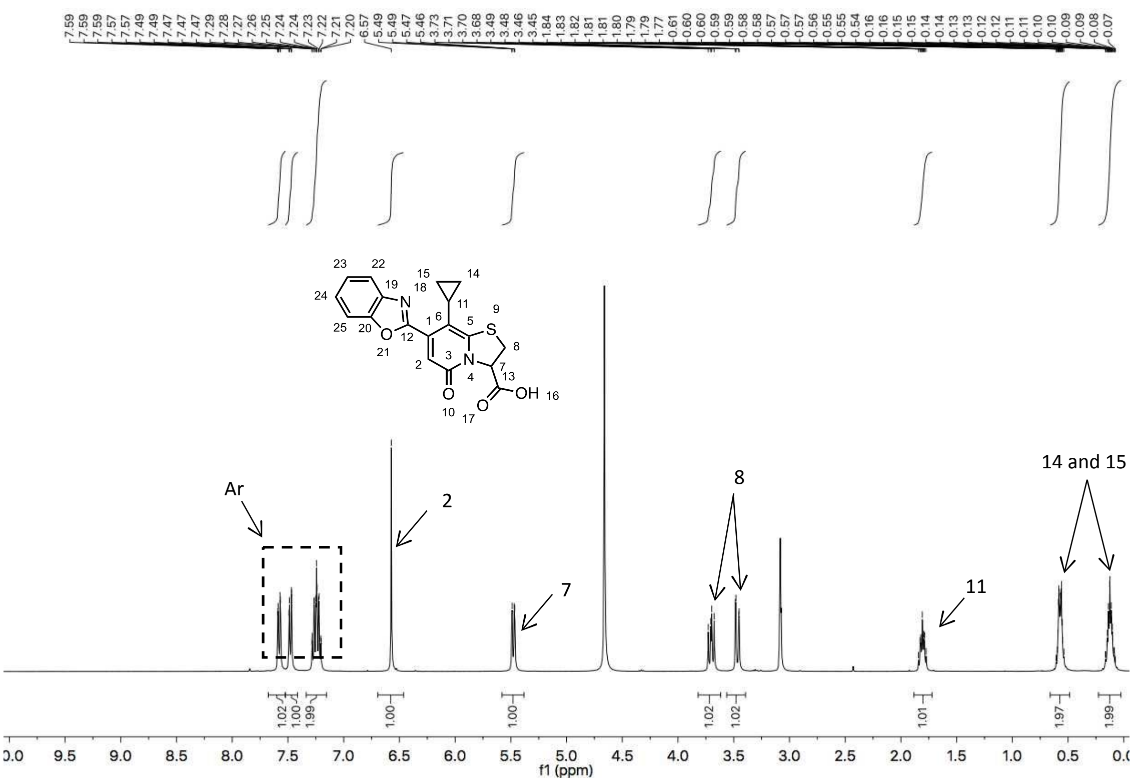

^{13}C NMR of **13**

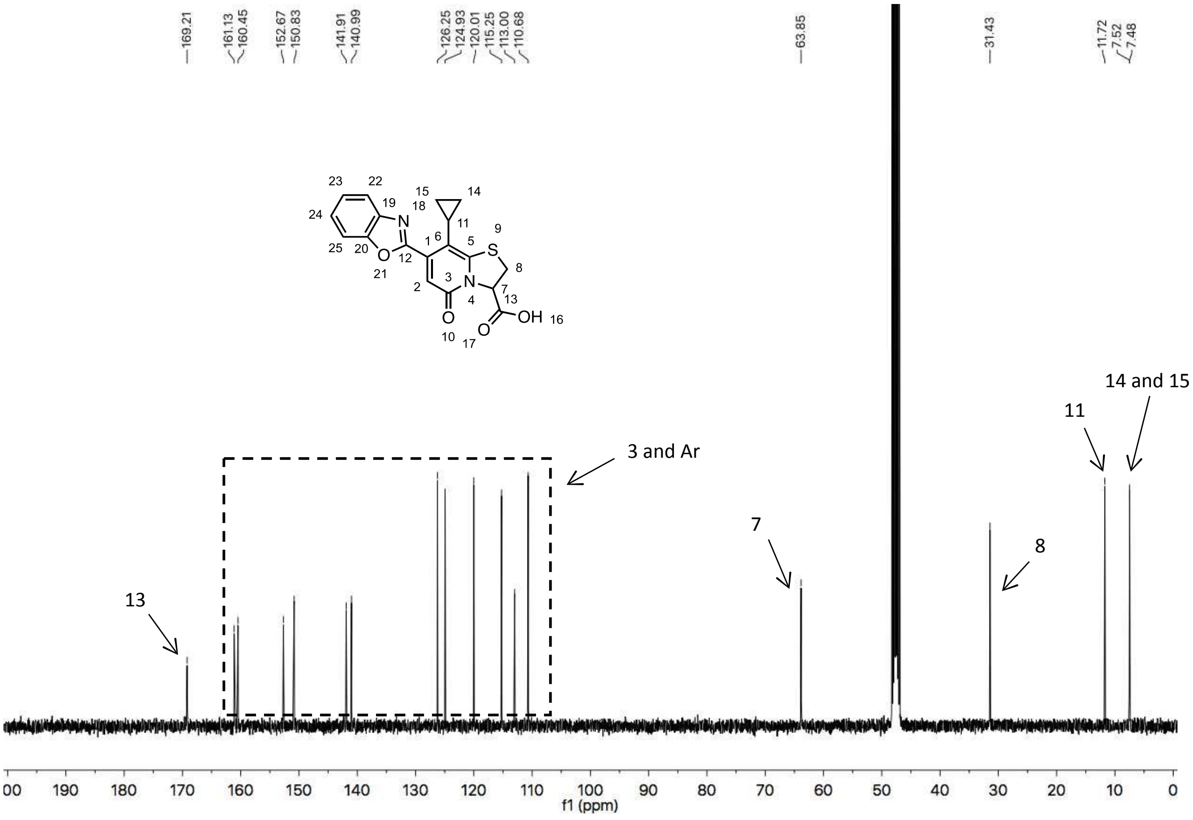

^{1}H NMR of **14a**

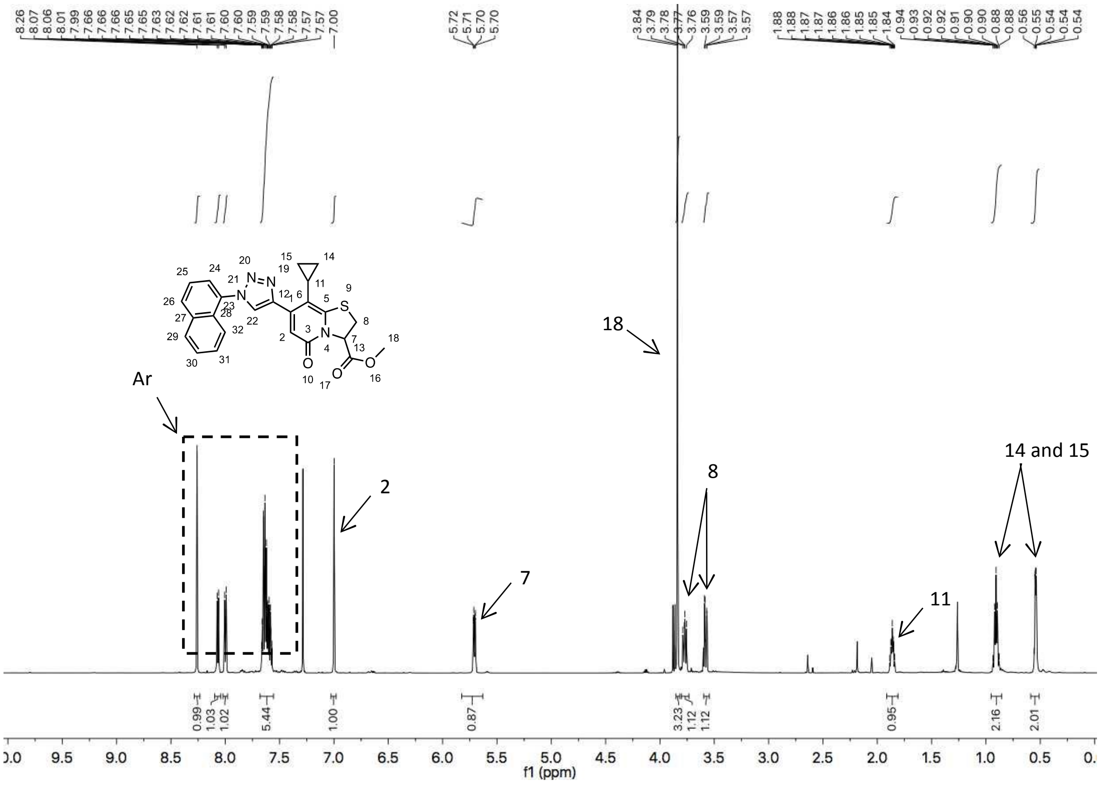

^{13}C NMR of **14a**

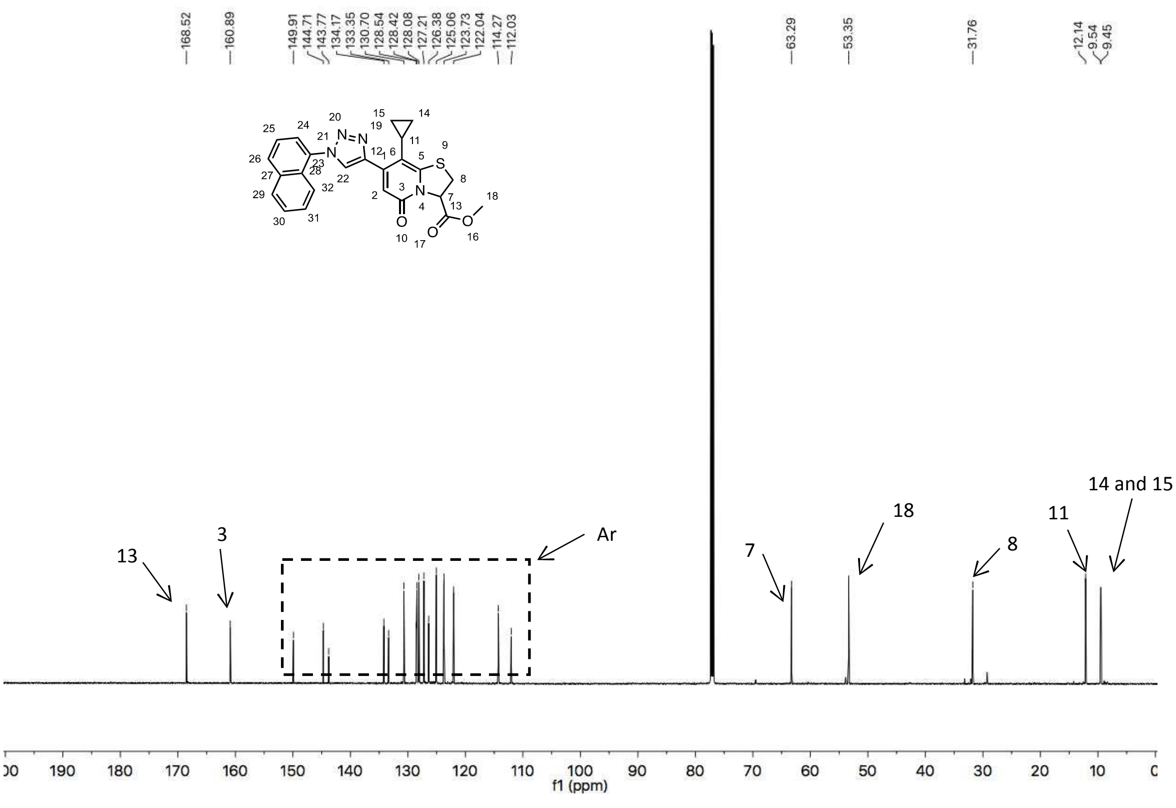

^{1}H NMR of **14b**

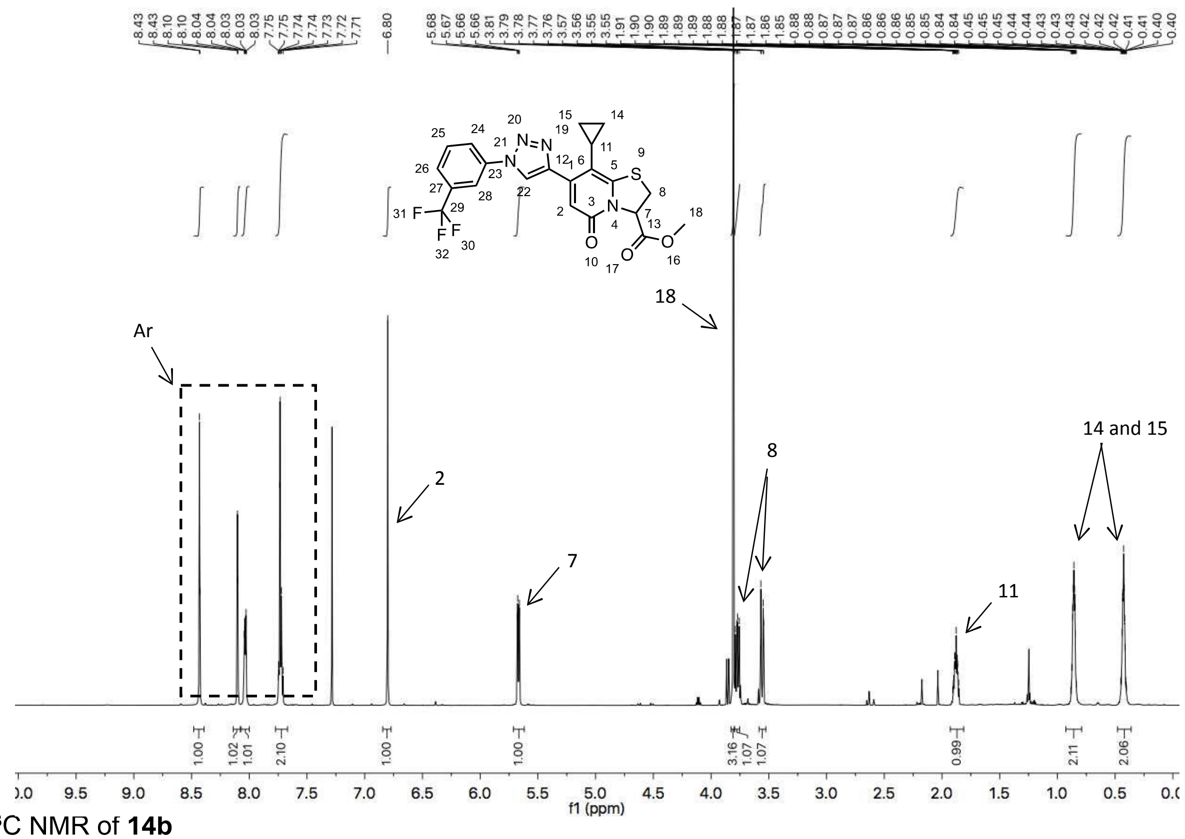

^{13}C NMR of **14b**

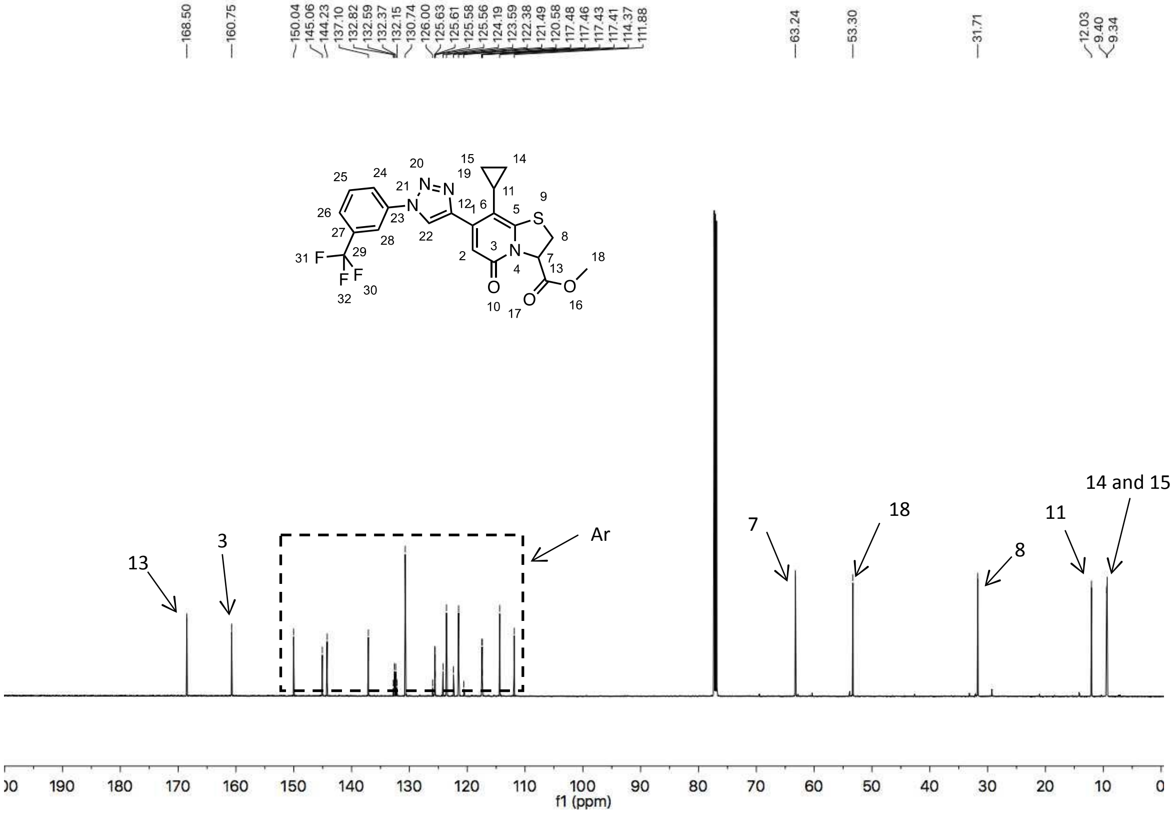

^{19}F NMR of **14b**

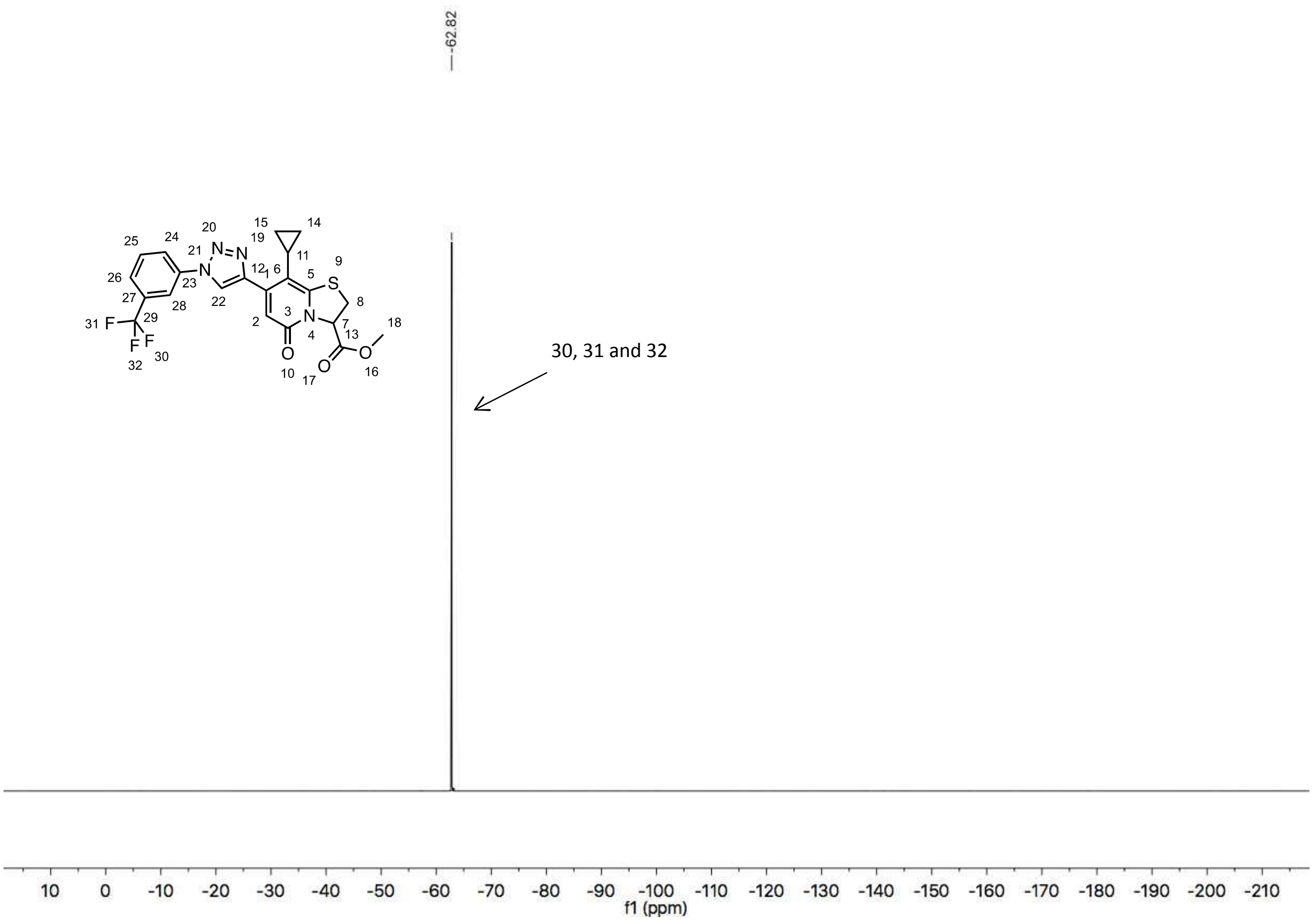

^{1}H NMR of **14c**

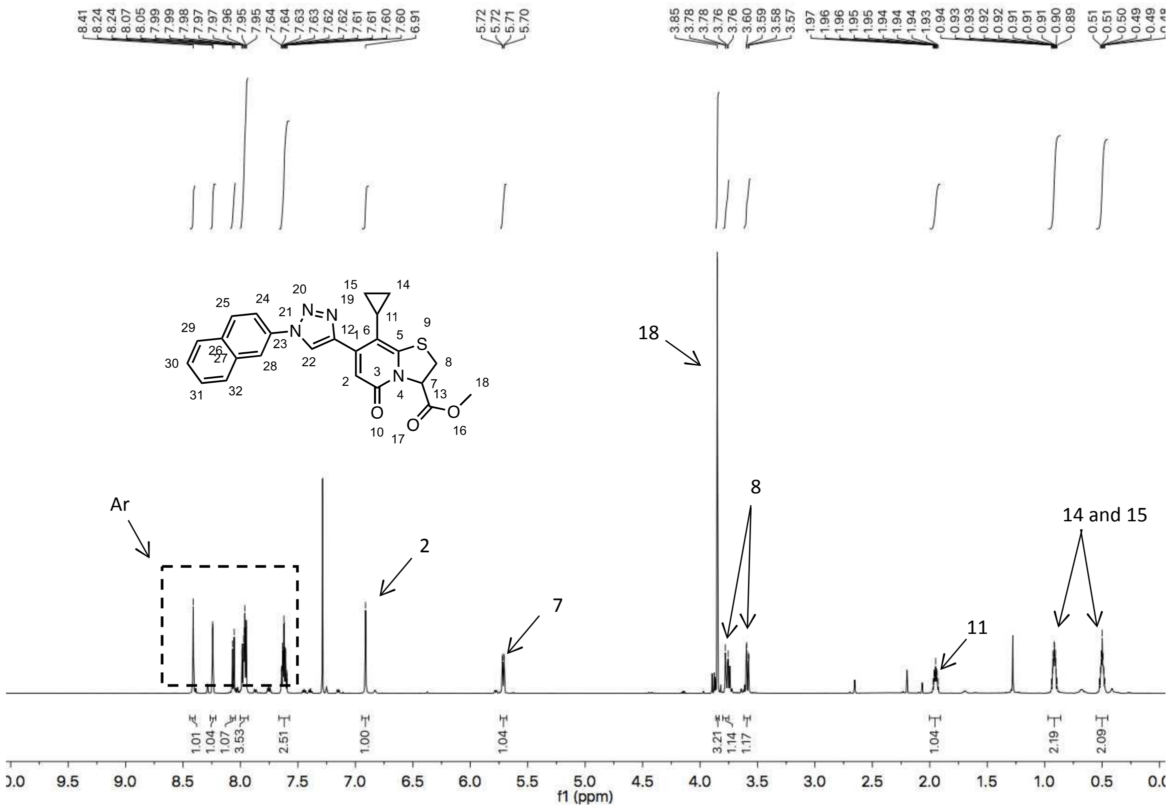

^{13}C NMR of **14c**

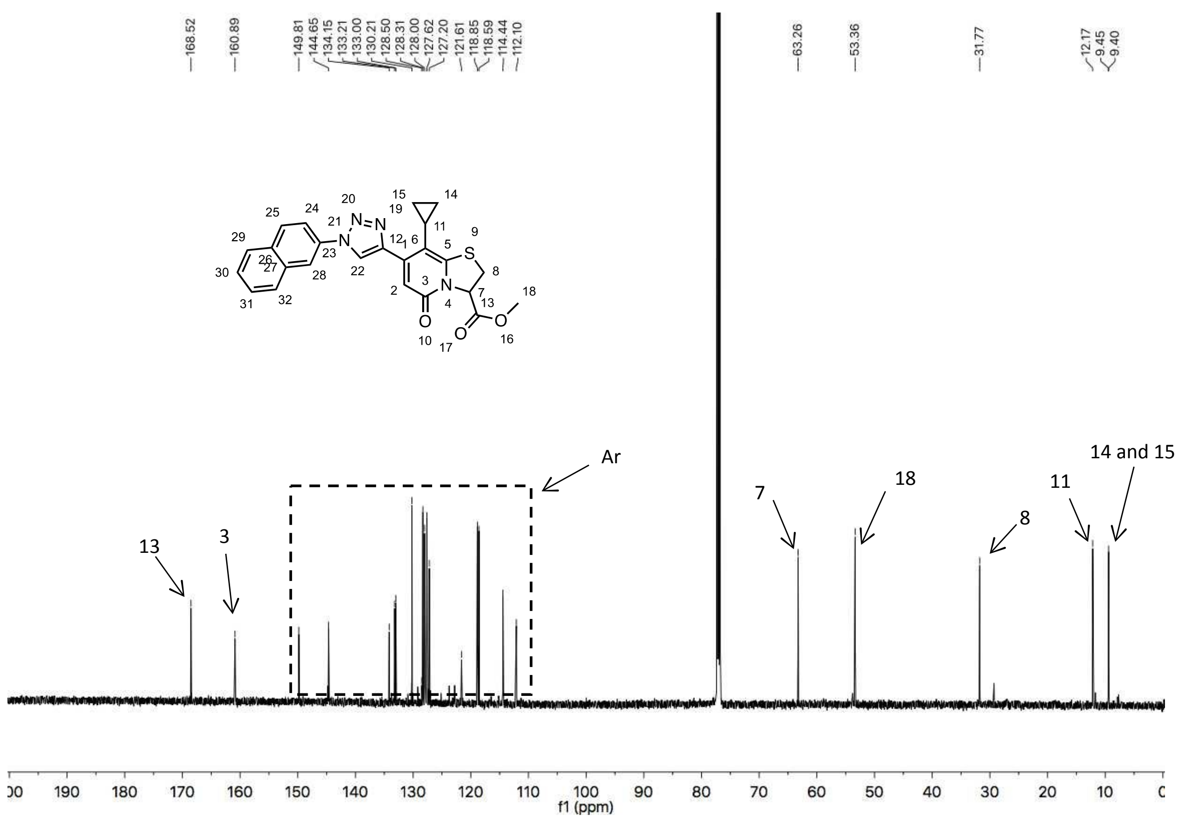

^{1}H NMR of **14d**

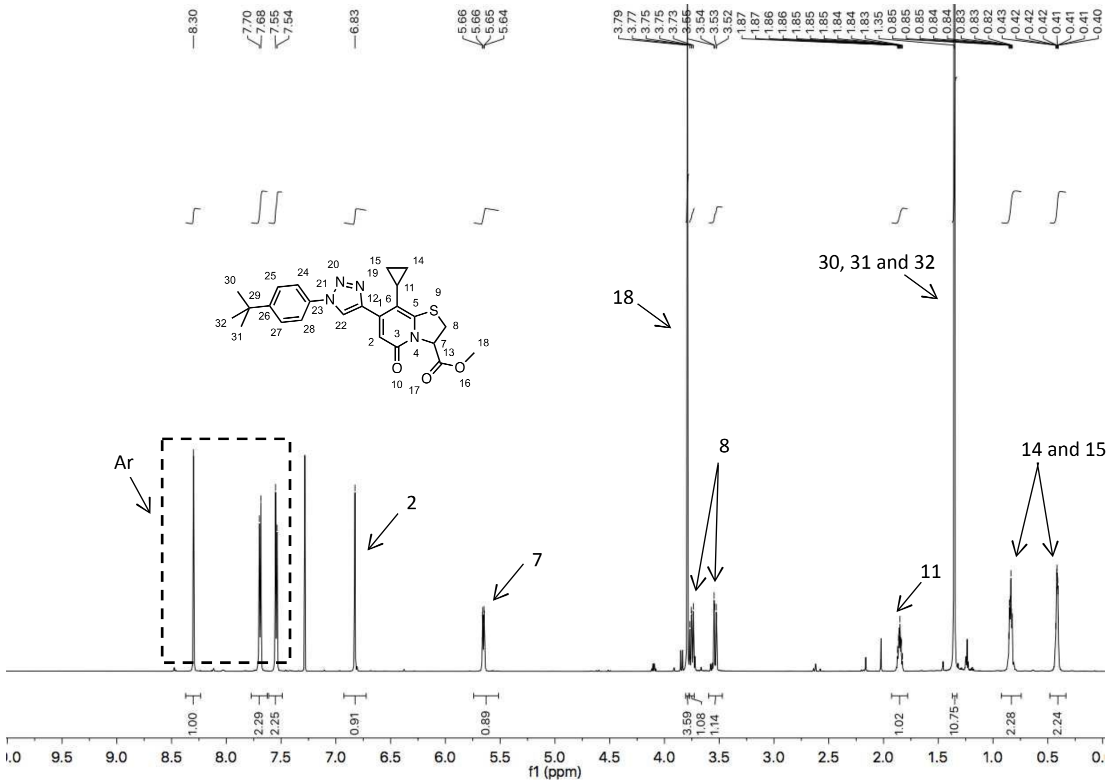

^{13}C NMR of **14d**

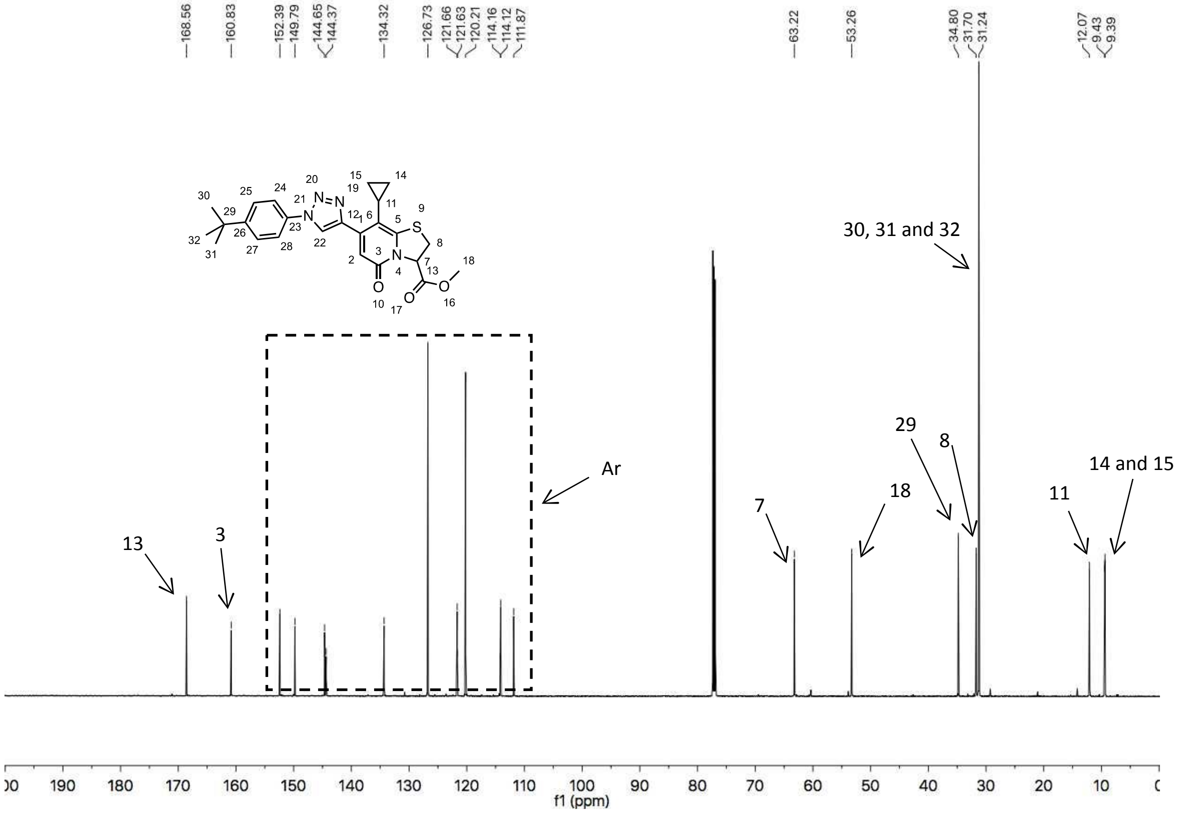

^{1}H NMR of **15a**

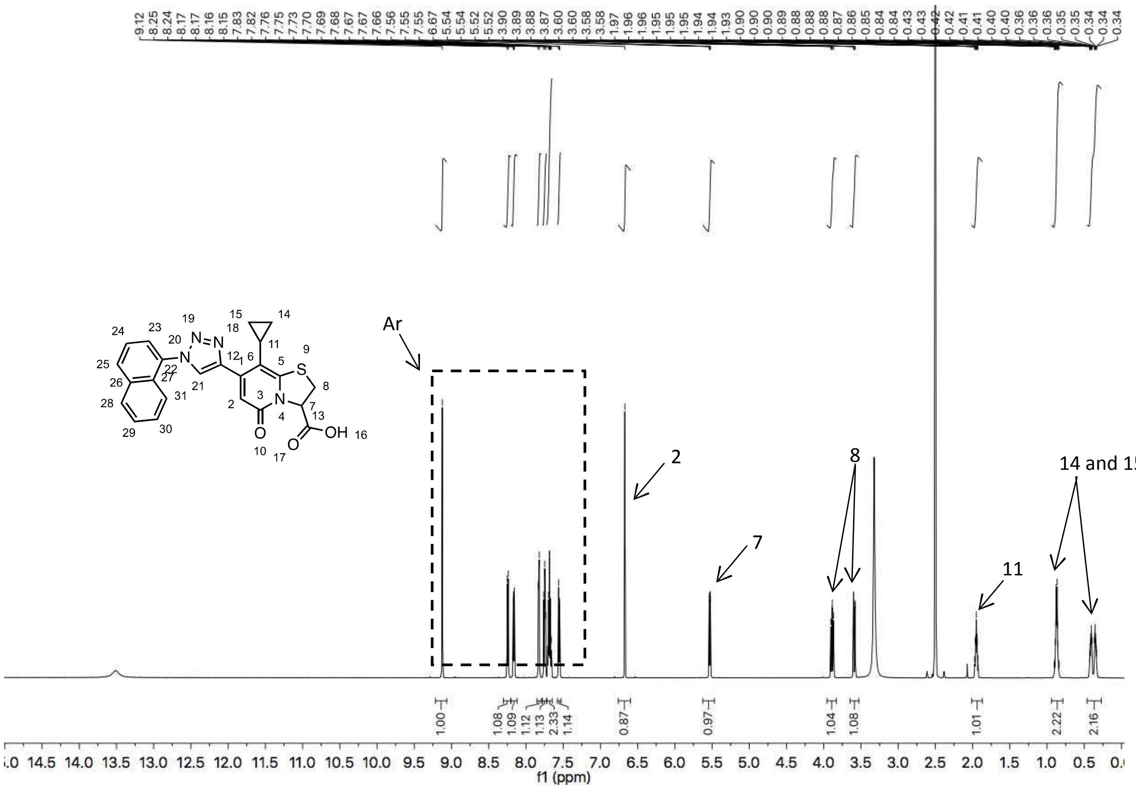

^{13}C NMR of **15a**

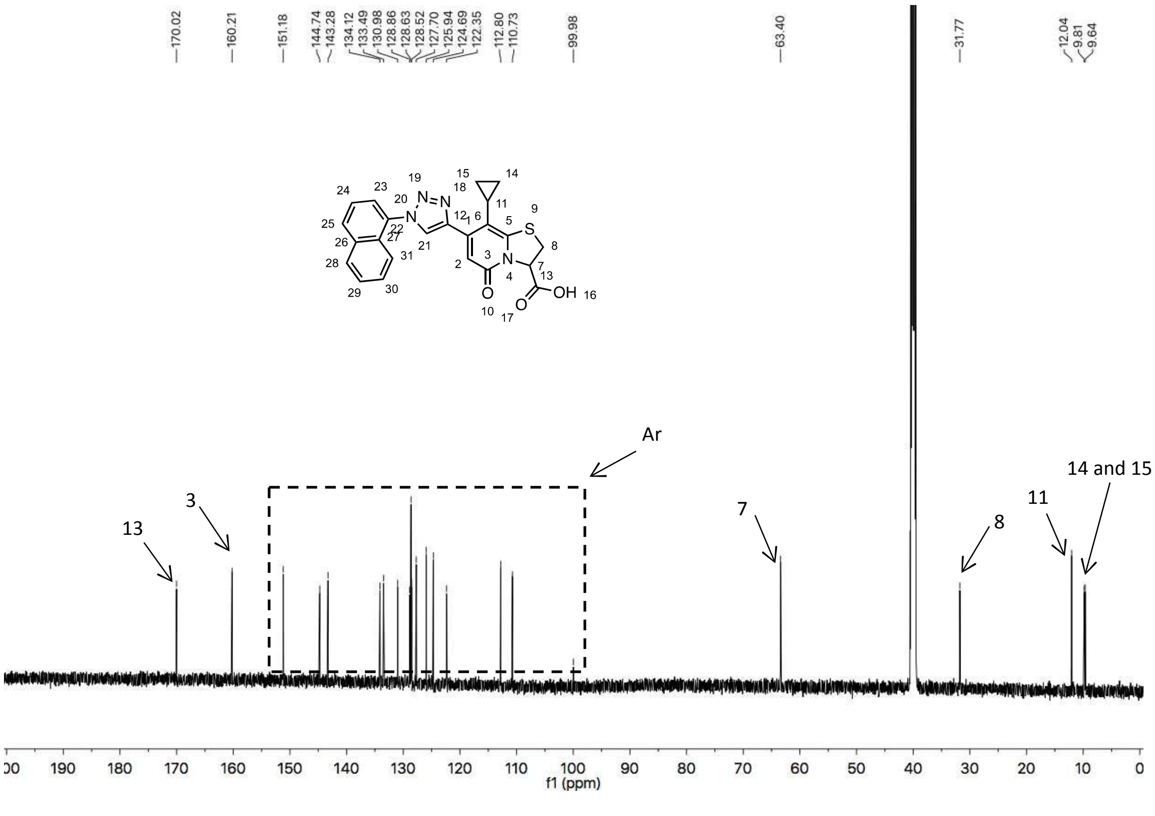

^{1}H NMR of **15b**

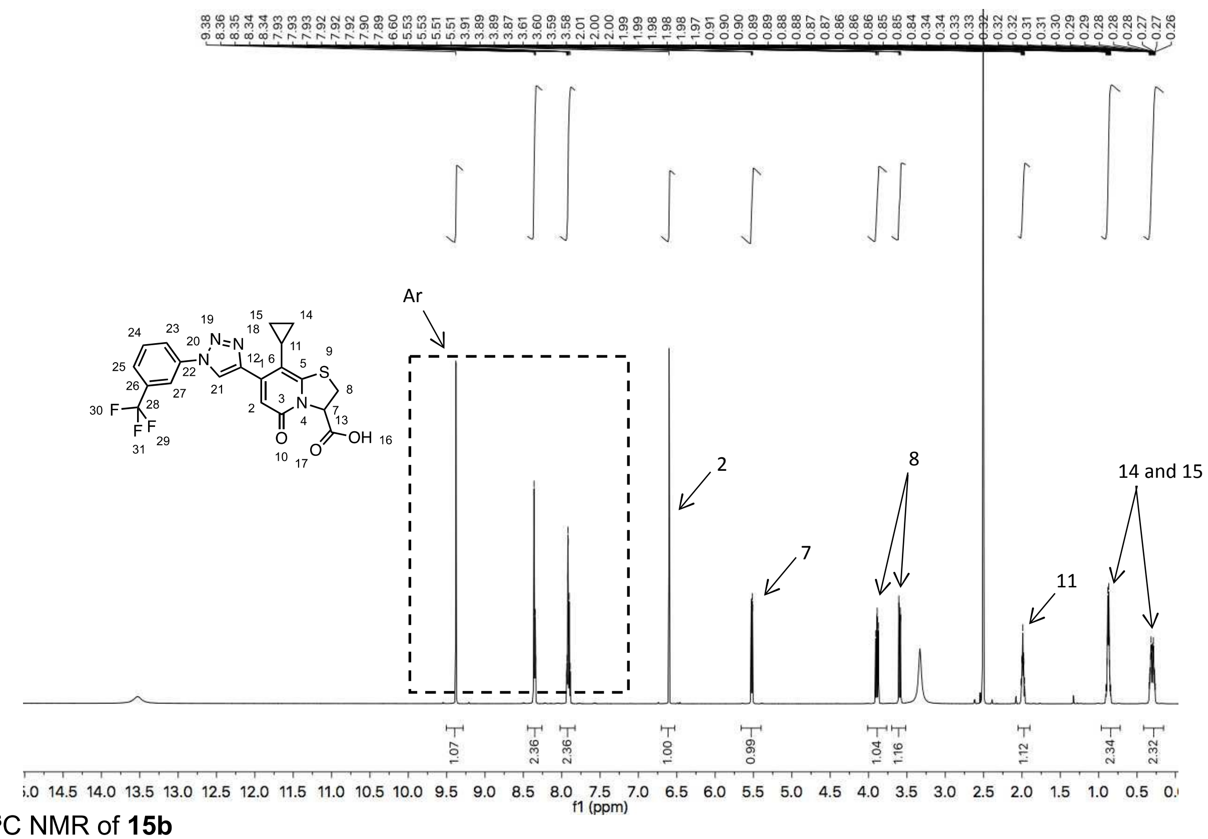

^{13}C NMR of **15b**

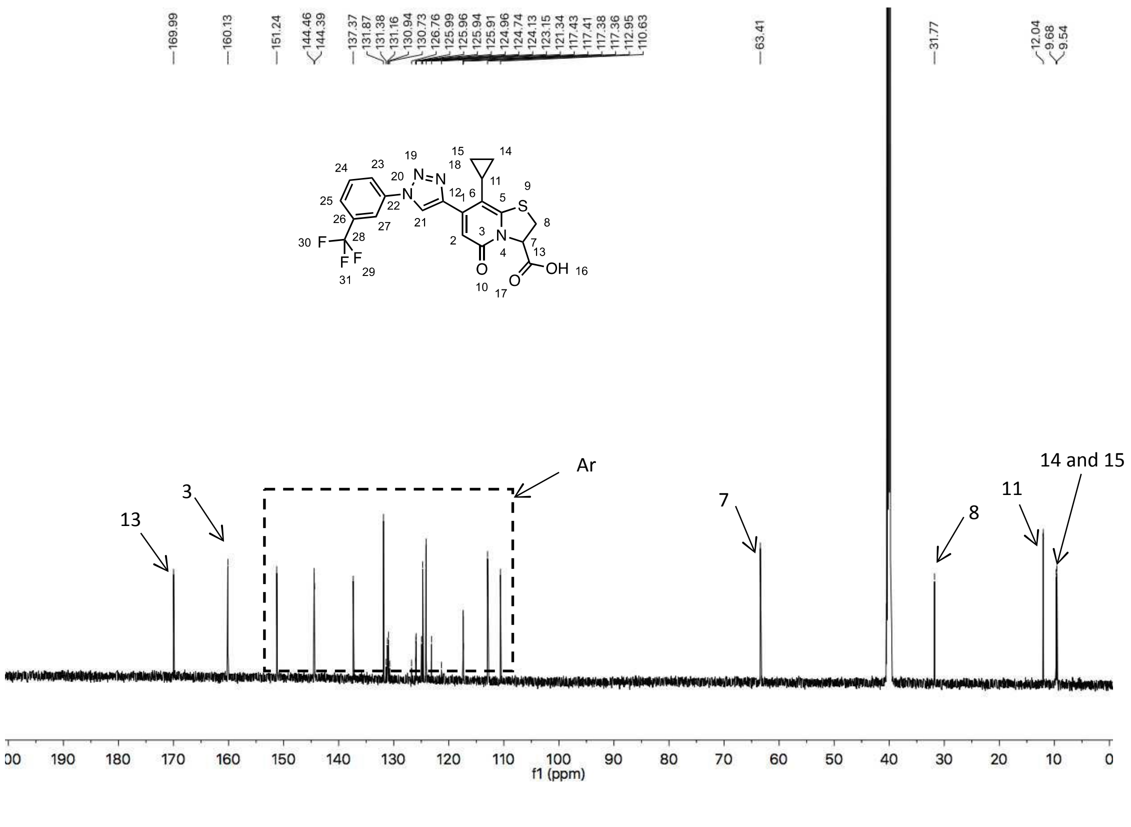

^{19}F NMR of **15b**

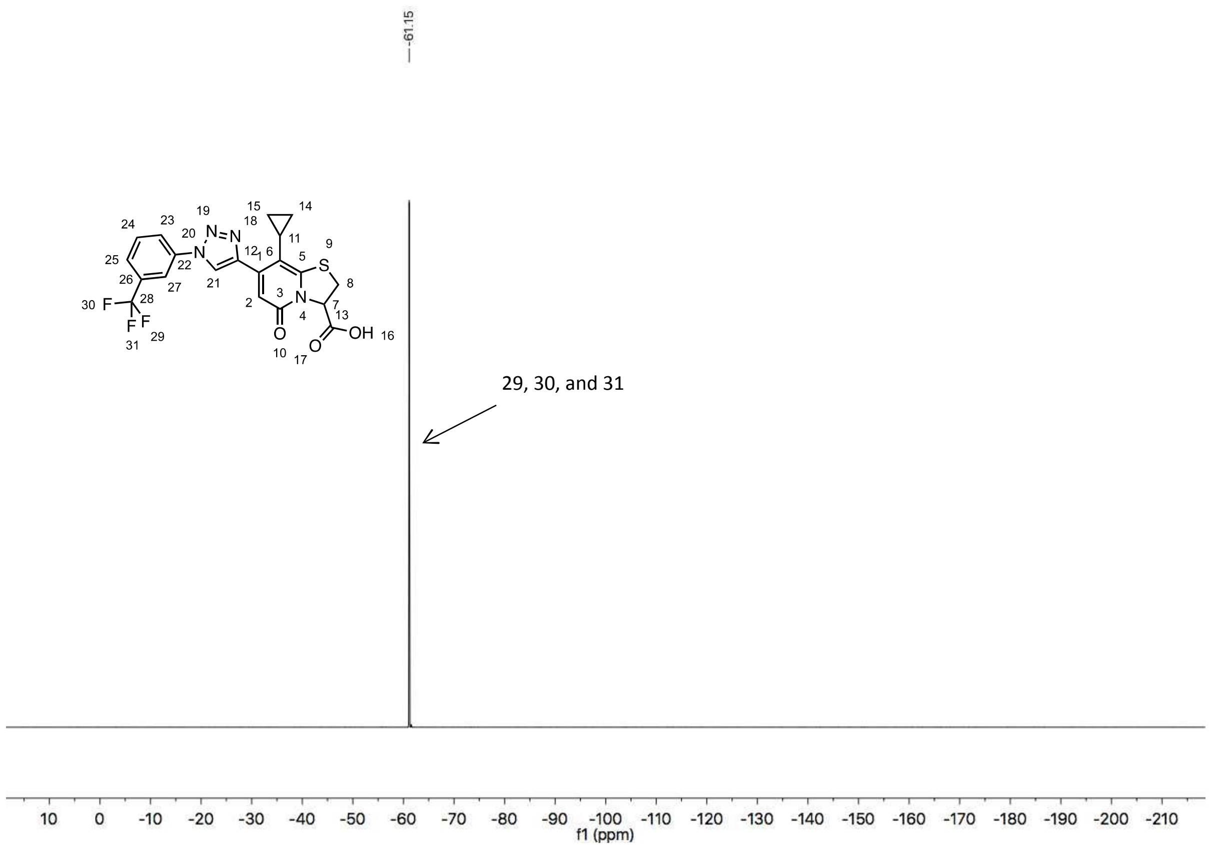

^{1}H NMR of **15c**

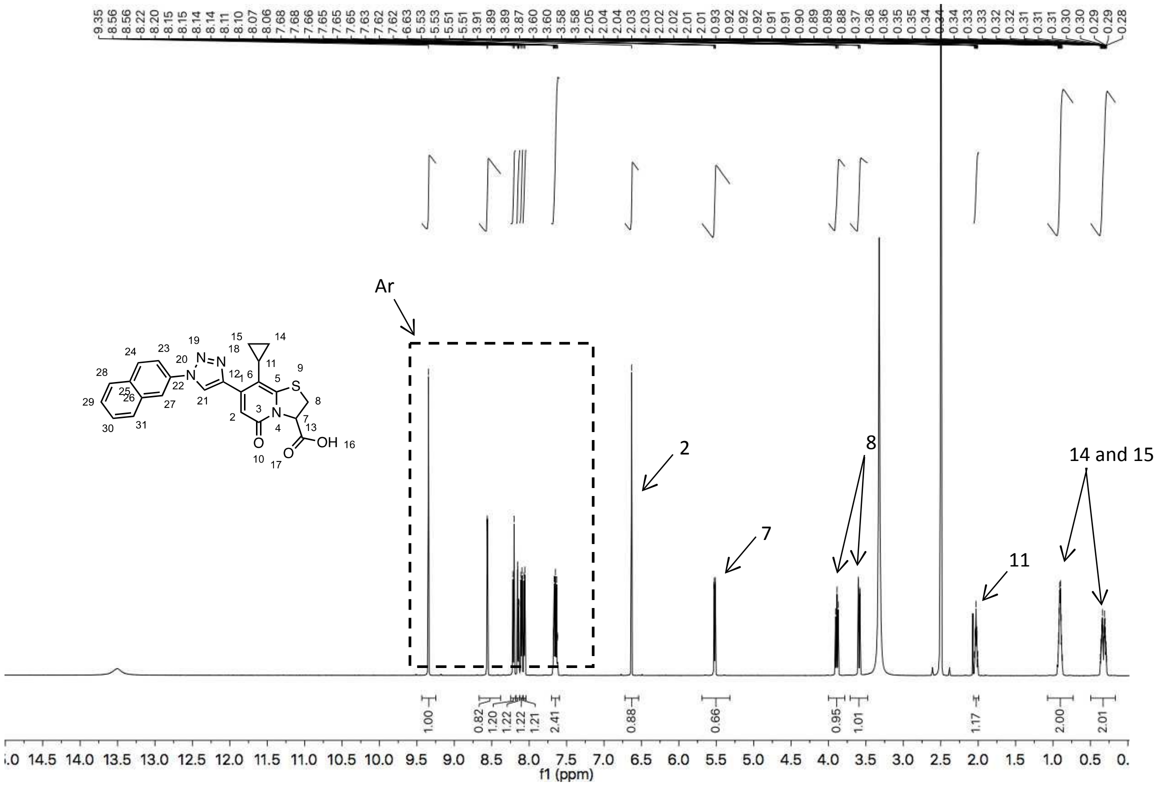

^{13}C NMR of **15c**

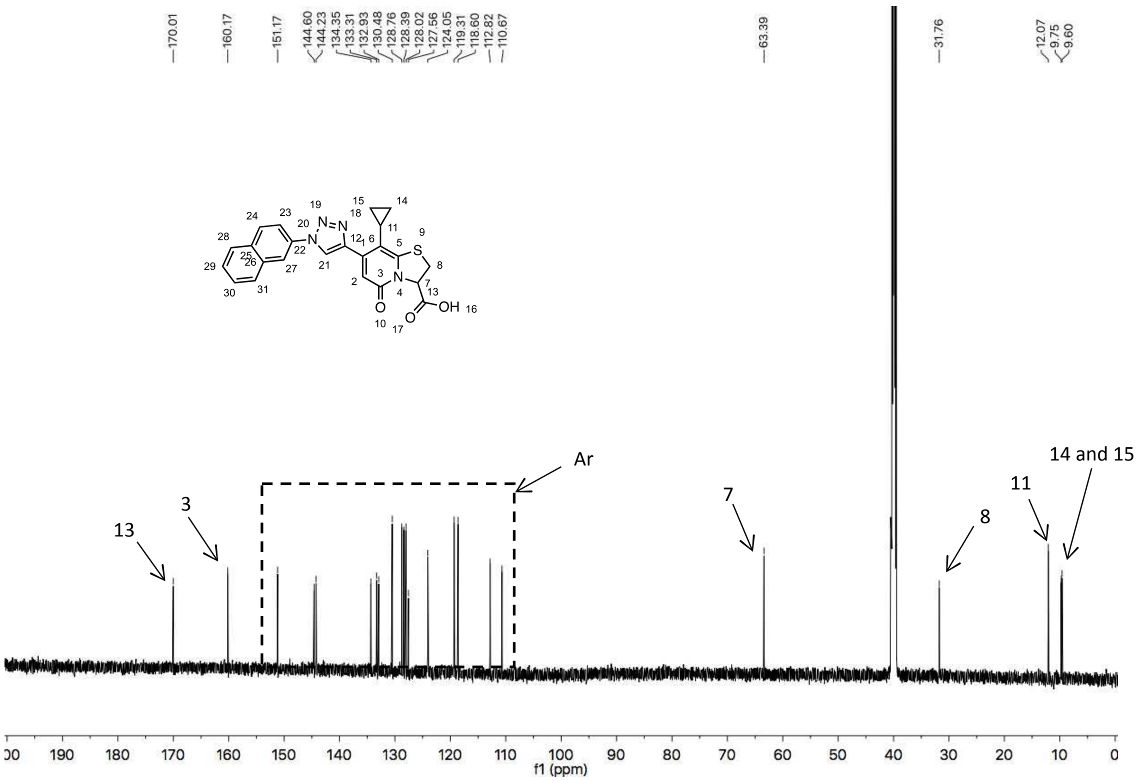

^{1}H NMR of **15d**

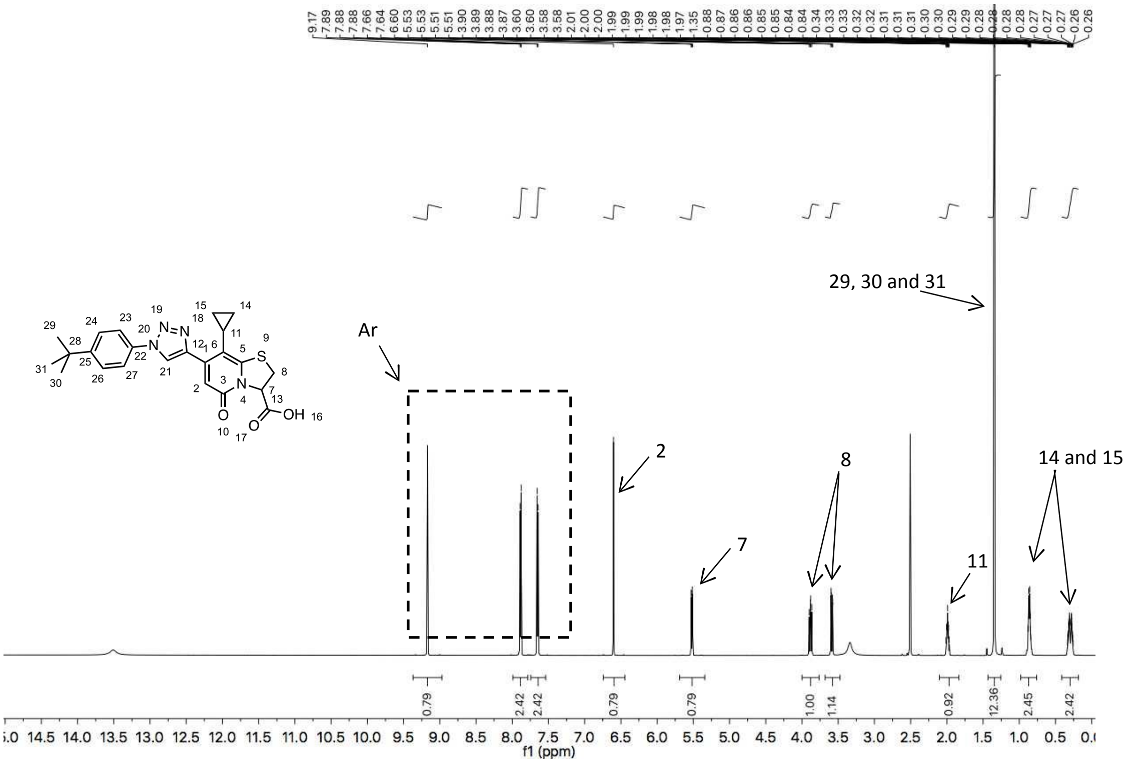

^{13}C NMR of **15d**

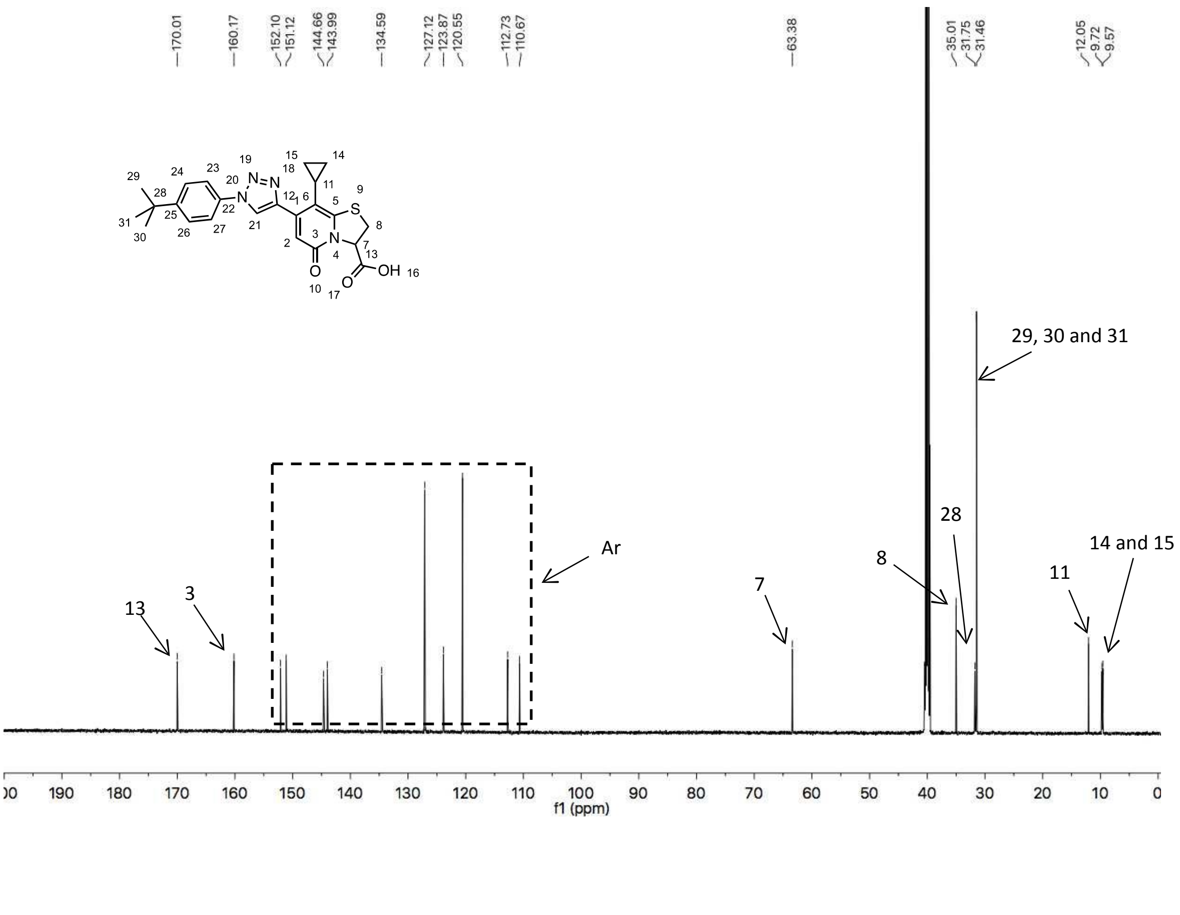

^{1}H NMR of **17a**

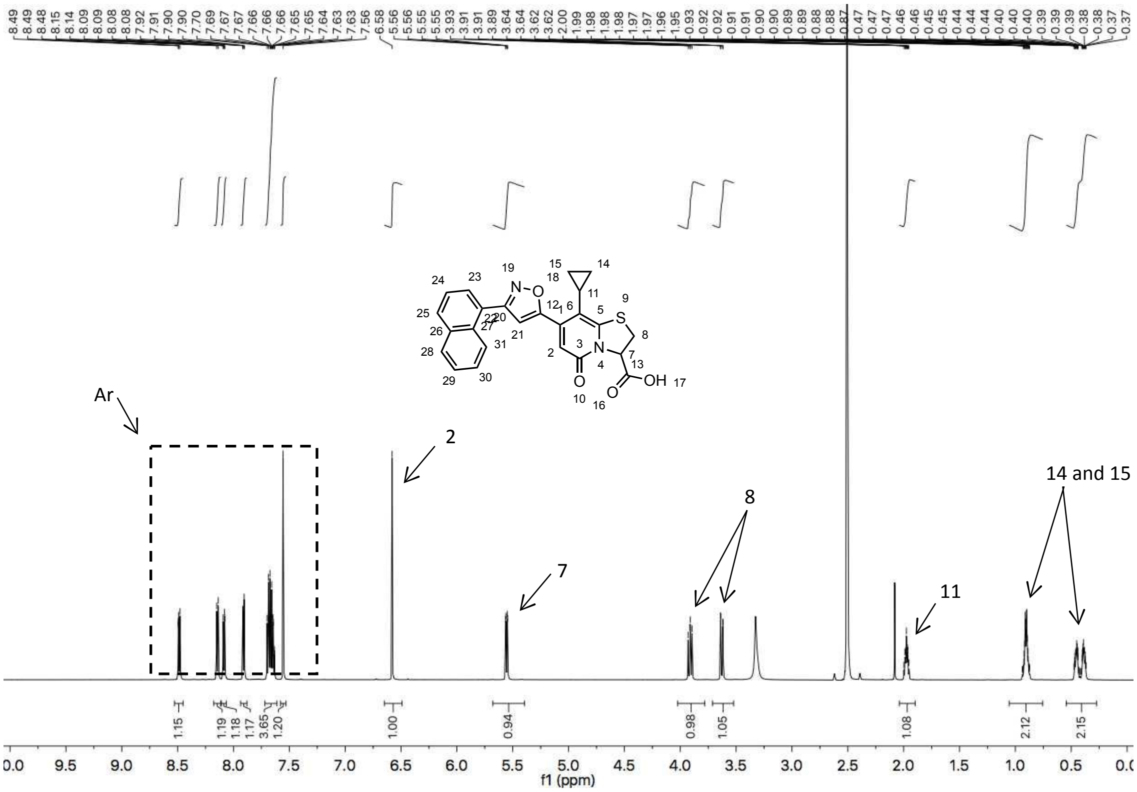

^{13}C NMR of **17a**

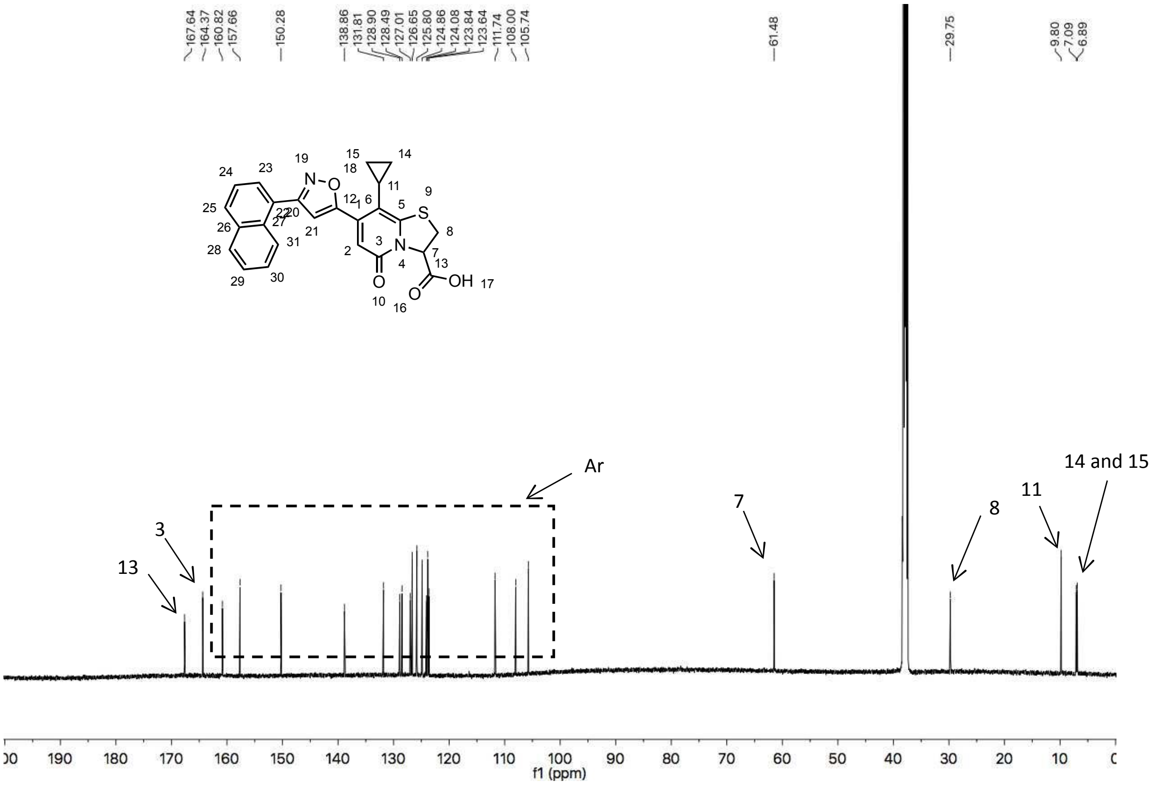

^{1}H NMR of **17b**

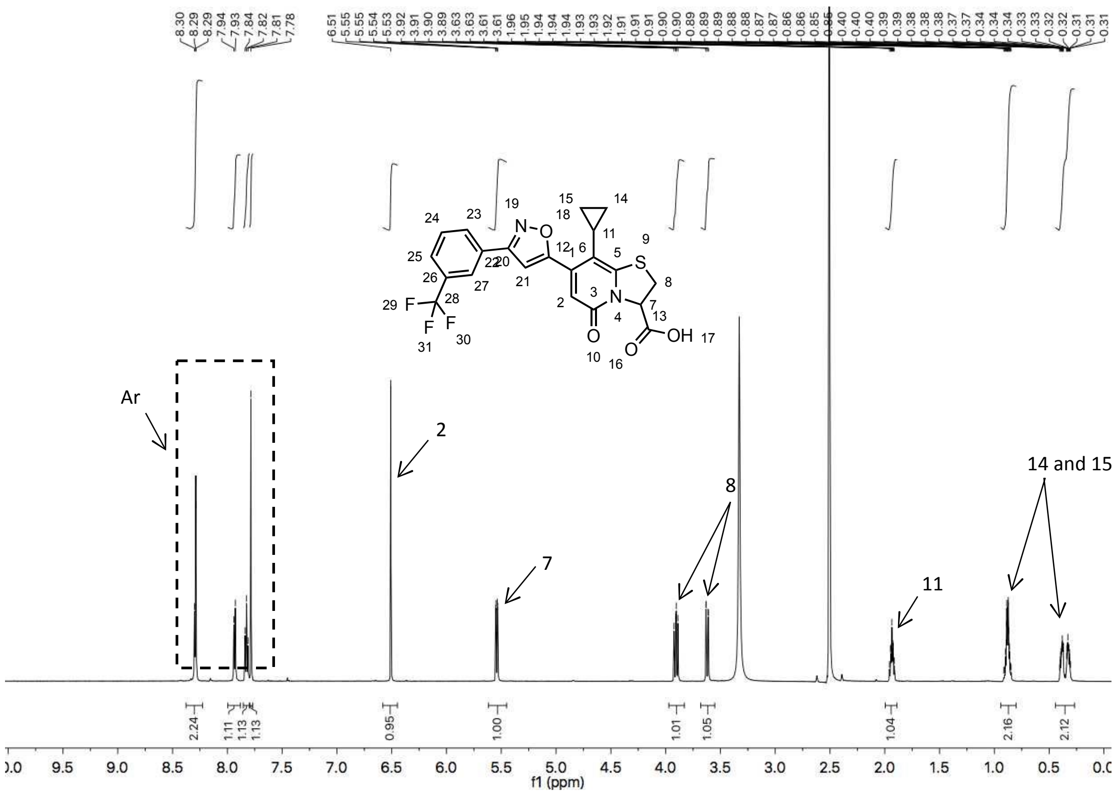

^{13}C NMR of **17b**

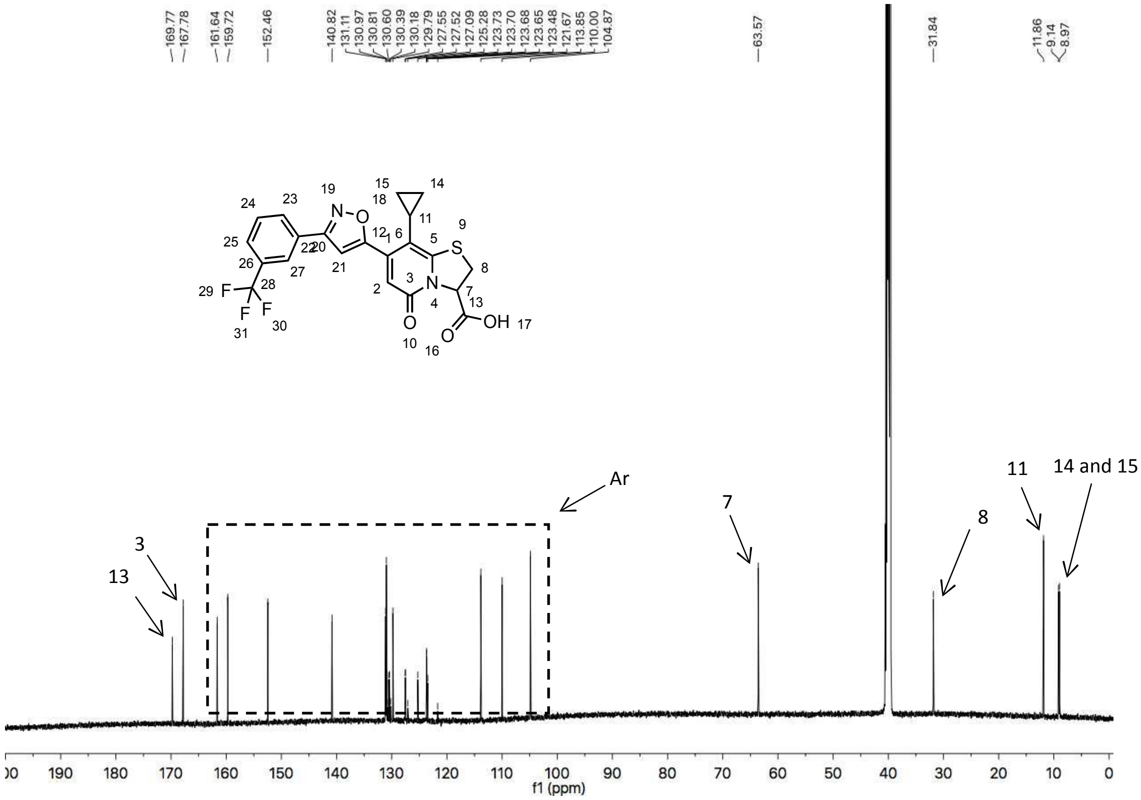

^{19}F NMR of **17b**

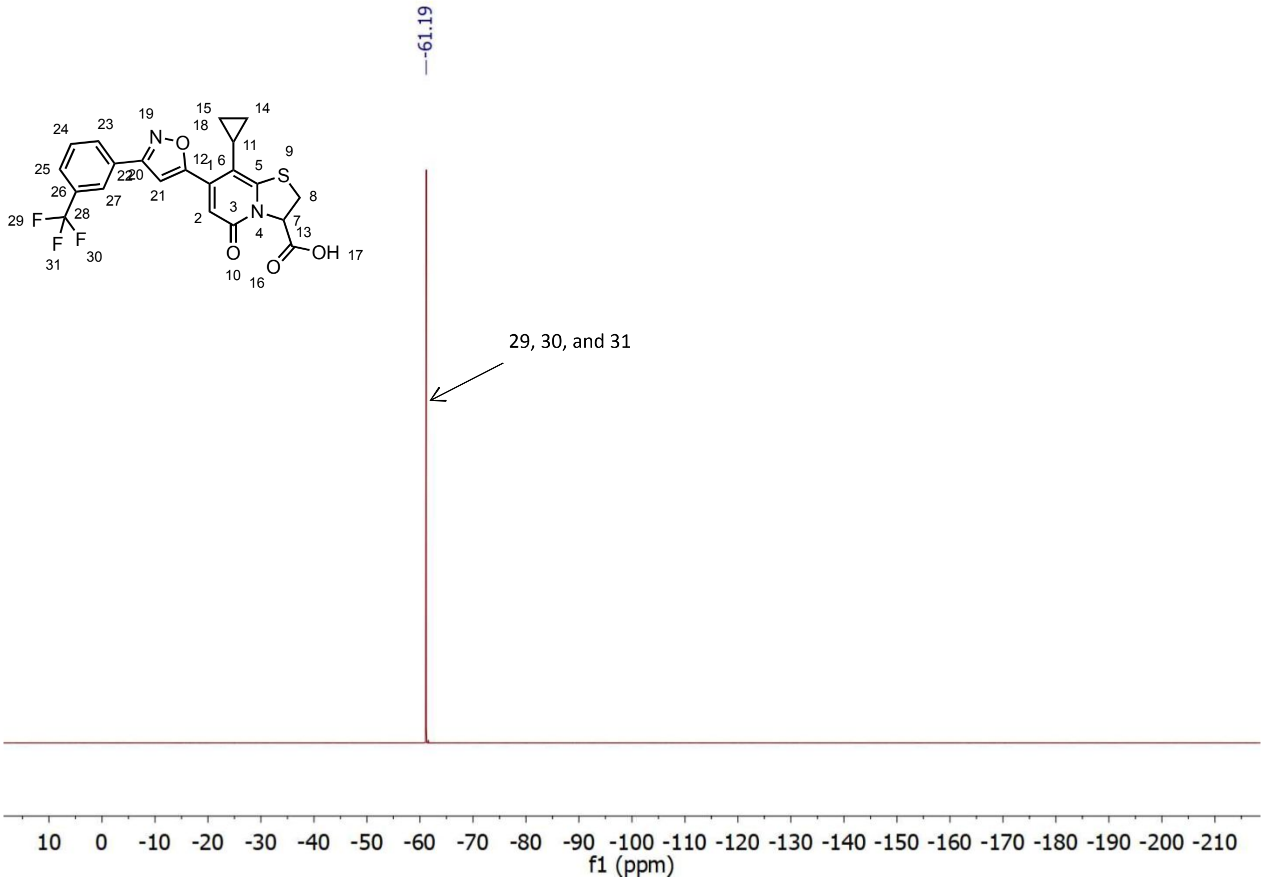

^{1}H NMR of **17c**

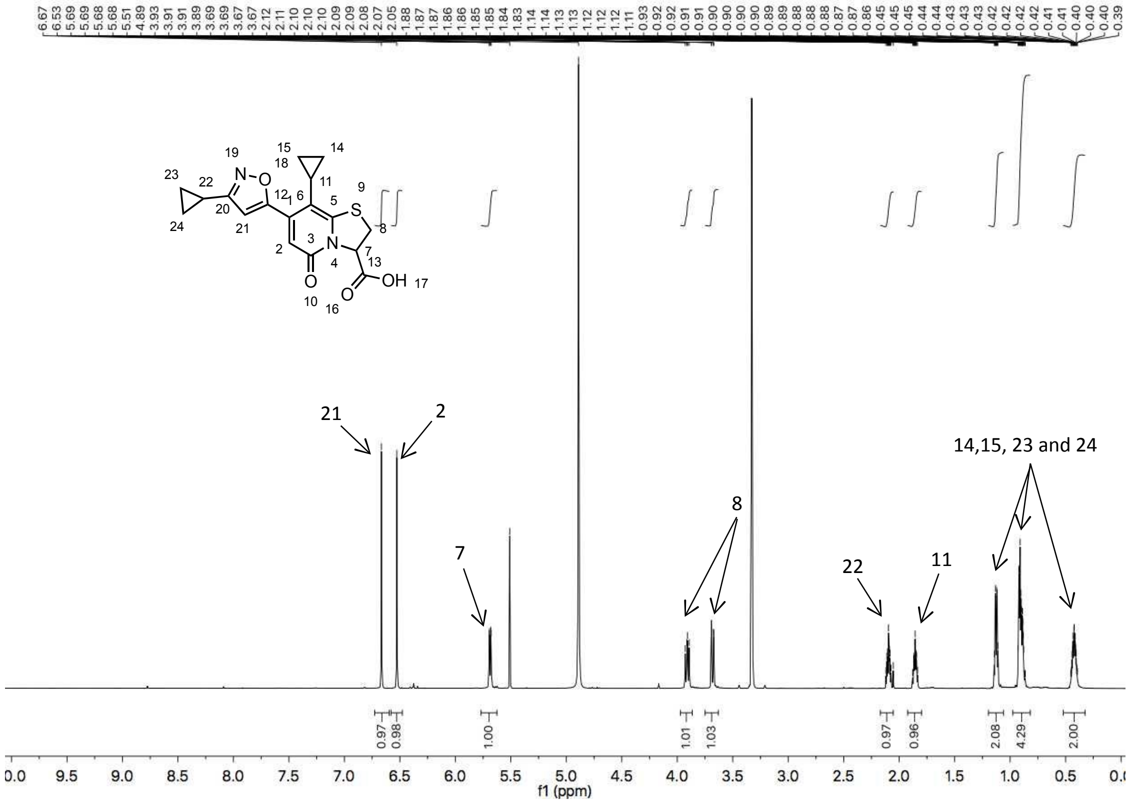

^{13}C NMR of **17c**

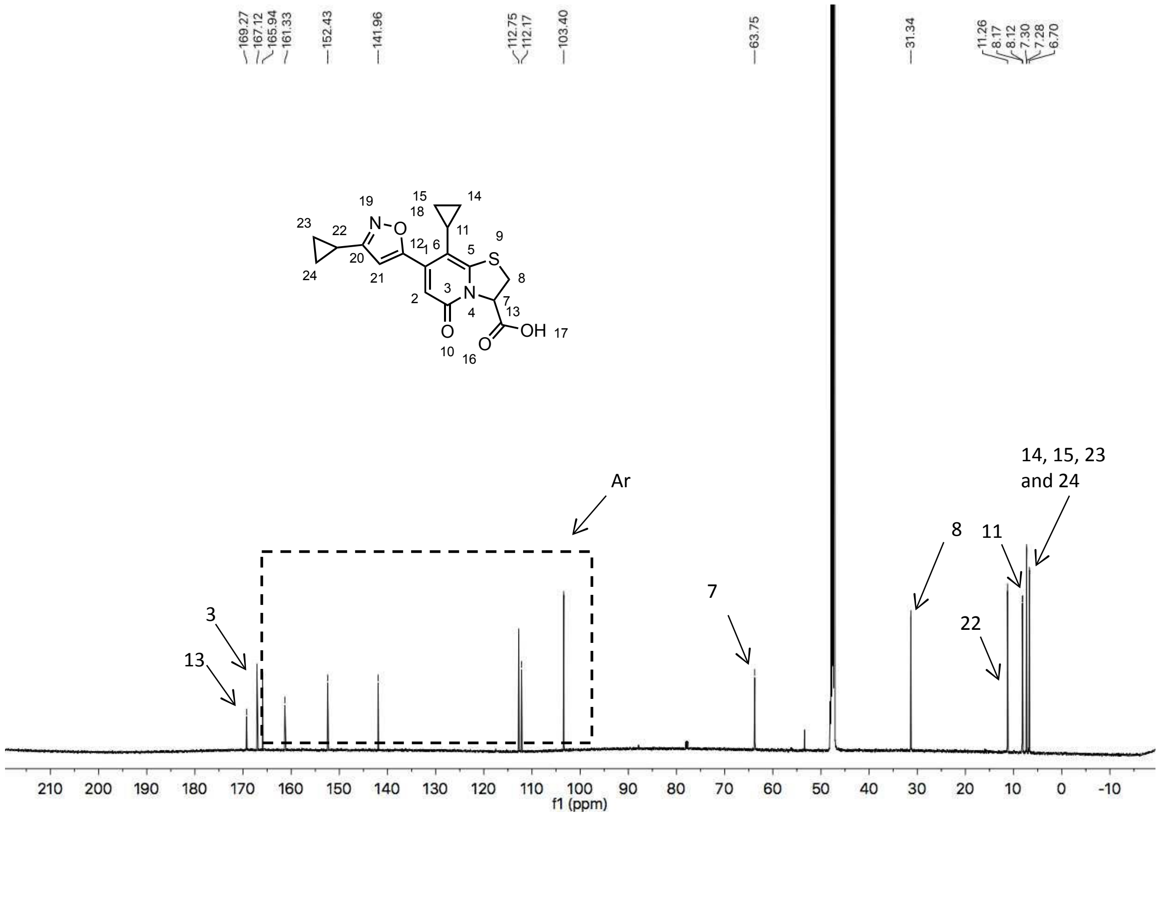

^{1}H NMR of **17d**

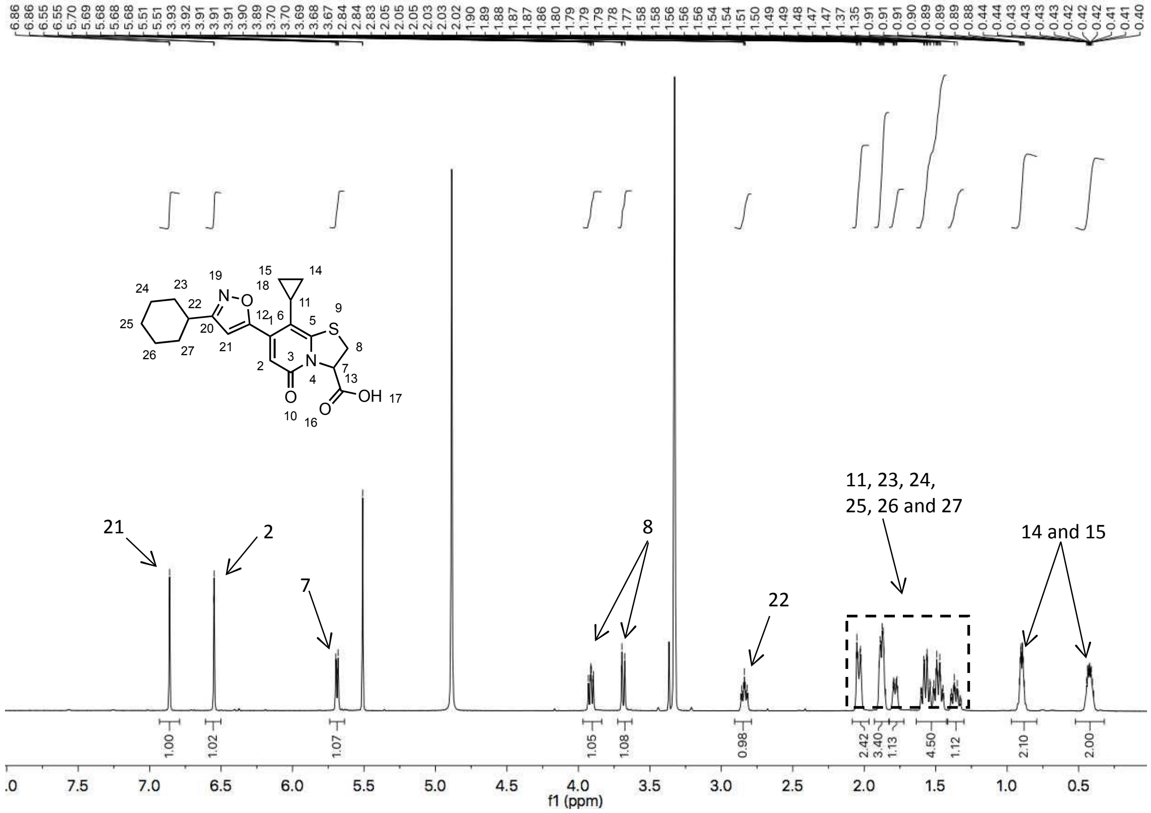

^{13}C NMR of **17d**

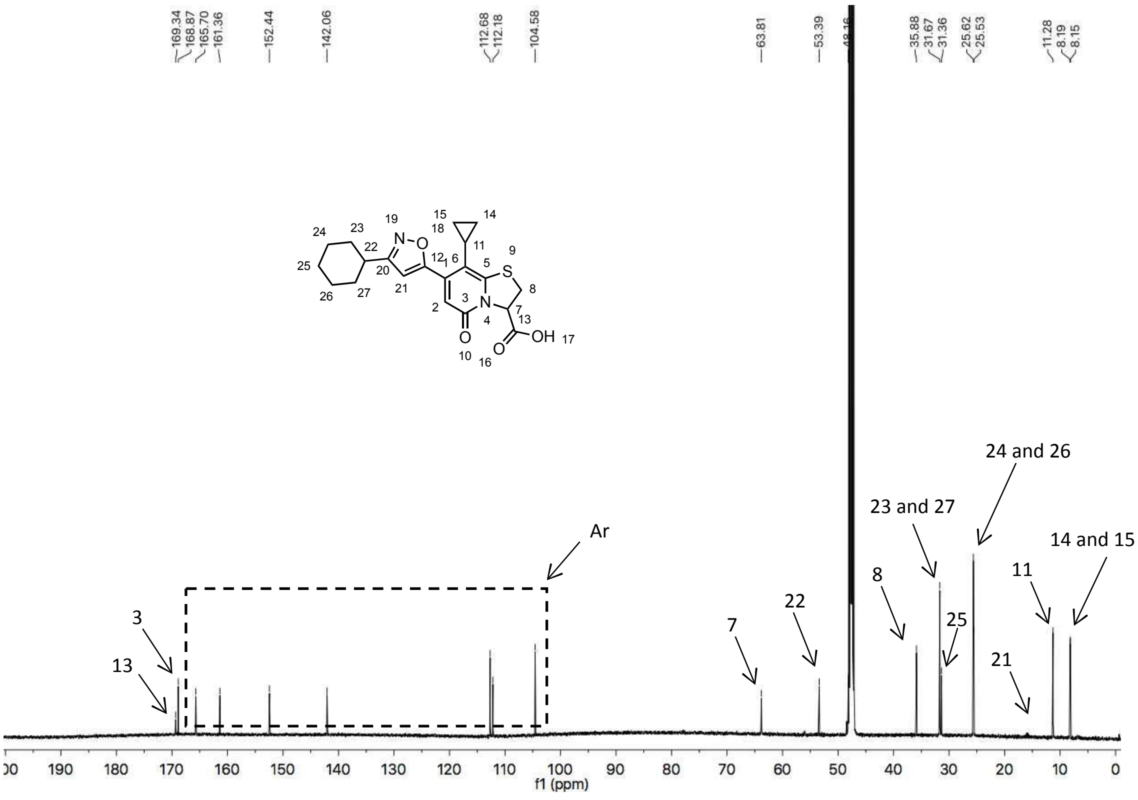

^1H NMR of **17e**

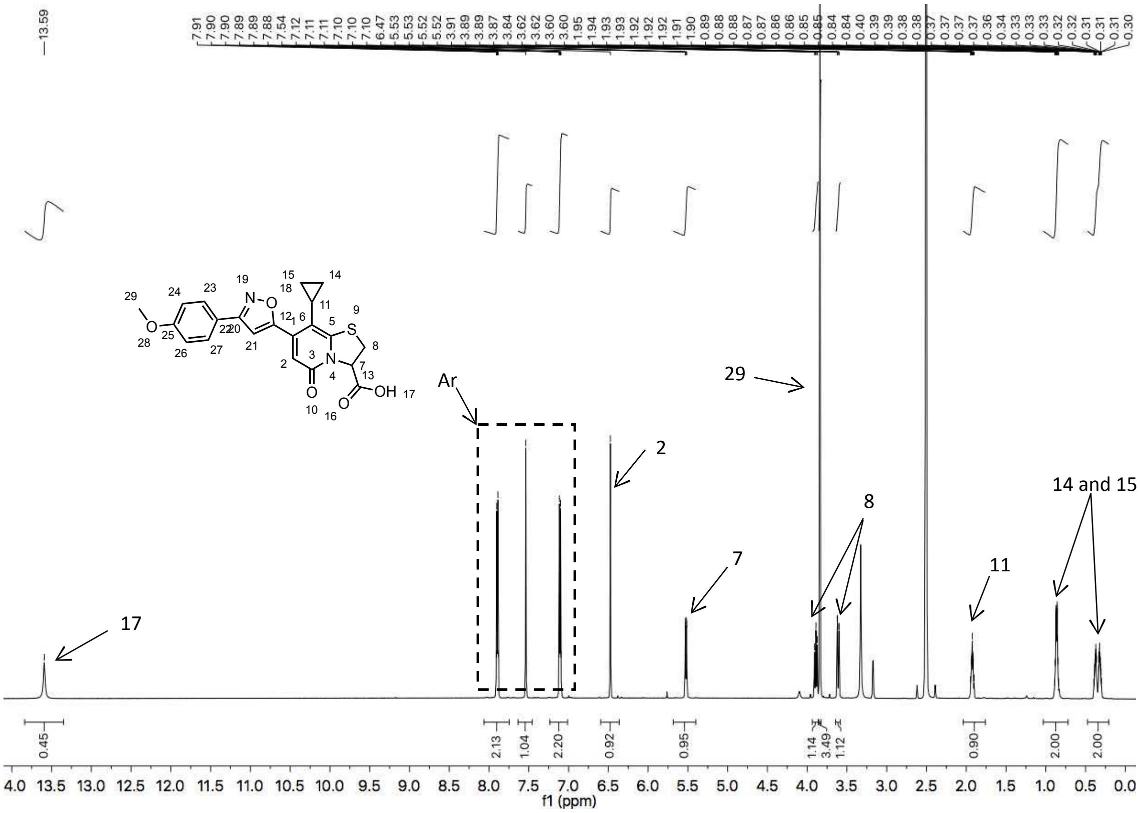

^{13}C NMR of **17e**

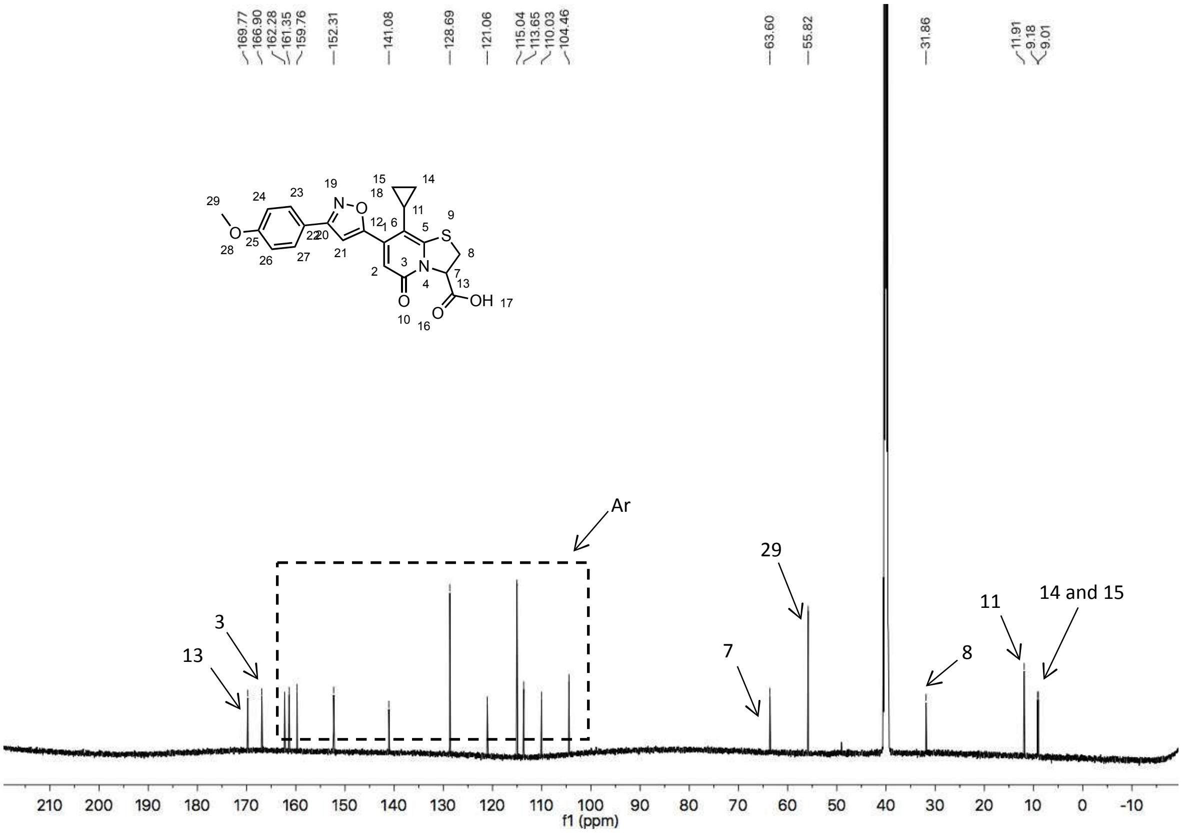

^{1}H NMR of **17f**

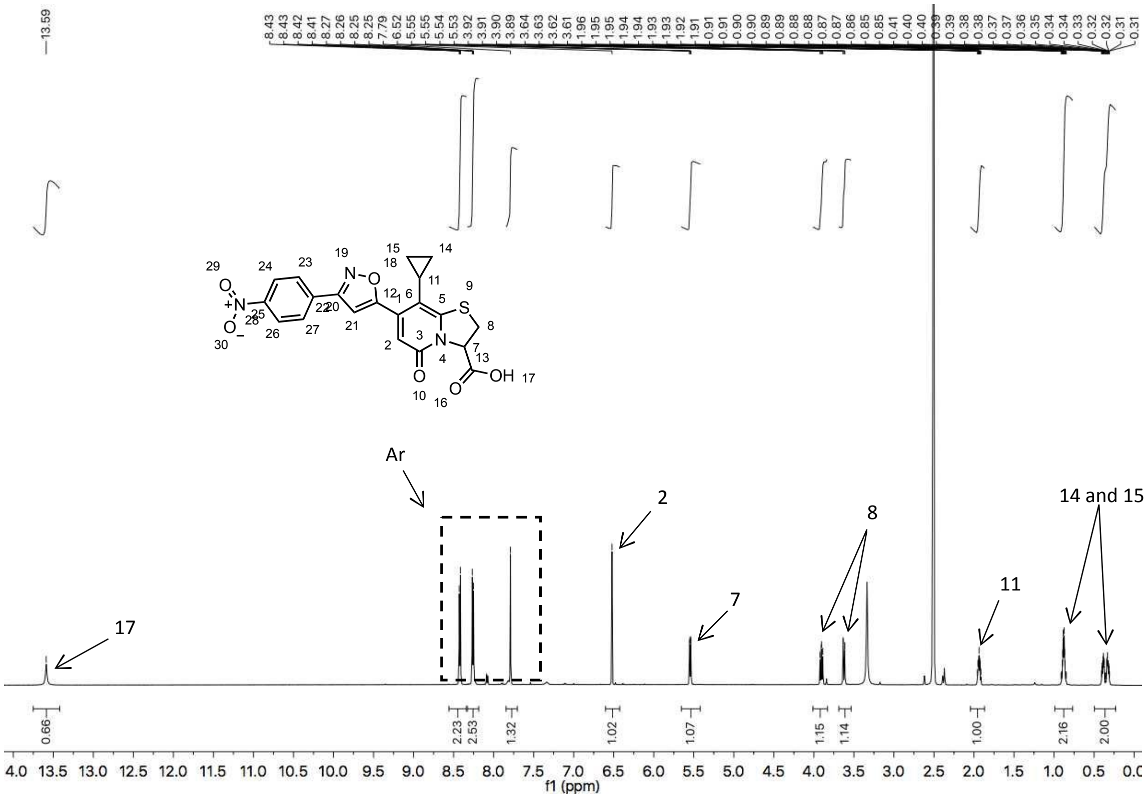

^{13}C NMR of **17e**

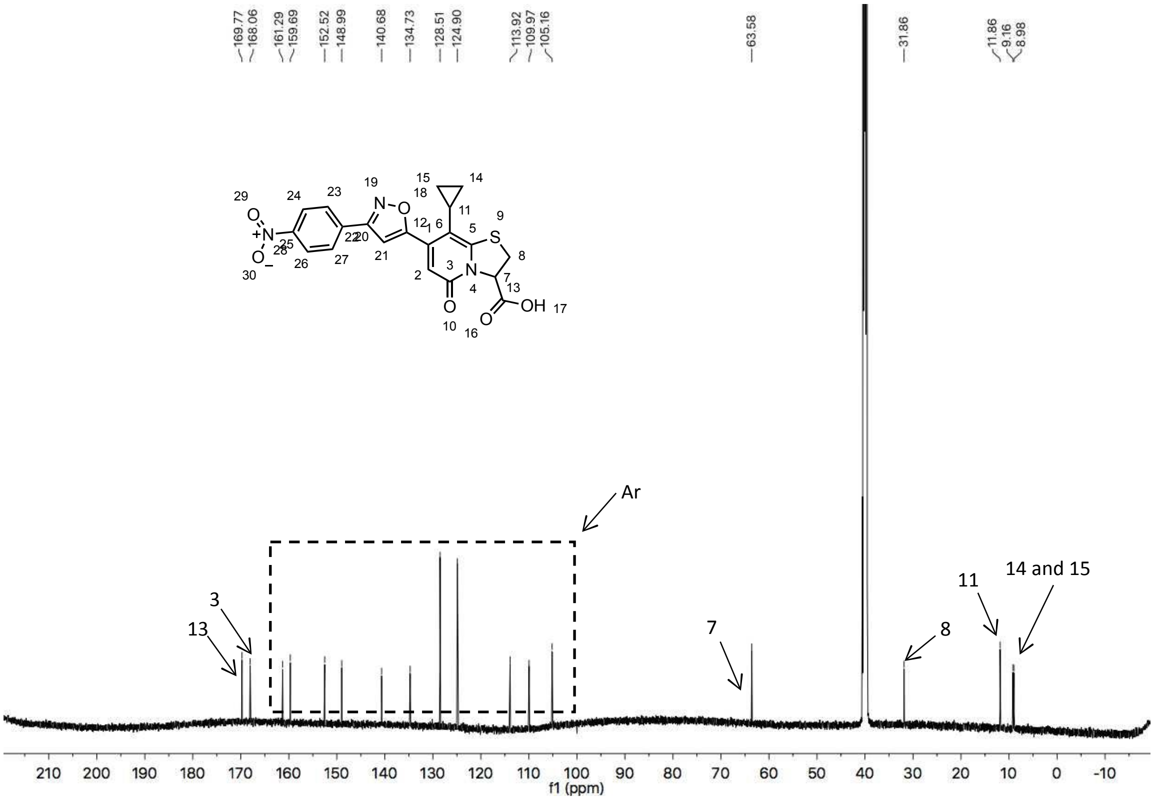

^{1}H NMR of **17g**

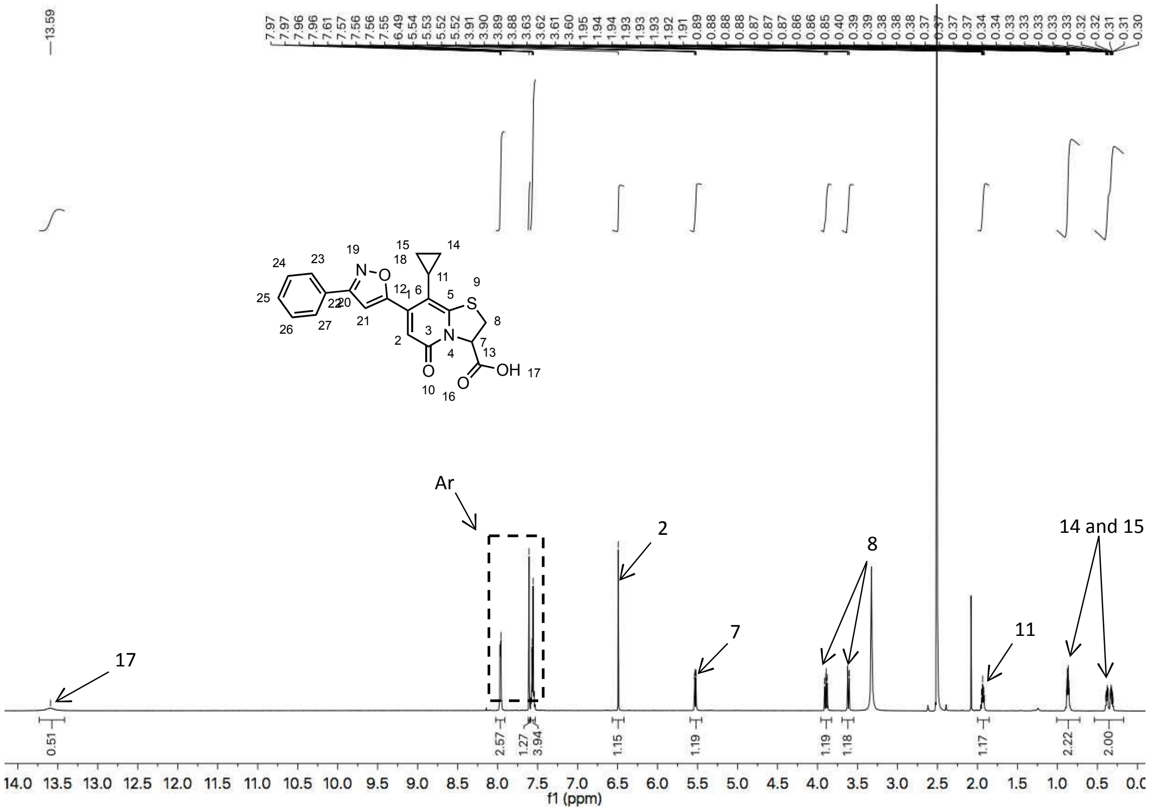

^{13}C NMR of **17g**

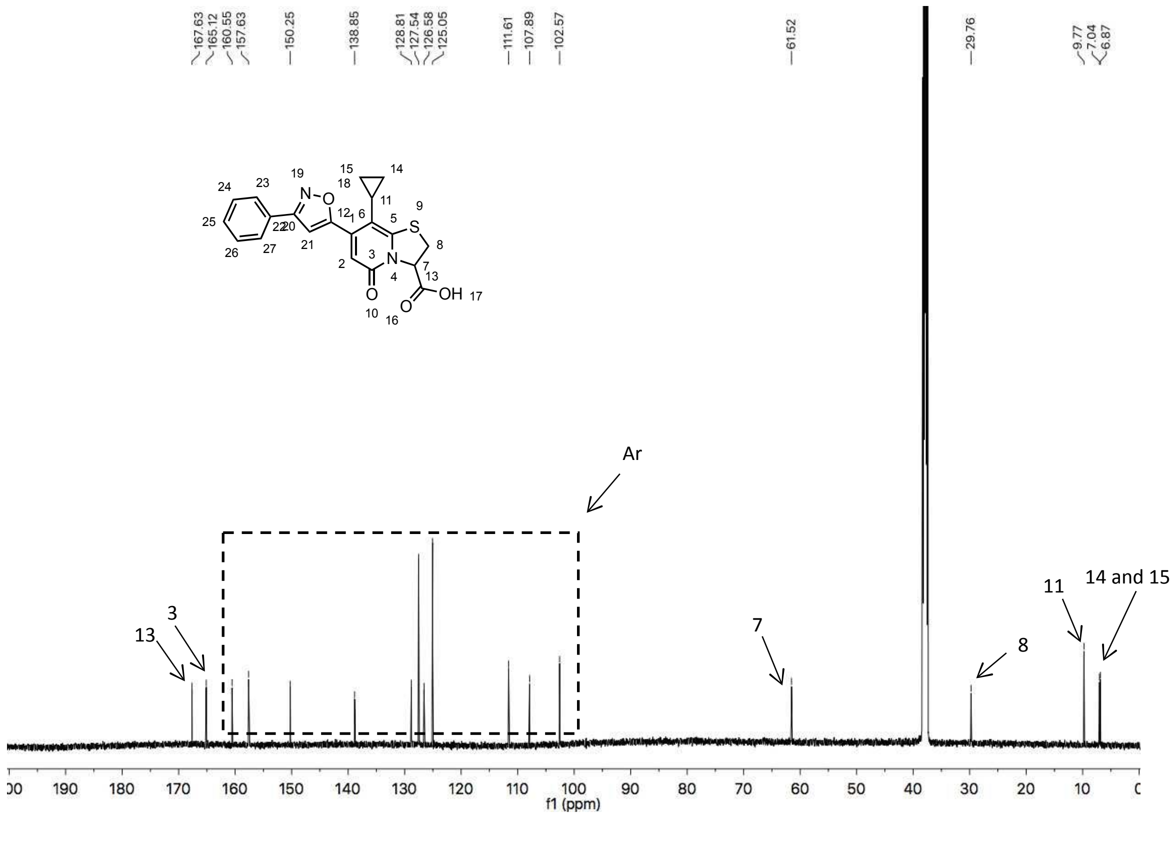

^{1}H NMR of **17h**

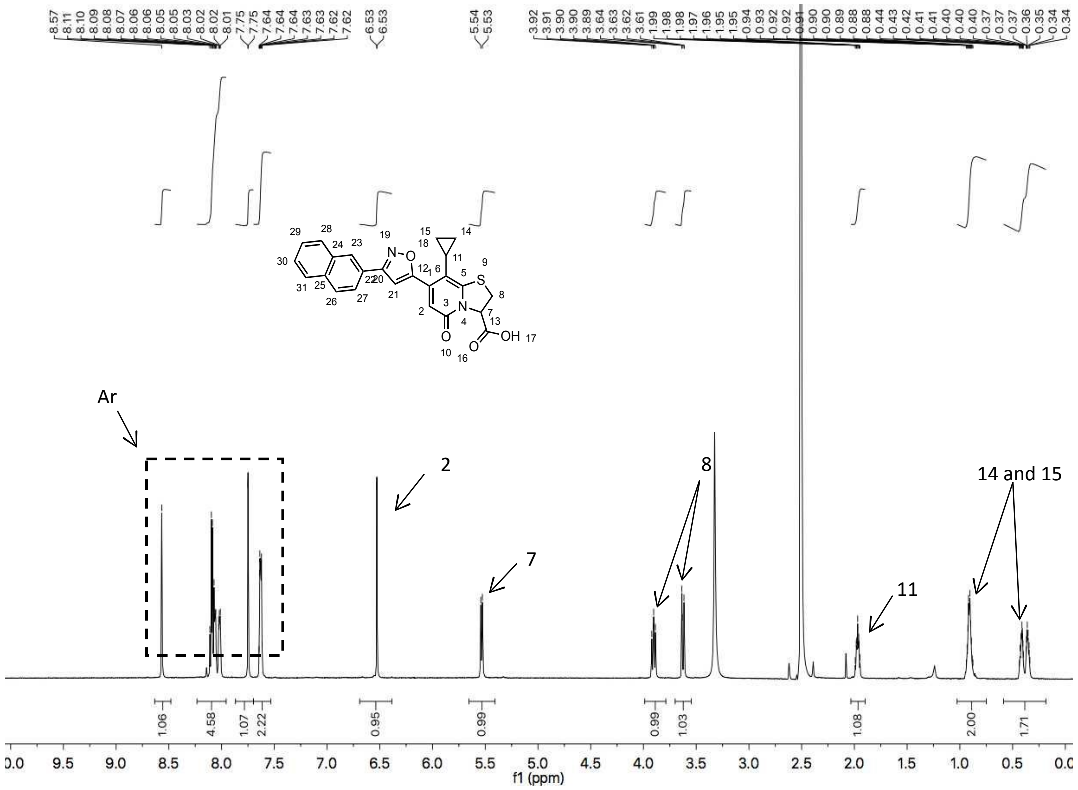

^{13}C NMR of **17h**

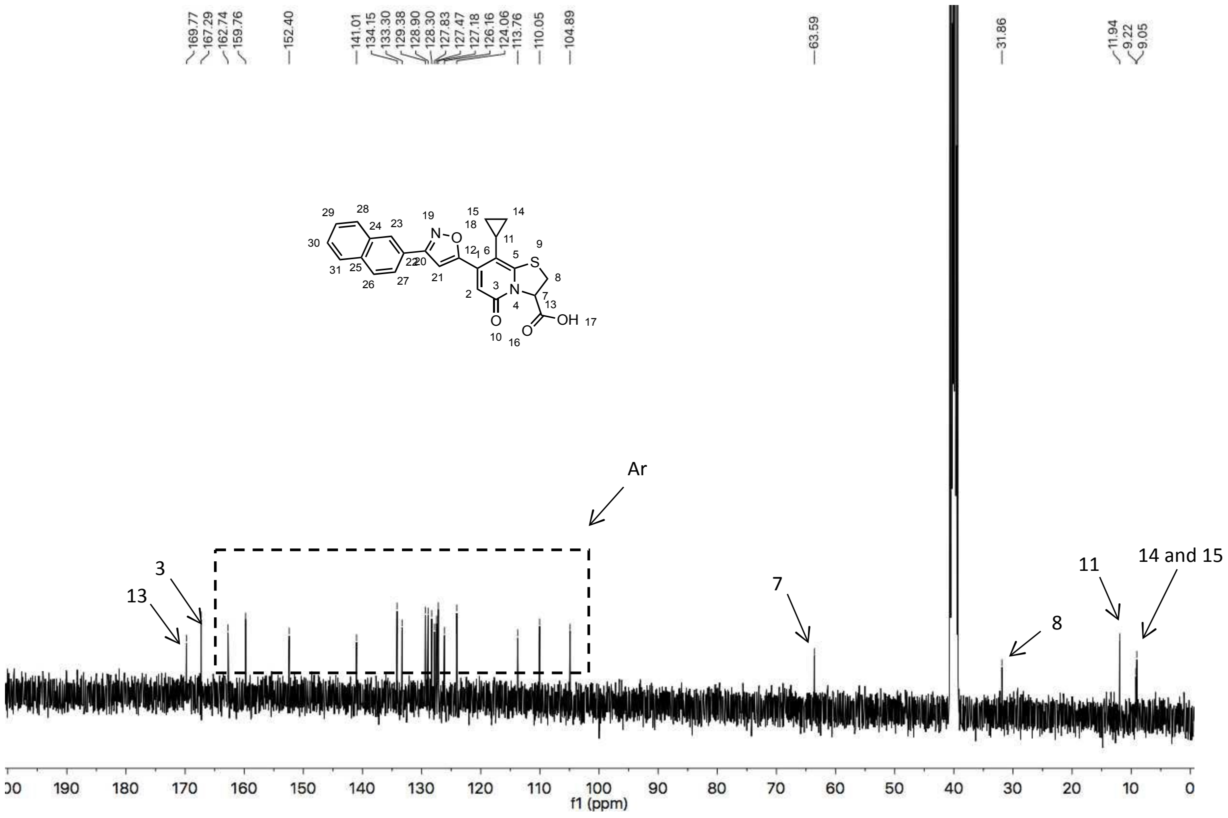

^{1}H NMR of **17i**

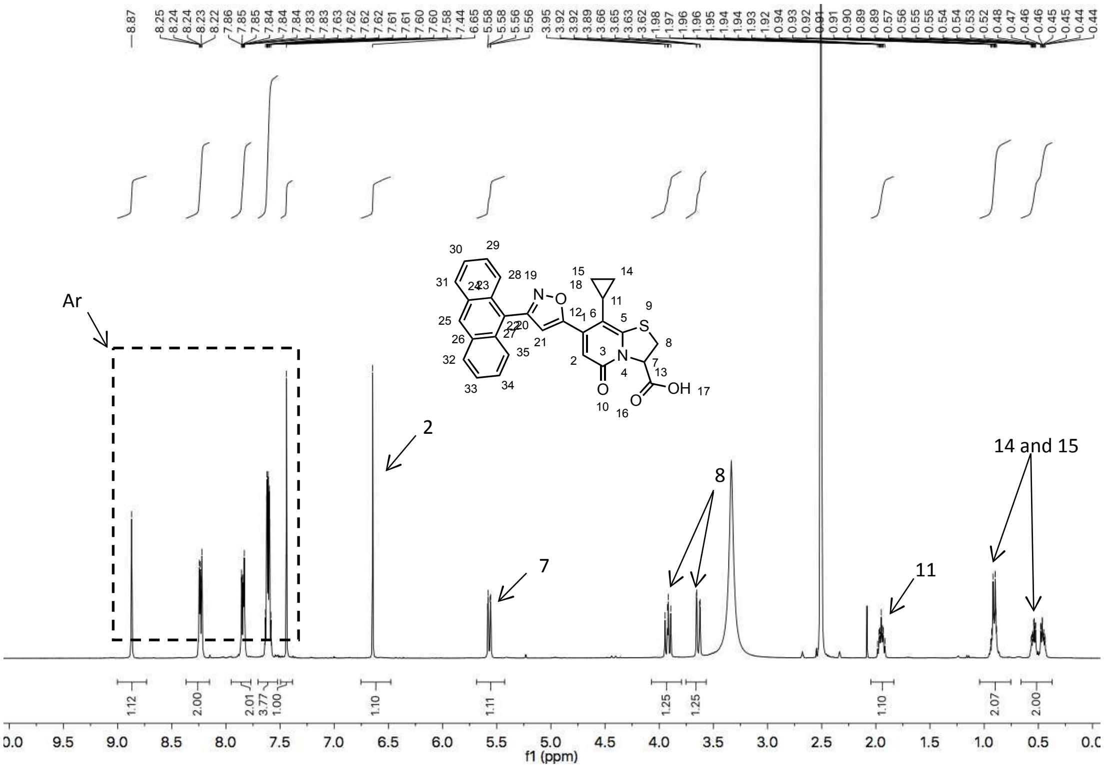

^{13}C NMR of **17i**

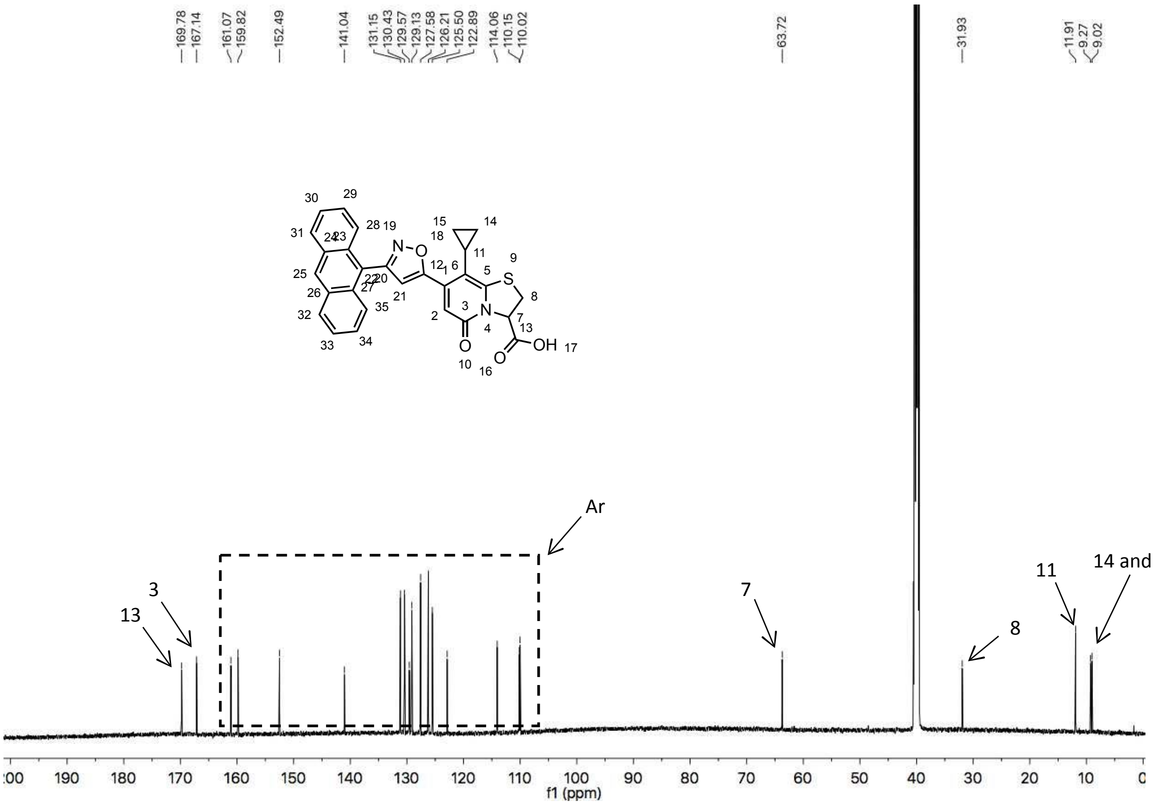

^{1}H NMR of **17j**

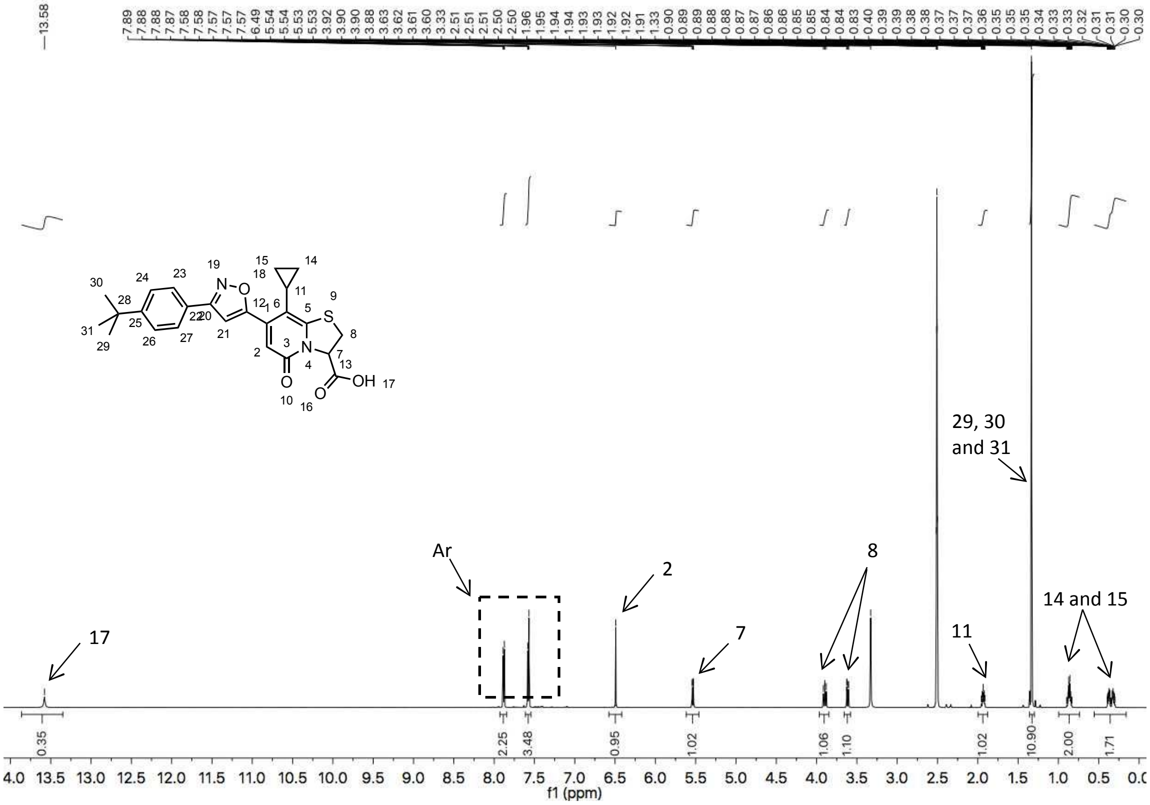

^{13}C NMR of **17j**

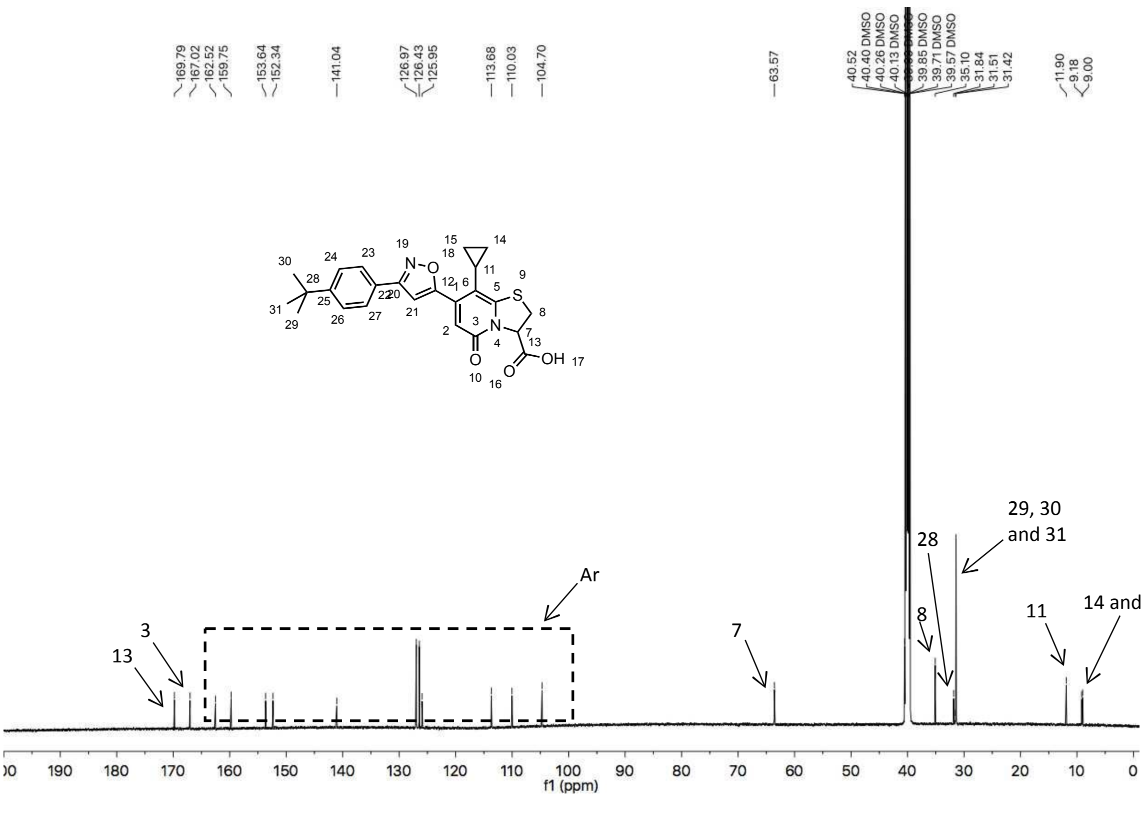

^{1}H NMR of **17k**

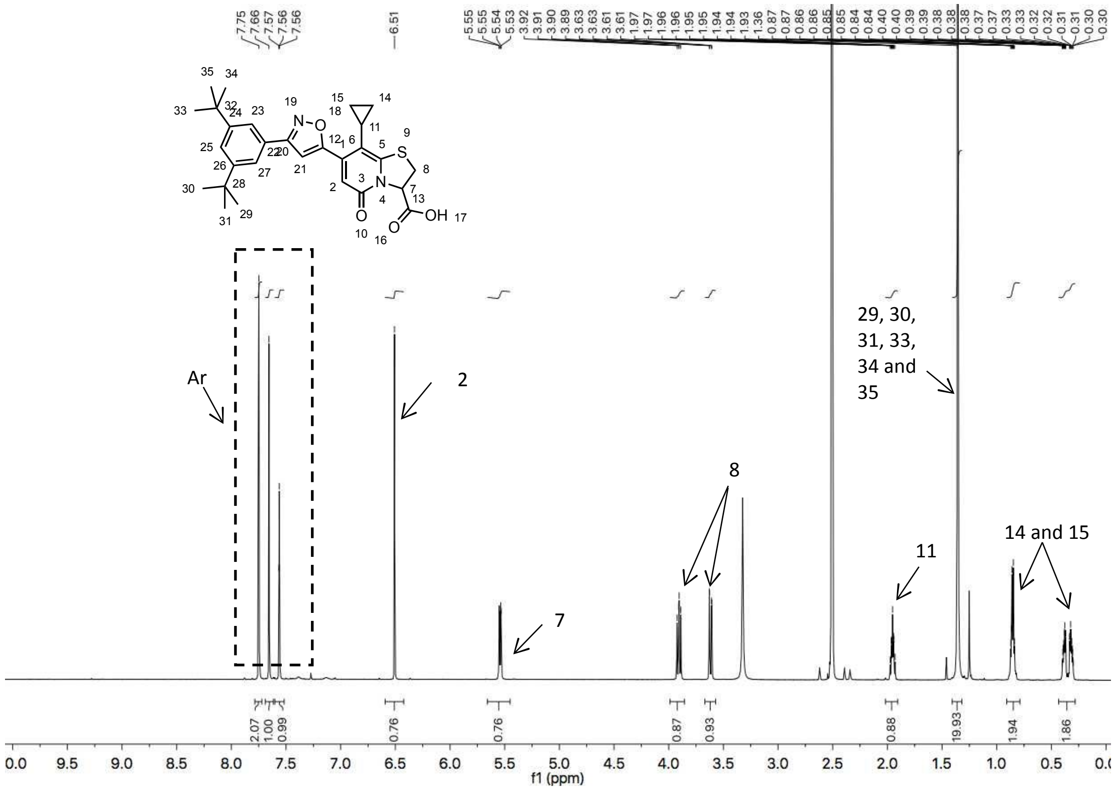

^{13}C NMR of **17k**

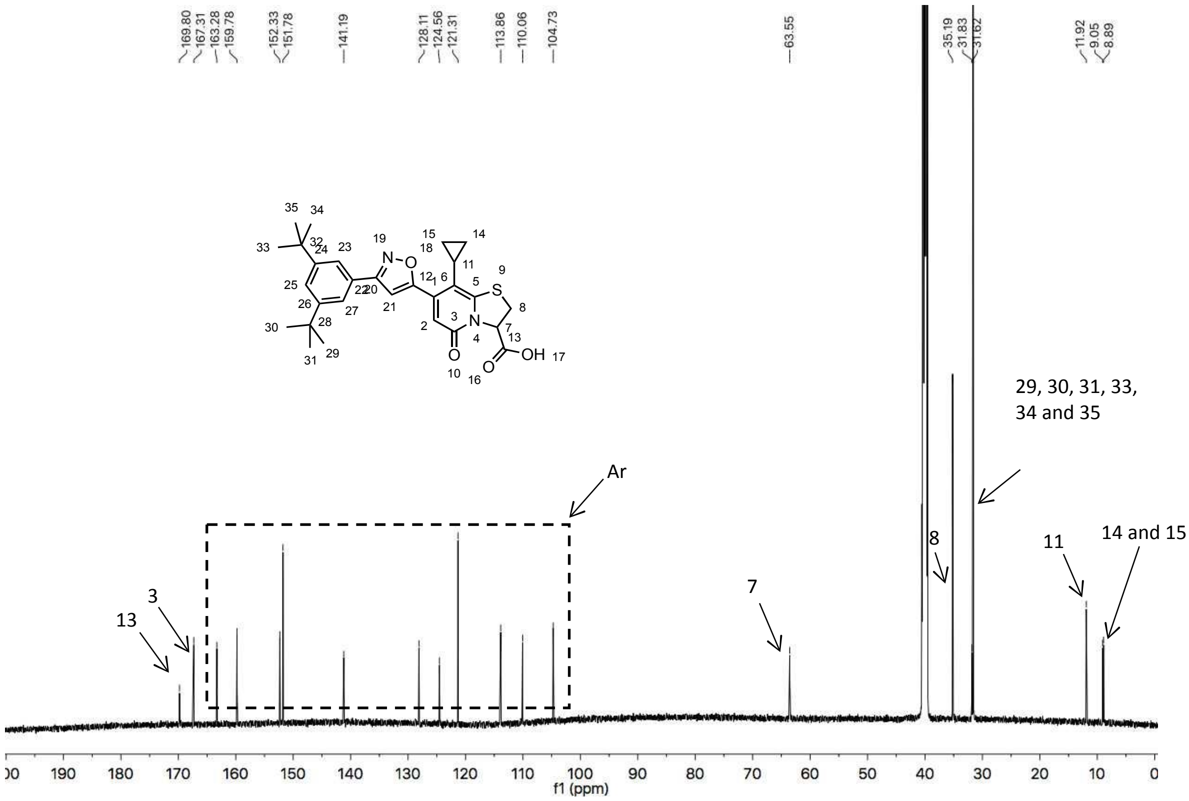

^{1}H NMR of **17l**

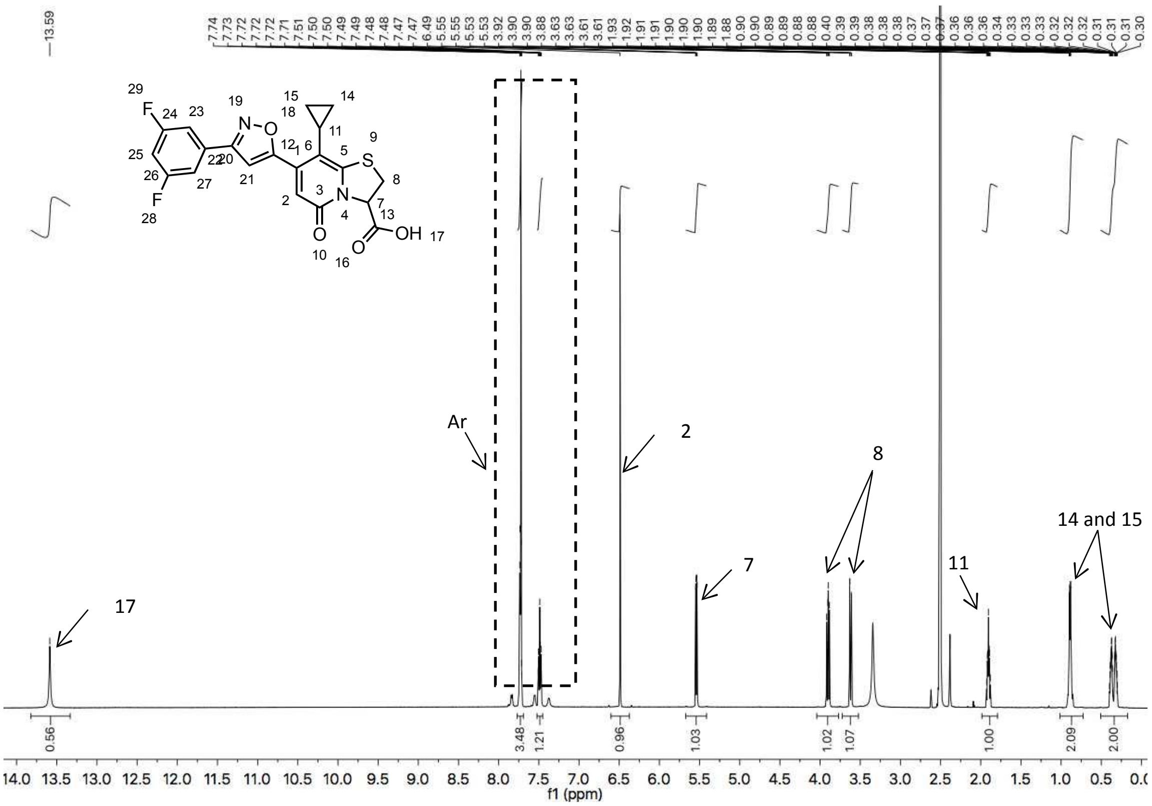

^{13}C NMR of **17l**

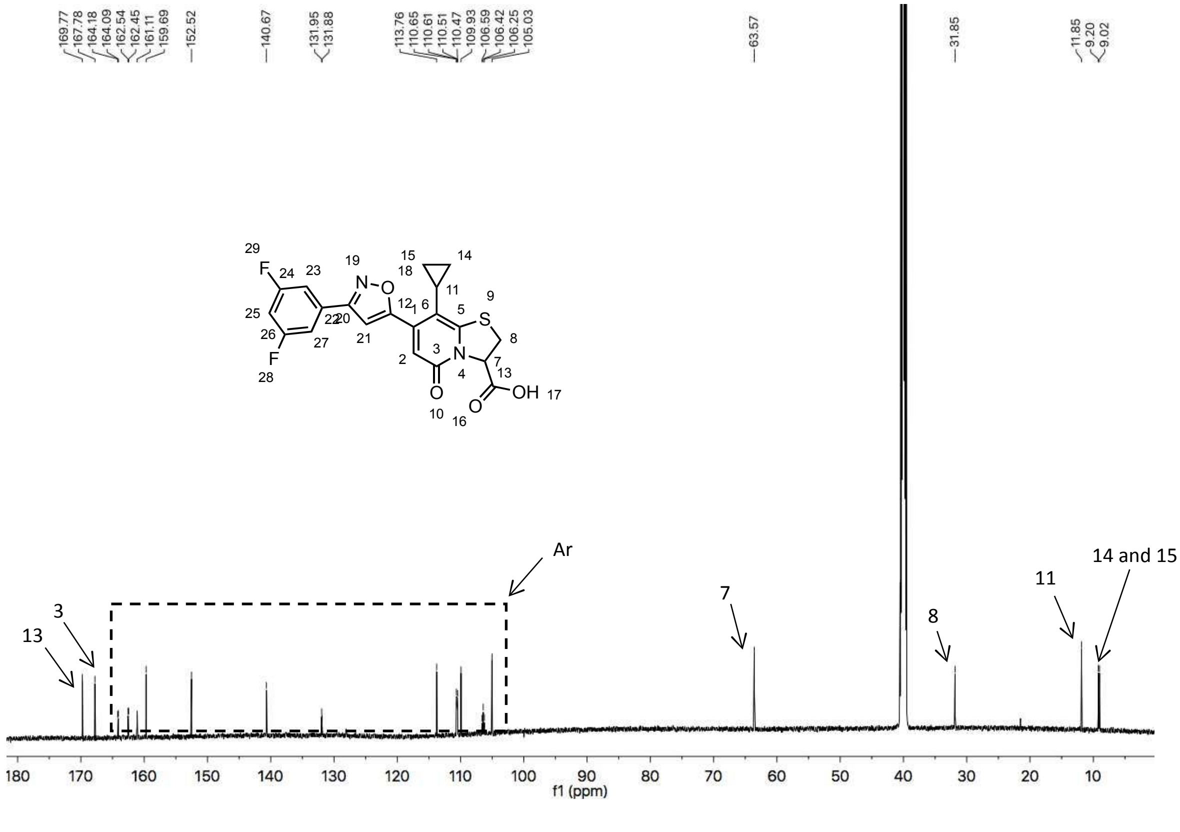

^{19}F NMR of **17l**

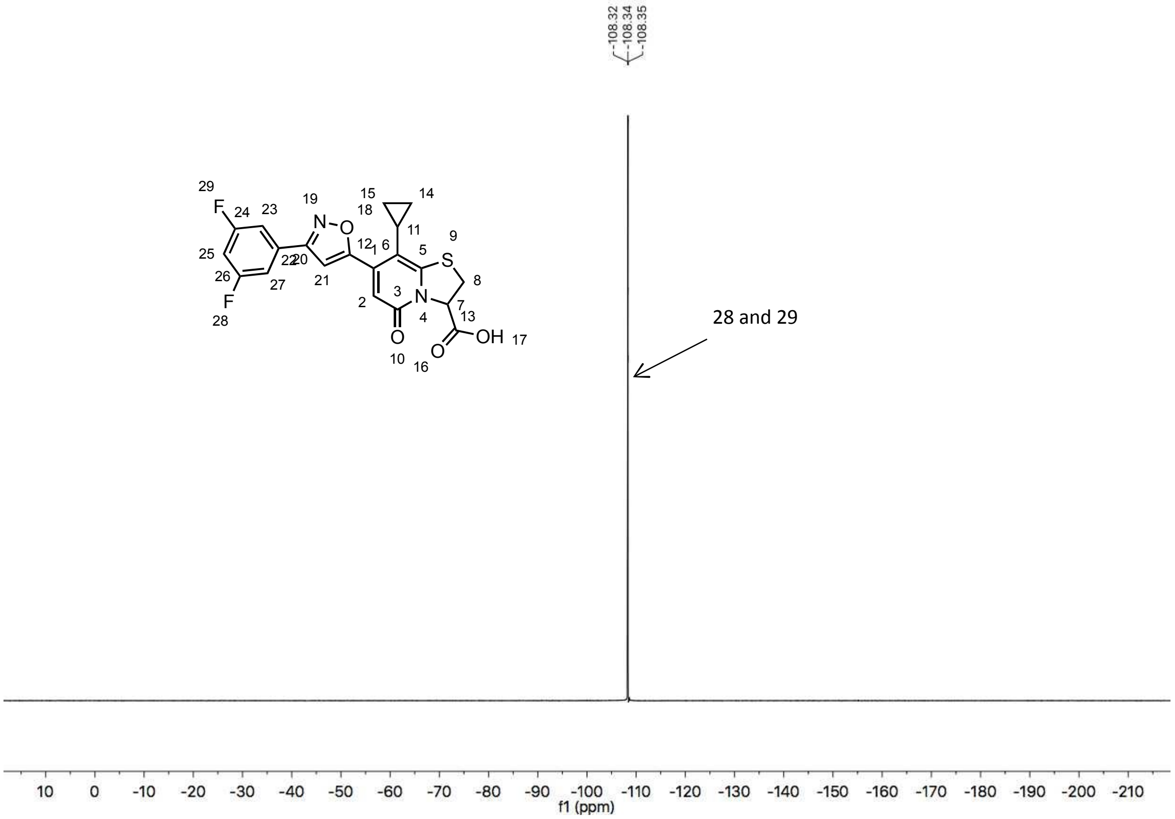

^{1}H NMR of **17m**

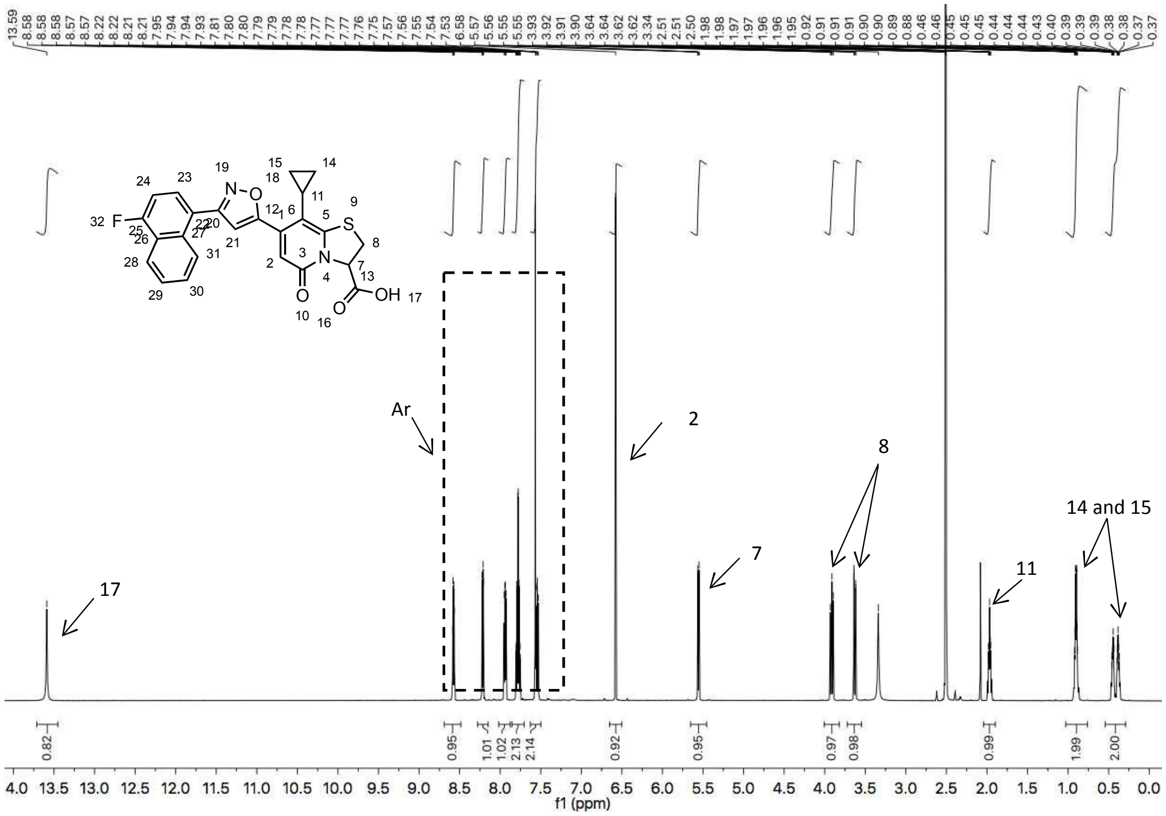

^{13}C NMR of **17m**

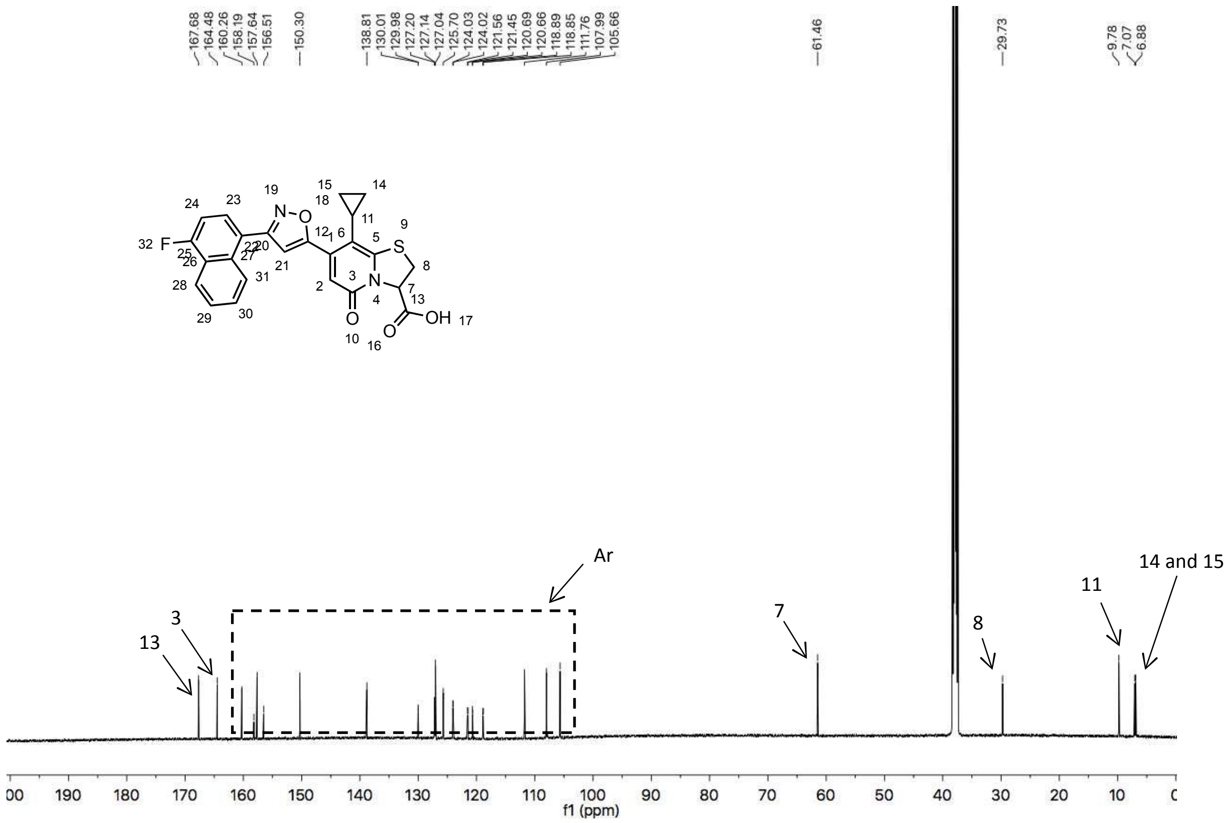

^{19}F NMR of **17m**

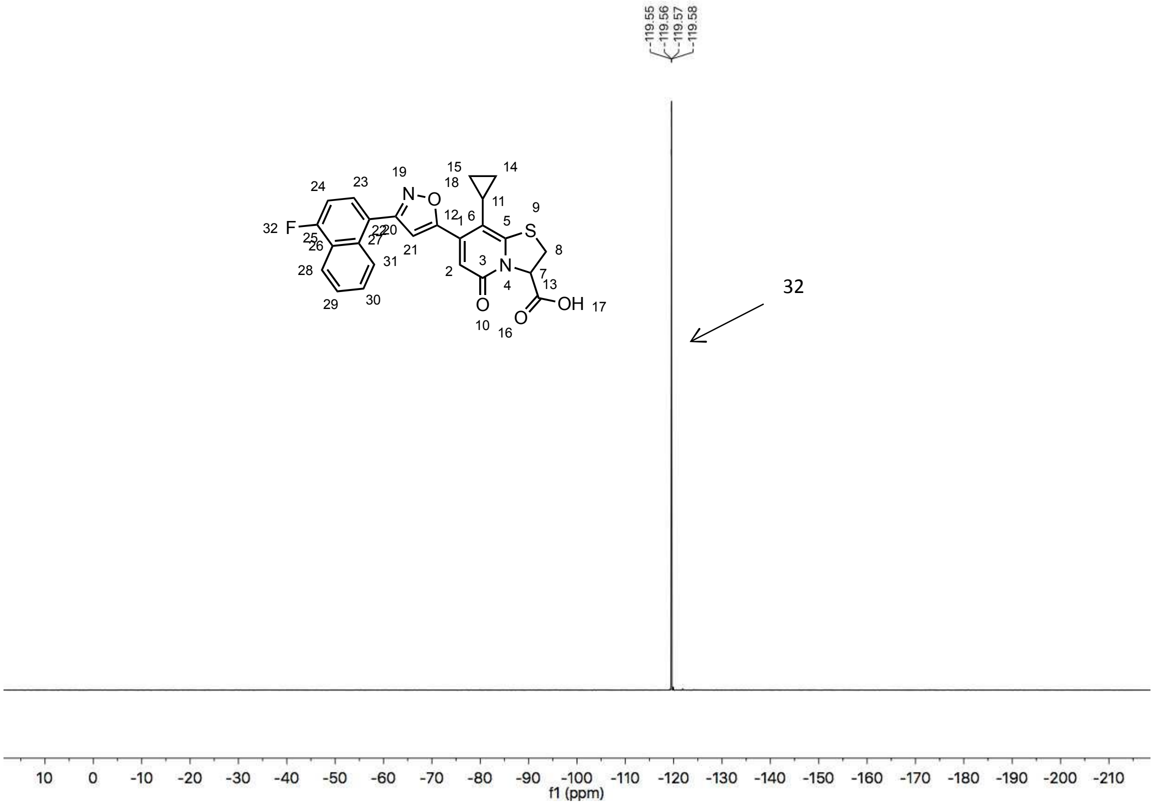

^{1}H NMR of **17n**

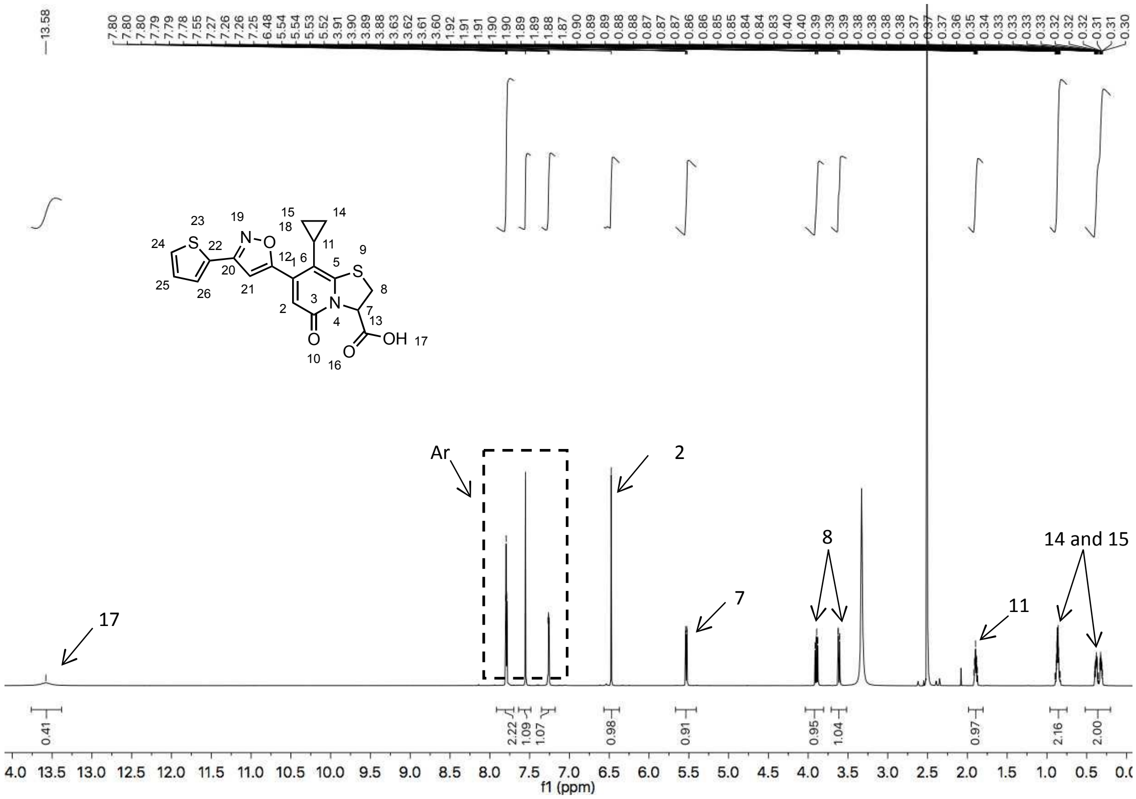

^{13}C NMR of **17n**

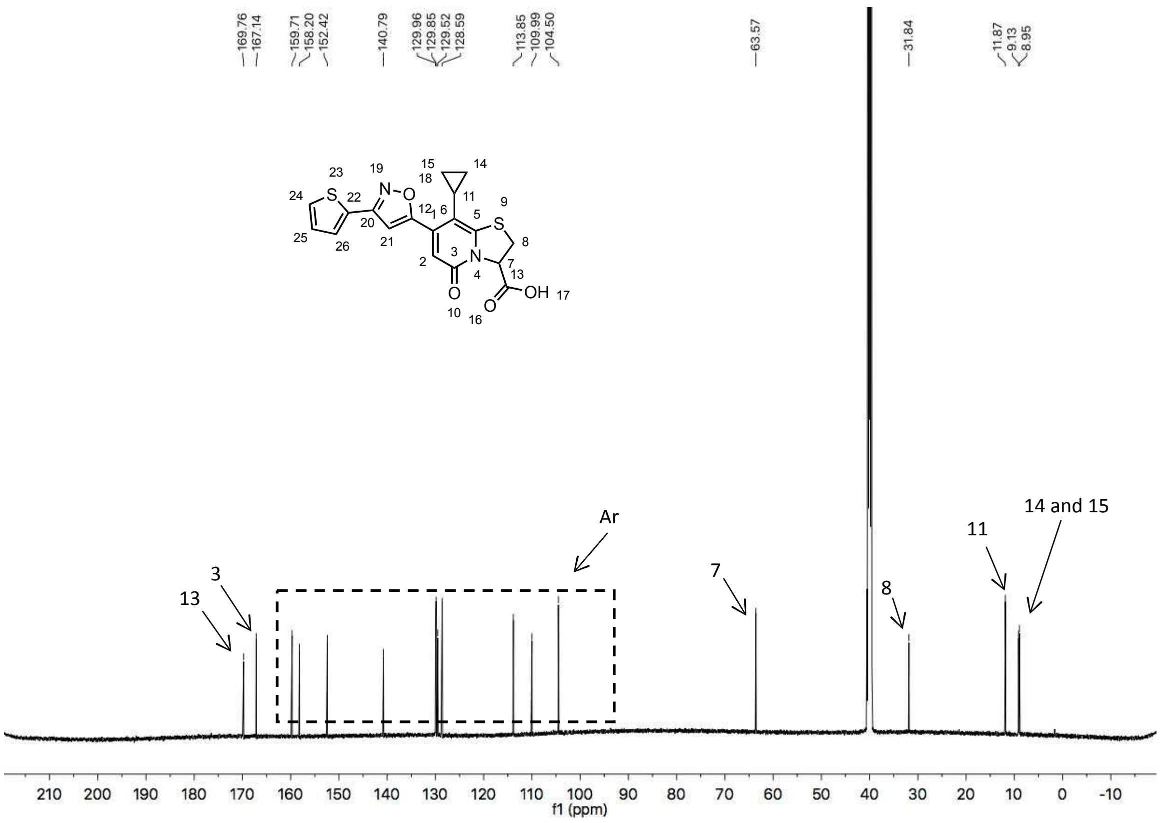

^{1}H NMR of **17o**

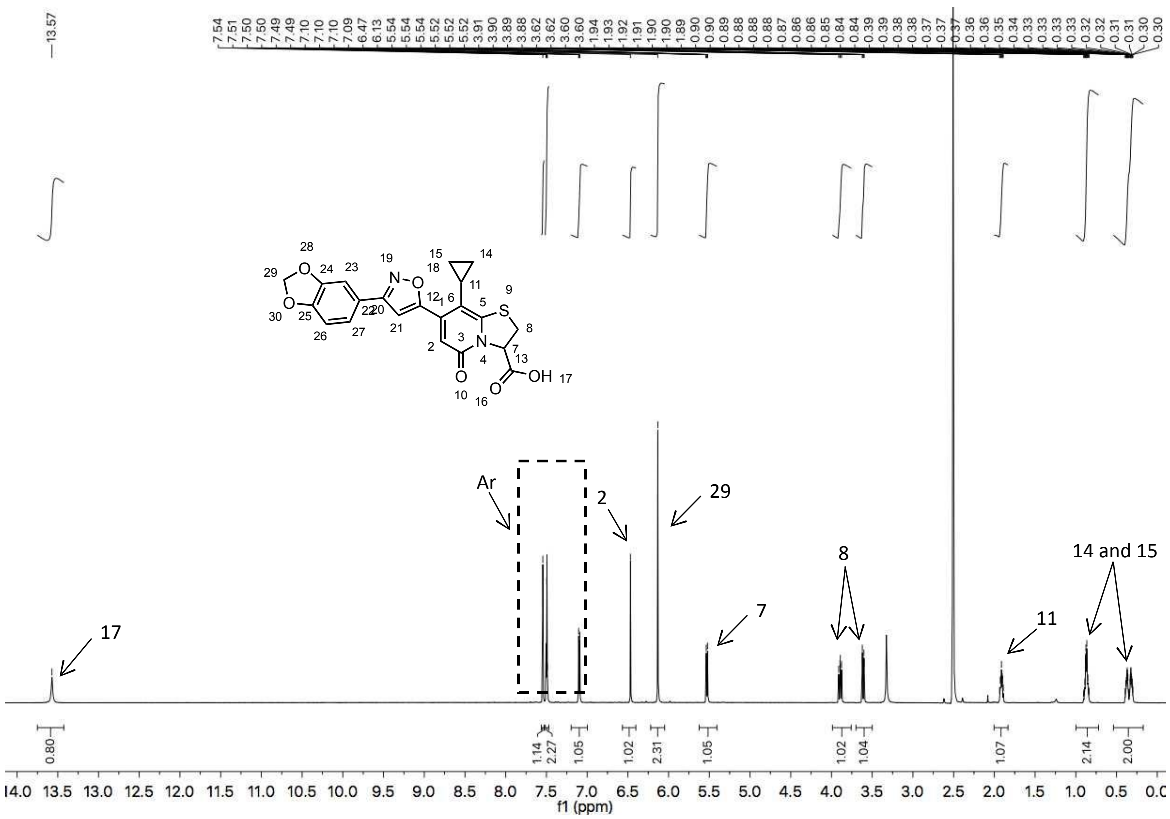

^{13}C NMR of **17o**

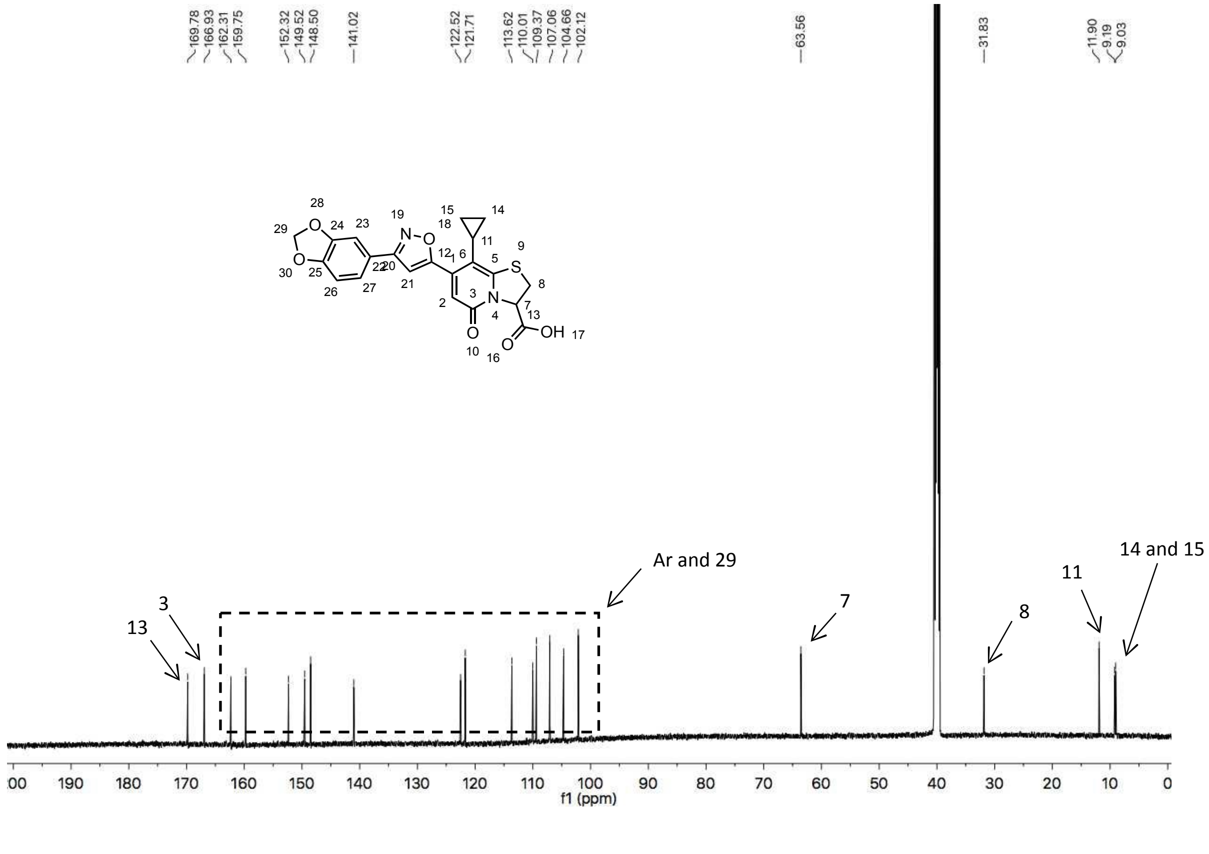

^{1}H NMR of **17p**

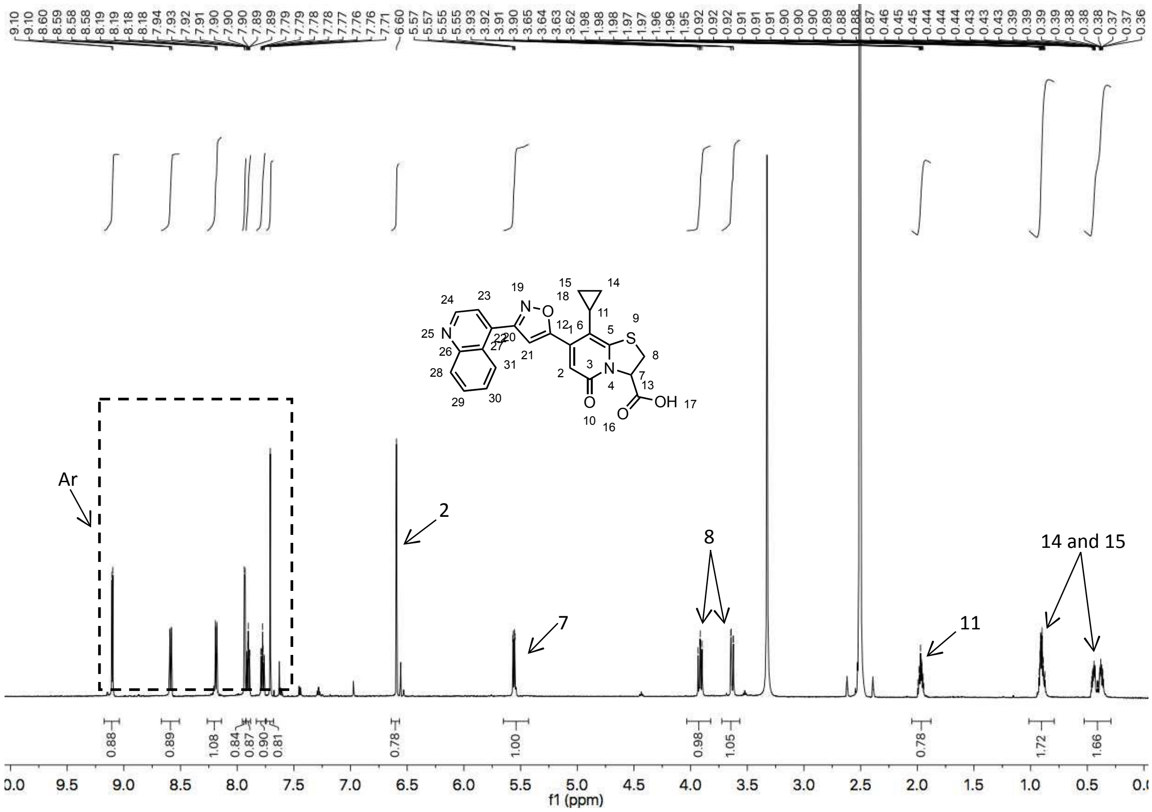

^{13}C NMR of **17p**

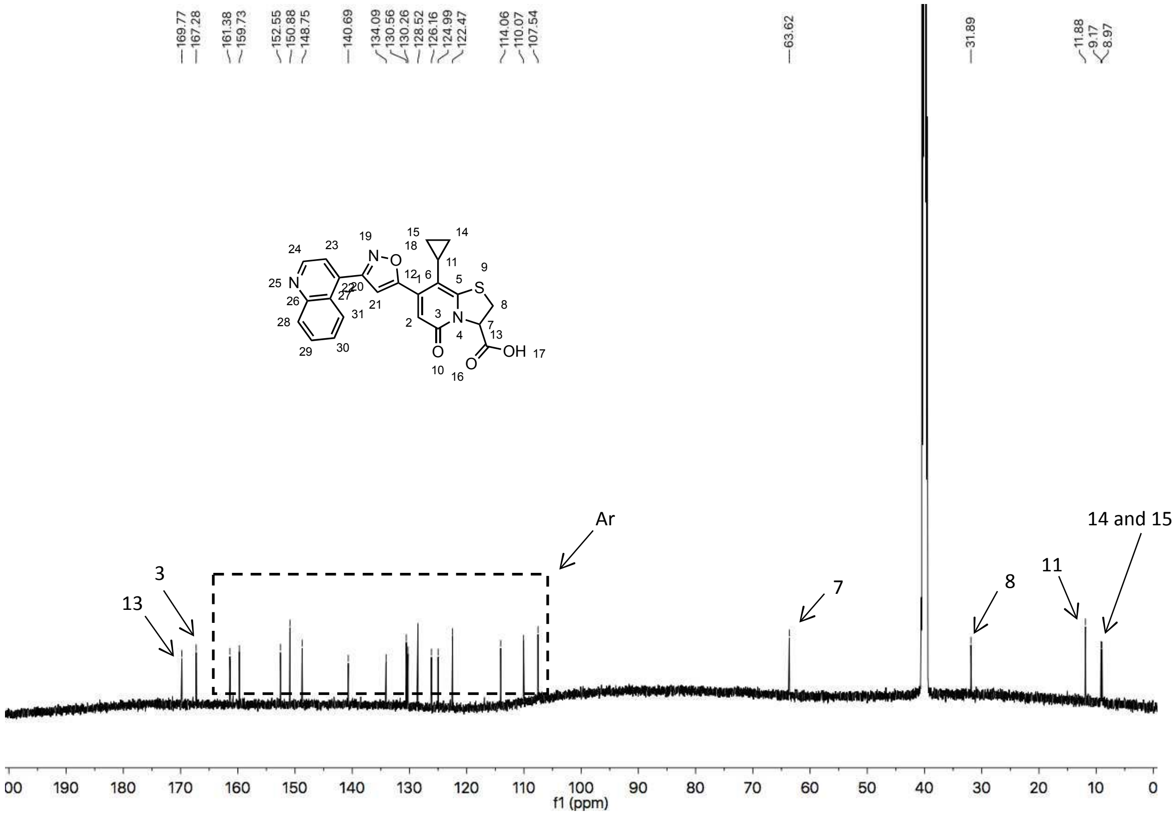

^{1}H NMR of **18a**

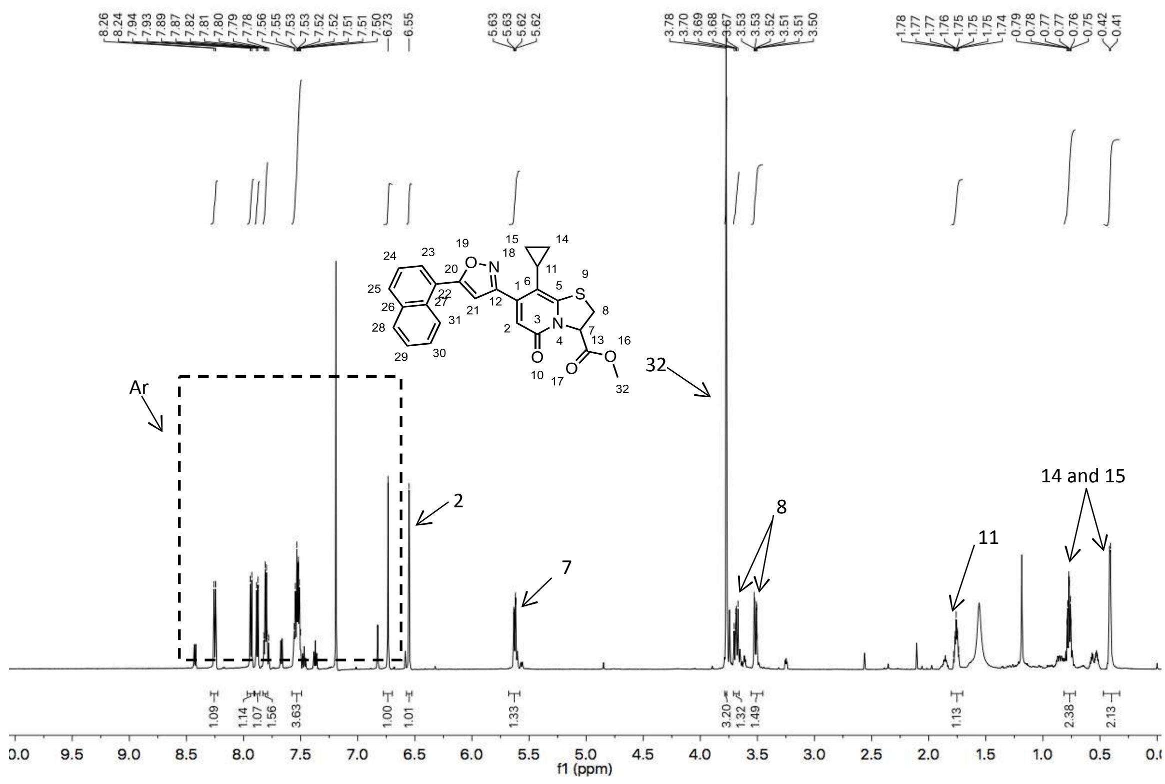

^{13}C NMR of **18a**

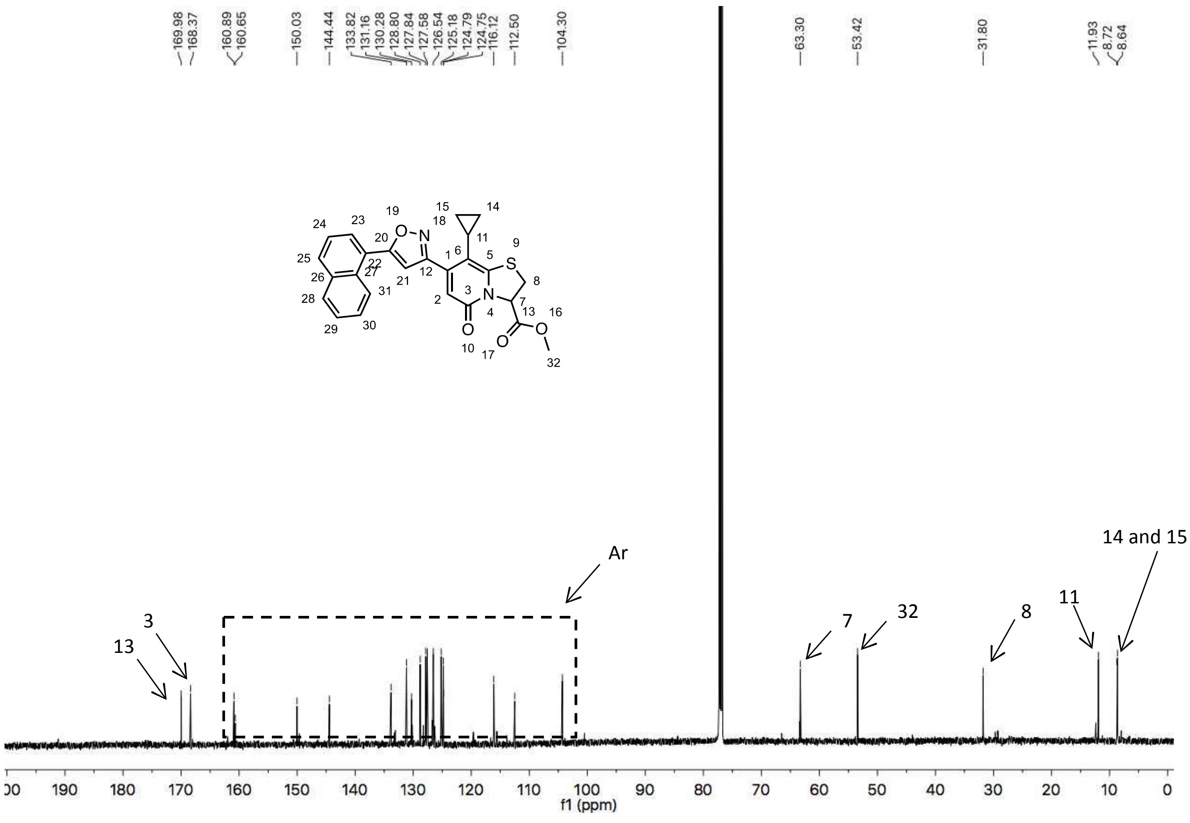

^{1}H NMR of **18b**

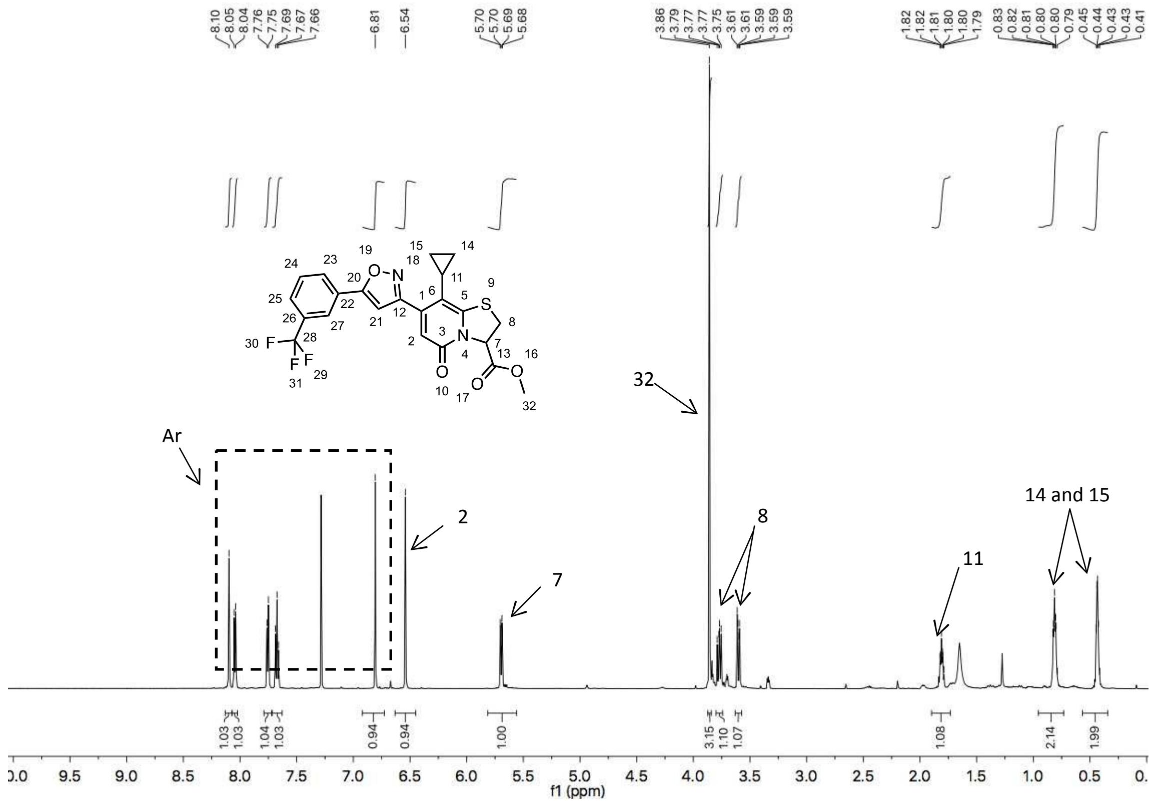

^{13}C NMR of **18b**

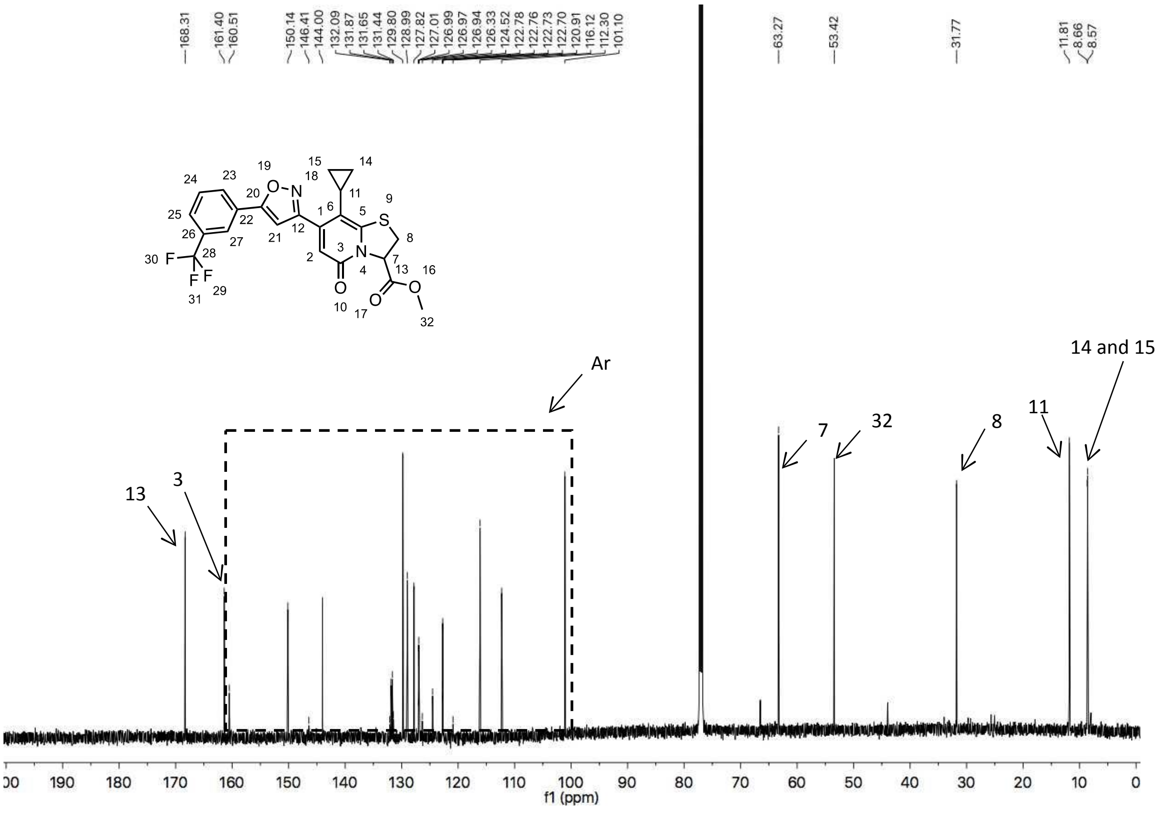

^{19}F NMR of **18b**

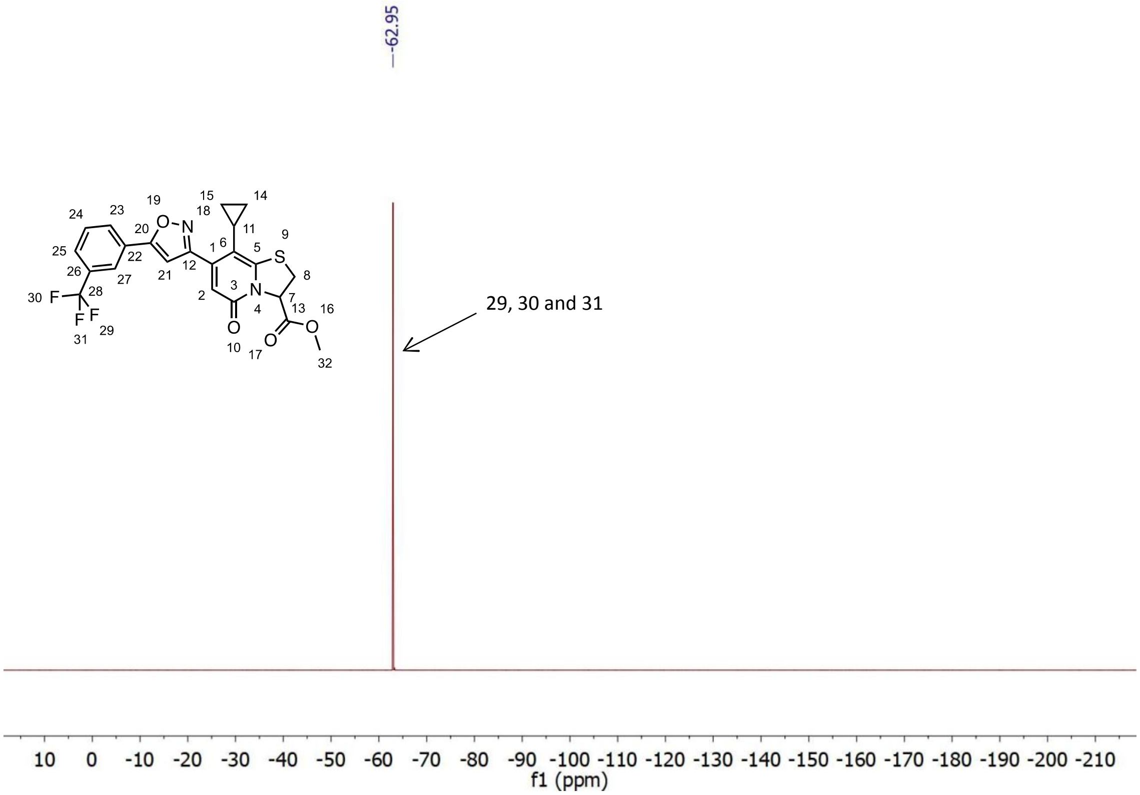

^{1}H NMR of **19a**

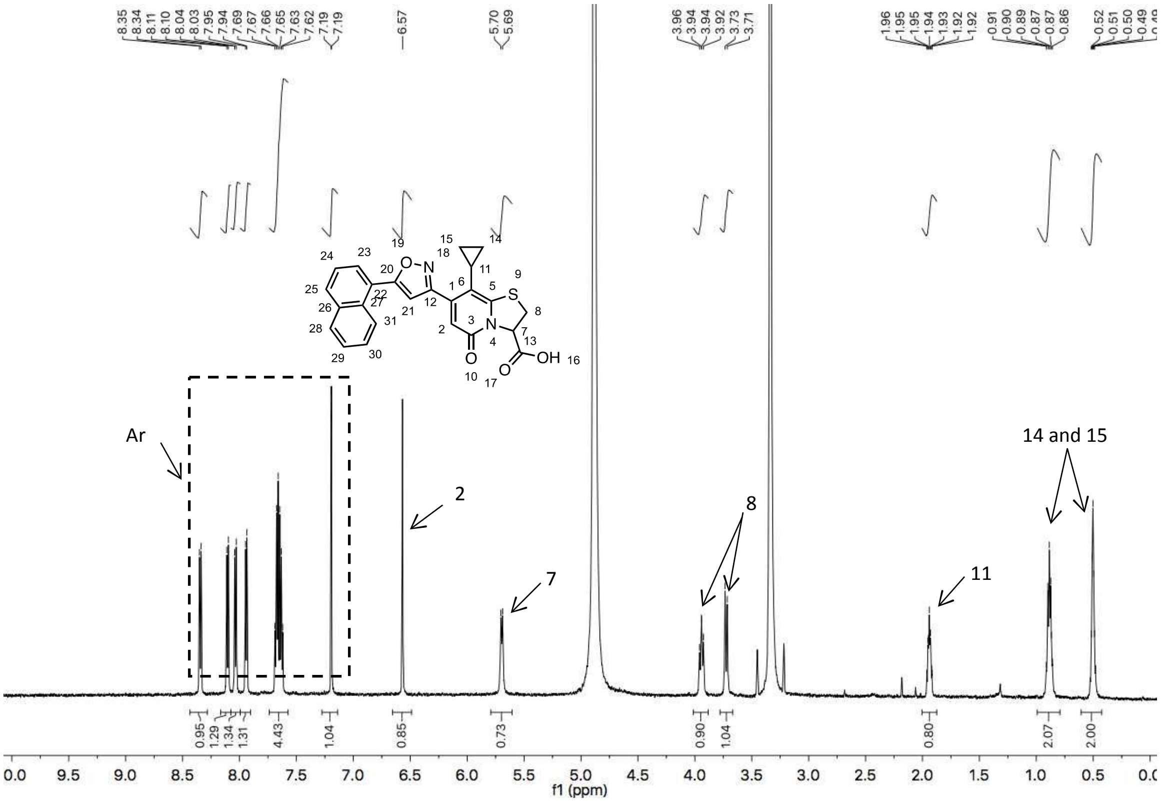

^{13}C NMR of **19a**

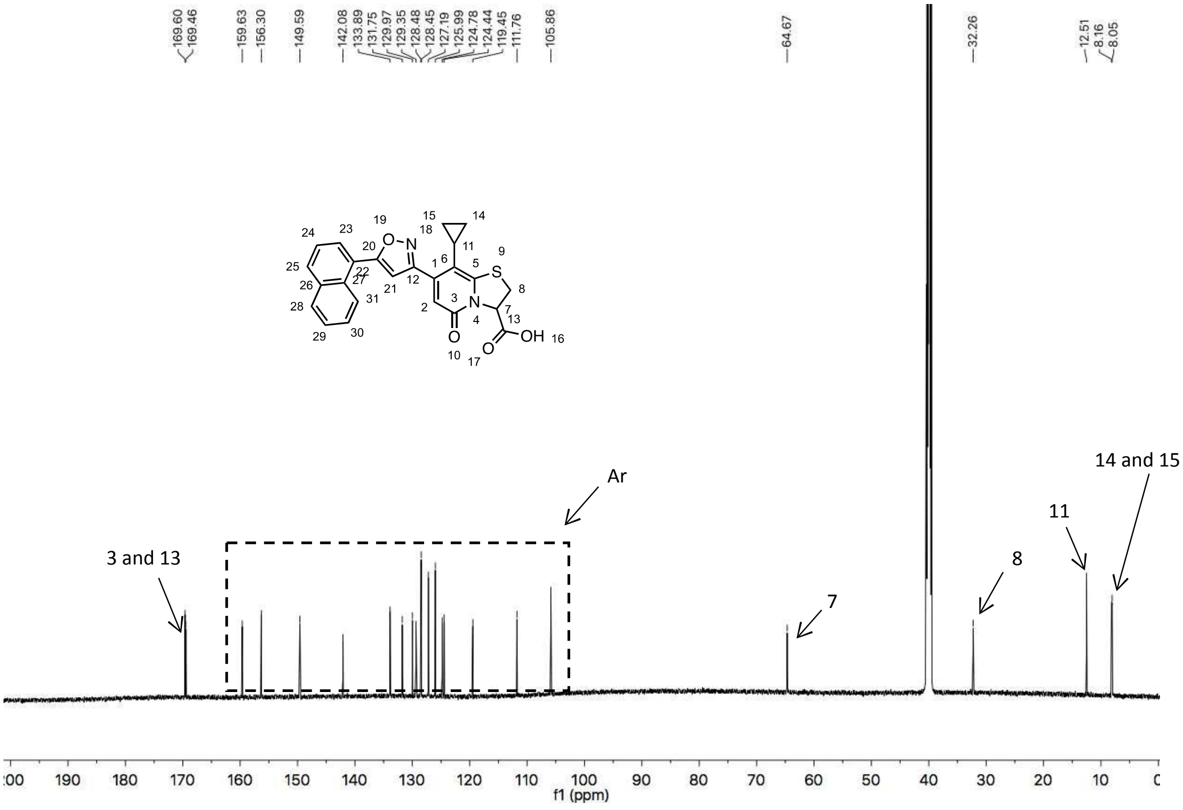

^{1}H NMR of **19b**

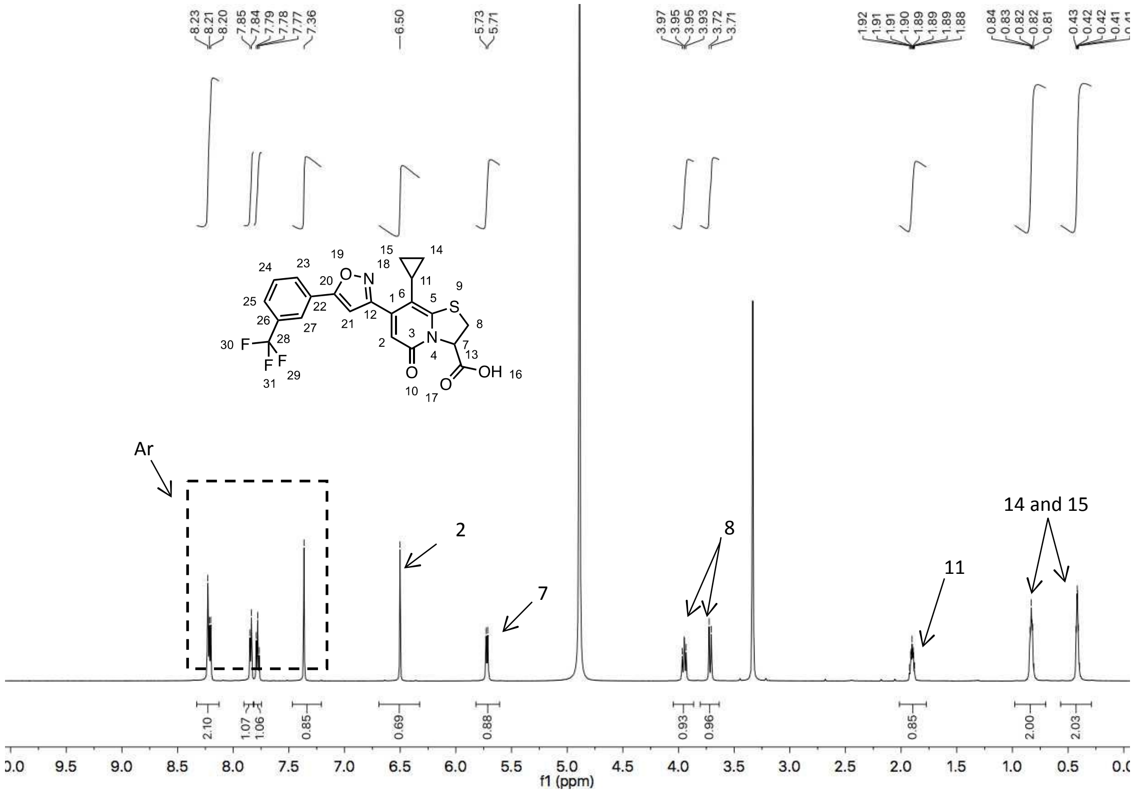

^{13}C NMR of **19b**

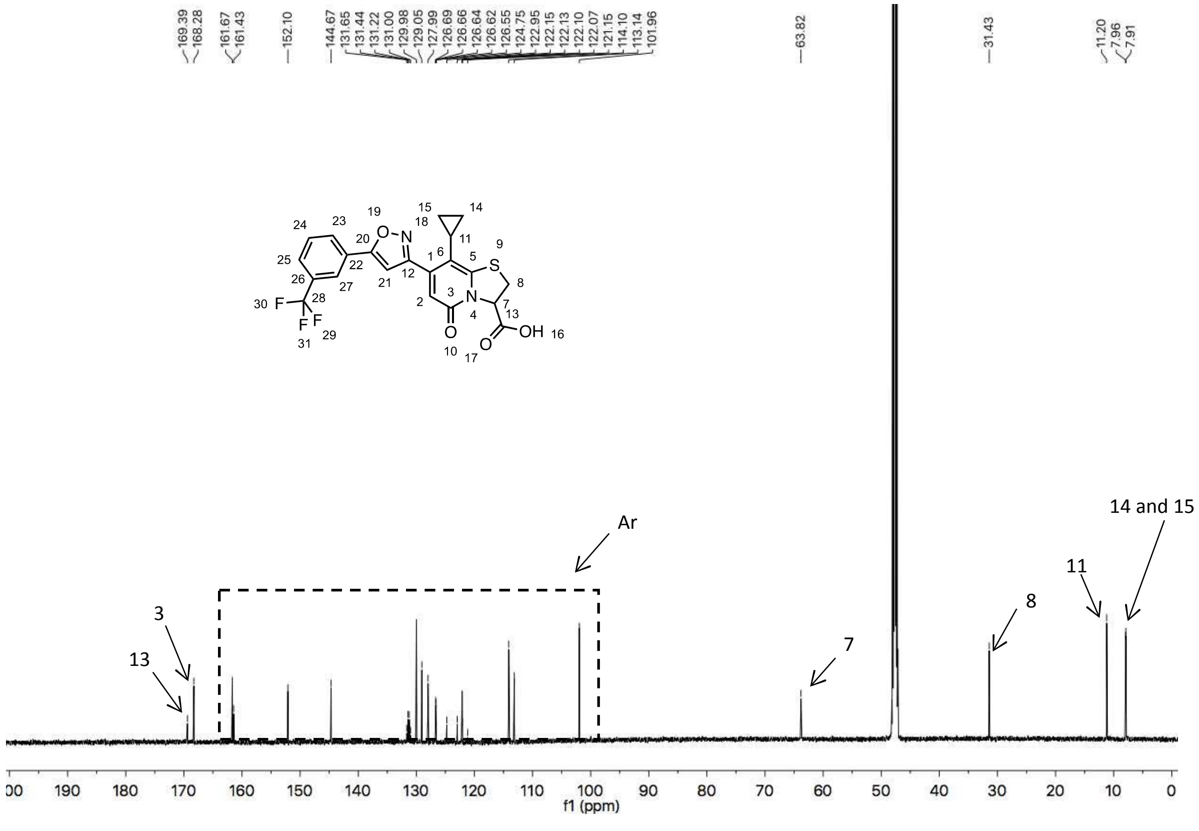

^{19}F NMR of **19b**

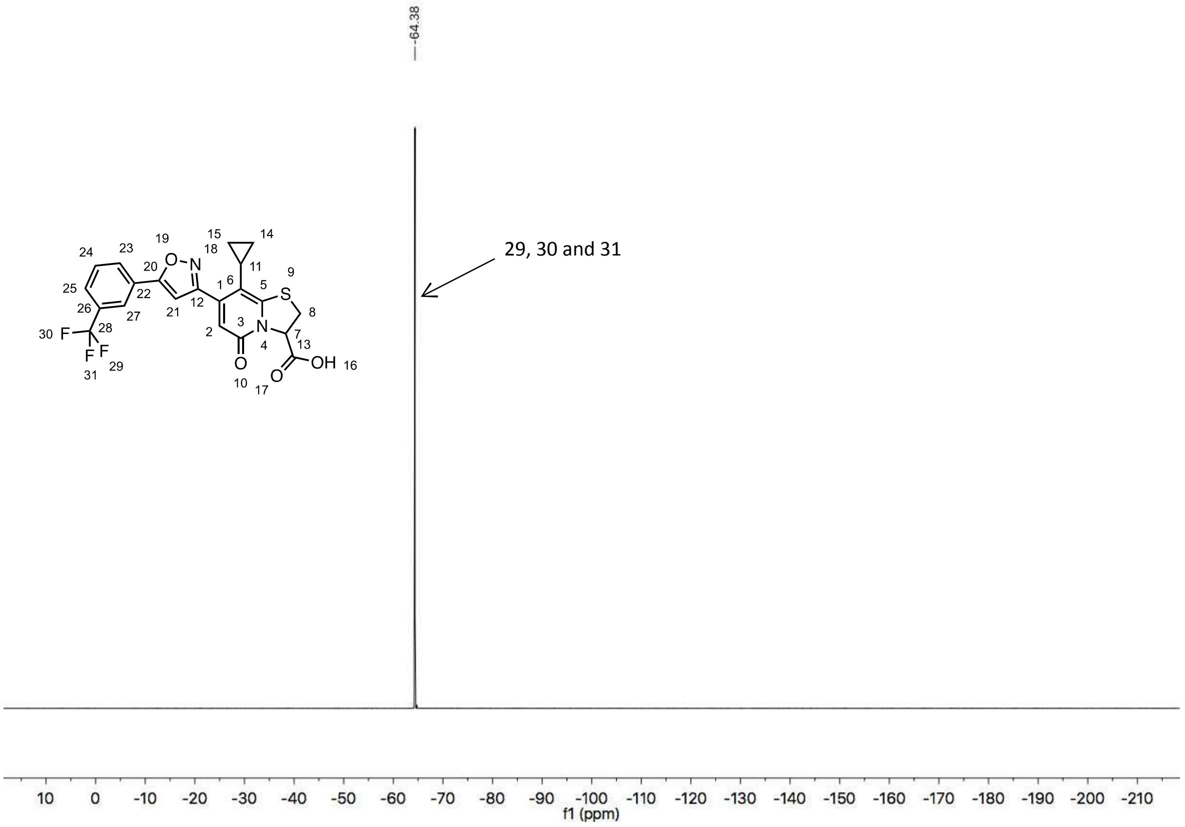

Supplement: Supplementary file 1 — jm3c00358_si_001.pdf [file jm3c00358_si_001.pdf]
